# Conformational impact of structural modifications in 2-fluorocyclohexanone

**DOI:** 10.3762/bjoc.13.172

**Published:** 2017-08-24

**Authors:** Francisco A Martins, Josué M Silla, Matheus P Freitas

**Affiliations:** 1 Department of Chemistry, Federal University of Lavras, 37200-000, Lavras, MG, Brazil

**Keywords:** classical effects, conformational analysis, 2-fluorocyclohexanone, hyperconjugation

## Abstract

2-Haloketones are building blocks that combine physical, chemical and biological features of materials and bioactive compounds, while organic fluorine plays a fundamental role in the design of performance organic molecules. Since these features are dependent on the three-dimensional chemical structure of a molecule, simple structural modifications can affect its conformational stability and, consequently, the corresponding physicochemical/biological property of interest. In this work, structural changes in 2-fluorocyclohexanone were theoretically studied with the aim at finding intramolecular interactions that induce the conformational equilibrium towards the axial or equatorial conformer. The interactions evaluated were hydrogen bonding, hyperconjugation, electrostatic and steric effects. While the gauche effect, originated from hyperconjugative interactions, does not appear to cause some preferences for the axial conformation of organofluorine heterocycles, more classical effects indeed rule the conformational equilibrium of the compounds. Spectroscopic parameters (NMR chemical shifts and coupling constants), which can be useful to determine the stereochemistry and the interactions operating in the series of 2-fluorocyclohexanone derivatives, were also calculated.

## Introduction

Organofluorine compounds are of special interest in materials, pharmaceutical and agricultural sciences, as the C–F bond is the most polar bond in organic chemistry, which can be useful in the design of performance organic molecules [1]. A fluorine substituent in organofluorine compounds affects conformational properties, since it can induce stereoelectronic effects, such as σ_C–H_ to σ*_C–F_ hyperconjugative interactions in case of an antiparallel oriented C–H bond. This is the origin of the so-called 'gauche effect', because electronegative C–X bonds do not participate in such hyperconjugative interactions and therefore often give way to the C–H bond in occupying the antiperiplanar orientation relative to the C–F bond [2–6]. The electrostatic fluorine gauche effect has also been introduced in cases where the partially negative fluorine interacts with a positive nitrogen, such as in the 3-fluoropiperidinium cation [7–13].

The carbonyl group also plays a determinant role in the conformational isomerism of ketones and aldehydes [14–17]. Here the α-carbonyl substituent can interact with the C=O bond, either through a dipolar interaction with the carbonyl oxygen or by electron delocalization (hyperconjugation) involving the good electron acceptor π*_C=O_ orbital. For example, 2-fluorocyclohexanone in the gas phase exists predominantly in the axial conformation, but the equatorial form is more stable in solution [18]. In a previous study [19], the introduction of an endocyclic oxygen in 2-fluorocyclohexanone to give the 3-fluorodihydro-2*H*-pyran-4(3*H*)-one was expected to favor the axial orientation of the fluorine substituent because of the prospective gauche arrangement with the endocyclic oxygen, giving rise to the fluorine gauche effect, but this did not appear, because of a predominant σ_C–H2_ → π*_C=O_ interaction in the equatorial conformer. The conformational induction through simple structural modifications can play an important role in the stereoselectivity of reactions (kinetically dependent on the conformation of the transition state) or in the ligand assessment to an enzyme binding site. Thus, the replacement of the endocyclic oxygen in 3-fluorodihydro-2*H*-pyran-4(3*H*)-one, as well as of the carbonyl oxygen, with other groups, can shift the conformational equilibrium of the resulting molecule towards the equatorial or the axial conformer. The axial conformer provides a gauche arrangement between the fluorine atom and the endocyclic group, while electron delocalization from electron donors, such as a σ_CH_ orbital to the vicinal π* orbital, as well as the spatial interaction between the fluorine substituent and the unsaturated bond, would also affect the conformational equilibrium.

The conformational equilibrium and other chemical/physical information for the compounds to be studied can be obtained using NMR parameters. For example, the long range ^4^*J*_H,H_ coupling constants passing through the carbonyl group in 2-bromocyclohexanone give insight on the coupling mechanism and is an experimental evidence for the hyperconjugation in this system [20]. In addition, the ^1^*J*_C,F_ coupling constant is a manifestation of the fluorine Perlin-like effect or its reversed analog, which can give information on the stereochemistry of organofluorine heterocycles [21–24].

**Figure 1:**
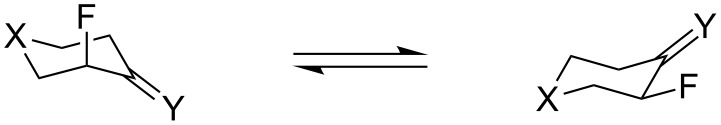
2-Fluorocyclohexanone (X = CH_2_ and Y = O) derivatives (X and Y = CH_2_, NH, O or S).

Thus, this computational study has multi-focus objectives, namely exploring the effect of replacing the carbonyl oxygen and the methylene group at position 4 by other groups in order to induce conformational changes (relative to the fluorine orientation), as well as to evaluate the interactions controlling the conformational equilibriums and to obtain insight on the spectroscopic (NMR) behavior of the studied compounds (Figure 1).

## Results and Discussion

The axial population of 2-fluorocyclohexanone in the gas phase has been found to be 64%, decreasing to only 2% in DMSO solution [18], which is in agreement with our theoretical findings (Table 1). This behavior is comprehensive, because the equatorial conformer has the C=O and C–F bonds in the same plane and similarly orientated. This should increase the molecular dipole moment, and favor the axial isomer. Because the two polar vectors are approximately at right angles, the electrostatic repulsion in the equatorial should be significantly relaxed in a high dielectric continuum.

Modifications of X and Y in the 2-fluorocyclohexanone framework of Figure 1 should cause conformational changes, as the intramolecular forces, as well as the solvent effect, are affected by the different groups (CH_2_, NH, O and S) attached to these positions. Indeed, diverse conformational behaviors are observed when changing X and Y (Table 1). Nevertheless, it is worth noting that, in general, the axial population increases in DMSO solution relative to the gas phase only in compounds with Y = CH_2_ (the population does not vary with the solvent when X = CH_2_ and NH_ax_). In these cases, the axial conformer has similar or larger molecular dipole moments than the equatorial form (differently from the other compounds). In addition, the π*_C=Y_ orbitals (Y = O, S and N) are better electron acceptors than the π*_C=C_ in electron delocalization processes. These highlight the role of electrostatic and hyperconjugative effects on the conformational isomerism of the studied compounds.

**Table 1:** Gibbs population (%) for the axial conformer (at 298 K and 1 atm) in vacuum/DMSO.^a^

Y/X	X = CH_2_	X = O	X = S	X = NH_ax_	X = NH_eq_

Y = CH_2_	55/55	40/84	12/52	83/79	12/53
	(2.0/2.5)	(2.7/1.8)	(2.8/1.9)	(1.4/2.3)	(2.8/2.5)
Y = O	65/5	42/14	21/4	88/11	15/4
	(3.1/4.9)	(2.0/3.3)	(1.5/3.2)	(2.1/4.4)	(3.6/4.7)
Y = S	78/10	53/22	39/13	95/24	26/9
	(2.8/4.4)	(1.7/2.9)	(1.3/2.7)	(1.9/4.1)	(3.2/4.3)
Y = NH*_Z_*	3/3	2/10	0/2	10/9	0/3
	(2.5/3.4)	(1.9/1.7)	(1.7/1.6)	(1.4/3.0)	(3.1/3.3)
Y = NH*_E_*	98/52	95/72	89/54	100/71	81/44
	(3.0/4.6)	(2.7/3.6)	(2.4/3.5)	(2.2/4.3)	(3.5/4.5)

^a^Molecular dipole moments for the axial/equatorial conformers in vacuum are given in parenthesis, second entries, in Debye.

Two particularly clear trends are related to the NH group at the Y position in vacuum: while the NH*_Z_* leads to a strong shift toward the equatorial conformer for compounds with any X, compounds with NH*_E_* at the Y position strongly favor the axial conformer. It is intuitive that the equatorial conformer of compounds with Y = NH*_Z_* experience intramolecular hydrogen bond F···HN (e.g., *d*_F···HN_ = 2.21 Å and F···H–N angle = 110.0°, when X/Y = CH_2_/NH*_Z_*), despite similar interactions hardly ever appear in organofluorine compounds when forming 5-membered rings [25–26]. In addition, it is also quite intuitive that fluorine tends to occupy the axial orientation in compounds with Y = NH*_E_*, as the nitrogen electron lone pair is directed toward the equatorial fluorine, thus causing electronic repulsion.

Other noticeable findings in the vacuum are those related to the compounds with endocyclic CH_2_ and NH_ax_ groups (X). Most compounds in this series (except those with NH*_Z_* as Y, which exhibit intramolecular hydrogen bond with the equatorial fluorine) show axial prevalence. On the other hand, the equatorial conformer is preferred when X = S and NH_eq_ (except for Y = NH*_E_*). While the axial preference for compounds with X = NH_ax_ can be determined by an electrostatic, dipolar relaxation F···H_ax_N (since an intramolecular hydrogen bond has not been clearly demonstrated in 3-fluoropiperidine [7–13]), such behavior for compounds with X = CH_2_ seems to be dependent on the C=Y bond, since X = CH_2_ is not a good proton donor. In this case, the axial population increases according to Y = CH_2_ < O < S, since the π*_C=C_ is a bad electron-acceptor orbital in σ_C–H2ax_ → π*_C=Y_ electron delocalizations and the classical steric/electrostatic repulsion of O and S with the equatorial fluorine is remarkable. This is corroborated by the Lewis and non-Lewis energies obtained by the natural orbital bond (NBO) decomposition analysis of Table 2 (which shows that the axial conformer is less favored by the Lewis term in Y = S than O), and also by the relatively low value for the σ_C–H2ax_ → π*_C=C_ interaction given in Table 3. It is worth mentioning that the σ_C–H2ax_ → π*_C=O_ and σ_C–H2ax_ → π*_C=S_ interactions are more stabilizing than σ_C–H2ax_ → π*_C=C_, but they are also accompanied by strong steric and electrostatic interactions (see the high values for both Lewis and non-Lewis terms when Y = O and S compared to CH_2_). When the X = S and NH_eq_ groups take place, the equatorial conformer predominates, as an axial hydrogen at X (either from the CH_2_ or NH_ax_) is replaced by an electron lone pair (LP), giving rise to F_ax···_LP_X_ repulsion.

**Table 2:** Relative electronic energies (Δ*E*_ax–eq_) from NBO analysis (total, Lewis and non-Lewis energies, in kcal mol^−1^) in vacuum.

X	Y	Total	Lewis	non-Lewis

CH_2_	CH_2_	−0.1	−3.7	3.6
CH_2_	O	−0.5	−10.4	9.9
CH_2_	S	−0.9	−8.1	7.2
CH_2_	NH*_Z_*	2.2	−3.7	5.9
CH_2_	NH*_E_*	−2.5	−10.1	7.6
O	CH_2_	0.3	1.5	−1.2
O	O	0.2	−4.7	4.9
O	S	−0.1	−2.9	2.9
O	NH*_Z_*	2.7	1.9	0.8
O	NH*_E_*	−1.8	−4.8	2.9
S	CH_2_	1.5	1.7	−0.2
S	O	0.9	−4.8	5.7
S	S	0.3	−2.4	2.7
S	NH*_Z_*	3.8	1.7	2.1
S	NH*_E_*	−1.1	−4.8	3.7
NH_ax_	CH_2_	−1.1	−3.8	2.7
NH_ax_	O	−1.4	−10.1	8.6
NH_ax_	S	−1.9	−7.5	5.7
NH_ax_	NH*_Z_*	1.2	−3.7	4.9
NH_ax_	NH*_E_*	−3.3	−10.1	6.8
NH_eq_	CH_2_	1.3	2.4	−1.1
NH_eq_	O	1.2	−3.5	4.7
NH_eq_	S	0.7	−1.8	2.5
NH_eq_	NH*_Z_*	3.7	2.5	1.2
NH_eq_	NH*_E_*	−1.0	−3.9	2.9

**Table 3:** Important antiperiplanar hyperconjugative interactions (given for axial/equatorial, in kcal mol^−1^).

X	Y	σ_C–H3_ → σ*_C-F_	σ_C–F_ → σ*_C–H3_	σ_C–H3_ → σ*_C–X_	σ_C–X_ → σ*_C–H3_

CH_2_	CH_2_	6.3/–	1.2/–	3.3/–	2.0/–
CH_2_	O	6.3/–	1.1/–	3.1/–	2.0/–
CH_2_	S	6.2/–	1.1/–	3.1/–	2.0/–
CH_2_	NH*_Z_*	6.4/–	1.1/–	3.1/–	2.0/–
CH_2_	NH*_E_*	6.3/–	1.2/–	3.2/–	2.0/–
O	CH_2_	5.8/–	1.0/–	4.4/–	0.9/–
O	O	5.7/–	1.0/–	4.2/–	0.9/–
O	S	5.5/–	1.0/–	4.4/–	0.9/–
O	NH*_Z_*	5.9/–	0.9/–	4.3/–	0.9/–
O	NH*_E_*	5.7/–	1.1/–	4.4/–	0.9/–
S	CH_2_	6.0/–	1.0/–	5.8/–	2.3/–
S	O	5.9/–	1.0/–	5.4/–	2.3/–
S	S	5.8/–	0.9/–	5.4/–	2.3/–
S	NH*_Z_*	6.0/–	0.9/–	5.6/–	2.4/–
S	NH*_E_*	6.0/–	1.0/–	5.8/–	2.3/–
NH_ax_	CH_2_	5.8/–	1.1/–	4.0/–	1.2/–
NH_ax_	O	5.6/–	1.1/–	3.7/–	1.2/–
NH_ax_	S	5.4/–	1.0/–	3.6/–	1.1/–
NH_ax_	NH*_Z_*	5.9/–	1.0/–	3.8/–	1.2/–
NH_ax_	NH*_E_*	5.8/–	1.1/–	3.8/–	1.2/–
NH_eq_	CH_2_	6.1/–	1.0/–	3.7/–	1.2/–
NH_eq_	O	6.1/–	1.0/–	3.5/–	1.2/–
NH_eq_	S	5.9/–	1.0/–	3.5/–	1.2/–
NH_eq_	NH*_Z_*	6.2/–	1.0/–	3.5/–	1.3/–
NH_eq_	NH*_E_*	6.1/–	1.0/–	3.6/–	1.2/–

X	Y	σ_C–X_ → σ*_C–F_	σ_C–F_ → σ*_C–X_	σ_C–H2_ → π*_C=Y_	σ_C1–C2_ → σ*_C–X_^a^

CH_2_	CH_2_	–/3.8	–/1.1	–/6.2	–/–
CH_2_	O	–/3.7	–/1.1	–/7.2	–/–
CH_2_	S	–/3.5	–/1.1	–/8.5	–/–
CH_2_	NH*_Z_*	–/4.0	–/1.1	–/6.5	–/–
CH_2_	NH*_E_*	–/3.6	–/1.1	–/6.5	–/–
O	CH_2_	–/2.0	–/1.4	–/6.2	–/–
O	O	–/1.8	–/1.3	–/7.4	0.6/–
O	S	–/1.7	–/1.3	–/8.6	–/–
O	NH*_Z_*	–/2.0	–/1.3	–/6.6	–/–
O	NH*_E_*	–/1.8	–/1.4	–/6.7	0.5/–
S	CH_2_	–/4.5	–/1.8	–/5.5	–/–
S	O	–/4.3	–/1.7	–/6.5	–/–
S	S	–/4.1	–/1.7	–/7.3	–/–
S	NH*_Z_*	–/4.7	–/1.7	–/5.9	–/–
S	NH*_E_*	–/4.2	–/1.8	–/5.8	–/–
NH_ax_	CH_2_	–/2.3	–/1.3	–/6.2	–/–
NH_ax_	O	–/2.1	–/1.3	–/7.2	–/–
NH_ax_	S	–/2.0	–/1.3	–/8.4	–/–
NH_ax_	NH*_Z_*	–/2.4	–/1.3	–/6.5	–/–
NH_ax_	NH*_E_*	–/2.1	–/1.3	–/6.5	–/–
NH_eq_	CH_2_	–/2.7	–/1.2	–/6.1	–/–
NH_eq_	O	–/2.6	–/1.1	–/7.4	–/–
NH_eq_	S	–/2.4	–/1.1	–/8.5	–/–
NH_eq_	NH*_Z_*	–/2.8	–/1.1	–/6.5	–/–
NH_eq_	NH*_E_*	–/2.5	–/1.2	–/6.6	–/–

^a^The reciprocal interaction σ_C–X_ → σ*_C1–C2_ was not observed for any system.

For compounds with X other than the NH group (which exhibit specific interactions), the axial population increases according to X = S < O < CH_2_, as a clear effect of size and/or electronegativity rather than hyperconjugation, thus revealing the steric and/or electrostatic nature of the interactions between the gauche X and F_ax_ groups. The opposite effect is observed for Y = S, O and CH_2_, i.e., the axial population increases according to Y = CH_2_ < O < S, since the repulsion with the F_eq_ substituent increases in this order. The hypothesis based on steric and electrostatic interactions is supported by the increase of the axial population from Y = CH_2_ to S, despite the better electron-acceptor ability of the π*_C=S_ in comparison to π*_C=O_ and π*_C=C_, e.g., in the σ_C–Hax_ → π*_C=Y_ interactions for the equatorial conformer (see NBO electron delocalization energies of Table 3).

The gauche effect does not appear to be induced by hyperconjugation involving the endocyclic X, even when X = O, such as in most cases where this phenomenon is observed. Differently from acyclic organofluorine compounds, hyperconjugation is not sufficiently effective in organofluorine heterocycles because they do not have enough antiperiplanar C–H bonds as electron donors to participate in σ_C–H_ → σ*_C–F_ and σ_C–H_ → σ*_C–X_ electron-delocalization processes. Intramolecular hydrogen bonding, when possible, is a better source of stabilization, but the interaction of F with Y (or the C=Y bond) appears to be more relevant as the controlling effect of the conformational isomerism of the studied compounds. Thus, classical effects explain satisfactorily the conformational behavior of the studied compounds, according to the NBO analysis and in spite of some stabilizing electronic delocalization interactions from electron-donor orbitals to antiperiplanar antibonding orbitals.

The electronic interactions mentioned above appeared to be sensitive to the solvent effect, since the population of the equatorial conformer increased with the solvent polarity, as dipolar repulsion between Y and F decreases in solvents with increasing dielectric constants.

Nuclear magnetic resonance (NMR) parameters, namely chemical shifts and coupling constants, can be useful to provide information on conformational population and intramolecular interactions. The ^3^*J*_H,H_ coupling constant has been widely used to estimate conformers population in an equilibrium [18], because of its dependence with the H–C–C–H dihedral angle, according to the Karplus curve. Chemical shifts can also be used, but they are more sensitive to solvent changes [27]. In the present study, the calculated H-1 and F-19 chemical shifts (Table 4) are too close for both conformers to differentiate them by comparing the absolute (calculated) and the mean experimental values. Also, the calculated long-range coupling constants ^4^*J*_H,H_ are too small to infer something about any coupling pathway involving hyperconjugative interaction via the C=Y bond, as in 2-bromocyclohexanone [20]. However, the ^1^*J*_C,F_ coupling constant varies significantly from one conformer to the other, which can give insight either into conformer populations or intramolecular interactions operating in these systems. Since most |^1^*J*_C,F_| values in Table 4 are larger for the equatorial conformer (except for the compound with X = NH_eq_ and Y = NH*_Z_*), the fluorine Perlin-like effect takes place. The literature [21–24] has attributed such effect to the interaction between the fluorine atom (or the C–F bond) and the Y group (or the C=Y bond), which has been well described by a spatial/dipolar interaction between these groups, despite a minor contribution from hyperconjugation. Thus, the fluorine Perlin-like effect shown in Table 4 supports our conclusions that classical effects govern the conformational equilibrium of the studied series of compounds.

**Table 4:** Calculated NMR parameters (*J* in Hz and δ in ppm, relative to TMS for ^1^H and TFA for ^19^F) in vacuum.

X	Y	^1^*J*_C,Fax_	^1^*J*_C,Feq_	^4^*J*_H2eq,H6eq_	^4^*J*_H2eq,H6ax_	^4^*J*_H2ax,H6eq_	^4^*J*_H2ax,H6ax_	δ_H2ax_	δ_H2eq_	δ_Fax_	δ_Feq_

CH_2_	CH_2_	–171.2	−192.2	0.3	−0.7	−0.6	−0.7	4.8	4.8	−111.1	−119.2
CH_2_	O	−181.8	−205.0	0.6	−0.5	−0.4	1.4	4.4	4.7	−123.0	−127.0
CH_2_	S	−184.5	−200.8	0.4	−0.7	−0.4	0.0	5.0	5.3	−103.9	−101.9
CH_2_	NH*_Z_*	−181.0	−189.3	0.6	−0.6	−0.5	0.1	4.3	4.7	−114.2	−125.9
CH_2_	NH*_E_*	−173.1	−204.7	0.5	−0.6	−0.4	0.2	4.9	4.7	−121.5	−123.6
O	CH_2_	−180.3	−194.6	0.4	−0.7	−0.7	−0.9	4.5	4.9	−113.7	−138.3
O	O	−189.6	−207.2	0.7	−0.5	−0.5	1.3	4.2	4.8	−126.8	−144.0
O	S	−192.5	−203.2	0.6	−0.6	−0.5	−0.3	4.8	5.3	−107.6	−120.3
O	NH*_Z_*	−189.3	−192.2	0.7	−0.6	−0.6	0.0	4.0	4.7	−118.2	−144.1
O	NH*_E_*	−181.7	−206.6	0.7	−0.6	−0.5	0.1	4.6	4.8	−124.7	−142.1
S	CH_2_	−181.7	−195.8	0.1	−0.7	−0.5	−0.6	4.8	4.9	−110.4	−110.1
S	O	−192.8	−209.2	0.4	−0.4	−0.3	1.2	4.4	4.8	−122.0	−118.2
S	S	−195.3	−204.6	0.2	−0.7	−0.4	−0.1	5.1	5.4	−102.7	−93.1
S	NH*_Z_*	−191.8	−193.0	0.4	−0.5	−0.4	0.1	4.4	4.8	−113.5	−117.4
S	NH*_E_*	−183.7	−208.7	0.3	−0.5	−0.4	0.2	4.9	4.9	−120.7	−114.6
NH_ax_	CH_2_	−169.6	−199.9	0.2	−0.7	−0.6	−0.7	4.5	4.6	−116.1	−128.7
NH_ax_	O	−179.4	−212.5	0.5	−0.5	−0.4	1.4	4.3	4.5	−126.9	−135.3
NH_ax_	S	−182.8	−208.2	0.4	−0.7	−0.5	0.0	4.9	5.1	−106.8	−110.7
NH_ax_	NH*_Z_*	−179.0	−197.4	0.5	−0.6	−0.5	0.1	4.1	4.5	−119.0	−135.3
NH_ax_	NH*_E_*	−170.8	−211.8	0.4	−0.6	−0.5	0.2	4.7	4.5	−126.2	−132.9
NH_eq_	CH_2_	−181.8	−190.2	0.5	−0.7	−0.6	−0.9	4.6	4.9	−112.0	−130.8
NH_eq_	O	−191.5	−202.9	0.8	−0.5	−0.5	0.1	4.2	4.8	−125.5	−137.4
NH_eq_	S	−194.1	−198.9	0.6	−0.6	−0.5	−0.4	4.8	5.4	−106.2	−113.6
NH_eq_	NH*_Z_*	−191.0	−187.6	0.8	−0.6	−0.6	−0.1	4.2	4.8	−116.5	−137.1
NH_eq_	NH*_E_*	−183.2	−202.5	0.7	−0.6	−0.5	0.0	4.7	4.8	−123.3	−134.8

## Conclusion

Hyperconjugation plays a secondary role as dictating interaction of the conformational isomerism of 2-fluorocyclohexanone derivatives investigated in this study. The more classical steric and electrostatic effects (and hydrogen bonding, when possible) should be invoked as the main interactions governing the conformational equilibrium of these compounds. The gauche effect does not appear to be due to hyperconjugation, despite some preferences for the axial conformation (in which the fluorine atom has a gauche arrangement relative to X), because of a lack of stabilizing σ_C–H_ → σ*_C–F_ and σ_C–H_ → σ*_C–X_ interactions. The fluorine Perlin-like effect observed for calculated ^1^*J*_C,F_ coupling constants confirms these findings.

## Computational Methods

Optimization and frequency calculations were performed at the ωB97X-D/6-311++g(d,p) [28–29] level (thus including empirical dispersion effects) to obtain the Gibbs free energies (1.00 atm and 298.15 K). Natural orbital bond (NBO) analyses were carried out for the optimized structures at the same level of theory using the NBO 6.0 program [30], in order to obtain the electronic delocalization energies, as well as the Lewis (steric and electrostatic interactions) and non-Lewis (electron delocalization) contributions through the NOSTAR keyword. Chemical shifts and spin–spin coupling constant calculations were also performed at the ωB97X-D/6-311+g(d,p) level. All the calculations were processed using the Gaussian 09 program [31] for the gas phase and implicit DMSO (using the polarizable continuum model [32]).

## Supporting Information

File 1 Computational data.
