# Supplementary material for: Conformational impact of structural modifications in 2-fluorocyclohexanone
Source: Beilstein J Org Chem. 2017 Aug 24;13:1781–7. doi: 10.3762/bjoc.13.172 (PMC5588668; doi:10.3762/bjoc.13.172)
Supplement: File 1 — Computational data. [file Beilstein_J_Org_Chem-13-1781-s001.pdf]

Supporting Information

for

Conformational impact of structural modifications in 2-fluorocyclohexanone

Francisco A. Martins, Josué M. Silla and Matheus P. Freitas*

Address: Department of Chemistry, Federal University of Lavras, 37200-000, Lavras, MG, Brazil

Email: Matheus P. Freitas - matheus@dqi.ufla.br

*Corresponding author

Computational data

Table of Contents:

Table S1. Compounds studied in this work S2

Standard orientation for compounds **1a**–**25e** in the gas phase S3

Standard orientation for compounds **1a**–**25e** in implicit DMSO S20

Harmonic frequencies calculated for the structures **1a**–**25e** in the gas phase S37

Harmonic frequencies calculated for the structures **1a**–**25e** in implicit DMSO S108

Table S1. Compounds studied in this work

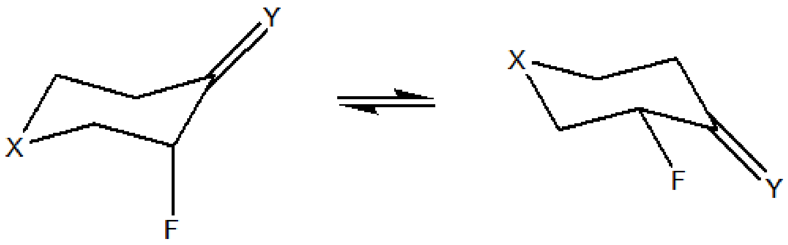

	Y = O	Y = S	Y = NH_Z	Y = NH_E	Y = CH_2
X = O	1a and 1e	2a and 2e	3a and 3e	4a and 4e	5a and 5e
X = S	6a and 6e	7a and 7e	8a and 8e	9a and 9e	10a and 10e
X = NH_{ax}	11a and 11e	12a and 12e	13a and 13e	14a and 14e	15a and 15e
X = NH_{eq}	16a and 16e	17a and 17e	18a and 18e	19a and 19e	20a and 20e
X = CH_2	21a and 21e	22a and 22e	23a and 23e	24a and 24e	25a and 25e

Gas phase

1a Standard orientation:

Center Number	Atomic Number	Atomic Type	Coordinates (Angstroms) X	Y	Z
1	6	0	1.240001	1.266690	0.037134
2	6	0	-0.149394	1.307063	0.700678
3	6	0	-1.058184	0.393506	-0.078436
4	6	0	-0.479860	-1.011476	-0.248379
5	6	0	0.933745	-0.901294	-0.809359
6	1	0	-0.056880	0.934594	1.724958
7	1	0	-0.558595	2.317992	0.710946
8	1	0	1.183814	1.708513	-0.969466
9	1	0	1.958363	1.839814	0.622720
10	1	0	-1.125502	-1.612045	-0.892146
11	1	0	1.410189	-1.882285	-0.805225
12	1	0	0.864162	-0.545187	-1.850005
13	8	0	1.741974	-0.048715	-0.037417
14	9	0	-0.429169	-1.613880	1.006827
15	8	0	-2.083332	0.728288	-0.614215

1e Standard orientation:

Center Number	Atomic Number	Atomic Type	Coordinates (Angstroms) X	Y	Z
1	6	0	-0.263937	-1.425759	-0.260106
2	6	0	0.800410	-0.540611	0.409087
3	6	0	0.519312	0.921449	0.089539
4	6	0	-0.919438	1.307511	0.359718
5	6	0	-1.862402	0.283038	-0.285696
6	1	0	0.752896	-0.677374	1.496010
7	1	0	-0.141973	-1.362229	-1.351254
8	1	0	-0.130740	-2.463225	0.045439
9	1	0	-1.094438	2.313690	-0.024327
10	1	0	-2.895414	0.468204	0.007807
11	1	0	-1.797273	0.352895	-1.381210
12	8	0	-1.553829	-1.029986	0.133309
13	8	0	1.346276	1.662711	-0.368511
14	1	0	-1.088411	1.307279	1.442021
15	9	0	2.045790	-0.919423	-0.025347

2a Standard orientation:

Center Number	Atomic Number	Atomic Type	Coordinates (Angstroms) X	Y	Z
1	6	0	1.147256	1.545925	0.006139
2	6	0	-0.028634	1.121587	0.910467
3	6	0	-0.773317	0.036820	0.198382
4	6	0	0.129718	-1.119197	-0.169012
5	6	0	1.306552	-0.576176	-0.982133
6	1	0	0.384735	0.728328	1.844579
7	1	0	0.754030	2.002678	-0.914508
8	1	0	1.772981	2.276256	0.518781
9	1	0	-0.410203	-1.888663	-0.722716
10	1	0	2.031467	-1.373360	-1.150426
11	1	0	0.921701	-0.229185	-1.953742
12	8	0	1.979406	0.453531	-0.305064
13	1	0	-0.678835	1.969624	1.125155
14	16	0	-2.306036	0.131219	-0.318713
15	9	0	0.621777	-1.696355	1.001082

2e

Standard orientation:

Center Number	Atomic Number	Atomic Type	Coordinates (Angstroms) X	Y	Z
1	6	0	1.264852	1.112087	-0.388282
2	6	0	-0.017304	0.990047	0.458488
3	6	0	-0.604711	-0.382449	0.266286
4	6	0	0.417770	-1.448925	0.551115
5	6	0	1.676362	-1.185561	-0.290379
6	1	0	0.237022	1.122418	1.517055
7	1	0	0.985530	1.061037	-1.450583
8	1	0	1.740637	2.072899	-0.193772
9	1	0	0.003506	-2.434309	0.337019
10	1	0	2.467077	-1.886685	-0.023873
11	1	0	1.442213	-1.310096	-1.357368
12	8	0	2.185180	0.107799	-0.050746
13	16	0	-2.101493	-0.662909	-0.281142
14	1	0	0.699983	-1.407720	1.609734
15	9	0	-0.872815	2.001712	0.098075

3a

Standard orientation:

Center Number	Atomic Number	Atomic Type	Coordinates (Angstroms) X	Y	Z
1	6	0	1.297276	1.240241	0.013165
2	6	0	-0.079816	1.321989	0.683416
3	6	0	-1.031697	0.423680	-0.052146
4	6	0	-0.494644	-0.983714	-0.236583
5	6	0	0.920326	-0.926503	-0.802467
6	1	0	0.014826	0.978832	1.718232
7	1	0	1.244752	1.669251	-0.999364
8	1	0	2.040576	1.796387	0.584234
9	1	0	-1.143568	-1.575658	-0.890317
10	1	0	1.363721	-1.923029	-0.789374
11	1	0	0.853270	-0.581092	-1.847447
12	8	0	1.762994	-0.092489	-0.051579
13	1	0	-0.462282	2.343497	0.677921
14	9	0	-0.466364	-1.615049	1.006424
15	7	0	-2.122682	0.856933	-0.530569
16	1	0	-2.627865	0.114482	-1.017405

3e

Standard orientation:

Center Number	Atomic Number	Atomic Type	Coordinates (Angstroms) X	Y	Z
1	6	0	-0.314664	-1.414570	-0.273414
2	6	0	0.753590	-0.565884	0.428119
3	6	0	0.524089	0.896589	0.115128
4	6	0	-0.892580	1.323983	0.387227
5	6	0	-1.858198	0.349090	-0.293732
6	1	0	0.701411	-0.726967	1.509981
7	1	0	-0.167109	-1.336176	-1.360736
8	1	0	-0.220155	-2.460190	0.019710
9	1	0	-1.034377	2.342101	0.021385
10	1	0	-2.890488	0.557015	-0.012558
11	1	0	-1.770150	0.440088	-1.386177
12	8	0	-1.600168	-0.986643	0.098855
13	7	0	1.378425	1.682873	-0.386945
14	1	0	-1.081424	1.308655	1.466315
15	9	0	2.008224	-0.971661	0.000480
16	1	0	2.267224	1.198208	-0.524435

4a Standard orientation:

Center Number	Atomic Number	Atomic Type	Coordinates (Angstroms) X	Y	Z
1	6	0	1.145203	1.365349	0.032066
2	6	0	-0.236424	1.279912	0.699507
3	6	0	-1.073495	0.288839	-0.063379
4	6	0	-0.382975	-1.043023	-0.250255
5	6	0	1.016301	-0.823161	-0.811412
6	1	0	-0.107223	0.926445	1.726857
7	1	0	1.044316	1.802486	-0.973686
8	1	0	1.816042	1.995865	0.615748
9	1	0	-0.977175	-1.687151	-0.900951
10	1	0	1.571623	-1.761594	-0.801338
11	1	0	0.916112	-0.479367	-1.853339
12	8	0	1.758734	0.098800	-0.049435
13	7	0	-2.199896	0.471144	-0.614669
14	1	0	-0.714638	2.262788	0.722471
15	9	0	-0.277376	-1.664094	0.996621
16	1	0	-2.534930	1.421474	-0.446367

4e Standard orientation:

Center Number	Atomic Number	Atomic Type	Coordinates (Angstroms) X	Y	Z
1	6	0	-0.308699	-1.415608	-0.285549
2	6	0	0.771659	-0.575911	0.411828
3	6	0	0.546206	0.887700	0.109764
4	6	0	-0.876709	1.306775	0.403721
5	6	0	-1.852562	0.342596	-0.280909
6	1	0	0.703000	-0.731303	1.495289
7	1	0	-0.169742	-1.332171	-1.373387
8	1	0	-0.211278	-2.462294	0.001697
9	1	0	-1.047818	2.332258	0.065157
10	1	0	-2.881113	0.554707	0.011825
11	1	0	-1.773352	0.446636	-1.373258
12	8	0	-1.595456	-0.992664	0.096571
13	7	0	1.473946	1.594906	-0.379207
14	1	0	-1.051220	1.269992	1.484585
15	9	0	2.005607	-1.013629	-0.004655
16	1	0	1.147710	2.548484	-0.541257

5a Standard orientation:

Center Number	Atomic Number	Atomic Type	Coordinates (Angstroms) X	Y	Z
1	6	0	1.198756	1.350346	0.002080
2	6	0	-0.168087	1.300263	0.691647
3	6	0	-1.047825	0.315741	-0.026400
4	6	0	-0.392691	-1.021385	-0.235719
5	6	0	1.007547	-0.844596	-0.810681
6	1	0	-0.022568	0.974448	1.726565
7	1	0	1.090107	1.773863	-1.008963
8	1	0	1.897637	1.971081	0.563796
9	1	0	-0.989117	-1.662350	-0.891429
10	1	0	1.538419	-1.797351	-0.793528
11	1	0	0.904011	-0.512778	-1.856702
12	8	0	1.784441	0.066983	-0.073967
13	1	0	-0.622658	2.293982	0.705580
14	9	0	-0.290753	-1.678336	0.997972
15	1	0	-2.703087	1.567069	-0.401721
16	6	0	-2.251523	0.587162	-0.518487
17	1	0	-2.828567	-0.163989	-1.048244

5e

Standard orientation:

Center Number	Atomic Number	Atomic Type	Coordinates (Angstroms) X	Y	Z
1	6	0	-0.398653	-1.398464	-0.300207
2	6	0	0.705523	-0.623377	0.427556
3	6	0	-0.824443	1.342171	0.422874
4	6	0	-1.842258	0.449821	-0.291709
5	1	0	0.624406	-0.805418	1.505437
6	1	0	-0.233219	-1.306846	-1.383902
7	1	0	-0.367944	-2.453010	-0.024887
8	1	0	-0.935486	2.379522	0.097329
9	1	0	-2.863486	0.708398	-0.009344
10	1	0	-1.739072	0.567417	-1.380764
11	8	0	-1.667566	-0.908656	0.062495
12	1	0	2.522969	1.156320	-0.575213
13	6	0	1.548993	1.582004	-0.367115
14	1	0	1.396888	2.634058	-0.583476
15	1	0	-1.026521	1.303170	1.499626
16	9	0	1.934387	-1.121748	0.016968
17	6	0	0.569591	0.844741	0.142355

6a

Standard orientation:

Center Number	Atomic Number	Atomic Type	Coordinates (Angstroms) X	Y	Z
1	6	0	-0.893932	-1.508511	0.024636
2	6	0	0.475866	-1.341283	0.704682
3	6	0	1.310346	-0.365531	-0.088519
4	6	0	0.758657	1.060105	-0.168785
5	6	0	-0.596546	1.073375	-0.866245
6	1	0	0.331603	-0.959868	1.718885
7	1	0	1.002194	-2.296774	0.742649
8	1	0	-0.770778	-1.909752	-0.985282
9	1	0	-1.509709	-2.210311	0.588590
10	1	0	1.476072	1.684197	-0.706579
11	1	0	-0.991241	2.090606	-0.880519
12	1	0	-0.458113	0.747039	-1.901651
13	16	0	-1.844475	0.040800	-0.039778
14	9	0	0.636633	1.565641	1.120399
15	8	0	2.296942	-0.674705	-0.707230

6e

Standard orientation:

Center Number	Atomic Number	Atomic Type	Coordinates (Angstroms) X	Y	Z
1	6	0	-0.257811	-1.337692	-0.375856
2	6	0	0.891611	-0.684108	0.390043
3	6	0	0.988620	0.811827	0.086378
4	6	0	-0.255287	1.620052	0.409489
5	6	0	-1.491415	1.090314	-0.325470
6	1	0	0.737985	-0.813747	1.467598
7	1	0	-0.096010	-1.228253	-1.451511
8	1	0	-0.290653	-2.402230	-0.141003
9	1	0	-0.433240	1.574208	1.489186
10	1	0	-0.055676	2.657387	0.132637
11	1	0	-2.374732	1.667629	-0.048945
12	1	0	-1.362116	1.177871	-1.407626
13	16	0	-1.864107	-0.638613	0.095566
14	9	0	2.068090	-1.311247	0.047737
15	8	0	1.979130	1.297977	-0.388316

7a

Standard orientation:

Center Number	Atomic Number	Atomic Type	Coordinates (Angstroms) X	Y	Z
1	6	0	-0.913932	-1.602443	0.111186
2	6	0	0.268016	-1.107144	0.967448
3	6	0	1.026033	-0.058805	0.207309
4	6	0	0.243949	1.210751	-0.063845
5	6	0	-0.952492	0.918610	-0.969446
6	1	0	-0.119917	-0.677663	1.895819
7	1	0	0.929437	-1.941754	1.203258
8	1	0	-0.541114	-2.047529	-0.815468
9	1	0	-1.470180	-2.367943	0.654034
10	1	0	0.890976	1.957747	-0.527190
11	1	0	-1.515545	1.839786	-1.127082
12	1	0	-0.577935	0.573165	-1.937234
13	16	0	-2.111715	-0.289597	-0.265133
14	9	0	-0.201259	1.739092	1.144228
15	16	0	2.498350	-0.282494	-0.432373

7e

Standard orientation:

Center Number	Atomic Number	Atomic Type	Coordinates (Angstroms) X	Y	Z
1	6	0	0.892784	1.252047	-0.447399
2	6	0	-0.313033	1.006979	0.464091
3	6	0	-0.864678	-0.387434	0.266936
4	6	0	0.112875	-1.476380	0.636168
5	6	0	1.371482	-1.421896	-0.241310
6	1	0	-0.011950	1.122297	1.511915
7	1	0	0.587356	1.141197	-1.490987
8	1	0	1.251211	2.270871	-0.296263
9	1	0	0.408345	-1.355114	1.684569
10	1	0	-0.376822	-2.444622	0.522928
11	1	0	2.067243	-2.209235	0.051955
12	1	0	1.108379	-1.576500	-1.291022
13	16	0	2.274755	0.143670	-0.063933
14	9	0	-1.253571	1.977383	0.195428
15	16	0	-2.334018	-0.680247	-0.343746

8a

Standard orientation:

Center Number	Atomic Number	Atomic Type	Coordinates (Angstroms) X	Y	Z
1	6	0	0.939665	1.502881	-0.002970
2	6	0	-0.426673	1.360503	0.678126
3	6	0	-1.297592	0.384230	-0.064389
4	6	0	-0.758070	-1.033555	-0.166255
5	6	0	0.600956	-1.079131	-0.854242
6	1	0	-0.282398	1.009895	1.704236
7	1	0	-0.938886	2.324171	0.701398
8	1	0	0.820288	1.885289	-1.020816
9	1	0	1.567785	2.205019	0.547078
10	1	0	-1.466303	-1.661642	-0.718221
11	1	0	0.974864	-2.104312	-0.854850
12	1	0	0.471843	-0.761670	-1.893879
13	16	0	1.867871	-0.061310	-0.042465
14	9	0	-0.657156	-1.569359	1.114272
15	7	0	-2.374755	0.771526	-0.610220
16	1	0	-2.845170	-0.001808	-1.084035

8e

Standard orientation:

Center Number	Atomic Number	Atomic Type	Coordinates (Angstroms) X	Y	Z
1	6	0	-0.271693	-1.336394	-0.372029
2	6	0	0.861559	-0.680437	0.407941
3	6	0	0.972416	0.804202	0.105434
4	6	0	-0.257629	1.614596	0.429728
5	6	0	-1.490058	1.107111	-0.323326
6	1	0	0.711832	-0.826893	1.482443
7	1	0	-0.092983	-1.226046	-1.444899
8	1	0	-0.316694	-2.399944	-0.134623
9	1	0	-0.452442	1.564125	1.506539
10	1	0	-0.050751	2.653569	0.164932
11	1	0	-2.374327	1.683808	-0.047826
12	1	0	-1.347473	1.205191	-1.402850
13	16	0	-1.881945	-0.625796	0.072963
14	9	0	2.053403	-1.315235	0.067841
15	7	0	1.991460	1.354277	-0.403812
16	1	0	2.725549	0.661641	-0.561499

9a

Standard orientation:

Center Number	Atomic Number	Atomic Type	Coordinates (Angstroms) X	Y	Z
1	6	0	-0.868406	-1.532409	0.016397
2	6	0	0.493037	-1.320027	0.692989
3	6	0	1.313622	-0.312135	-0.072804
4	6	0	0.714202	1.075273	-0.180584
5	6	0	-0.646252	1.055680	-0.864721
6	1	0	0.335368	-0.956606	1.712361
7	1	0	1.032980	-2.270560	0.745130
8	1	0	-0.733857	-1.922242	-0.996978
9	1	0	-1.462441	-2.255654	0.577109
10	1	0	1.406428	1.713310	-0.733946
11	1	0	-1.068353	2.061762	-0.870187
12	1	0	-0.506638	0.737234	-1.902173
13	16	0	-1.864762	-0.012718	-0.039870
14	9	0	0.585273	1.606793	1.102213
15	7	0	2.404243	-0.523880	-0.683770
16	1	0	2.698327	-1.496028	-0.574589

9e

Standard orientation:

Center Number	Atomic Number	Atomic Type	Coordinates (Angstroms) X	Y	Z
1	6	0	-0.289763	-1.329795	-0.387661
2	6	0	0.865171	-0.710037	0.397185
3	6	0	1.004065	0.771447	0.102369
4	6	0	-0.216573	1.594371	0.462722
5	6	0	-1.462040	1.132461	-0.300371
6	1	0	0.690525	-0.844158	1.471217
7	1	0	-0.107017	-1.216207	-1.459414
8	1	0	-0.355639	-2.394039	-0.158583
9	1	0	-0.406358	1.509724	1.537925
10	1	0	-0.015595	2.647193	0.240382
11	1	0	-2.331104	1.725207	-0.010655
12	1	0	-1.317324	1.251258	-1.377770
13	16	0	-1.888745	-0.597139	0.061904
14	9	0	2.018727	-1.391535	0.074973
15	7	0	2.068478	1.204963	-0.426064
16	1	0	2.009378	2.213644	-0.571337

10a Standard orientation:

Center Number	Atomic Number	Atomic Type	Coordinates (Angstroms) X	Y	Z
1	6	0	0.910104	1.532448	-0.010495
2	6	0	-0.445394	1.341090	0.676788
3	6	0	-1.295690	0.327966	-0.042699
4	6	0	-0.709879	-1.054081	-0.173596
5	6	0	0.653107	-1.062039	-0.854447
6	1	0	-0.277257	1.007881	1.705613
7	1	0	-0.971074	2.299537	0.716293
8	1	0	0.772861	1.905319	-1.029940
9	1	0	1.518856	2.255972	0.534488
10	1	0	-1.391073	-1.698875	-0.737727
11	1	0	1.061109	-2.074076	-0.844580
12	1	0	0.519072	-0.755661	-1.896342
13	16	0	1.889095	0.000121	-0.050620
14	9	0	-0.589340	-1.621096	1.099439
15	6	0	-2.477037	0.611810	-0.581617
16	1	0	-3.062570	-0.143619	-1.095903
17	1	0	-2.902640	1.608282	-0.520538

10e Standard orientation:

Center Number	Atomic Number	Atomic Type	Coordinates (Angstroms) X	Y	Z
1	6	0	-0.328665	-1.329573	-0.384280
2	6	0	0.821519	-0.721708	0.410978
3	6	0	0.995263	0.748227	0.128705
4	6	0	-0.205661	1.595937	0.469228
5	6	0	-1.453060	1.163467	-0.305642
6	1	0	0.647355	-0.875396	1.481915
7	1	0	-0.131384	-1.221030	-1.453893
8	1	0	-0.421646	-2.391045	-0.150602
9	1	0	-0.416673	1.523729	1.542299
10	1	0	0.015631	2.643891	0.246972
11	1	0	-2.316222	1.767324	-0.019801
12	1	0	-1.295707	1.283084	-1.381164
13	16	0	-1.917743	-0.560694	0.042740
14	9	0	1.979614	-1.427139	0.087166
15	6	0	2.108870	1.240387	-0.401262
16	1	0	2.206140	2.302325	-0.600558
17	1	0	2.950277	0.602033	-0.639848

11a Standard orientation:

Center Number	Atomic Number	Atomic Type	Coordinates (Angstroms) X	Y	Z
1	6	0	1.101648	1.402436	0.022088
2	6	0	-0.284932	1.289231	0.711322
3	6	0	-1.105394	0.287834	-0.056167
4	6	0	-0.414157	-1.062052	-0.220196
5	6	0	0.948489	-0.837019	-0.883645
6	1	0	-0.145085	0.929094	1.735431
7	1	0	-0.804189	2.248429	0.733390
8	1	0	0.971063	1.874592	-0.958202
9	1	0	1.750856	2.055412	0.608025
10	1	0	-1.050805	-1.752470	-0.775434
11	1	0	1.477103	-1.790454	-0.950838
12	1	0	0.776417	-0.482145	-1.906978
13	7	0	1.769714	0.127262	-0.176262
14	1	0	2.070606	-0.259626	0.709911
15	9	0	-0.190602	-1.604576	1.052535
16	8	0	-2.149059	0.530617	-0.609337

11e

Standard orientation:

Center Number	Atomic Number	Atomic Type	Coordinates (Angstroms) X	Y	Z
1	6	0	-0.229578	-1.440294	-0.294178
2	6	0	0.810278	-0.542499	0.406426
3	6	0	0.520336	0.919236	0.092870
4	6	0	-0.909137	1.314981	0.394215
5	6	0	-1.879495	0.328152	-0.293075
6	1	0	0.745867	-0.684273	1.493482
7	1	0	-0.066395	-1.364393	-1.374778
8	1	0	-0.057561	-2.480256	-0.011193
9	1	0	-1.067153	1.271796	1.478617
10	1	0	-1.066130	2.342838	0.062323
11	1	0	-2.908766	0.552101	-0.006617
12	1	0	-1.813896	0.462793	-1.378238
13	1	0	-1.838367	-1.291421	0.961464
14	9	0	2.076159	-0.897234	0.006026
15	7	0	-1.597249	-1.065786	0.004155
16	8	0	1.336661	1.656121	-0.393242

12a

Standard orientation:

Center Number	Atomic Number	Atomic Type	Coordinates (Angstroms) X	Y	Z
1	6	0	1.101102	1.583822	-0.016005
2	6	0	-0.057015	1.124837	0.918907
3	6	0	-0.782526	0.016227	0.227357
4	6	0	0.125180	-1.133695	-0.129981
5	6	0	1.241736	-0.598752	-1.043918
6	1	0	0.377727	0.750879	1.851881
7	1	0	-0.732004	1.951539	1.141287
8	1	0	0.663267	2.054120	-0.903352
9	1	0	1.700234	2.340575	0.493772
10	1	0	-0.424337	-1.958327	-0.584165
11	1	0	1.933342	-1.413104	-1.270237
12	1	0	0.786186	-0.272840	-1.985548
13	7	0	1.966946	0.504351	-0.448409
14	1	0	2.532790	0.170305	0.321862
15	9	0	0.734939	-1.621069	1.035988
16	16	0	-2.311946	0.092586	-0.311793

12e

Standard orientation:

Center Number	Atomic Number	Atomic Type	Coordinates (Angstroms) X	Y	Z
1	6	0	1.220886	1.154219	-0.427360
2	6	0	-0.038708	1.000462	0.459452
3	6	0	-0.599635	-0.384349	0.283062
4	6	0	0.427015	-1.435930	0.599395
5	6	0	1.674722	-1.212337	-0.290735
6	1	0	0.236373	1.138865	1.513457
7	1	0	0.893371	1.110481	-1.471689
8	1	0	1.653896	2.141371	-0.256937
9	1	0	0.730657	-1.344583	1.650144
10	1	0	0.003737	-2.429132	0.446506
11	1	0	2.451537	-1.932074	-0.025946
12	1	0	1.399958	-1.398657	-1.334441
13	1	0	2.682457	0.271363	0.693876
14	9	0	-0.933604	1.992043	0.133495
15	7	0	2.214741	0.130043	-0.192818
16	16	0	-2.078651	-0.695545	-0.300598

13a Standard orientation:

Center Number	Atomic Number	Atomic Type	Coordinates (Angstroms) X	Y	Z
1	6	0	1.202916	1.349832	-0.004099
2	6	0	-0.179331	1.320416	0.683534
3	6	0	-1.072463	0.351721	-0.036880
4	6	0	-0.455643	-1.021491	-0.217023
5	6	0	0.923468	-0.891420	-0.867136
6	1	0	-0.054366	0.988063	1.719969
7	1	0	-0.646910	2.306213	0.687145
8	1	0	1.098241	1.806198	-0.995268
9	1	0	1.891420	1.976557	0.566198
10	1	0	-1.112774	-1.682532	-0.790551
11	1	0	1.393220	-1.876722	-0.912499
12	1	0	0.779562	-0.545824	-1.898320
13	7	0	1.801467	0.033774	-0.177073
14	1	0	2.070320	-0.353301	0.719635
15	9	0	-0.286161	-1.603699	1.048144
16	7	0	-2.188418	0.712845	-0.520683
17	1	0	-2.648296	-0.066042	-0.995685

13e Standard orientation:

Center Number	Atomic Number	Atomic Type	Coordinates (Angstroms) X	Y	Z
1	6	0	-0.285784	-1.432190	-0.300882
2	6	0	0.761767	-0.570317	0.422414
3	6	0	0.528075	0.893759	0.117450
4	6	0	-0.880124	1.334038	0.411579
5	6	0	-1.873313	0.397146	-0.303759
6	1	0	0.698490	-0.732308	1.505431
7	1	0	-0.102070	-1.351127	-1.377966
8	1	0	-0.155868	-2.479806	-0.022592
9	1	0	-1.060059	1.283877	1.492884
10	1	0	-0.999683	2.370918	0.092509
11	1	0	-2.900212	0.648877	-0.030989
12	1	0	-1.783068	0.543863	-1.385750
13	1	0	-1.908373	-1.239979	0.928361
14	9	0	2.035366	-0.956081	0.024394
15	7	0	-1.646326	-1.013704	-0.023581
16	7	0	1.378820	1.676640	-0.397986
17	1	0	2.261353	1.185245	-0.551278

14a Standard orientation:

Center Number	Atomic Number	Atomic Type	Coordinates (Angstroms) X	Y	Z
1	6	0	1.036970	1.466382	0.015598
2	6	0	-0.339989	1.270357	0.693963
3	6	0	-1.106854	0.207774	-0.047451
4	6	0	-0.334759	-1.078086	-0.228696
5	6	0	1.017405	-0.783198	-0.878283
6	1	0	-0.184725	0.939661	1.726437
7	1	0	-0.902298	2.208395	0.715921
8	1	0	0.885373	1.925458	-0.968470
9	1	0	1.645918	2.157799	0.601822
10	1	0	-0.926682	-1.797696	-0.795770
11	1	0	1.601203	-1.704796	-0.933212
12	1	0	0.833721	-0.446122	-1.905554
13	7	0	1.781075	0.232090	-0.174342
14	1	0	2.094588	-0.132436	0.717059
15	9	0	-0.088332	-1.629566	1.040614
16	7	0	-2.245439	0.307584	-0.597963
17	1	0	-2.638200	1.238746	-0.448411

14e Standard orientation:

Center Number	Atomic Number	Atomic Type	Coordinates (Angstroms) X	Y	Z
1	6	0	-0.273817	-1.430525	-0.321301
2	6	0	0.781509	-0.578185	0.406471
3	6	0	0.547162	0.885934	0.112074
4	6	0	-0.867515	1.312794	0.432809
5	6	0	-1.870266	0.385647	-0.285154
6	1	0	0.694364	-0.737360	1.490102
7	1	0	-0.097072	-1.334870	-1.398269
8	1	0	-0.135819	-2.480841	-0.058015
9	1	0	-1.030264	1.239847	1.515195
10	1	0	-1.019579	2.356732	0.143142
11	1	0	-2.892919	0.634373	0.006394
12	1	0	-1.794258	0.549622	-1.365930
13	1	0	-1.890029	-1.267160	0.921353
14	9	0	2.037428	-0.991664	0.026653
15	7	0	-1.636421	-1.025673	-0.029163
16	7	0	1.464074	1.592168	-0.400505
17	1	0	1.132708	2.545172	-0.555567

15a Standard orientation:

Center Number	Atomic Number	Atomic Type	Coordinates (Angstroms) X	Y	Z
1	6	0	1.120755	1.434899	-0.015603
2	6	0	-0.247642	1.301373	0.681497
3	6	0	-1.077072	0.259039	-0.016294
4	6	0	-0.364725	-1.048440	-0.220070
5	6	0	1.000353	-0.825674	-0.870839
6	1	0	-0.085092	0.994239	1.721167
7	1	0	-0.768820	2.262417	0.696293
8	1	0	0.974952	1.877953	-1.008145
9	1	0	1.767770	2.111667	0.547439
10	1	0	-0.970698	-1.751417	-0.797901
11	1	0	1.541636	-1.774067	-0.909211
12	1	0	0.831251	-0.498182	-1.903882
13	7	0	1.814561	0.165916	-0.190269
14	1	0	2.110411	-0.197234	0.708068
15	9	0	-0.141343	-1.644265	1.038558
16	6	0	-2.297492	0.463984	-0.501577
17	1	0	-2.843186	-0.323578	-1.011551
18	1	0	-2.793120	1.424085	-0.400112

15e Standard orientation:

Center Number	Atomic Number	Atomic Type	Coordinates (Angstroms) X	Y	Z
1	6	0	-0.369210	-1.417451	-0.329602
2	6	0	0.714002	-0.627621	0.419683
3	6	0	0.572426	0.842375	0.143075
4	6	0	-0.813772	1.349988	0.443267
5	6	0	-1.856746	0.495069	-0.299389
6	1	0	0.619875	-0.809703	1.498721
7	1	0	-0.170427	-1.323109	-1.402960
8	1	0	-0.299805	-2.476239	-0.070969
9	1	0	-1.007567	1.279224	1.521988
10	1	0	-0.903038	2.403044	0.162974
11	1	0	-2.869785	0.795463	-0.021094
12	1	0	-1.752415	0.664049	-1.377175
13	1	0	-1.989292	-1.164567	0.889888
14	9	0	1.963949	-1.105711	0.041521
15	7	0	-1.711590	-0.933590	-0.056730
16	6	0	1.547538	1.579699	-0.375992
17	1	0	1.395273	2.633410	-0.584396
18	1	0	2.517344	1.152608	-0.599808

16a

Standard orientation:

Center Number	Atomic Number	Atomic Type	X	Y	Z
1	6	0	-1.275230	-1.267197	0.039915
2	6	0	0.118924	-1.331265	0.688216
3	6	0	1.043914	-0.430196	-0.089520
4	6	0	0.527383	1.003486	-0.239587
5	6	0	-0.887327	0.973385	-0.803544
6	1	0	0.041739	-0.958872	1.713940
7	1	0	0.519298	-2.345380	0.690673
8	1	0	-1.219080	-1.721658	-0.965358
9	1	0	-1.978374	-1.857082	0.630913
10	1	0	1.205040	1.572947	-0.878065
11	1	0	-1.292264	1.987821	-0.802269
12	1	0	-0.803805	0.640751	-1.855284
13	7	0	-1.728357	0.113082	0.007673
14	9	0	0.523600	1.595942	1.020826
15	1	0	-2.695966	0.180182	-0.274091
16	8	0	2.055442	-0.792880	-0.634311

Coordinates (Angstroms)

16e

Standard orientation:

Center Number	Atomic Number	Atomic Type	X	Y	Z
1	6	0	-0.267536	-1.433704	-0.267863
2	6	0	0.798576	-0.563976	0.403009
3	6	0	0.546422	0.906672	0.083422
4	6	0	-0.877674	1.348394	0.355021
5	6	0	-1.873749	0.369868	-0.278926
6	1	0	0.741194	-0.697779	1.489497
7	1	0	-0.126524	-1.364914	-1.359798
8	1	0	-0.116141	-2.473006	0.028907
9	1	0	-1.035081	1.356573	1.439163
10	1	0	-1.003656	2.361736	-0.028195
11	1	0	-2.890077	0.629687	0.023576
12	1	0	-1.816720	0.465289	-1.376831
13	9	0	2.047698	-0.951018	-0.018710
14	7	0	-1.574285	-0.979936	0.175847
15	1	0	-2.294663	-1.629935	-0.106688
16	8	0	1.397019	1.625942	-0.367518

Coordinates (Angstroms)

17a

Standard orientation:

Center Number	Atomic Number	Atomic Type	X	Y	Z
1	6	0	-1.148048	-1.570334	0.021868
2	6	0	0.034982	-1.135833	0.908895
3	6	0	0.774900	-0.049594	0.189847
4	6	0	-0.106638	1.133356	-0.157491
5	6	0	-1.298766	0.638267	-0.974609
6	1	0	-0.369297	-0.735922	1.844315
7	1	0	0.696668	-1.974618	1.122494
8	1	0	-0.742899	-2.041542	-0.890883
9	1	0	-1.740659	-2.318371	0.552256
10	1	0	0.457001	1.891017	-0.702955
11	1	0	-1.983314	1.473839	-1.136451
12	1	0	-0.902064	0.322189	-1.956484
13	7	0	-1.985359	-0.418182	-0.257417
14	9	0	-0.560720	1.714799	1.024947
15	1	0	-2.842380	-0.680631	-0.722686
16	16	0	2.302023	-0.158566	-0.341454

Coordinates (Angstroms)

17e Standard orientation:

Center Number	Atomic Number	Atomic Type	X	Y	Z
1	6	0	1.235509	1.153840	-0.389227
2	6	0	-0.036325	0.997490	0.453753
3	6	0	-0.604435	-0.386410	0.256199
4	6	0	0.414108	-1.456969	0.551559
5	6	0	1.685280	-1.214656	-0.274395
6	1	0	0.217201	1.123515	1.513031
7	1	0	0.940484	1.105863	-1.450860
8	1	0	1.662692	2.139336	-0.195862
9	1	0	0.681784	-1.407109	1.613643
10	1	0	-0.012632	-2.437396	0.342335
11	1	0	2.447585	-1.942709	0.009774
12	1	0	1.448368	-1.369517	-1.340639
13	9	0	-0.922612	1.990947	0.110594
14	7	0	2.181307	0.124251	0.000270
15	1	0	3.080967	0.280143	-0.433010
16	16	0	-2.099807	-0.677511	-0.290186

Coordinates (Angstroms)

18a Standard orientation:

Center Number	Atomic Number	Atomic Type	X	Y	Z
1	6	0	1.315780	1.254644	0.016273
2	6	0	-0.067417	1.341513	0.673818
3	6	0	-1.024222	0.446196	-0.061397
4	6	0	-0.528821	-0.981102	-0.226590
5	6	0	0.882645	-0.984361	-0.799845
6	1	0	0.018802	0.997342	1.709123
7	1	0	-0.453062	2.361370	0.663856
8	1	0	1.256697	1.697985	-0.993970
9	1	0	2.035827	1.837566	0.594110
10	1	0	-1.203446	-1.553404	-0.871420
11	1	0	1.266594	-2.007025	-0.791788
12	1	0	0.795151	-0.659609	-1.854217
13	1	0	2.709966	-0.218374	-0.307926
14	7	0	1.749058	-0.133788	-0.007871
15	9	0	-0.532872	-1.600577	1.022317
16	7	0	-2.103291	0.895476	-0.553260
17	1	0	-2.618838	0.156192	-1.034259

Coordinates (Angstroms)

18e Standard orientation:

Center Number	Atomic Number	Atomic Type	X	Y	Z
1	6	0	-0.310157	-1.422772	-0.281298
2	6	0	0.753947	-0.582919	0.423331
3	6	0	0.546202	0.885044	0.107930
4	6	0	-0.855722	1.359142	0.387066
5	6	0	-1.868665	0.426360	-0.284712
6	1	0	0.690529	-0.740934	1.504657
7	1	0	-0.143562	-1.338053	-1.368743
8	1	0	-0.190806	-2.469878	0.003428
9	1	0	-1.033126	1.345729	1.468145
10	1	0	-0.955184	2.383813	0.027110
11	1	0	-2.883269	0.704263	0.007921
12	1	0	-1.788476	0.544558	-1.378932
13	1	0	-2.343901	-1.567944	-0.176667
14	9	0	2.014532	-0.993258	0.010883
15	7	0	-1.615191	-0.944327	0.142001
16	7	0	1.419190	1.650791	-0.394517
17	1	0	2.295391	1.143391	-0.531164

Coordinates (Angstroms)

19a Standard orientation:

Center Number	Atomic Number	Atomic Type	Coordinates (Angstroms) X	Y	Z
1	6	0	1.170799	1.373292	0.036495
2	6	0	-0.215254	1.299015	0.694471
3	6	0	-1.066610	0.319454	-0.070486
4	6	0	-0.426851	-1.042158	-0.240945
5	6	0	0.977283	-0.891220	-0.810267
6	1	0	-0.093565	0.939536	1.720567
7	1	0	-0.689852	2.283345	0.717776
8	1	0	1.062897	1.825021	-0.966544
9	1	0	1.823413	2.021247	0.625432
10	1	0	-1.052768	-1.663778	-0.882986
11	1	0	1.469006	-1.866379	-0.807164
12	1	0	0.860116	-0.569320	-1.861653
13	1	0	2.713638	0.065564	-0.306783
14	7	0	1.749017	0.041195	-0.007857
15	9	0	-0.358566	-1.657224	1.011174
16	7	0	-2.180252	0.534756	-0.636982
17	1	0	-2.483352	1.497834	-0.480949

19e Standard orientation:

Center Number	Atomic Number	Atomic Type	Coordinates (Angstroms) X	Y	Z
1	6	0	-0.307579	-1.423870	-0.292175
2	6	0	0.771943	-0.595315	0.407673
3	6	0	0.569522	0.873790	0.103782
4	6	0	-0.837821	1.341059	0.407601
5	6	0	-1.860752	0.425126	-0.273320
6	1	0	0.693144	-0.747459	1.490534
7	1	0	-0.147063	-1.336264	-1.380031
8	1	0	-0.187974	-2.471923	-0.011959
9	1	0	-1.002929	1.300225	1.489739
10	1	0	-0.963888	2.376229	0.080010
11	1	0	-2.871814	0.708297	0.027200
12	1	0	-1.783546	0.557082	-1.366622
13	1	0	-2.343587	-1.565156	-0.186825
14	9	0	2.010590	-1.038417	0.005190
15	7	0	-1.612123	-0.947971	0.138110
16	7	0	1.511537	1.559431	-0.389504
17	1	0	1.204567	2.519762	-0.550365

20a Standard orientation:

Center Number	Atomic Number	Atomic Type	Coordinates (Angstroms) X	Y	Z
1	6	0	1.217177	1.364535	0.006967
2	6	0	-0.157053	1.318498	0.683794
3	6	0	-1.043511	0.338552	-0.034791
4	6	0	-0.428187	-1.022320	-0.226457
5	6	0	0.974326	-0.904460	-0.809339
6	1	0	-0.019286	0.990039	1.718856
7	1	0	-0.613792	2.310940	0.694923
8	1	0	1.101958	1.802826	-1.001382
9	1	0	1.891470	2.007915	0.576918
10	1	0	-1.051517	-1.646569	-0.872637
11	1	0	1.447622	-1.888973	-0.799898
12	1	0	0.852050	-0.591942	-1.863382
13	1	0	2.732325	0.027252	-0.350619
14	7	0	1.774583	0.021582	-0.029438
15	9	0	-0.357983	-1.671084	1.013198
16	6	0	-2.237080	0.632474	-0.539142
17	1	0	-2.828045	-0.110202	-1.065731
18	1	0	-2.667045	1.623714	-0.435952

20e Standard orientation:

Center Number	Atomic Number	Atomic Type	Coordinates (Angstroms) X	Y	Z
1	6	0	-0.394638	-1.407833	-0.304884
2	6	0	0.706530	-0.638527	0.423366
3	6	0	0.590207	0.832766	0.134936
4	6	0	-0.787147	1.373082	0.424455
5	6	0	-1.848632	0.525329	-0.282786
6	1	0	0.616738	-0.816218	1.500784
7	1	0	-0.209374	-1.313741	-1.388445
8	1	0	-0.336740	-2.464261	-0.034787
9	1	0	-0.979769	1.332150	1.502774
10	1	0	-0.856343	2.416475	0.108328
11	1	0	-2.848523	0.853893	0.010246
12	1	0	-1.748419	0.668501	-1.372795
13	1	0	-2.435856	-1.440038	-0.244643
14	9	0	1.940326	-1.141614	0.025999
15	7	0	-1.680683	-0.868389	0.108068
16	6	0	1.583641	1.549611	-0.377007
17	1	0	1.451293	2.604338	-0.594220
18	1	0	2.549085	1.105586	-0.586183

21a Standard orientation:

Center Number	Atomic Number	Atomic Type	Coordinates (Angstroms) X	Y	Z
1	6	0	1.159089	1.399298	0.033402
2	6	0	-0.232334	1.319496	0.693011
3	6	0	-1.087235	0.349906	-0.083923
4	6	0	-0.481958	-1.048789	-0.219034
5	6	0	0.891348	-0.952939	-0.866806
6	1	0	-0.125081	0.945788	1.716972
7	1	0	-0.728808	2.289887	0.720319
8	1	0	1.056961	1.857800	-0.957259
9	1	0	1.801058	2.058723	0.622498
10	1	0	-1.168555	-1.695312	-0.767142
11	1	0	1.331003	-1.952531	-0.918228
12	1	0	0.748533	-0.607653	-1.897950
13	1	0	2.760822	0.098025	-0.617720
14	9	0	-0.339320	-1.580788	1.070548
15	6	0	1.799650	0.016189	-0.103819
16	1	0	2.003754	-0.393633	0.890331
17	8	0	-2.114646	0.640879	-0.642967

21e Standard orientation:

Center Number	Atomic Number	Atomic Type	Coordinates (Angstroms) X	Y	Z
1	6	0	-0.216019	-1.455517	-0.299239
2	6	0	0.810153	-0.563296	0.394038
3	6	0	0.546519	0.905940	0.074935
4	6	0	-0.867137	1.349506	0.395423
5	6	0	-1.894895	0.429450	-0.282441
6	1	0	0.737170	-0.687864	1.483243
7	1	0	-0.065229	-1.371433	-1.381128
8	1	0	-0.024548	-2.494947	-0.022686
9	1	0	-1.001633	1.300267	1.483614
10	1	0	-0.978187	2.390008	0.088104
11	1	0	-2.904224	0.726838	0.012935
12	1	0	-1.830392	0.561685	-1.368366
13	1	0	-2.355613	-1.678990	-0.454455
14	9	0	2.084357	-0.924410	0.019808
15	1	0	-1.806239	-1.191654	1.140798
16	6	0	-1.641396	-1.037171	0.067218
17	8	0	1.380792	1.624038	-0.407491

22a Standard orientation:

Center Number	Atomic Number	Atomic Type	Coordinates (Angstroms) X	Y	Z
1	6	0	1.118116	1.612251	0.012306
2	6	0	-0.056697	1.146345	0.899118
3	6	0	-0.787090	0.044978	0.191004
4	6	0	0.092687	-1.144708	-0.134728
5	6	0	1.252695	-0.696119	-1.017026
6	1	0	0.343698	0.751067	1.839972
7	1	0	-0.737451	1.966626	1.124684
8	1	0	0.709817	2.075278	-0.893375
9	1	0	1.680668	2.385140	0.542013
10	1	0	-0.489584	-1.946542	-0.588516
11	1	0	1.897002	-1.557466	-1.211449
12	1	0	0.834277	-0.375620	-1.977821
13	1	0	2.814270	0.792406	-1.050024
14	9	0	0.616567	-1.646372	1.067378
15	6	0	2.034193	0.448139	-0.366003
16	1	0	2.537843	0.073011	0.530134
17	16	0	-2.316442	0.136758	-0.339379

22e Standard orientation:

Center Number	Atomic Number	Atomic Type	Coordinates (Angstroms) X	Y	Z
1	6	0	1.187567	1.207948	-0.421478
2	6	0	-0.056470	1.006585	0.445076
3	6	0	-0.602712	-0.388105	0.256888
4	6	0	0.419908	-1.442424	0.594465
5	6	0	1.681708	-1.260320	-0.267238
6	1	0	0.204964	1.125579	1.505771
7	1	0	0.877517	1.148412	-1.470293
8	1	0	1.571038	2.215888	-0.247303
9	1	0	0.694619	-1.334657	1.652664
10	1	0	-0.017892	-2.430960	0.459541
11	1	0	2.425792	-2.007810	0.019023
12	1	0	1.423968	-1.447976	-1.315373
13	1	0	3.101766	0.288061	-0.790565
14	9	0	-0.981733	1.982743	0.148118
15	1	0	2.626707	0.288403	0.900287
16	6	0	2.249639	0.150026	-0.120431
17	16	0	-2.084420	-0.708243	-0.310657

23a Standard orientation:

Center Number	Atomic Number	Atomic Type	Coordinates (Angstroms) X	Y	Z
1	6	0	1.228636	1.368710	0.006535
2	6	0	-0.156510	1.338026	0.673059
3	6	0	-1.062086	0.387121	-0.059062
4	6	0	-0.500972	-1.016304	-0.211641
5	6	0	0.875991	-0.980995	-0.857428
6	1	0	-0.049482	0.990919	1.707150
7	1	0	-0.621548	2.324209	0.688742
8	1	0	1.133848	1.814493	-0.990897
9	1	0	1.897053	2.015004	0.581327
10	1	0	-1.194418	-1.651391	-0.771307
11	1	0	1.277366	-1.997433	-0.893295
12	1	0	0.745667	-0.644828	-1.893274
13	1	0	2.784019	0.001002	-0.631518
14	9	0	-0.384507	-1.581044	1.068003
15	6	0	1.822459	-0.035825	-0.112850
16	1	0	2.015113	-0.438857	0.886621
17	7	0	-2.161719	0.777566	-0.556724
18	1	0	-2.640128	0.008924	-1.030177

23e Standard orientation:

Center Number	Atomic Number	Atomic Type	Coordinates (Angstroms) X	Y	Z
1	6	0	-0.267170	-1.447843	-0.305695
2	6	0	0.763610	-0.586159	0.411500
3	6	0	0.550867	0.882441	0.099189
4	6	0	-0.840955	1.364362	0.414247
5	6	0	-1.887982	0.490594	-0.291822
6	1	0	0.691637	-0.732598	1.496452
7	1	0	-0.096168	-1.355626	-1.383841
8	1	0	-0.112304	-2.494999	-0.034593
9	1	0	-0.999011	1.306276	1.499242
10	1	0	-0.918823	2.411696	0.120600
11	1	0	-2.893072	0.812704	-0.007125
12	1	0	-1.800321	0.636469	-1.374446
13	1	0	-2.409622	-1.603735	-0.502210
14	9	0	2.046032	-0.976279	0.039409
15	1	0	-1.874283	-1.152658	1.108573
16	6	0	-1.686682	-0.988950	0.039914
17	7	0	1.417043	1.647842	-0.415647
18	1	0	2.288254	1.137405	-0.571795

24a Standard orientation:

Center Number	Atomic Number	Atomic Type	Coordinates (Angstroms) X	Y	Z
1	6	0	1.073382	1.477836	0.027250
2	6	0	-0.304708	1.291676	0.686335
3	6	0	-1.097426	0.256097	-0.069806
4	6	0	-0.392892	-1.074485	-0.225641
5	6	0	0.973638	-0.887599	-0.867390
6	1	0	-0.166722	0.939799	1.714721
7	1	0	-0.856204	2.234917	0.725468
8	1	0	0.936873	1.925530	-0.964507
9	1	0	1.668116	2.181761	0.615325
10	1	0	-1.028806	-1.762671	-0.783258
11	1	0	1.481714	-1.854490	-0.911360
12	1	0	0.809814	-0.558179	-1.900361
13	1	0	2.765002	0.295437	-0.623486
14	9	0	-0.210788	-1.620436	1.057648
15	1	0	-2.575835	1.352341	-0.492387
16	6	0	1.812123	0.144837	-0.109331
17	1	0	2.046294	-0.245999	0.886096
18	7	0	-2.223979	0.403614	-0.633368

24e Standard orientation:

Center Number	Atomic Number	Atomic Type	Coordinates (Angstroms) X	Y	Z
1	6	0	-0.256873	-1.446308	-0.326404
2	6	0	0.783821	-0.595413	0.396246
3	6	0	0.569858	0.873116	0.095142
4	6	0	-0.827899	1.341489	0.440035
5	6	0	-1.883212	0.482744	-0.272838
6	1	0	0.688376	-0.739467	1.481559
7	1	0	-0.088818	-1.341632	-1.403908
8	1	0	-0.096013	-2.495840	-0.068871
9	1	0	-0.970414	1.254761	1.524829
10	1	0	-0.936163	2.398146	0.181644
11	1	0	-2.884718	0.801179	0.028546
12	1	0	-1.806014	0.646903	-1.353649
13	1	0	-2.401233	-1.606210	-0.517668
14	9	0	2.048456	-1.012270	0.042575
15	1	0	1.182837	2.520659	-0.574579
16	1	0	-1.859496	-1.182317	1.098425
17	6	0	-1.676000	-1.001077	0.031969
18	7	0	1.496770	1.560992	-0.423486

25a

Standard orientation:

Center Number	Atomic Number	Atomic Type	Coordinates (Angstroms) X	Y	Z
1	6	0	1.143241	1.456584	-0.004704
2	6	0	-0.227724	1.317191	0.674024
3	6	0	-1.071208	0.292718	-0.036177
4	6	0	-0.409782	-1.047648	-0.215738
5	6	0	0.961070	-0.914071	-0.863450
6	1	0	-0.078241	0.991973	1.710446
7	1	0	-0.747643	2.277800	0.705249
8	1	0	1.005856	1.889723	-1.003093
9	1	0	1.768349	2.151785	0.562809
10	1	0	-1.051687	-1.728161	-0.780767
11	1	0	1.437338	-1.897846	-0.895068
12	1	0	0.804721	-0.592598	-1.900045
13	1	0	2.793818	0.208095	-0.648649
14	9	0	-0.236997	-1.633406	1.056177
15	6	0	-2.280012	0.531892	-0.534102
16	1	0	-2.845319	-0.241066	-1.045257
17	1	0	-2.747538	1.507144	-0.443198
18	6	0	1.839081	0.099752	-0.126741
19	1	0	2.065328	-0.284710	0.873317

25e

Standard orientation:

Center Number	Atomic Number	Atomic Type	Coordinates (Angstroms) X	Y	Z
1	6	0	-0.352464	-1.435077	-0.332258
2	6	0	0.716550	-0.641845	0.409415
3	6	0	0.593903	0.830702	0.125387
4	6	0	-0.774312	1.377059	0.446475
5	6	0	-1.866669	0.586619	-0.287582
6	1	0	0.616751	-0.808640	1.490488
7	1	0	-0.164121	-1.331716	-1.406504
8	1	0	-0.256585	-2.493747	-0.077721
9	1	0	-0.949465	1.296827	1.528097
10	1	0	-0.821318	2.438595	0.192670
11	1	0	-2.854536	0.956185	0.002120
12	1	0	-1.762704	0.753338	-1.365849
13	1	0	-2.500165	-1.471028	-0.553845
14	9	0	1.974075	-1.124362	0.054676
15	6	0	1.581174	1.550354	-0.394584
16	1	0	1.447173	2.606996	-0.601398
17	1	0	2.542688	1.106434	-0.621252
18	1	0	-1.956767	-1.086164	1.071282
19	6	0	-1.749452	-0.909116	0.008118

Implicit DMSO

1a Standard orientation:

Center Number	Atomic Number	Atomic Type	Coordinates (Angstroms) X	Y	Z
1	6	0	1.273204	1.252383	0.029502
2	6	0	-0.132772	1.337748	0.649843
3	6	0	-1.051123	0.408302	-0.092514
4	6	0	-0.482678	-1.001425	-0.263207
5	6	0	0.940841	-0.921592	-0.794757
6	1	0	-0.074776	1.011569	1.693000
7	1	0	-0.520153	2.356241	0.614024
8	1	0	1.261450	1.667442	-0.987367
9	1	0	1.981202	1.824420	0.627282
10	1	0	-1.123352	-1.601391	-0.910283
11	1	0	1.394789	-1.912706	-0.783425
12	1	0	0.902471	-0.563602	-1.833996
13	8	0	1.745664	-0.081092	0.001045
14	9	0	-0.466022	-1.598332	1.005432
15	8	0	-2.109697	0.724907	-0.581210

1e Standard orientation:

Center Number	Atomic Number	Atomic Type	Coordinates (Angstroms) X	Y	Z
1	6	0	-0.281125	-1.430960	-0.252582
2	6	0	0.788318	-0.546984	0.409468
3	6	0	0.523027	0.914446	0.090677
4	6	0	-0.906024	1.320064	0.343593
5	6	0	-1.861724	0.300072	-0.291187
6	1	0	0.756271	-0.681637	1.495421
7	1	0	-0.166216	-1.378769	-1.343875
8	1	0	-0.153647	-2.464618	0.066447
9	1	0	-1.075506	2.321713	-0.053153
10	1	0	-2.888848	0.505249	0.006319
11	1	0	-1.798794	0.353630	-1.385935
12	8	0	-1.565677	-1.015674	0.143761
13	8	0	1.375689	1.645841	-0.352068
14	1	0	-1.074246	1.336100	1.425541
15	9	0	2.038451	-0.930315	-0.038236

2a Standard orientation:

Center Number	Atomic Number	Atomic Type	Coordinates (Angstroms) X	Y	Z
1	6	0	1.174916	1.539079	-0.002416
2	6	0	-0.026754	1.145042	0.880812
3	6	0	-0.771953	0.052980	0.184090
4	6	0	0.120766	-1.113928	-0.176310
5	6	0	1.315815	-0.594433	-0.973705
6	1	0	0.359179	0.769722	1.834080
7	1	0	0.814883	1.985226	-0.939310
8	1	0	1.795840	2.266674	0.518185
9	1	0	-0.416263	-1.884325	-0.729033
10	1	0	2.024777	-1.406174	-1.137468
11	1	0	0.952184	-0.236342	-1.947215
12	8	0	1.996602	0.423174	-0.278233
13	1	0	-0.665512	2.007783	1.066391
14	16	0	-2.314637	0.135529	-0.313933
15	9	0	0.591061	-1.692096	1.012036

2e

Standard orientation:

Center Number	Atomic Number	Atomic Type	Coordinates (Angstroms) X	Y	Z
1	6	0	1.268123	1.117643	-0.384645
2	6	0	-0.015777	0.984830	0.459472
3	6	0	-0.599203	-0.385288	0.264158
4	6	0	0.419074	-1.451572	0.542194
5	6	0	1.681457	-1.183685	-0.293808
6	1	0	0.227502	1.120819	1.518348
7	1	0	0.997857	1.067105	-1.448338
8	1	0	1.736215	2.079688	-0.180262
9	1	0	0.013095	-2.437985	0.320454
10	1	0	2.466151	-1.887936	-0.021851
11	1	0	1.455987	-1.300648	-1.361813
12	8	0	2.188186	0.112873	-0.043301
13	16	0	-2.105148	-0.662215	-0.277454
14	1	0	0.694849	-1.414512	1.602026
15	9	0	-0.881869	1.997151	0.092543

3a

Standard orientation:

Center Number	Atomic Number	Atomic Type	Coordinates (Angstroms) X	Y	Z
1	6	0	1.310416	1.234599	0.006478
2	6	0	-0.073202	1.337130	0.659962
3	6	0	-1.029922	0.430112	-0.058338
4	6	0	-0.496671	-0.978734	-0.244691
5	6	0	0.918469	-0.936097	-0.802105
6	1	0	0.009361	1.020589	1.704490
7	1	0	1.277058	1.646129	-1.012051
8	1	0	2.047974	1.793256	0.581498
9	1	0	-1.146851	-1.576502	-0.887277
10	1	0	1.350684	-1.937015	-0.793000
11	1	0	0.863805	-0.584469	-1.842982
12	8	0	1.762735	-0.108530	-0.034642
13	1	0	-0.439574	2.364107	0.633010
14	9	0	-0.470968	-1.599288	1.016303
15	7	0	-2.137338	0.845705	-0.518211
16	1	0	-2.638792	0.093735	-0.993643

3e

Standard orientation:

Center Number	Atomic Number	Atomic Type	Coordinates (Angstroms) X	Y	Z
1	6	0	-0.320510	-1.416804	-0.270015
2	6	0	0.748221	-0.565665	0.428294
3	6	0	0.525327	0.895688	0.112929
4	6	0	-0.888447	1.328487	0.380775
5	6	0	-1.859511	0.356395	-0.296850
6	1	0	0.702791	-0.721812	1.509883
7	1	0	-0.179165	-1.344459	-1.357485
8	1	0	-0.224849	-2.459376	0.031675
9	1	0	-1.037752	2.344644	0.012987
10	1	0	-2.888012	0.575634	-0.013023
11	1	0	-1.772934	0.437970	-1.388806
12	8	0	-1.606385	-0.981890	0.103787
13	7	0	1.395329	1.674554	-0.380996
14	1	0	-1.073672	1.316303	1.459982
15	9	0	2.005286	-0.975902	-0.004437
16	1	0	2.279320	1.178852	-0.509392

4a Standard orientation:

Center Number	Atomic Number	Atomic Type	Coordinates (Angstroms) X	Y	Z
1	6	0	1.163852	1.361673	0.026692
2	6	0	-0.232030	1.305233	0.662118
3	6	0	-1.070037	0.297236	-0.075389
4	6	0	-0.381654	-1.036604	-0.265260
5	6	0	1.028158	-0.835311	-0.798726
6	1	0	-0.130098	0.990444	1.705281
7	1	0	1.097400	1.774846	-0.989435
8	1	0	1.825236	1.995462	0.616279
9	1	0	-0.964956	-1.683985	-0.920789
10	1	0	1.568919	-1.781893	-0.784259
11	1	0	0.954362	-0.487029	-1.839418
12	8	0	1.763331	0.078505	-0.014181
13	7	0	-2.217394	0.468278	-0.589694
14	1	0	-0.700996	2.291164	0.642432
15	9	0	-0.304056	-1.664190	0.992498
16	1	0	-2.547983	1.419362	-0.417868

4e Standard orientation:

Center Number	Atomic Number	Atomic Type	Coordinates (Angstroms) X	Y	Z
1	6	0	-0.312611	-1.420528	-0.282636
2	6	0	0.764841	-0.576715	0.413541
3	6	0	0.545358	0.885429	0.109807
4	6	0	-0.873415	1.310900	0.395892
5	6	0	-1.852603	0.347748	-0.283277
6	1	0	0.708865	-0.732875	1.495643
7	1	0	-0.179697	-1.340180	-1.370768
8	1	0	-0.212043	-2.465129	0.010343
9	1	0	-1.041424	2.332835	0.049142
10	1	0	-2.877464	0.567531	0.013220
11	1	0	-1.776122	0.440975	-1.374979
12	8	0	-1.597817	-0.992774	0.102310
13	7	0	1.478436	1.595993	-0.371342
14	1	0	-1.044949	1.282903	1.476960
15	9	0	2.008773	-1.016076	-0.011787
16	1	0	1.147932	2.547862	-0.532533

5a Standard orientation:

Center Number	Atomic Number	Atomic Type	Coordinates (Angstroms) X	Y	Z
1	6	0	1.206622	1.350674	-0.001634
2	6	0	-0.166135	1.316143	0.672915
3	6	0	-1.046970	0.319633	-0.030245
4	6	0	-0.393917	-1.016381	-0.246464
5	6	0	1.009162	-0.850684	-0.809484
6	1	0	-0.032017	1.020931	1.718969
7	1	0	1.115209	1.756689	-1.019433
8	1	0	1.901639	1.974633	0.560358
9	1	0	-0.988855	-1.661463	-0.896094
10	1	0	1.532626	-1.807381	-0.798449
11	1	0	0.917407	-0.510258	-1.851496
12	8	0	1.785664	0.055373	-0.055517
13	1	0	-0.615987	2.311272	0.658713
14	9	0	-0.294742	-1.679506	1.001213
15	1	0	-2.713431	1.560414	-0.388065
16	6	0	-2.260604	0.581088	-0.505677
17	1	0	-2.838176	-0.175102	-1.027744

5e

Standard orientation:

Center Number	Atomic Number	Atomic Type	Coordinates (Angstroms) X	Y	Z
1	6	0	-0.397572	-1.401380	-0.299657
2	6	0	0.702485	-0.621907	0.429123
3	6	0	-0.824742	1.341926	0.421922
4	6	0	-1.843520	0.452829	-0.292470
5	1	0	0.627948	-0.804448	1.506180
6	1	0	-0.236393	-1.309534	-1.383336
7	1	0	-0.363003	-2.455075	-0.022088
8	1	0	-0.937548	2.377971	0.095260
9	1	0	-2.862673	0.714658	-0.008127
10	1	0	-1.741906	0.565211	-1.380866
11	8	0	-1.669252	-0.911789	0.063236
12	1	0	2.527032	1.167412	-0.571991
13	6	0	1.549316	1.586154	-0.365134
14	1	0	1.392890	2.637708	-0.580873
15	1	0	-1.024303	1.303396	1.498805
16	9	0	1.937558	-1.124356	0.014638
17	6	0	0.569691	0.845080	0.141117

6a

Standard orientation:

Center Number	Atomic Number	Atomic Type	Coordinates (Angstroms) X	Y	Z
1	6	0	-0.908510	-1.511066	0.004458
2	6	0	0.473175	-1.363298	0.663007
3	6	0	1.311114	-0.366307	-0.094020
4	6	0	0.749688	1.055723	-0.183911
5	6	0	-0.610696	1.076908	-0.864470
6	1	0	0.347757	-1.016921	1.692628
7	1	0	0.991772	-2.323212	0.669972
8	1	0	-0.808874	-1.890425	-1.015234
9	1	0	-1.514191	-2.219579	0.569340
10	1	0	1.458916	1.686907	-0.721817
11	1	0	-0.997620	2.096456	-0.879374
12	1	0	-0.487468	0.745168	-1.898977
13	16	0	-1.847727	0.046539	-0.018841
14	9	0	0.642193	1.558291	1.116906
15	8	0	2.338123	-0.649925	-0.664702

6e

Standard orientation:

Center Number	Atomic Number	Atomic Type	Coordinates (Angstroms) X	Y	Z
1	6	0	-0.267663	-1.343895	-0.372579
2	6	0	0.881356	-0.684911	0.386287
3	6	0	0.986777	0.808199	0.086414
4	6	0	-0.249750	1.624845	0.387260
5	6	0	-1.493281	1.092952	-0.333858
6	1	0	0.743938	-0.815343	1.464285
7	1	0	-0.115779	-1.239086	-1.449464
8	1	0	-0.303219	-2.405186	-0.126159
9	1	0	-0.420580	1.599380	1.468552
10	1	0	-0.052075	2.657328	0.093295
11	1	0	-2.368524	1.677170	-0.049757
12	1	0	-1.374894	1.168563	-1.417081
13	16	0	-1.866198	-0.631684	0.105293
14	9	0	2.067236	-1.312790	0.033673
15	8	0	2.000068	1.287010	-0.361568

7a Standard orientation:

Center Number	Atomic Number	Atomic Type	Coordinates (Angstroms) X	Y	Z
1	6	0	-0.933747	-1.603996	0.090022
2	6	0	0.267051	-1.133772	0.933063
3	6	0	1.024228	-0.072205	0.193171
4	6	0	0.246449	1.201845	-0.072774
5	6	0	-0.961743	0.928665	-0.964466
6	1	0	-0.099691	-0.725614	1.879603
7	1	0	0.920430	-1.980973	1.142246
8	1	0	-0.585680	-2.033229	-0.852480
9	1	0	-1.484615	-2.372666	0.632240
10	1	0	0.888451	1.952728	-0.533960
11	1	0	-1.512337	1.857248	-1.118546
12	1	0	-0.603365	0.576604	-1.934954
13	16	0	-2.122150	-0.270899	-0.245958
14	9	0	-0.181882	1.726264	1.152744
15	16	0	2.513419	-0.274956	-0.420476

7e Standard orientation:

Center Number	Atomic Number	Atomic Type	Coordinates (Angstroms) X	Y	Z
1	6	0	0.900688	1.256651	-0.442595
2	6	0	-0.308298	1.001572	0.460652
3	6	0	-0.860562	-0.389521	0.260741
4	6	0	0.112651	-1.482035	0.617542
5	6	0	1.377298	-1.421241	-0.251449
6	1	0	-0.023505	1.119330	1.511070
7	1	0	0.607511	1.147834	-1.489401
8	1	0	1.257010	2.273794	-0.279338
9	1	0	0.397339	-1.371259	1.669577
10	1	0	-0.371320	-2.450922	0.491627
11	1	0	2.067077	-2.211581	0.044443
12	1	0	1.124552	-1.564629	-1.304374
13	16	0	2.277180	0.143789	-0.053260
14	9	0	-1.257415	1.975241	0.183535
15	16	0	-2.344217	-0.675807	-0.332038

8a Standard orientation:

Center Number	Atomic Number	Atomic Type	Coordinates (Angstroms) X	Y	Z
1	6	0	0.947353	1.504338	-0.013205
2	6	0	-0.424806	1.372917	0.657961
3	6	0	-1.297806	0.385890	-0.067930
4	6	0	-0.755368	-1.030847	-0.173068
5	6	0	0.602453	-1.083384	-0.856614
6	1	0	-0.289442	1.044762	1.692796
7	1	0	-0.927292	2.341856	0.665656
8	1	0	0.840257	1.872579	-1.036650
9	1	0	1.571414	2.209134	0.536629
10	1	0	-1.461322	-1.667299	-0.712933
11	1	0	0.971501	-2.109628	-0.862900
12	1	0	0.477641	-0.759338	-1.893350
13	16	0	1.865993	-0.066800	-0.034773
14	9	0	-0.651758	-1.555402	1.123632
15	7	0	-2.392453	0.753111	-0.595661
16	1	0	-2.856608	-0.029909	-1.058803

8e Standard orientation:

Center Number	Atomic Number	Atomic Type	Coordinates (Angstroms) X	Y	Z
1	6	0	-0.270725	-1.340200	-0.370553
2	6	0	0.858971	-0.676491	0.407264
3	6	0	0.969183	0.806649	0.103113
4	6	0	-0.261076	1.615112	0.424257
5	6	0	-1.495479	1.106235	-0.325071
6	1	0	0.716675	-0.819434	1.482129
7	1	0	-0.097624	-1.231977	-1.443968
8	1	0	-0.314132	-2.401449	-0.125018
9	1	0	-0.450396	1.566430	1.501615
10	1	0	-0.065930	2.655912	0.158288
11	1	0	-2.376709	1.683035	-0.043184
12	1	0	-1.358855	1.200761	-1.404934
13	16	0	-1.880411	-0.627582	0.074755
14	9	0	2.057365	-1.310351	0.065442
15	7	0	1.997795	1.351465	-0.399004
16	1	0	2.727461	0.653100	-0.551018

9a Standard orientation:

Center Number	Atomic Number	Atomic Type	Coordinates (Angstroms) X	Y	Z
1	6	0	-0.878862	-1.536025	0.000996
2	6	0	0.492705	-1.339954	0.658444
3	6	0	1.313299	-0.312934	-0.079877
4	6	0	0.706601	1.071589	-0.197092
5	6	0	-0.659740	1.058256	-0.863231
6	1	0	0.352865	-1.010159	1.692060
7	1	0	1.028845	-2.292330	0.676094
8	1	0	-0.765347	-1.905049	-1.021351
9	1	0	-1.464454	-2.265529	0.560907
10	1	0	1.388355	1.716615	-0.753690
11	1	0	-1.075749	2.066286	-0.872837
12	1	0	-0.534386	0.731168	-1.898912
13	16	0	-1.866805	-0.008135	-0.018606
14	9	0	0.593892	1.609791	1.095757
15	7	0	2.431743	-0.509228	-0.647241
16	1	0	2.727493	-1.479945	-0.531140

9e Standard orientation:

Center Number	Atomic Number	Atomic Type	Coordinates (Angstroms) X	Y	Z
1	6	0	-0.293797	-1.336015	-0.385286
2	6	0	0.857382	-0.709296	0.396373
3	6	0	1.002064	0.769825	0.100726
4	6	0	-0.214410	1.596926	0.452467
5	6	0	-1.463452	1.134520	-0.303420
6	1	0	0.696284	-0.845646	1.470391
7	1	0	-0.118453	-1.224440	-1.458130
8	1	0	-0.360930	-2.398027	-0.148169
9	1	0	-0.397238	1.520815	1.529068
10	1	0	-0.013965	2.646280	0.220229
11	1	0	-2.327850	1.728973	-0.006541
12	1	0	-1.326730	1.248148	-1.381341
13	16	0	-1.888619	-0.595193	0.065936
14	9	0	2.022676	-1.394435	0.067655
15	7	0	2.074943	1.206600	-0.413938
16	1	0	2.011383	2.214961	-0.556973

10a

Standard orientation:

Center Number	Atomic Number	Atomic Type	Coordinates (Angstroms) X	Y	Z
1	6	0	0.916726	1.535926	-0.017555
2	6	0	-0.443677	1.354719	0.660423
3	6	0	-1.295065	0.329930	-0.043927
4	6	0	-0.708514	-1.049750	-0.184409
5	6	0	0.655168	-1.064860	-0.857478
6	1	0	-0.283678	1.048935	1.699421
7	1	0	-0.965913	2.314853	0.676071
8	1	0	0.790506	1.893373	-1.042934
9	1	0	1.523792	2.262216	0.524314
10	1	0	-1.387719	-1.698254	-0.742750
11	1	0	1.059457	-2.077890	-0.857407
12	1	0	0.526547	-0.747736	-1.895708
13	16	0	1.887370	-0.004739	-0.040746
14	9	0	-0.588147	-1.623875	1.101728
15	6	0	-2.487694	0.603194	-0.565414
16	1	0	-3.072716	-0.158011	-1.071763
17	1	0	-2.916531	1.598258	-0.502703

10e

Standard orientation:

Center Number	Atomic Number	Atomic Type	Coordinates (Angstroms) X	Y	Z
1	6	0	-0.325146	-1.334745	-0.383873
2	6	0	0.819174	-0.718777	0.412253
3	6	0	0.992947	0.749923	0.127425
4	6	0	-0.209385	1.595017	0.471002
5	6	0	-1.457149	1.164296	-0.303101
6	1	0	0.650899	-0.874023	1.482859
7	1	0	-0.131628	-1.225282	-1.453762
8	1	0	-0.418222	-2.394952	-0.145975
9	1	0	-0.416496	1.521767	1.544472
10	1	0	0.007632	2.642912	0.248090
11	1	0	-2.319954	1.764769	-0.011746
12	1	0	-1.303586	1.284562	-1.378366
13	16	0	-1.915143	-0.564205	0.040553
14	9	0	1.986262	-1.426569	0.088179
15	6	0	2.104784	1.247616	-0.403556
16	1	0	2.195075	2.310229	-0.602381
17	1	0	2.950846	0.616443	-0.646549

11a

Standard orientation:

Center Number	Atomic Number	Atomic Type	Coordinates (Angstroms) X	Y	Z
1	6	0	1.145028	1.377166	0.018542
2	6	0	-0.250613	1.302584	0.695305
3	6	0	-1.091467	0.319649	-0.064394
4	6	0	-0.436144	-1.046989	-0.233583
5	6	0	0.944933	-0.867102	-0.869043
6	1	0	-0.123754	0.945007	1.721899
7	1	0	-0.739586	2.277242	0.713320
8	1	0	1.036218	1.842657	-0.966799
9	1	0	1.796678	2.019179	0.612308
10	1	0	-1.077873	-1.718088	-0.804999
11	1	0	1.441465	-1.838329	-0.916036
12	1	0	0.799526	-0.521224	-1.898708
13	7	0	1.782896	0.081228	-0.155697
14	1	0	2.050046	-0.302735	0.743454
15	9	0	-0.264114	-1.603064	1.046980
16	8	0	-2.144548	0.580428	-0.602293

11e Standard orientation:

Center Number	Atomic Number	Atomic Type	Coordinates (Angstroms) X	Y	Z
1	6	0	-0.244869	-1.444433	-0.288074
2	6	0	0.797670	-0.546946	0.409237
3	6	0	0.521451	0.912418	0.096747
4	6	0	-0.897876	1.323082	0.384131
5	6	0	-1.875952	0.340705	-0.302407
6	1	0	0.747034	-0.691200	1.493824
7	1	0	-0.081713	-1.378737	-1.369182
8	1	0	-0.078731	-2.479902	0.011889
9	1	0	-1.060041	1.281881	1.466906
10	1	0	-1.051796	2.349430	0.047319
11	1	0	-2.898949	0.582856	-0.012131
12	1	0	-1.806922	0.467931	-1.387711
13	1	0	-1.832201	-1.251243	0.980208
14	9	0	2.071457	-0.903758	-0.005733
15	7	0	-1.609014	-1.054729	0.011137
16	8	0	1.360095	1.640869	-0.381910

12a Standard orientation:

Center Number	Atomic Number	Atomic Type	Coordinates (Angstroms) X	Y	Z
1	6	0	1.106660	1.584579	-0.016327
2	6	0	-0.054283	1.124374	0.917861
3	6	0	-0.778044	0.020914	0.224758
4	6	0	0.122736	-1.131794	-0.138395
5	6	0	1.249916	-0.600107	-1.041088
6	1	0	0.385112	0.749328	1.847622
7	1	0	-0.721730	1.955154	1.144939
8	1	0	0.671014	2.052894	-0.905055
9	1	0	1.698218	2.339699	0.502523
10	1	0	-0.422425	-1.951978	-0.603866
11	1	0	1.936037	-1.420140	-1.260762
12	1	0	0.800210	-0.275455	-1.985255
13	7	0	1.974544	0.502055	-0.439967
14	1	0	2.529197	0.168600	0.339638
15	9	0	0.714825	-1.633707	1.035582
16	16	0	-2.313298	0.098943	-0.312569

12e Standard orientation:

Center Number	Atomic Number	Atomic Type	Coordinates (Angstroms) X	Y	Z
1	6	0	1.222374	1.157858	-0.426248
2	6	0	-0.036477	0.995129	0.464056
3	6	0	-0.592412	-0.386046	0.287276
4	6	0	0.430023	-1.434226	0.599795
5	6	0	1.676048	-1.208201	-0.297791
6	1	0	0.232398	1.142172	1.515487
7	1	0	0.894057	1.118652	-1.470519
8	1	0	1.650192	2.144016	-0.242068
9	1	0	0.740203	-1.337172	1.646522
10	1	0	0.015273	-2.430649	0.448445
11	1	0	2.447107	-1.930100	-0.026914
12	1	0	1.398244	-1.394293	-1.340263
13	1	0	2.667667	0.270182	0.703787
14	9	0	-0.944216	1.986983	0.128898
15	7	0	2.216519	0.134194	-0.193201
16	16	0	-2.078760	-0.697006	-0.300293

13a Standard orientation:

Center Number	Atomic Number	Atomic Type	Coordinates (Angstroms) X	Y	Z
1	6	0	1.234765	1.327779	-0.008684
2	6	0	-0.149208	1.327078	0.676960
3	6	0	-1.061766	0.376603	-0.040962
4	6	0	-0.474666	-1.009192	-0.225526
5	6	0	0.912705	-0.915569	-0.859625
6	1	0	-0.027548	0.993992	1.713299
7	1	0	-0.588047	2.326141	0.683801
8	1	0	1.140435	1.779456	-1.002639
9	1	0	1.928037	1.945182	0.564925
10	1	0	-1.138598	-1.655986	-0.803565
11	1	0	1.356966	-1.912642	-0.893038
12	1	0	0.781584	-0.578047	-1.894247
13	7	0	1.808438	-0.003083	-0.168276
14	1	0	2.052601	-0.386424	0.737946
15	9	0	-0.338097	-1.594060	1.050780
16	7	0	-2.178381	0.748818	-0.520296
17	1	0	-2.643942	-0.025471	-0.996486

13e Standard orientation:

Center Number	Atomic Number	Atomic Type	Coordinates (Angstroms) X	Y	Z
1	6	0	-0.291369	-1.432873	-0.298953
2	6	0	0.755061	-0.569243	0.423970
3	6	0	0.527894	0.892930	0.117084
4	6	0	-0.877344	1.335695	0.407707
5	6	0	-1.872725	0.401209	-0.309672
6	1	0	0.696684	-0.729717	1.505263
7	1	0	-0.107862	-1.356648	-1.376362
8	1	0	-0.160889	-2.476639	-0.009188
9	1	0	-1.057321	1.279886	1.487384
10	1	0	-1.005534	2.372289	0.090961
11	1	0	-2.895108	0.662992	-0.032647
12	1	0	-1.780815	0.544731	-1.391798
13	1	0	-1.893900	-1.213550	0.943627
14	9	0	2.033666	-0.958234	0.019808
15	7	0	-1.652636	-1.010806	-0.020141
16	7	0	1.392924	1.669016	-0.393705
17	1	0	2.270634	1.166983	-0.539408

14a Standard orientation:

Center Number	Atomic Number	Atomic Type	Coordinates (Angstroms) X	Y	Z
1	6	0	1.071434	1.451207	0.013946
2	6	0	-0.315120	1.285257	0.676885
3	6	0	-1.097098	0.233206	-0.059082
4	6	0	-0.349778	-1.066828	-0.246445
5	6	0	1.024714	-0.807301	-0.859366
6	1	0	-0.173764	0.961386	1.713458
7	1	0	-0.858237	2.233302	0.686624
8	1	0	0.940924	1.899336	-0.977263
9	1	0	1.679431	2.137938	0.605008
10	1	0	-0.938338	-1.770375	-0.836067
11	1	0	1.583557	-1.744801	-0.894319
12	1	0	0.870431	-0.476089	-1.892843
13	7	0	1.797713	0.197943	-0.146851
14	1	0	2.072740	-0.162219	0.760021
15	9	0	-0.158821	-1.644156	1.028813
16	7	0	-2.245791	0.352696	-0.588948
17	1	0	-2.615723	1.291207	-0.428970

14e

Standard orientation:

Center Number	Atomic Number	Atomic Type	Coordinates (Angstroms) X	Y	Z
1	6	0	-0.278723	-1.435350	-0.316514
2	6	0	0.773725	-0.578697	0.409138
3	6	0	0.545604	0.883473	0.114120
4	6	0	-0.864987	1.314200	0.426982
5	6	0	-1.867118	0.388337	-0.292825
6	1	0	0.701053	-0.741204	1.490294
7	1	0	-0.103181	-1.345657	-1.394401
8	1	0	-0.140131	-2.481902	-0.040551
9	1	0	-1.029830	1.239841	1.507762
10	1	0	-1.014502	2.356364	0.134027
11	1	0	-2.886119	0.646164	-0.000076
12	1	0	-1.787132	0.546949	-1.373667
13	1	0	-1.873898	-1.241395	0.939228
14	9	0	2.041168	-0.993578	0.016244
15	7	0	-1.640841	-1.025636	-0.023803
16	7	0	1.466951	1.594959	-0.391988
17	1	0	1.129453	2.546002	-0.543684

15a

Standard orientation:

Center Number	Atomic Number	Atomic Type	Coordinates (Angstroms) X	Y	Z
1	6	0	1.144634	1.423515	-0.017269
2	6	0	-0.225191	1.309802	0.676523
3	6	0	-1.069969	0.278050	-0.019885
4	6	0	-0.378674	-1.038319	-0.233744
5	6	0	0.999034	-0.843706	-0.860756
6	1	0	-0.064404	1.007897	1.718052
7	1	0	-0.731520	2.278154	0.686924
8	1	0	1.007296	1.858810	-1.013985
9	1	0	1.795053	2.096031	0.545901
10	1	0	-0.987408	-1.727065	-0.822952
11	1	0	1.522268	-1.802257	-0.887697
12	1	0	0.845925	-0.521806	-1.897373
13	7	0	1.825300	0.140851	-0.176754
14	1	0	2.092338	-0.218608	0.733188
15	9	0	-0.189604	-1.656345	1.033323
16	6	0	-2.291674	0.498189	-0.498166
17	1	0	-2.845687	-0.280130	-1.013650
18	1	0	-2.773486	1.464945	-0.391261

15e

Standard orientation:

Center Number	Atomic Number	Atomic Type	Coordinates (Angstroms) X	Y	Z
1	6	0	-0.369119	-1.419264	-0.328987
2	6	0	0.709689	-0.626078	0.422070
3	6	0	0.572053	0.842619	0.143516
4	6	0	-0.815015	1.347267	0.444019
5	6	0	-1.855776	0.495594	-0.303877
6	1	0	0.621885	-0.811319	1.498904
7	1	0	-0.169869	-1.325859	-1.402682
8	1	0	-0.297805	-2.475886	-0.063304
9	1	0	-1.010305	1.268213	1.520836
10	1	0	-0.906383	2.400363	0.166784
11	1	0	-2.866425	0.799816	-0.023781
12	1	0	-1.748324	0.664533	-1.381314
13	1	0	-1.970896	-1.148208	0.901721
14	9	0	1.967696	-1.107756	0.037519
15	7	0	-1.714376	-0.936408	-0.056461
16	6	0	1.546523	1.584721	-0.373615
17	1	0	1.389134	2.638122	-0.580268
18	1	0	2.520227	1.165731	-0.598098

16a Standard orientation:

Center Number	Atomic Number	Atomic Type	Coordinates (Angstroms) X	Y	Z
1	6	0	-1.300531	-1.253861	0.034896
2	6	0	0.108377	-1.356182	0.644001
3	6	0	1.039051	-0.439593	-0.101139
4	6	0	0.524639	0.994359	-0.256734
5	6	0	-0.899099	0.983888	-0.787574
6	1	0	0.063727	-1.027062	1.687256
7	1	0	0.491601	-2.376001	0.606011
8	1	0	-1.280996	-1.678986	-0.982123
9	1	0	-1.993412	-1.850179	0.629891
10	1	0	1.193913	1.565129	-0.900925
11	1	0	-1.288119	2.003934	-0.788672
12	1	0	-0.845220	0.643539	-1.836643
13	7	0	-1.736639	0.135949	0.046002
14	9	0	0.557264	1.585103	1.016087
15	1	0	-2.701496	0.205914	-0.249581
16	8	0	2.083309	-0.784442	-0.604089

16e Standard orientation:

Center Number	Atomic Number	Atomic Type	Coordinates (Angstroms) X	Y	Z
1	6	0	-0.288676	-1.435382	-0.260495
2	6	0	0.784644	-0.571248	0.403880
3	6	0	0.551612	0.898934	0.085859
4	6	0	-0.861230	1.361750	0.340845
5	6	0	-1.868963	0.386946	-0.283024
6	1	0	0.744429	-0.702785	1.489926
7	1	0	-0.157337	-1.372765	-1.353054
8	1	0	-0.146680	-2.473727	0.041905
9	1	0	-1.016632	1.387209	1.425040
10	1	0	-0.979486	2.371368	-0.053724
11	1	0	-2.879859	0.666645	0.016258
12	1	0	-1.810508	0.464954	-1.380606
13	9	0	2.039703	-0.964897	-0.030098
14	7	0	-1.588644	-0.963738	0.187761
15	1	0	-2.311896	-1.602855	-0.115254
16	8	0	1.427105	1.605774	-0.354541

17a Standard orientation:

Center Number	Atomic Number	Atomic Type	Coordinates (Angstroms) X	Y	Z
1	6	0	-1.178912	-1.557672	0.017315
2	6	0	0.031350	-1.155779	0.883102
3	6	0	0.773523	-0.066059	0.176133
4	6	0	-0.096994	1.125978	-0.170086
5	6	0	-1.310286	0.651164	-0.962184
6	1	0	-0.342790	-0.770139	1.837372
7	1	0	0.680478	-2.009856	1.070943
8	1	0	-0.806627	-2.013918	-0.914655
9	1	0	-1.764912	-2.308453	0.549175
10	1	0	0.463855	1.880078	-0.720955
11	1	0	-1.976783	1.499518	-1.128800
12	1	0	-0.936806	0.314621	-1.944303
13	7	0	-2.009620	-0.387942	-0.225031
14	9	0	-0.519612	1.718638	1.030535
15	1	0	-2.858363	-0.647451	-0.710369
16	16	0	2.310856	-0.167646	-0.337731

17e Standard orientation:

Center Number	Atomic Number	Atomic Type	Coordinates (Angstroms) X	Y	Z
1	6	0	1.239355	1.155253	-0.385420
2	6	0	-0.033392	0.992197	0.454681
3	6	0	-0.599513	-0.388703	0.255781
4	6	0	0.414214	-1.459923	0.543553
5	6	0	1.689446	-1.209940	-0.276487
6	1	0	0.207415	1.122572	1.514925
7	1	0	0.953771	1.102206	-1.448625
8	1	0	1.661515	2.141886	-0.190308
9	1	0	0.673645	-1.415807	1.607713
10	1	0	-0.006014	-2.441093	0.327095
11	1	0	2.446846	-1.942129	0.005999
12	1	0	1.459538	-1.353901	-1.344324
13	9	0	-0.930992	1.987237	0.105819
14	7	0	2.186285	0.128011	0.009749
15	1	0	3.077201	0.283866	-0.443640
16	16	0	-2.103728	-0.675758	-0.287632

18a Standard orientation:

Center Number	Atomic Number	Atomic Type	Coordinates (Angstroms) X	Y	Z
1	6	0	1.327996	1.244615	0.013806
2	6	0	-0.061525	1.353137	0.655270
3	6	0	-1.022766	0.452506	-0.066902
4	6	0	-0.530084	-0.974933	-0.237074
5	6	0	0.883337	-0.989085	-0.795814
6	1	0	0.009573	1.033433	1.700057
7	1	0	-0.431170	2.378736	0.627215
8	1	0	1.284852	1.668436	-1.003739
9	1	0	2.044359	1.833319	0.589061
10	1	0	-1.204294	-1.551398	-0.874181
11	1	0	1.257440	-2.014811	-0.799683
12	1	0	0.810031	-0.652688	-1.844989
13	1	0	2.707276	-0.226818	-0.320134
14	7	0	1.754601	-0.149700	0.011368
15	9	0	-0.544817	-1.587845	1.029309
16	7	0	-2.115999	0.887470	-0.544864
17	1	0	-2.626665	0.140566	-1.018637

18e Standard orientation:

Center Number	Atomic Number	Atomic Type	Coordinates (Angstroms) X	Y	Z
1	6	0	-0.322883	-1.421835	-0.276313
2	6	0	0.746309	-0.584837	0.423596
3	6	0	0.550551	0.882880	0.106794
4	6	0	-0.847105	1.366094	0.381387
5	6	0	-1.865930	0.435895	-0.286961
6	1	0	0.692238	-0.736565	1.505426
7	1	0	-0.165645	-1.339889	-1.364406
8	1	0	-0.207476	-2.468061	0.010513
9	1	0	-1.020358	1.357978	1.462978
10	1	0	-0.950204	2.390129	0.020485
11	1	0	-2.877620	0.726943	-0.000296
12	1	0	-1.782255	0.541178	-1.381055
13	1	0	-2.351533	-1.550082	-0.195210
14	9	0	2.009395	-1.002798	0.006102
15	7	0	-1.625932	-0.935703	0.149878
16	7	0	1.440494	1.638011	-0.390594
17	1	0	2.310719	1.118203	-0.519362

19a Standard orientation:

Center Number	Atomic Number	Atomic Type	Coordinates (Angstroms) X	Y	Z
1	6	0	1.195785	1.360344	0.034342
2	6	0	-0.206721	1.324452	0.656943
3	6	0	-1.062520	0.331207	-0.082892
4	6	0	-0.427338	-1.032888	-0.258448
5	6	0	0.987939	-0.901327	-0.794744
6	1	0	-0.119417	1.005620	1.700466
7	1	0	-0.668012	2.313931	0.635354
8	1	0	1.124195	1.785567	-0.980848
9	1	0	1.841060	2.013135	0.624344
10	1	0	-1.043079	-1.654926	-0.908548
11	1	0	1.464223	-1.883665	-0.798728
12	1	0	0.898137	-0.566075	-1.842857
13	1	0	2.720006	0.037470	-0.285122
14	7	0	1.759238	0.015868	0.030718
15	9	0	-0.396621	-1.658236	1.004167
16	7	0	-2.198451	0.538422	-0.611131
17	1	0	-2.495911	1.502316	-0.449878

19e Standard orientation:

Center Number	Atomic Number	Atomic Type	Coordinates (Angstroms) X	Y	Z
1	6	0	-0.318603	-1.424712	-0.289445
2	6	0	0.761739	-0.598615	0.409116
3	6	0	0.572555	0.869949	0.104145
4	6	0	-0.828755	1.349035	0.399501
5	6	0	-1.857527	0.433003	-0.272724
6	1	0	0.696168	-0.750551	1.491297
7	1	0	-0.167557	-1.335200	-1.378066
8	1	0	-0.201691	-2.473304	-0.011612
9	1	0	-0.988740	1.322014	1.482685
10	1	0	-0.948157	2.380637	0.061822
11	1	0	-2.864781	0.727032	0.026639
12	1	0	-1.782063	0.552259	-1.365972
13	1	0	-2.347858	-1.551351	-0.204959
14	9	0	2.010452	-1.048330	-0.000123
15	7	0	-1.619902	-0.943152	0.146777
16	7	0	1.522565	1.554201	-0.383899
17	1	0	1.215523	2.514140	-0.544424

20a Standard orientation:

Center Number	Atomic Number	Atomic Type	Coordinates (Angstroms) X	Y	Z
1	6	0	1.230200	1.356048	0.007843
2	6	0	-0.151867	1.332681	0.667943
3	6	0	-1.042743	0.345468	-0.038056
4	6	0	-0.431364	-1.014492	-0.239704
5	6	0	0.975661	-0.908313	-0.804760
6	1	0	-0.030846	1.034316	1.714943
7	1	0	-0.600021	2.328428	0.651641
8	1	0	1.132848	1.775610	-1.008351
9	1	0	1.903008	2.003687	0.573566
10	1	0	-1.053512	-1.639660	-0.882975
11	1	0	1.440163	-1.896674	-0.810641
12	1	0	0.867427	-0.580206	-1.853430
13	1	0	2.730410	0.017422	-0.360819
14	7	0	1.782174	0.005125	-0.008177
15	9	0	-0.374338	-1.674712	1.014016
16	6	0	-2.244241	0.634753	-0.529257
17	1	0	-2.836403	-0.109735	-1.052131
18	1	0	-2.673126	1.626481	-0.424760

20e Standard orientation:

Center Number	Atomic Number	Atomic Type	Coordinates (Angstroms) X	Y	Z
1	6	0	-0.401859	-1.406787	-0.302556
2	6	0	0.700343	-0.639567	0.424723
3	6	0	0.594609	0.831373	0.134545
4	6	0	-0.781323	1.376712	0.422876
5	6	0	-1.846088	0.531275	-0.281504
6	1	0	0.619318	-0.816963	1.502120
7	1	0	-0.224300	-1.309684	-1.386709
8	1	0	-0.346322	-2.464033	-0.036419
9	1	0	-0.970231	1.341424	1.502171
10	1	0	-0.847678	2.418857	0.103793
11	1	0	-2.844002	0.867496	0.006827
12	1	0	-1.744699	0.665326	-1.371434
13	1	0	-2.436144	-1.428174	-0.272612
14	9	0	1.939601	-1.151446	0.023801
15	7	0	-1.688400	-0.865849	0.112664
16	6	0	1.590945	1.547281	-0.376497
17	1	0	1.458652	2.602045	-0.593904
18	1	0	2.558035	1.105949	-0.586217

21a Standard orientation:

Center Number	Atomic Number	Atomic Type	Coordinates (Angstroms) X	Y	Z
1	6	0	1.197880	1.375324	0.029934
2	6	0	-0.203559	1.333256	0.672992
3	6	0	-1.073966	0.375277	-0.092219
4	6	0	-0.497186	-1.034242	-0.234499
5	6	0	0.889925	-0.975689	-0.850972
6	1	0	-0.113350	0.967721	1.701828
7	1	0	-0.675831	2.315808	0.691304
8	1	0	1.120234	1.824466	-0.966501
9	1	0	1.841391	2.027028	0.624626
10	1	0	-1.186102	-1.665287	-0.796408
11	1	0	1.302074	-1.987132	-0.889570
12	1	0	0.773774	-0.636025	-1.886808
13	1	0	2.776333	0.032594	-0.585362
14	9	0	-0.404417	-1.575592	1.064102
15	6	0	1.808413	-0.022172	-0.081983
16	1	0	1.992171	-0.423788	0.919589
17	8	0	-2.114997	0.676802	-0.631142

21e Standard orientation:

Center Number	Atomic Number	Atomic Type	Coordinates (Angstroms) X	Y	Z
1	6	0	-0.228698	-1.457942	-0.295987
2	6	0	0.797476	-0.567688	0.396441
3	6	0	0.546396	0.899222	0.077778
4	6	0	-0.856920	1.357657	0.387135
5	6	0	-1.892027	0.439969	-0.286383
6	1	0	0.735404	-0.693480	1.483794
7	1	0	-0.081292	-1.380361	-1.378809
8	1	0	-0.045467	-2.495418	-0.008355
9	1	0	-0.990773	1.311542	1.474924
10	1	0	-0.963944	2.397357	0.075360
11	1	0	-2.894934	0.749026	0.016148
12	1	0	-1.831590	0.567313	-1.372829
13	1	0	-2.366520	-1.663809	-0.450742
14	9	0	2.082858	-0.928501	0.013301
15	1	0	-1.813782	-1.175035	1.144661
16	6	0	-1.649848	-1.025758	0.071232
17	8	0	1.401113	1.608327	-0.400645

22a Standard orientation:

Center Number	Atomic Number	Atomic Type	Coordinates (Angstroms) X	Y	Z
1	6	0	1.131276	1.608889	0.012556
2	6	0	-0.052799	1.150394	0.893190
3	6	0	-0.782863	0.053209	0.184835
4	6	0	0.088105	-1.141283	-0.147061
5	6	0	1.263806	-0.700543	-1.009029
6	1	0	0.343072	0.754359	1.835613
7	1	0	-0.726644	1.976115	1.118521
8	1	0	0.733510	2.070264	-0.898036
9	1	0	1.688726	2.379912	0.548836
10	1	0	-0.492134	-1.937113	-0.612277
11	1	0	1.900607	-1.568376	-1.197171
12	1	0	0.859646	-0.378698	-1.975108
13	1	0	2.829869	0.778322	-1.032535
14	9	0	0.585122	-1.660856	1.067455
15	6	0	2.044810	0.439963	-0.352260
16	1	0	2.541700	0.068654	0.549323
17	16	0	-2.318654	0.146281	-0.339853

22e Standard orientation:

Center Number	Atomic Number	Atomic Type	Coordinates (Angstroms) X	Y	Z
1	6	0	1.189677	1.211091	-0.418793
2	6	0	-0.052739	1.001265	0.446374
3	6	0	-0.596036	-0.390312	0.256984
4	6	0	0.421687	-1.444630	0.588213
5	6	0	1.686339	-1.255313	-0.271049
6	1	0	0.199491	1.125654	1.506207
7	1	0	0.886541	1.152912	-1.469724
8	1	0	1.570613	2.217610	-0.234438
9	1	0	0.693529	-1.336707	1.646153
10	1	0	-0.010774	-2.434706	0.449247
11	1	0	2.426399	-2.003185	0.021195
12	1	0	1.434574	-1.441113	-1.320672
13	1	0	3.102288	0.296249	-0.785837
14	9	0	-0.992845	1.978614	0.143695
15	1	0	2.624224	0.290467	0.905912
16	6	0	2.251821	0.153945	-0.115549
17	16	0	-2.087236	-0.708187	-0.308024

23a Standard orientation:

Center Number	Atomic Number	Atomic Type	Coordinates (Angstroms) X	Y	Z
1	6	0	1.256599	1.347945	0.003361
2	6	0	-0.131249	1.344161	0.666649
3	6	0	-1.051944	0.407977	-0.063544
4	6	0	-0.516137	-1.004591	-0.222094
5	6	0	0.867709	-0.999098	-0.848298
6	1	0	-0.029614	0.997309	1.701411
7	1	0	-0.573016	2.341382	0.685258
8	1	0	1.173305	1.787820	-0.997335
9	1	0	1.930407	1.987036	0.578996
10	1	0	-1.215085	-1.627577	-0.784707
11	1	0	1.247155	-2.023786	-0.877209
12	1	0	0.751360	-0.668786	-1.887089
13	1	0	2.788674	-0.048476	-0.621380
14	9	0	-0.431222	-1.570863	1.070449
15	6	0	1.826432	-0.066691	-0.104130
16	1	0	2.012360	-0.463633	0.899269
17	7	0	-2.153934	0.805480	-0.556050
18	1	0	-2.635468	0.039909	-1.030565

23e Standard orientation:

Center Number	Atomic Number	Atomic Type	Coordinates (Angstroms) X	Y	Z
1	6	0	-0.272489	-1.447298	-0.305106
2	6	0	0.756528	-0.585673	0.412588
3	6	0	0.550210	0.881297	0.098332
4	6	0	-0.838327	1.367285	0.411038
5	6	0	-1.888002	0.493780	-0.293705
6	1	0	0.688366	-0.729645	1.496261
7	1	0	-0.104291	-1.358595	-1.384017
8	1	0	-0.120533	-2.492438	-0.026652
9	1	0	-0.993193	1.305681	1.495350
10	1	0	-0.922223	2.415131	0.119472
11	1	0	-2.889110	0.821361	-0.003720
12	1	0	-1.803915	0.636987	-1.376929
13	1	0	-2.412726	-1.597862	-0.501578
14	9	0	2.047111	-0.977048	0.038315
15	1	0	-1.876185	-1.146117	1.110206
16	6	0	-1.690369	-0.984605	0.041887
17	7	0	1.429883	1.638970	-0.414116
18	1	0	2.295321	1.117434	-0.564613

24a Standard orientation:

Center Number	Atomic Number	Atomic Type	Coordinates (Angstroms) X	Y	Z
1	6	0	1.107991	1.460604	0.025643
2	6	0	-0.281442	1.307178	0.668392
3	6	0	-1.087706	0.279131	-0.079622
4	6	0	-0.405638	-1.063119	-0.244218
5	6	0	0.978494	-0.906904	-0.849706
6	1	0	-0.160077	0.965079	1.702440
7	1	0	-0.816241	2.259446	0.692202
8	1	0	0.994058	1.898469	-0.972740
9	1	0	1.703722	2.160245	0.616638
10	1	0	-1.039183	-1.737248	-0.821092
11	1	0	1.464474	-1.885339	-0.882704
12	1	0	0.843066	-0.579312	-1.887073
13	1	0	2.786305	0.238802	-0.583546
14	9	0	-0.276016	-1.632535	1.045504
15	1	0	-2.556660	1.397947	-0.470653
16	6	0	1.823098	0.113369	-0.083204
17	1	0	2.033999	-0.270136	0.920553
18	7	0	-2.225444	0.443328	-0.621038

24e Standard orientation:

Center Number	Atomic Number	Atomic Type	Coordinates (Angstroms) X	Y	Z
1	6	0	-0.260839	-1.448870	-0.325482
2	6	0	0.774773	-0.596557	0.398067
3	6	0	0.568203	0.870473	0.096102
4	6	0	-0.824705	1.344858	0.435276
5	6	0	-1.881553	0.483986	-0.274218
6	1	0	0.690161	-0.743185	1.481257
7	1	0	-0.098126	-1.345895	-1.404167
8	1	0	-0.102977	-2.497080	-0.062092
9	1	0	-0.964535	1.260202	1.519742
10	1	0	-0.928987	2.399744	0.171666
11	1	0	-2.878982	0.806845	0.033126
12	1	0	-1.809037	0.646139	-1.355421
13	1	0	-2.402115	-1.604008	-0.515474
14	9	0	2.054706	-1.014021	0.038207
15	1	0	1.180439	2.519705	-0.568105
16	1	0	-1.858969	-1.178388	1.101367
17	6	0	-1.678420	-0.998852	0.035093

18	7	0	1.499431	1.561697	-0.419256

25a

Standard orientation:

Center Number	Atomic Number	Atomic Type	Coordinates (Angstroms) X	Y	Z
1	6	0	1.167902	1.442923	-0.004851
2	6	0	-0.206892	1.325634	0.669906
3	6	0	-1.064372	0.310409	-0.038530
4	6	0	-0.422978	-1.037219	-0.230391
5	6	0	0.958812	-0.928214	-0.853365
6	1	0	-0.061434	1.006404	1.709128
7	1	0	-0.713953	2.292968	0.695606
8	1	0	1.040494	1.870201	-1.006845
9	1	0	1.798573	2.133519	0.561482
10	1	0	-1.068483	-1.704455	-0.804650
11	1	0	1.418702	-1.919804	-0.879938
12	1	0	0.818698	-0.609681	-1.892824
13	1	0	2.803647	0.167895	-0.632961
14	9	0	-0.283155	-1.644732	1.051125
15	6	0	-2.273987	0.563969	-0.530756
16	1	0	-2.846817	-0.199956	-1.047432
17	1	0	-2.729031	1.544915	-0.434804
18	6	0	1.845581	0.076375	-0.114767
19	1	0	2.063608	-0.302694	0.889644

25e

Standard orientation:

Center Number	Atomic Number	Atomic Type	Coordinates (Angstroms) X	Y	Z
1	6	0	-0.353407	-1.435531	-0.333459
2	6	0	0.710762	-0.641246	0.410991
3	6	0	0.593837	0.830493	0.125456
4	6	0	-0.774391	1.376982	0.447435
5	6	0	-1.866397	0.586482	-0.287675
6	1	0	0.615558	-0.810679	1.490235
7	1	0	-0.168197	-1.331693	-1.408532
8	1	0	-0.259938	-2.493520	-0.076031
9	1	0	-0.948045	1.292432	1.528283
10	1	0	-0.820714	2.438500	0.194317
11	1	0	-2.852434	0.956822	0.005374
12	1	0	-1.764820	0.754096	-1.366152
13	1	0	-2.498567	-1.470630	-0.557153
14	9	0	1.979839	-1.126546	0.054588
15	6	0	1.581032	1.552843	-0.394927
16	1	0	1.443689	2.609421	-0.600511
17	1	0	2.545365	1.115070	-0.623975
18	1	0	-1.956506	-1.087277	1.070093
19	6	0	-1.750427	-0.908963	0.007639

Vibrational frequencies (gas phase)

1a

	1	2	3
	A	A	A
Frequencies --	90.2151	158.2994	229.1940
Red. masses --	6.1826	3.5053	4.6487
Frc consts --	0.0296	0.0518	0.1439
IR Inten --	9.7251	0.5613	5.2886
	4	5	6
	A	A	A
Frequencies --	293.0812	396.0234	440.0788
Red. masses --	3.9482	2.1110	5.0952
Frc consts --	0.1998	0.1951	0.5814
IR Inten --	2.7653	7.8303	1.7235
	7	8	9
	A	A	A
Frequencies --	483.2138	506.0571	653.1741
Red. masses --	5.5501	2.3320	3.9689
Frc consts --	0.7635	0.3519	0.9976
IR Inten --	17.4440	4.1102	15.6659
	10	11	12
	A	A	A
Frequencies --	754.5997	795.8006	873.9698
Red. masses --	4.0653	2.4029	2.9389
Frc consts --	1.3639	0.8966	1.3226
IR Inten --	7.0868	16.1460	14.0293
	13	14	15
	A	A	A
Frequencies --	889.1817	943.5656	1023.4835
Red. masses --	2.3678	2.1215	3.1108
Frc consts --	1.1030	1.1128	1.9199
IR Inten --	16.2294	19.5896	2.0128
	16	17	18
	A	A	A
Frequencies --	1037.8478	1063.9652	1117.6337
Red. masses --	2.2359	2.1085	2.8512
Frc consts --	1.4189	1.4063	2.0984
IR Inten --	45.1435	26.1092	34.2440
	19	20	21
	A	A	A
Frequencies --	1155.5522	1213.4435	1264.4257
Red. masses --	3.7820	1.4356	1.9772
Frc consts --	2.9754	1.2454	1.8625
IR Inten --	33.3133	16.8224	96.2697
	22	23	24
	A	A	A
Frequencies --	1266.9803	1315.2988	1338.7756
Red. masses --	1.4113	1.5277	1.6007
Frc consts --	1.3348	1.5572	1.6903
IR Inten --	7.1947	9.9805	11.3537
	25	26	27
	A	A	A
Frequencies --	1352.8219	1373.5642	1396.9705
Red. masses --	1.2826	1.2265	1.3855
Frc consts --	1.3830	1.3634	1.5930
IR Inten --	5.9858	17.6849	9.6173
	28	29	30
	A	A	A
Frequencies --	1423.1928	1458.8702	1477.3201
Red. masses --	1.3607	1.1154	1.0879
Frc consts --	1.6238	1.3987	1.3990
IR Inten --	13.3370	15.3137	7.2247

	31	32	33
	A	A	A
Frequencies --	1518.1904	1852.6223	2994.7830
Red. masses --	1.0933	11.3679	1.0708
Frc consts --	1.4847	22.9881	5.6582
IR Inten --	4.9707	227.7048	34.8917

	34	35	36
	A	A	A
Frequencies --	2999.4689	3082.1603	3118.1068
Red. masses --	1.0718	1.0606	1.0883
Frc consts --	5.6812	5.9363	6.2340
IR Inten --	58.1900	3.3016	17.0046

	37	38	39
	A	A	A
Frequencies --	3138.7360	3141.0375	3154.9042
Red. masses --	1.0927	1.0942	1.1047
Frc consts --	6.3423	6.3604	6.4785
IR Inten --	24.8336	11.3269	6.9152

1e

	1	2	3
	A	A	A
Frequencies --	94.1883	153.0165	272.0407
Red. masses --	5.5425	2.4696	8.0933
Frc consts --	0.0290	0.0341	0.3529
IR Inten --	3.6178	6.3794	0.9925

	4	5	6
	A	A	A
Frequencies --	343.3677	354.6370	410.2270
Red. masses --	3.2946	3.6245	4.4191
Frc consts --	0.2289	0.2686	0.4382
IR Inten --	1.8425	20.2729	1.3111

	7	8	9
	A	A	A
Frequencies --	445.9960	520.7776	609.2475
Red. masses --	2.9505	3.2550	4.5710
Frc consts --	0.3458	0.5201	0.9996
IR Inten --	0.8196	6.0400	14.5056

	10	11	12
	A	A	A
Frequencies --	714.8336	764.8831	876.6929
Red. masses --	2.8265	2.5191	2.5534
Frc consts --	0.8510	0.8683	1.1563
IR Inten --	10.7592	3.3217	17.3645

	13	14	15
	A	A	A
Frequencies --	918.3870	997.4290	1011.3321
Red. masses --	2.6141	1.3716	2.9816
Frc consts --	1.2990	0.8039	1.7967
IR Inten --	16.3680	1.0440	37.9083

	16	17	18
	A	A	A
Frequencies --	1075.8909	1120.4653	1124.8296
Red. masses --	3.3646	3.4560	2.7504
Frc consts --	2.2947	2.5564	2.0503
IR Inten --	7.0021	31.8906	26.2756

	19	20	21
	A	A	A
Frequencies --	1159.8206	1192.9982	1250.5382
Red. masses --	4.3502	1.6962	1.8504
Frc consts --	3.4478	1.4223	1.7049
IR Inten --	105.6293	17.4950	86.3881

	22	23	24
	A	A	A
Frequencies --	1262.7889	1313.8745	1329.9708
Red. masses --	1.3390	1.4279	1.2706
Frc consts --	1.2581	1.4523	1.3241

```
 IR Inten    --      4.1598                 6.8478                 9.8727
```

```
                     25                     26                     27
                      A                      A                      A
 Frequencies --   1355.1818              1362.0086              1410.5360
 Red. masses --      1.2913                 1.3040                 1.4127
 Frc consts  --      1.3972                 1.4252                 1.6561
 IR Inten    --      2.1204                 5.5025                11.9437

                     28                     29                     30
                      A                      A                      A
 Frequencies --   1431.6409              1458.8130              1501.7671
 Red. masses --      1.4827                 1.1021                 1.0757
 Frc consts  --      1.7904                 1.3818                 1.4294
 IR Inten    --      3.3600                13.5711                 2.0597

                     31                     32                     33
                      A                      A                      A
 Frequencies --   1515.0188              1863.0100              3010.3475
 Red. masses --      1.0984                12.0419                 1.0714
 Frc consts  --      1.4854                24.6250                 5.7203
 IR Inten    --      7.2941               235.2296                37.9012

                     34                     35                     36
                      A                      A                      A
 Frequencies --   3014.0865              3063.5376              3070.2136
 Red. masses --      1.0702                 1.0762                 1.0694
 Frc consts  --      5.7282                 5.9512                 5.9392
 IR Inten    --     40.7318                 6.4662                20.7866

                     37                     38                     39
                      A                      A                      A
 Frequencies --   3139.0421              3143.4418              3147.4956
 Red. masses --      1.0940                 1.1024                 1.0962
 Frc consts  --      6.3514                 6.4177                 6.3986
 IR Inten    --     25.2180                 6.6208                15.8230
```

2a

```
                      1                      2                      3
                      A                      A                      A
 Frequencies --     85.1817               159.0900               242.9620
 Red. masses --      6.5074                 3.9866                 4.1267
 Frc consts  --      0.0278                 0.0594                 0.1435
 IR Inten    --      3.4478                 0.1076                 6.9522

                      4                      5                      6
                      A                      A                      A
 Frequencies --    269.3738               353.3672               406.6726
 Red. masses --      5.3602                 3.6517                 2.3444
 Frc consts  --      0.2292                 0.2687                 0.2284
 IR Inten    --      2.5230                 2.4451                 7.1606

                      7                      8                      9
                      A                      A                      A
 Frequencies --    419.7065               505.8886               634.1389
 Red. masses --      4.8885                 2.7032                 5.2046
 Frc consts  --      0.5074                 0.4076                 1.2331
 IR Inten    --      1.5017                 1.1919                 5.0280

                     10                     11                     12
                      A                      A                      A
 Frequencies --    711.1176               783.7898               870.0274
 Red. masses --      4.3526                 2.2485                 3.1448
 Frc consts  --      1.2968                 0.8139                 1.4025
 IR Inten    --     22.7696                19.5961                10.4982

                     13                     14                     15
                      A                      A                      A
 Frequencies --    881.7734               937.0309              1014.3188
 Red. masses --      2.5273                 2.1656                 3.0808
 Frc consts  --      1.1578                 1.1203                 1.8675
```

```
 IR Inten    --     13.9051                22.9091                11.1093

                     16                     17                     18
                      A                      A                      A
 Frequencies --   1036.7002              1053.6673              1095.9753
 Red. masses --      2.5525                 1.7948                 2.8046
 Frc consts  --      1.6163                 1.1740                 1.9848
 IR Inten    --     60.3124                 6.2775                71.0141

                     19                     20                     21
                      A                      A                      A
 Frequencies --   1156.6136              1186.5410              1241.9156
 Red. masses --      3.4481                 1.8549                 2.7868
 Frc consts  --      2.7178                 1.5387                 2.5324
 IR Inten    --     47.0805                22.2945                24.1006

                     22                     23                     24
                      A                      A                      A
 Frequencies --   1265.2864              1270.2297              1323.7035
 Red. masses --      1.5220                 1.4393                 1.4682
 Frc consts  --      1.4356                 1.3683                 1.5158
 IR Inten    --     17.4647                50.1552                34.7541

                     25                     26                     27
                      A                      A                      A
 Frequencies --   1340.6189              1342.2495              1388.1082
 Red. masses --      1.2703                 1.2997                 1.4790
 Frc consts  --      1.3451                 1.3796                 1.6791
 IR Inten    --     16.9430                24.9764                25.8284

                     28                     29                     30
                      A                      A                      A
 Frequencies --   1391.2030              1432.8628              1477.6152
 Red. masses --      1.4619                 1.4914                 1.1546
 Frc consts  --      1.6671                 1.8041                 1.4852
 IR Inten    --     33.5625                 1.8888                10.4742

                     31                     32                     33
                      A                      A                      A
 Frequencies --   1481.9536              1512.9413              3002.2585
 Red. masses --      1.0854                 1.0967                 1.0700
 Frc consts  --      1.4045                 1.4790                 5.6824
 IR Inten    --      6.6118                 6.9267                26.8751

                     34                     35                     36
                      A                      A                      A
 Frequencies --   3004.2452              3070.8657              3125.4928
 Red. masses --      1.0709                 1.0620                 1.0888
 Frc consts  --      5.6946                 5.9007                 6.2668
 IR Inten    --     55.2867                 3.3533                 9.0498

                     37                     38                     39
                      A                      A                      A
 Frequencies --   3135.8034              3142.8231              3149.8861
 Red. masses --      1.0938                 1.0945                 1.1032
 Frc consts  --      6.3372                 6.3693                 6.4488
 IR Inten    --     18.2210                20.7415                 4.5966
```

2e

```
                      1                      2                      3
                      A                      A                      A
 Frequencies --     81.4562               161.6959               231.8291
 Red. masses --      5.6144                 2.6560                14.3712
 Frc consts  --      0.0219                 0.0409                 0.4551
 IR Inten    --      0.9157                 3.6077                 0.6251

                      4                      5                      6
                      A                      A                      A
 Frequencies --    318.8163               359.8378               387.9086
 Red. masses --      3.6461                 4.2610                 2.7766
 Frc consts  --      0.2184                 0.3251                 0.2462
```

```
IR Inten    --      6.0851                11.4739                 3.2429




                       7                     8                      9
                       A                     A                      A
Frequencies --    447.7094              487.9864               579.6609
Red. masses --      3.2301                4.3631                 4.4945
Frc consts  --      0.3815                0.6121                 0.8898
IR Inten    --      0.6646                2.4563                 4.1887

                      10                    11                     12
                       A                     A                      A
Frequencies --    660.7033              742.0612               849.6491
Red. masses --      3.1308                3.0683                 2.1288
Frc consts  --      0.8052                0.9955                 0.9054
IR Inten    --     24.9134                2.8600                 8.7469

                      13                    14                     15
                       A                     A                      A
Frequencies --    912.4926             1004.8285              1014.2413
Red. masses --      2.5849                2.9469                 1.4581
Frc consts  --      1.2681                1.7531                 0.8837
IR Inten    --      4.1865               50.4676                 2.7224

                      16                    17                     18
                       A                     A                      A
Frequencies --   1052.5530             1096.0734              1120.1109
Red. masses --      2.9687                2.3888                 3.8054
Frc consts  --      1.9378                1.6909                 2.8130
IR Inten    --      4.8118               11.6526                27.7486

                      19                    20                     21
                       A                     A                      A
Frequencies --   1157.2890             1190.5901              1212.9339
Red. masses --      4.6255                1.7663                 1.8974
Frc consts  --      3.6500                1.4751                 1.6447
IR Inten    --    119.2508                8.3503                17.6627

                      22                    23                     24
                       A                     A                      A
Frequencies --   1249.1993             1297.1755              1321.8083
Red. masses --      1.7677                1.4818                 1.2894
Frc consts  --      1.6253                1.4690                 1.3274
IR Inten    --     51.3914                2.3220                 2.6166

                      25                    26                     27
                       A                     A                      A
Frequencies --   1340.3996             1354.8454              1367.9799
Red. masses --      1.3636                1.3918                 1.7213
Frc consts  --      1.4435                1.5052                 1.8979
IR Inten    --     47.0604               22.3242                65.6792

                      28                    29                     30
                       A                     A                      A
Frequencies --   1405.1931             1429.3791              1472.9646
Red. masses --      1.4230                1.4521                 1.1176
Frc consts  --      1.6555                1.7480                 1.4287
IR Inten    --      4.1666                8.3827                12.4383

                      31                    32                     33
                       A                     A                      A
Frequencies --   1504.8727             1519.5804              3015.4895
Red. masses --      1.0758                1.0995                 1.0699
Frc consts  --      1.4355                1.4958                 5.7318
IR Inten    --      1.4626                9.2009                20.1786

                      34                    35                     36
                       A                     A                      A
Frequencies --   3016.8531             3051.5115              3064.0367
Red. masses --      1.0701                1.0673                 1.0810
Frc consts  --      5.7384                5.8555                 5.9796
IR Inten    --     49.5582                3.5633                25.7750
```

	37	38	39
	A	A	A
Frequencies --	3141.9844	3144.8122	3145.3704
Red. masses --	1.0947	1.0990	1.0971
Frc consts --	6.3670	6.4038	6.3949
IR Inten --	26.4145	6.1111	13.2714

3a

	1	2	3
	A	A	A
Frequencies --	96.0723	162.1047	234.2382
Red. masses --	4.6464	3.5014	4.6083
Frc consts --	0.0253	0.0542	0.1490
IR Inten --	4.3552	0.1430	3.8990

	4	5	6
	A	A	A
Frequencies --	296.8566	397.4849	435.2084
Red. masses --	3.5237	2.0541	3.7027
Frc consts --	0.1830	0.1912	0.4132
IR Inten --	4.6961	9.6806	9.4382

	7	8	9
	A	A	A
Frequencies --	472.8064	503.6122	654.3191
Red. masses --	5.5882	2.1616	4.2204
Frc consts --	0.7360	0.3230	1.0646
IR Inten --	9.3184	4.9816	5.5600

	10	11	12
	A	A	A
Frequencies --	744.6796	798.7577	877.0321
Red. masses --	3.2767	2.5477	2.7158
Frc consts --	1.0706	0.9577	1.2308
IR Inten --	32.2087	9.2357	11.5872

	13	14	15
	A	A	A
Frequencies --	883.7838	939.6472	953.8197
Red. masses --	2.1420	1.9105	1.5695
Frc consts --	0.9858	0.9939	0.8413
IR Inten --	7.1905	92.6773	12.8062

	16	17	18
	A	A	A
Frequencies --	1023.4960	1047.5542	1081.6811
Red. masses --	3.0259	2.2268	1.9449
Frc consts --	1.8676	1.4397	1.3407
IR Inten --	1.4134	46.3468	16.4478

	19	20	21
	A	A	A
Frequencies --	1115.3937	1144.4900	1186.4400
Red. masses --	2.4248	1.7604	3.2351
Frc consts --	1.7774	1.3586	2.6831
IR Inten --	26.7862	52.8334	80.6159

	22	23	24
	A	A	A
Frequencies --	1229.2991	1272.2467	1321.1200
Red. masses --	1.2285	1.3629	1.4726
Frc consts --	1.0938	1.2998	1.5143
IR Inten --	4.2730	49.4622	14.3071

	25	26	27
	A	A	A
Frequencies --	1337.5613	1348.1776	1368.6074
Red. masses --	1.3970	1.3681	1.2534
Frc consts --	1.4726	1.4651	1.3832
IR Inten --	12.4124	2.4568	14.1183

	28	29	30
	A	A	A
Frequencies --	1379.8519	1417.7895	1425.7697
Red. masses --	1.2853	1.6465	1.5171
Frc consts --	1.4419	1.9500	1.8170
IR Inten --	8.5917	24.7868	5.2108

	31	32	33
	A	A	A
Frequencies --	1463.3872	1475.5056	1514.4159
Red. masses --	1.1427	1.0907	1.0953
Frc consts --	1.4417	1.3991	1.4801
IR Inten --	15.4724	7.8643	5.2149
	34	35	36
	A	A	A
Frequencies --	1766.8300	2986.9284	2997.6581
Red. masses --	9.2553	1.0709	1.0717
Frc consts --	17.0228	5.6291	5.6738
IR Inten --	77.0467	37.9166	57.5891
	37	38	39
	A	A	A
Frequencies --	3077.3597	3077.5789	3135.3312
Red. masses --	1.0636	1.0858	1.0924
Frc consts --	5.9345	6.0590	6.3272
IR Inten --	3.2519	36.8276	24.8356
	40	41	42
	A	A	A
Frequencies --	3138.3259	3148.5777	3479.8139
Red. masses --	1.0940	1.1031	1.0745
Frc consts --	6.3482	6.4431	7.6661
IR Inten --	18.7402	7.2695	1.5070

3e

	1	2	3
	A	A	A
Frequencies --	111.2512	162.3384	290.9333
Red. masses --	4.4253	2.4835	4.9406
Frc consts --	0.0323	0.0386	0.2464
IR Inten --	0.8022	3.7622	4.1100
	4	5	6
	A	A	A
Frequencies --	346.2823	358.0566	408.0011
Red. masses --	3.3374	3.6330	4.0859
Frc consts --	0.2358	0.2744	0.4007
IR Inten --	5.0409	22.4262	0.5179
	7	8	9
	A	A	A
Frequencies --	449.2057	511.5816	600.8234
Red. masses --	2.8622	3.1644	4.2207
Frc consts --	0.3403	0.4880	0.8977
IR Inten --	0.6060	8.2754	13.9903
	10	11	12
	A	A	A
Frequencies --	704.4835	779.9219	878.8134
Red. masses --	2.5656	2.6139	2.3008
Frc consts --	0.7502	0.9368	1.0470
IR Inten --	13.7191	2.5585	10.7170
	13	14	15
	A	A	A
Frequencies --	907.1956	934.8745	1010.4959
Red. masses --	2.4156	1.2248	2.9127
Frc consts --	1.1713	0.6307	1.7523
IR Inten --	33.0019	41.7178	28.0331
	16	17	18
	A	A	A
Frequencies --	1050.2359	1075.4929	1106.2286
Red. masses --	1.7026	2.2811	2.8797
Frc consts --	1.1065	1.5546	2.0763
IR Inten --	10.0269	44.9244	62.0011
	19	20	21
	A	A	A
Frequencies --	1123.0775	1142.8069	1183.1417
Red. masses --	2.7131	2.7055	3.3177

```
 Frc consts  --      2.0162                 2.0818                 2.7363
 IR Inten    --      4.3655                44.5831                53.8128



                     22                     23                     24
                      A                      A                      A
 Frequencies --   1229.2260              1263.5092              1320.3472
 Red. masses --      1.2021                 1.3562                 1.3602
 Frc consts  --      1.0701                 1.2757                 1.3971
 IR Inten    --     11.3004                36.1156                 5.6279

                     25                     26                     27
                      A                      A                      A
 Frequencies --   1327.7333              1335.8567              1367.2404
 Red. masses --      1.3102                 1.2065                 1.2999
 Frc consts  --      1.3608                 1.2686                 1.4317
 IR Inten    --     34.4820                 7.4907                 2.2529

                     28                     29                     30
                      A                      A                      A
 Frequencies --   1379.0324              1420.9929              1437.2644
 Red. masses --      1.3524                 1.4195                 1.7021
 Frc consts  --      1.5153                 1.6887                 2.0717
 IR Inten    --      4.6817                31.8863                 5.0712

                     31                     32                     33
                      A                      A                      A
 Frequencies --   1464.8744              1502.1101              1514.8425
 Red. masses --      1.1346                 1.0802                 1.0993
 Frc consts  --      1.4344                 1.4361                 1.4863
 IR Inten    --     14.7587                 2.1409                 7.7836

                     34                     35                     36
                      A                      A                      A
 Frequencies --   1772.0739              3007.5790              3010.6178
 Red. masses --      9.9429                 1.0710                 1.0698
 Frc consts  --     18.3962                 5.7077                 5.7130
 IR Inten    --     91.1539                33.5191                47.6643

                     37                     38                     39
                      A                      A                      A
 Frequencies --   3063.5713              3081.2957              3135.7376
 Red. masses --      1.0646                 1.0837                 1.0942
 Frc consts  --      5.8871                 6.0622                 6.3392
 IR Inten    --      8.2569                23.0740                29.1097

                     40                     41                     42
                      A                      A                      A
 Frequencies --   3141.8099              3142.5963              3494.2290
 Red. masses --      1.1006                 1.0965                 1.0735
 Frc consts  --      6.4009                 6.3805                 7.7227
 IR Inten    --      5.6331                21.2168                 5.1470
```

4a

```
                      1                      2                      3
                      A                      A                      A
 Frequencies --    104.9947               167.9402               234.4443
 Red. masses --      4.7432                 3.7659                 4.0465
 Frc consts  --      0.0308                 0.0626                 0.1310
 IR Inten    --      4.9104                 0.8386                 6.8122

                      4                      5                      6
                      A                      A                      A
 Frequencies --    298.8838               400.1190               437.3846
 Red. masses --      3.4939                 2.0839                 4.4104
 Frc consts  --      0.1839                 0.1966                 0.4971
 IR Inten    --      1.9791                 6.5421                 4.5345

                      7                      8                      9
                      A                      A                      A
 Frequencies --    473.1344               506.6870               658.6518
 Red. masses --      4.2648                 2.2697                 4.1303
 Frc consts  --      0.5625                 0.3433                 1.0557
```

```
 IR Inten    --     17.2291                1.4407                 5.7864



                     10                     11                     12
                      A                      A                      A
 Frequencies --    752.5469               795.8973               875.6270
 Red. masses --      3.8933                 2.4634                 3.0004
 Frc consts  --      1.2991                 0.9194                 1.3554
 IR Inten    --     15.5920                14.1610                11.7044

                     13                     14                     15
                      A                      A                      A
 Frequencies --    885.6564               935.9856               952.7139
 Red. masses --      2.0109                 1.7348                 1.6087
 Frc consts  --      0.9293                 0.8955                 0.8603
 IR Inten    --     32.8281                 7.3913                45.1164

                     16                     17                     18
                      A                      A                      A
 Frequencies --   1023.3868              1044.9560              1084.4843
 Red. masses --      3.1625                 2.2017                 1.9597
 Frc consts  --      1.9514                 1.4165                 1.3580
 IR Inten    --      1.8783                27.8017                80.3168

                     19                     20                     21
                      A                      A                      A
 Frequencies --   1112.7668              1145.5979              1183.9292
 Red. masses --      2.4014                 1.7063                 3.3428
 Frc consts  --      1.7519                 1.3194                 2.7607
 IR Inten    --     27.4773                19.4785                83.8751

                     22                     23                     24
                      A                      A                      A
 Frequencies --   1231.8836              1274.2559              1325.0031
 Red. masses --      1.2269                 1.3600                 1.4534
 Frc consts  --      1.0970                 1.3010                 1.5033
 IR Inten    --      2.4901                47.5169                10.9471

                     25                     26                     27
                      A                      A                      A
 Frequencies --   1332.9623              1360.6809              1375.8834
 Red. masses --      1.3894                 1.3327                 1.2455
 Frc consts  --      1.4545                 1.4537                 1.3892
 IR Inten    --      0.1009                 2.7078                17.4678

                     28                     29                     30
                      A                      A                      A
 Frequencies --   1378.6885              1426.2719              1428.1978
 Red. masses --      1.3485                 1.5675                 1.6691
 Frc consts  --      1.5102                 1.8787                 2.0059
 IR Inten    --     18.3189                 5.6936                51.5133


                     31                     32                     33
                      A                      A                      A
 Frequencies --   1465.0105              1480.5253              1517.3327
 Red. masses --      1.1102                 1.0897                 1.0958
 Frc consts  --      1.4038                 1.4074                 1.4865
 IR Inten    --     13.4626                 7.6393                 5.0096

                     34                     35                     36
                      A                      A                      A
 Frequencies --   1770.5709              2992.0122              2998.4561
 Red. masses --      9.2331                 1.0716                 1.0705
 Frc consts  --     17.0539                 5.6522                 5.6707
 IR Inten    --     79.2452                48.6078                48.5974

                     37                     38                     39
                      A                      A                      A
 Frequencies --   3069.9226              3120.7733              3126.8172
 Red. masses --      1.0593                 1.0883                 1.1024
 Frc consts  --      5.8820                 6.2451                 6.3505
 IR Inten    --     10.9633                16.3251                18.3448
```

	40	41	42
	A	A	A
Frequencies --	3133.0197	3139.1061	3484.6586
Red. masses --	1.0966	1.0939	1.0744
Frc consts --	6.3417	6.3508	7.6870
IR Inten --	22.3238	14.6065	1.7274

4e

	1	2	3
	A	A	A
Frequencies --	99.8283	162.7613	283.9303
Red. masses --	4.6959	2.4917	6.0313
Frc consts --	0.0276	0.0389	0.2865
IR Inten --	1.8007	6.0430	0.5030
	4	5	6
	A	A	A
Frequencies --	344.9853	355.5900	409.1262
Red. masses --	3.1287	3.4428	3.8941
Frc consts --	0.2194	0.2565	0.3840
IR Inten --	5.4212	9.5471	4.4659
	7	8	9
	A	A	A
Frequencies --	446.6304	512.5693	597.4297
Red. masses --	2.8478	3.1785	3.9914
Frc consts --	0.3347	0.4920	0.8394
IR Inten --	1.5905	5.5779	17.0482
	10	11	12
	A	A	A
Frequencies --	701.7745	785.5815	876.3017
Red. masses --	2.5833	2.5603	2.6163
Frc consts --	0.7496	0.9309	1.1837
IR Inten --	16.5589	0.5145	16.5574
	13	14	15
	A	A	A
Frequencies --	899.0744	922.9000	1010.1820
Red. masses --	1.5510	1.6572	2.9293
Frc consts --	0.7387	0.8317	1.7612
IR Inten --	20.5502	19.0545	51.1932
	16	17	18
	A	A	A
Frequencies --	1047.6774	1074.9943	1115.4710
Red. masses --	1.6754	2.1208	2.4222
Frc consts --	1.0835	1.4440	1.7757
IR Inten --	13.8074	5.4865	16.4683
	19	20	21
	A	A	A
Frequencies --	1120.8303	1147.6140	1179.6027
Red. masses --	2.7063	3.2033	3.4679
Frc consts --	2.0031	2.4857	2.8431
IR Inten --	15.4935	126.1447	77.9647
	22	23	24
	A	A	A
Frequencies --	1222.1510	1263.6077	1319.4427
Red. masses --	1.1744	1.4100	1.4003
Frc consts --	1.0335	1.3264	1.4363
IR Inten --	1.8374	45.5245	14.6248
	25	26	27
	A	A	A
Frequencies --	1332.5429	1338.1599	1364.3245
Red. masses --	1.3372	1.2394	1.2722
Frc consts --	1.3990	1.3076	1.3952
IR Inten --	2.0945	28.3938	4.0961
	28	29	30
	A	A	A
Frequencies --	1380.0184	1421.3437	1444.4990
Red. masses --	1.3398	1.3977	1.7784

Frc consts --	1.5033	1.6637	2.1863
IR Inten --	3.9296	5.4580	11.6305

	31	32	33
	A	A	A
Frequencies --	1465.3468	1501.7427	1514.7697
Red. masses --	1.1089	1.0800	1.1003
Frc consts --	1.4029	1.4350	1.4875
IR Inten --	15.3814	1.5506	8.0424
	34	35	36
	A	A	A
Frequencies --	1773.4416	3002.8787	3012.8374
Red. masses --	9.4693	1.0711	1.0700
Frc consts --	17.5470	5.6903	5.7223
IR Inten --	87.4057	43.8404	36.0801
	37	38	39
	A	A	A
Frequencies --	3057.3493	3062.7041	3118.3222
Red. masses --	1.0694	1.0745	1.1030
Frc consts --	5.8894	5.9385	6.3195
IR Inten --	3.1852	33.5287	20.8167
	40	41	42
	A	A	A
Frequencies --	3132.7958	3146.4730	3493.5041
Red. masses --	1.0953	1.0962	1.0747
Frc consts --	6.3337	6.3945	7.7279
IR Inten --	26.0481	19.0793	1.8461

**5a**	1	2	3
	A	A	A
Frequencies --	109.0214	168.6143	234.6645
Red. masses --	3.7429	3.6214	3.8662
Frc consts --	0.0262	0.0607	0.1254
IR Inten --	0.8168	0.3277	6.0803
	4	5	6
	A	A	A
Frequencies --	297.7924	384.2299	414.8383
Red. masses --	3.1182	2.2622	2.1588
Frc consts --	0.1629	0.1968	0.2189
IR Inten --	2.2949	4.5110	4.4487
	7	8	9
	A	A	A
Frequencies --	461.5955	497.9635	647.0683
Red. masses --	5.0652	2.2896	4.1129
Frc consts --	0.6359	0.3345	1.0146
IR Inten --	5.2773	0.5848	2.7297
	10	11	12
	A	A	A
Frequencies --	728.5857	762.8337	803.9768
Red. masses --	1.8220	1.5759	2.4026
Frc consts --	0.5698	0.5403	0.9150
IR Inten --	25.3551	14.0612	5.8936
	13	14	15
	A	A	A
Frequencies --	876.6488	894.0978	932.7303
Red. masses --	2.7765	2.6029	1.9499
Frc consts --	1.2572	1.2260	0.9995
IR Inten --	9.4146	27.4185	22.5944
	16	17	18
	A	A	A
Frequencies --	966.8468	1011.4759	1022.0257
Red. masses --	1.3543	1.6215	2.9869
Frc consts --	0.7459	0.9774	1.8382
IR Inten --	41.4388	10.5959	1.2832

```
                      19                     20                     21
                       A                      A                      A
 Frequencies --   1050.8963              1083.3141              1113.5639
 Red. masses --      2.3895                 1.8944                 2.2658
 Frc consts  --      1.5548                 1.3099                 1.6554
 IR Inten    --     65.8782                16.2681                25.7992

                      22                     23                     24
                       A                      A                      A
 Frequencies --   1183.2299              1237.3854              1277.3354
 Red. masses --      4.5779                 1.2223                 1.3937
 Frc consts  --      3.7762                 1.1026                 1.3397
 IR Inten    --     85.6182                 3.0237                40.7029

                      25                     26                     27
                       A                      A                      A
 Frequencies --   1313.2463              1328.4850              1350.3641
 Red. masses --      1.6222                 1.3829                 1.5145
 Frc consts  --      1.6484                 1.4380                 1.6271
 IR Inten    --      1.4591                 3.5888                 4.9869

                      28                     29                     30
                       A                      A                      A
 Frequencies --   1368.8423              1370.5887              1401.7002
 Red. masses --      1.3041                 1.4176                 1.4248
 Frc consts  --      1.4397                 1.5690                 1.6493
 IR Inten    --     18.9537                 3.8699                 2.5132

                      31                     32                     33
                       A                      A                      A
 Frequencies --   1427.1165              1459.9967              1477.8969
 Red. masses --      1.3757                 1.1377                 1.1224
 Frc consts  --      1.6508                 1.4288                 1.4445
 IR Inten    --     10.1623                 7.5959                 6.0154

                      34                     35                     36
                       A                      A                      A
 Frequencies --   1479.7124              1512.1504              1753.0715
 Red. masses --      1.1205                 1.0977                 6.0668
 Frc consts  --      1.4455                 1.4788                10.9852
 IR Inten    --     15.8575                 5.5490                12.5635


                      37                     38                     39
                       A                      A                      A
 Frequencies --   2988.9457              2991.4745              3066.8325
 Red. masses --      1.0714                 1.0709                 1.0605
 Frc consts  --      5.6392                 5.6461                 5.8769
 IR Inten    --     29.4154                73.1924                14.4663

                      40                     41                     42
                       A                      A                      A
 Frequencies --   3087.4056              3122.9285              3130.2421
 Red. masses --      1.0873                 1.1024                 1.0947
 Frc consts  --      6.1065                 6.3347                 6.3196
 IR Inten    --     33.1253                22.9536                32.4132

                      43                     44                     45
                       A                      A                      A
 Frequencies --   3135.0211              3147.8794              3235.9538
 Red. masses --      1.0936                 1.0619                 1.1163
 Frc consts  --      6.3326                 6.1994                 6.8872
 IR Inten    --     18.0461                 6.8358                10.9902
```

5e

```
                       1                      2                      3
                       A                      A                      A
 Frequencies --    114.4247               173.1093               282.2243
 Red. masses --      3.6503                 2.4947                 3.9039
 Frc consts  --      0.0282                 0.0440                 0.1832
 IR Inten    --      0.1336                 2.5177                 3.3613

                       4                      5                      6
                       A                      A                      A
 Frequencies --    338.1884               357.4635               397.0520
```

```
Red. masses --      2.9374                 3.5717                 2.8746
Frc consts  --      0.1979                 0.2689                 0.2670
IR Inten    --      7.4341                11.1300                 1.7954



                      7                      8                      9
                      A                      A                      A
Frequencies --    448.3527               484.9805               575.6142
Red. masses --      2.7536                 3.4771                 3.7935
Frc consts  --      0.3261                 0.4819                 0.7405
IR Inten    --      0.3925                 2.2308                 6.3441

                     10                     11                     12
                      A                      A                      A
Frequencies --    681.9401               753.4291               801.8901
Red. masses --      2.3317                 1.1514                 2.2725
Frc consts  --      0.6389                 0.3851                 0.8609
IR Inten    --     23.5889                 0.0457                 0.2879

                     13                     14                     15
                      A                      A                      A
Frequencies --    871.7458               896.9981               961.0027
Red. masses --      2.1615                 2.2932                 1.3400
Frc consts  --      0.9678                 1.0871                 0.7291
IR Inten    --      6.0925                 8.3826                47.9231

                     16                     17                     18
                      A                      A                      A
Frequencies --    990.1043              1020.7472              1051.9359
Red. masses --      2.1295                 2.0789                 1.5930
Frc consts  --      1.2300                 1.2762                 1.0386
IR Inten    --     25.2621                22.0481                 1.5642

                     19                     20                     21
                      A                      A                      A
Frequencies --   1098.2265              1100.4775              1126.8432
Red. masses --      4.1030                 2.7186                 2.5183
Frc consts  --      2.9157                 1.9398                 1.8840
IR Inten    --     37.8250                49.3891                18.6462

                     22                     23                     24
                      A                      A                      A
Frequencies --   1169.8673              1220.3109              1260.9762
Red. masses --      4.7066                 1.2771                 1.4683
Frc consts  --      3.7952                 1.1205                 1.3756
IR Inten    --     80.6696                 1.5658                36.7129

                     25                     26                     27
                      A                      A                      A
Frequencies --   1286.5619              1323.0693              1333.7810
Red. masses --      1.5394                 1.3506                 1.2135
Frc consts  --      1.5013                 1.3930                 1.2719
IR Inten    --      2.5380                 4.2769                 3.9846

                     28                     29                     30
                      A                      A                      A
Frequencies --   1365.1205              1378.7629              1408.3859
Red. masses --      1.3120                 1.4168                 1.4788
Frc consts  --      1.4406                 1.5868                 1.7283
IR Inten    --      1.4377                 5.9795                 3.8097

                     31                     32                     33
                      A                      A                      A
Frequencies --   1425.4204              1451.1513              1474.3882
Red. masses --      1.4200                 1.1683                 1.1321
Frc consts  --      1.6999                 1.4495                 1.4499
IR Inten    --      7.1697                 6.0164                15.2849

                     34                     35                     36
                      A                      A                      A
Frequencies --   1500.9341              1514.1838              1753.2013
Red. masses --      1.0797                 1.1001                 6.3394
Frc consts  --      1.4331                 1.4861                11.4805
IR Inten    --      1.0663                 8.4688                17.8089
```

```
                      37                     38                     39
                       A                      A                      A
 Frequencies --   3000.5847              3010.4253              3051.0465
 Red. masses --      1.0707                 1.0689                 1.0642
 Frc consts  --      5.6795                 5.7076                 5.8370
 IR Inten    --     43.9765                41.0884                13.2785

                      40                     41                     42
                       A                      A                      A
 Frequencies --   3063.9922              3117.4320              3127.3811
 Red. masses --      1.0832                 1.0996                 1.0953
 Frc consts  --      5.9917                 6.2964                 6.3119
 IR Inten    --     35.9957                24.0042                32.9979

                      43                     44                     45
                       A                      A                      A
 Frequencies --   3134.0965              3162.4675              3257.7386
 Red. masses --      1.0972                 1.0615                 1.1162
 Frc consts  --      6.3498                 6.2550                 6.9792
 IR Inten    --     25.0810                 6.4718                 3.9955
```

6a

```
                       1                      2                      3
                       A                      A                      A
 Frequencies --     83.8067               143.0961               200.9247
 Red. masses --      7.2784                 4.8657                 4.3367
 Frc consts  --      0.0301                 0.0587                 0.1032
 IR Inten    --     10.0548                 0.2892                 1.5538

                       4                      5                      6
                       A                      A                      A
 Frequencies --    251.1251               319.5787               389.5705
 Red. masses --      3.0359                 2.6139                 6.0928
 Frc consts  --      0.1128                 0.1573                 0.5448
 IR Inten    --      5.7049                 1.8044                 3.6548

                       7                      8                      9
                       A                      A                      A
 Frequencies --    419.4072               500.0119               616.1115
 Red. masses --      4.3741                 3.0137                 4.1365
 Frc consts  --      0.4533                 0.4439                 0.9251
 IR Inten    --      3.2871                13.5846                11.0245

                      10                     11                     12
                       A                      A                      A
 Frequencies --    670.6127               722.8259               735.6200
 Red. masses --      4.6062                 4.8290                 4.0872
 Frc consts  --      1.2205                 1.4865                 1.3031
 IR Inten    --      6.4431                 1.1585                 1.6968

                      13                     14                     15
                       A                      A                      A
 Frequencies --    776.8414               836.3573               956.7885
 Red. masses --      2.3032                 1.8151                 2.6509
 Frc consts  --      0.8189                 0.7481                 1.4298
 IR Inten    --      4.6751                 4.5149                 1.4180

                      16                     17                     18
                       A                      A                      A
 Frequencies --    980.7752              1006.0292              1009.2999
 Red. masses --      2.1956                 2.2263                 2.0374
 Frc consts  --      1.2444                 1.3276                 1.2228
 IR Inten    --     11.8096                 6.8717                 8.9577

                      19                     20                     21
                       A                      A                      A
 Frequencies --   1081.1430              1151.9242              1165.4700
 Red. masses --      3.0900                 1.7210                 1.1301
 Frc consts  --      2.1280                 1.3455                 0.9044
 IR Inten    --     73.7822                20.9267                 2.0620

                      22                     23                     24
                       A                      A                      A
 Frequencies --   1238.8514              1277.7554              1291.5596
 Red. masses --      1.1785                 1.5310                 1.4802
 Frc consts  --      1.0656                 1.4727                 1.4548
```

IR Inten --	1.9033	5.0913	3.7404

	25	26	27
	A	A	A
Frequencies --	1327.1056	1351.6322	1362.2846
Red. masses --	1.3411	1.4057	1.6069
Frc consts --	1.3917	1.5131	1.7570
IR Inten --	11.9380	11.6995	23.9002
	28	29	30
	A	A	A
Frequencies --	1364.3135	1453.8903	1469.2615
Red. masses --	1.3863	1.0894	1.1045
Frc consts --	1.5203	1.3568	1.4048
IR Inten --	7.1562	9.1931	16.4710
	31	32	33
	A	A	A
Frequencies --	1482.2006	1847.2739	3068.4562
Red. masses --	1.0862	11.3160	1.0643
Frc consts --	1.4059	22.7513	5.9042
IR Inten --	6.6642	219.9228	16.2650
	34	35	36
	A	A	A
Frequencies --	3072.8669	3081.6141	3114.4730
Red. masses --	1.0635	1.0584	1.0873
Frc consts --	5.9166	5.9220	6.2137
IR Inten --	12.8389	5.6798	17.8030

	37	38	39
	A	A	A
Frequencies --	3139.6284	3143.0010	3147.3311
Red. masses --	1.1011	1.1017	1.1070
Frc consts --	6.3947	6.4124	6.4607
IR Inten --	5.0947	3.5611	5.5039

6e

	1	2	3
	A	A	A
Frequencies --	84.2371	155.2594	253.4167
Red. masses --	6.9518	2.9427	2.6223
Frc consts --	0.0291	0.0418	0.0992
IR Inten --	5.4358	5.4964	1.7428
	4	5	6
	A	A	A
Frequencies --	285.6834	340.7629	346.4741
Red. masses --	6.8765	4.5314	4.8440
Frc consts --	0.3307	0.3100	0.3426
IR Inten --	0.7054	3.5718	4.5856
	7	8	9
	A	A	A
Frequencies --	367.5972	467.4034	590.5588
Red. masses --	6.2049	2.6778	4.9592
Frc consts --	0.4940	0.3447	1.0190
IR Inten --	3.1809	0.6979	15.8255
	10	11	12
	A	A	A
Frequencies --	690.2523	700.9470	760.7463
Red. masses --	3.7343	3.4985	2.4582
Frc consts --	1.0483	1.0128	0.8382
IR Inten --	2.9084	3.5569	0.5388
	13	14	15
	A	A	A
Frequencies --	788.7417	850.5687	922.8525
Red. masses --	4.3110	1.9903	1.5779

```
 Frc consts  --      1.5801                 0.8484                 0.7917
 IR Inten    --      6.0880                20.7589                 7.0779



                     16                     17                     18
                      A                      A                      A
 Frequencies --    997.5859              1042.7263              1069.0322
 Red. masses --      2.3189                 2.2991                 3.0877
 Frc consts  --      1.3596                 1.4728                 2.0791
 IR Inten    --     11.1621                 6.9900                13.0377

                     19                     20                     21
                      A                      A                      A
 Frequencies --   1100.0502              1136.2563              1146.3129
 Red. masses --      2.2125                 1.4955                 2.0172
 Frc consts  --      1.5775                 1.1376                 1.5617
 IR Inten    --     77.4791                47.8140                 4.3073

                     22                     23                     24
                      A                      A                      A
 Frequencies --   1218.0184              1262.6481              1284.8627
 Red. masses --      1.2930                 1.3377                 1.2997
 Frc consts  --      1.1302                 1.2566                 1.2641
 IR Inten    --      2.5190                 3.3931                 3.4648

                     25                     26                     27
                      A                      A                      A
 Frequencies --   1328.1195              1351.7481              1359.6067
 Red. masses --      1.2322                 1.4407                 1.4192
 Frc consts  --      1.2806                 1.5510                 1.5456
 IR Inten    --     11.3299                 9.5943                 2.2909

                     28                     29                     30
                      A                      A                      A
 Frequencies --   1398.2369              1465.4239              1481.1666
 Red. masses --      1.5445                 1.0906                 1.0931
 Frc consts  --      1.7791                 1.3798                 1.4129
 IR Inten    --      2.1274                13.3639                 5.0304

                     31                     32                     33
                      A                      A                      A
 Frequencies --   1489.6624              1858.9758              3062.4899
 Red. masses --      1.0897                11.9656                 1.0701
 Frc consts  --      1.4247                24.3630                 5.9130
 IR Inten    --      8.8034               228.0358                 2.1207

                     34                     35                     36
                      A                      A                      A
 Frequencies --   3066.8882              3071.0412              3079.1867
 Red. masses --      1.0723                 1.0631                 1.0632
 Frc consts  --      5.9423                 5.9076                 5.9394
 IR Inten    --      4.3218                19.8860                17.4308

                     37                     38                     39
                      A                      A                      A
 Frequencies --   3129.1073              3136.1378              3147.4427
 Red. masses --      1.1014                 1.1054                 1.1045
 Frc consts  --      6.3539                 6.4055                 6.4463
 IR Inten    --      8.7263                 6.1247                 2.8282
```

7a

```
                      1                      2                      3
                      A                      A                      A
 Frequencies --     72.2179               145.8271               200.3857
 Red. masses --      8.1451                 5.1793                 4.1296
 Frc consts  --      0.0250                 0.0649                 0.0977
 IR Inten    --      3.6724                 0.2514                 1.4879

                      4                      5                      6
                      A                      A                      A
 Frequencies --    248.0833               320.8275               349.0473
 Red. masses --      4.2476                 2.7243                 7.3232
 Frc consts  --      0.1540                 0.1652                 0.5257
```

```
 IR Inten    --      6.0415                 2.6661                 0.3076

                      7                      8                      9
                      A                      A                      A
 Frequencies --    387.3601               439.8468               593.4913
 Red. masses --      4.2191                 2.6107                 5.4990
 Frc consts  --      0.3730                 0.2976                 1.1412
 IR Inten    --      2.9198                 0.6468                 5.4565

                     10                     11                     12
                      A                      A                      A
 Frequencies --    670.3074               684.8700               712.6719
 Red. masses --      4.3734                 5.0202                 4.0045
 Frc consts  --      1.1577                 1.3874                 1.1983
 IR Inten    --      9.5604                 5.1735                 1.2848

                     13                     14                     15
                      A                      A                      A
 Frequencies --    767.9373               845.1339               941.8186
 Red. masses --      2.4395                 1.8024                 2.7717
 Frc consts  --      0.8476                 0.7585                 1.4485
 IR Inten    --      3.1348                 5.3448                 0.9725

                     16                     17                     18
                      A                      A                      A
 Frequencies --    964.3310               989.1551              1012.6535
 Red. masses --      2.2022                 2.1791                 2.0882
 Frc consts  --      1.2066                 1.2562                 1.2617
 IR Inten    --      5.8750                11.6611                14.0562

                     19                     20                     21
                      A                      A                      A
 Frequencies --   1063.4405              1136.1614              1150.9846
 Red. masses --      3.7097                 1.5249                 1.4028
 Frc consts  --      2.4718                 1.1598                 1.0949
 IR Inten    --    108.9551                 2.1263                12.1574

                     22                     23                     24
                      A                      A                      A
 Frequencies --   1222.2126              1254.8301              1267.9594
 Red. masses --      1.4346                 1.6057                 1.5353
 Frc consts  --      1.2626                 1.4897                 1.4543
 IR Inten    --     17.7708                52.3446                 7.7978

                     25                     26                     27
                      A                      A                      A
 Frequencies --   1300.6157              1319.3374              1341.4760
 Red. masses --      1.5142                 1.2648                 1.5898
 Frc consts  --      1.5091                 1.2972                 1.6856
 IR Inten    --     35.6119                10.8779                 6.7160

                     28                     29                     30
                      A                      A                      A
 Frequencies --   1355.6574              1408.9233              1458.2927
 Red. masses --      1.2164                 1.8019                 1.0858
 Frc consts  --      1.3171                 2.1074                 1.3605
 IR Inten    --      6.1338                22.6522                 8.9852

                     31                     32                     33
                      A                      A                      A
 Frequencies --   1477.8999              1486.7620              3067.8250
 Red. masses --      1.0794                 1.1325                 1.0640
 Frc consts  --      1.3890                 1.4749                 5.9001
 IR Inten    --     12.5947                 9.2573                12.8884

                     34                     35                     36
                      A                      A                      A
 Frequencies --   3073.4149              3074.5340              3118.1027
 Red. masses --      1.0624                 1.0598                 1.0876
 Frc consts  --      5.9126                 5.9022                 6.2304
 IR Inten    --     14.0387                 3.5738                10.0012
```

	37	38	39
	A	A	A
Frequencies --	3137.9603	3142.5128	3145.3839
Red. masses --	1.1012	1.1060	1.1023
Frc consts --	6.3888	6.4353	6.4254
IR Inten --	5.9326	4.5249	2.3355

7e

	1	2	3
	A	A	A
Frequencies --	70.7073	157.3519	239.5141
Red. masses --	7.5651	3.1118	8.1170
Frc consts --	0.0223	0.0454	0.2744
IR Inten --	1.3865	3.5297	0.7651
	4	5	6
	A	A	A
Frequencies --	244.2897	325.4755	344.6357
Red. masses --	3.4392	4.4899	4.0965
Frc consts --	0.1209	0.2802	0.2867
IR Inten --	3.5812	0.1834	5.1757
	7	8	9
	A	A	A
Frequencies --	371.6184	438.2728	555.6053
Red. masses --	5.9816	2.7058	4.9989
Frc consts --	0.4867	0.3062	0.9092
IR Inten --	1.6885	0.4675	6.3804
	10	11	12
	A	A	A
Frequencies --	628.8173	692.3597	731.2761
Red. masses --	4.2683	3.8903	2.8235
Frc consts --	0.9944	1.0987	0.8896
IR Inten --	8.3176	3.4692	1.3777
	13	14	15
	A	A	A
Frequencies --	790.1652	832.4577	940.0558
Red. masses --	4.4349	1.7291	1.6122
Frc consts --	1.6314	0.7060	0.8394
IR Inten --	7.6695	7.7686	6.3535
	16	17	18
	A	A	A
Frequencies --	983.7792	1006.2131	1049.5967
Red. masses --	2.5680	2.2245	2.8256
Frc consts --	1.4643	1.3270	1.8340
IR Inten --	4.1758	23.9671	3.4955
	19	20	21
	A	A	A
Frequencies --	1095.7118	1131.7002	1139.7453
Red. masses --	2.9102	1.3890	1.9323
Frc consts --	2.0586	1.0481	1.4789
IR Inten --	62.3087	39.4004	0.9023
	22	23	24
	A	A	A
Frequencies --	1189.1921	1236.7708	1270.9788
Red. masses --	1.4723	1.5424	1.2575
Frc consts --	1.2268	1.3901	1.1968
IR Inten --	3.8470	21.7276	2.3602
	25	26	27
	A	A	A
Frequencies --	1306.5719	1325.7574	1333.7910
Red. masses --	1.5000	1.5526	1.2815
Frc consts --	1.5087	1.6079	1.3432
IR Inten --	48.3626	10.2682	0.8811
	28	29	30
	A	A	A
Frequencies --	1380.1477	1387.6820	1476.8623
Red. masses --	1.5745	1.7153	1.0846
Frc consts --	1.7671	1.9461	1.3938

```
 IR Inten    --     16.6466                31.5995                 2.0614



                     31                     32                     33
                      A                      A                      A
 Frequencies --   1483.4826              1487.6246              3056.8412
 Red. masses --      1.0960                 1.0992                 1.0664
 Frc consts  --      1.4212                 1.4333                 5.8712
 IR Inten    --     20.9376                 5.2781                 2.5418

                     34                     35                     36
                      A                      A                      A
 Frequencies --   3066.0722              3077.7545              3084.9388
 Red. masses --      1.0797                 1.0621                 1.0613
 Frc consts  --      5.9805                 5.9277                 5.9506
 IR Inten    --     10.0798                15.4312                13.5305

                     37                     38                     39
                      A                      A                      A
 Frequencies --   3131.0061              3145.2679              3153.6422
 Red. masses --      1.1004                 1.1044                 1.1052
 Frc consts  --      6.3556                 6.4369                 6.4762
 IR Inten    --      6.5316                 5.5012                 2.4411
```

8a

```
                      1                      2                      3
                      A                      A                      A
 Frequencies --     86.5724               143.5165               205.4713
 Red. masses --      5.0121                 4.8979                 4.2619
 Frc consts  --      0.0221                 0.0594                 0.1060
 IR Inten    --      4.5569                 0.1265                 1.3736

                      4                      5                      6
                      A                      A                      A
 Frequencies --    256.8985               323.1002               387.9268
 Red. masses --      2.8117                 2.6492                 5.2273
 Frc consts  --      0.1093                 0.1629                 0.4635
 IR Inten    --      5.0645                 1.8333                 2.2150

                      7                      8                      9
                      A                      A                      A
 Frequencies --    414.8112               487.9826               624.8180
 Red. masses --      3.7271                 2.5160                 4.2374
 Frc consts  --      0.3779                 0.3530                 0.9747
 IR Inten    --     10.5451                13.2935                 5.0351

                     10                     11                     12
                      A                      A                      A
 Frequencies --    670.9240               713.3390               724.0267
 Red. masses --      4.7057                 3.3804                 5.1397
 Frc consts  --      1.2480                 1.0135                 1.5874
 IR Inten    --      6.1505                 7.1849                 3.3784

                     13                     14                     15
                      A                      A                      A
 Frequencies --    782.0098               836.9374               934.0137
 Red. masses --      2.3509                 1.7916                 1.4601
 Frc consts  --      0.8470                 0.7394                 0.7505
 IR Inten    --      3.7718                 0.9880                68.2678

                     16                     17                     18
                      A                      A                      A
 Frequencies --    957.1838               981.3958              1023.0024
 Red. masses --      2.6463                 2.0410                 1.8912
 Frc consts  --      1.4285                 1.1582                 1.1661
 IR Inten    --     10.4814                 9.2519                 4.0422

                     19                     20                     21
                      A                      A                      A
 Frequencies --   1025.7925              1076.2626              1131.1746
 Red. masses --      1.7737                 2.8569                 1.7961
 Frc consts  --      1.0996                 1.9498                 1.3541
 IR Inten    --     12.5608                73.6721                41.3513
```

	22	23	24
	A	A	A
Frequencies --	1184.6344	1212.7614	1247.5048
Red. masses --	1.1236	1.1882	1.1873
Frc consts --	0.9290	1.0296	1.0886
IR Inten --	5.7487	24.4288	2.9778

	25	26	27
	A	A	A
Frequencies --	1294.7990	1326.7163	1346.4630
Red. masses --	1.5043	1.3083	1.3307
Frc consts --	1.4859	1.3568	1.4215
IR Inten --	8.2928	10.6263	26.2698

	28	29	30
	A	A	A
Frequencies --	1353.6849	1371.6789	1414.6140
Red. masses --	1.2948	1.3922	2.0309
Frc consts --	1.3979	1.5434	2.3945
IR Inten --	6.6094	12.2086	18.1265

	31	32	33
	A	A	A
Frequencies --	1452.9983	1469.5737	1478.2347
Red. masses --	1.0963	1.1142	1.0992
Frc consts --	1.3637	1.4178	1.4152
IR Inten --	8.9641	17.4277	6.7258

	34	35	36
	A	A	A
Frequencies --	1760.8180	3060.8570	3069.1350
Red. masses --	9.1638	1.0754	1.0667
Frc consts --	16.7399	5.9361	5.9202
IR Inten --	72.4364	6.3080	16.7380

	37	38	39
	A	A	A
Frequencies --	3074.0502	3077.8489	3137.9335
Red. masses --	1.0725	1.0597	1.1008
Frc consts --	5.9711	5.9147	6.3864
IR Inten --	44.9992	6.1727	7.1721

	40	41	42
	A	A	A
Frequencies --	3140.8551	3142.6717	3475.7921
Red. masses --	1.1016	1.1050	1.0745
Frc consts --	6.4027	6.4301	7.6481
IR Inten --	5.9668	4.7986	1.4661

8e

	1	2	3
	A	A	A
Frequencies --	101.4355	162.4574	256.2731
Red. masses --	5.0399	2.9914	2.5458
Frc consts --	0.0306	0.0465	0.0985
IR Inten --	1.6711	3.8908	3.0378

	4	5	6
	A	A	A
Frequencies --	300.5078	346.4593	349.5318
Red. masses --	4.6675	4.6138	4.7814
Frc consts --	0.2483	0.3263	0.3442
IR Inten --	3.4040	4.4463	7.7486

	7	8	9
	A	A	A
Frequencies --	369.7103	463.5511	579.5649
Red. masses --	5.9136	2.4833	4.3056
Frc consts --	0.4762	0.3144	0.8521
IR Inten --	0.6341	2.7955	18.5691

```
                     10                     11                     12
                      A                      A                      A
Frequencies --    682.7011               690.0668               771.5966
Red. masses --      3.4060                 3.5616                 2.6415
Frc consts  --      0.9353                 0.9993                 0.9266
IR Inten    --      8.3310                 2.4041                 2.5384

                     13                     14                     15
                      A                      A                      A
Frequencies --    788.7274               849.0172               910.6160
Red. masses --      3.9815                 1.8078                 1.4386
Frc consts  --      1.4593                 0.7678                 0.7029
IR Inten    --      5.4765                23.4817                34.5774

                     16                     17                     18
                      A                      A                      A
Frequencies --    979.4528              1013.2434              1039.5176
Red. masses --      1.9158                 1.4577                 2.0409
Frc consts  --      1.0828                 0.8818                 1.2994
IR Inten    --     14.9969                30.7562                 7.2201

                     19                     20                     21
                      A                      A                      A
Frequencies --   1056.3931              1086.2399              1123.0909
Red. masses --      3.2723                 2.6298                 2.1040
Frc consts  --      2.1516                 1.8282                 1.5636
IR Inten    --     49.6956                47.2758                13.5130

                     22                     23                     24
                      A                      A                      A
Frequencies --   1168.6582              1214.2614              1237.2809
Red. masses --      1.2442                 1.2221                 1.2721
Frc consts  --      1.0012                 1.0616                 1.1474
IR Inten    --      9.7621                 2.7866                18.3638

                     25                     26                     27
                      A                      A                      A
Frequencies --   1277.3657              1328.1481              1331.3222
Red. masses --      1.2508                 1.2322                 1.3193
Frc consts  --      1.2025                 1.2806                 1.3778
IR Inten    --      1.4662                 1.6798                39.9518

                     28                     29                     30
                      A                      A                      A
Frequencies --   1368.8551              1371.3240              1423.6263
Red. masses --      1.2850                 1.3943                 1.9508
Frc consts  --      1.4187                 1.5448                 2.3294
IR Inten    --      4.8500                 5.3316                24.0684

                     31                     32                     33
                      A                      A                      A
Frequencies --   1471.3438              1480.9061              1487.5106
Red. masses --      1.0959                 1.1131                 1.0933
Frc consts  --      1.3979                 1.4383                 1.4253
IR Inten    --     11.3317                 7.5327                 9.5130

                     34                     35                     36
                      A                      A                      A
Frequencies --   1767.9645              3060.7065              3070.5055
Red. masses --      9.9038                 1.0632                 1.0619
Frc consts  --     18.2389                 5.8684                 5.8989
IR Inten    --     85.4710                 7.9606                13.7437

                     37                     38                     39
                      A                      A                      A
Frequencies --   3075.6877              3084.4088              3125.8378
Red. masses --      1.0670                 1.0778                 1.1007
Frc consts  --      5.9470                 6.0414                 6.3364
IR Inten    --      6.7502                19.8817                 8.7203

                     40                     41                     42
```

```
                      A                      A                      A
 Frequencies --   3135.3999              3148.0355              3493.1624
 Red. masses --      1.1055                 1.1048                 1.0734
 Frc consts  --      6.4031                 6.4507                 7.7168
 IR Inten    --      7.6529                 4.1553                 7.0834
```

9a

```
                      1                      2                      3
                      A                      A                      A
 Frequencies --     93.3564               146.4966               206.5622
 Red. masses --      5.1341                 5.0284                 4.0463
 Frc consts  --      0.0264                 0.0636                 0.1017
 IR Inten    --      5.1398                 0.6194                 1.8041

                      4                      5                      6
                      A                      A                      A
 Frequencies --    258.5727               324.6502               391.0285
 Red. masses --      2.7488                 2.6441                 5.2052
 Frc consts  --      0.1083                 0.1642                 0.4689
 IR Inten    --      5.7699                 2.2404                 2.9512

                      7                      8                      9
                      A                      A                      A
 Frequencies --    416.4293               490.4948               629.7740
 Red. masses --      4.3083                 2.3596                 4.1879
 Frc consts  --      0.4402                 0.3345                 0.9786
 IR Inten    --      3.3695                12.1097                 4.7718

                     10                     11                     12
                      A                      A                      A
 Frequencies --    671.7302               718.0716               727.0941
 Red. masses --      4.6056                 4.0300                 4.7665
 Frc consts  --      1.2244                 1.2243                 1.4847
 IR Inten    --      7.5069                 4.5002                 3.3674

                     13                     14                     15
                      A                      A                      A
 Frequencies --    777.8342               838.4363               925.1058
 Red. masses --      2.3878                 1.7553                 1.3589
 Frc consts  --      0.8512                 0.7270                 0.6852
 IR Inten    --      2.6433                10.3360                20.7546

                     16                     17                     18
                      A                      A                      A
 Frequencies --    955.0686               976.9658              1022.1750
 Red. masses --      2.7507                 1.9944                 2.0983
 Frc consts  --      1.4783                 1.1216                 1.2917
 IR Inten    --      0.1812                26.9721                15.5383

                     19                     20                     21
                      A                      A                      A
 Frequencies --   1027.6970              1074.9338              1130.5753
 Red. masses --      1.7344                 2.7984                 1.7642
 Frc consts  --      1.0793                 1.9051                 1.3286
 IR Inten    --     17.5447                79.4288                31.1783

                     22                     23                     24
                      A                      A                      A
 Frequencies --   1182.2007              1206.7887              1243.4090
 Red. masses --      1.1315                 1.1855                 1.1741
 Frc consts  --      0.9318                 1.0172                 1.0695
 IR Inten    --      5.5700                18.9602                 2.4338

                     25                     26                     27
                      A                      A                      A
 Frequencies --   1299.4665              1327.4592              1340.1995
 Red. masses --      1.5160                 1.3312                 1.3096
 Frc consts  --      1.5083                 1.3820                 1.3859
 IR Inten    --     10.3770                10.9799                 1.0283

                     28                     29                     30
                      A                      A                      A
 Frequencies --   1360.5791              1367.5042              1418.8187
 Red. masses --      1.3124                 1.3049                 2.3389
 Frc consts  --      1.4314                 1.4377                 2.7741
 IR Inten    --      9.2401                15.8150                60.0263
```

	31	32	33
	A	A	A
Frequencies --	1454.0450	1473.7595	1479.1981
Red. masses --	1.1032	1.0905	1.0986
Frc consts --	1.3743	1.3955	1.4163
IR Inten --	10.5760	16.8447	6.3108
	34	35	36
	A	A	A
Frequencies --	1764.1371	3061.8964	3064.9962
Red. masses --	9.1476	1.0605	1.0649
Frc consts --	16.7734	5.8581	5.8940
IR Inten --	75.0056	16.6562	23.2694
	37	38	39
	A	A	A
Frequencies --	3074.0698	3115.4567	3119.5261
Red. masses --	1.0633	1.0877	1.1021
Frc consts --	5.9201	6.2203	6.3193
IR Inten --	11.7185	15.5314	9.3818
	40	41	42
	A	A	A
Frequencies --	3138.3864	3145.3206	3486.3499
Red. masses --	1.1023	1.1018	1.0745
Frc consts --	6.3969	6.4221	7.6945
IR Inten --	7.6558	3.5174	1.2771

9e

	1	2	3
	A	A	A
Frequencies --	93.5682	162.9627	254.9896
Red. masses --	5.4759	3.0263	2.5510
Frc consts --	0.0282	0.0474	0.0977
IR Inten --	2.4883	5.8346	2.2157
	4	5	6
	A	A	A
Frequencies --	298.4039	344.8053	348.2700
Red. masses --	5.5779	3.5040	5.5835
Frc consts --	0.2926	0.2455	0.3990
IR Inten --	0.1897	2.1917	1.2086
	7	8	9
	A	A	A
Frequencies --	369.2596	471.6816	577.3881
Red. masses --	6.1980	2.4744	3.9966
Frc consts --	0.4979	0.3244	0.7850
IR Inten --	4.1948	2.6880	19.3021
	10	11	12
	A	A	A
Frequencies --	686.2648	692.2537	778.1838
Red. masses --	3.2306	3.7919	2.8275
Frc consts --	0.8964	1.0706	1.0088
IR Inten --	5.9617	0.5431	3.6892
	13	14	15
	A	A	A
Frequencies --	789.2063	850.4512	894.4982
Red. masses --	3.5014	1.8434	1.4003
Frc consts --	1.2849	0.7856	0.6601
IR Inten --	5.2902	10.0843	25.5937
	16	17	18
	A	A	A
Frequencies --	972.3429	1005.4802	1037.6727
Red. masses --	1.6583	1.7188	2.1621
Frc consts --	0.9237	1.0238	1.3717
IR Inten --	24.9756	21.0804	15.4868

```
                     19                     20                     21
                      A                      A                      A
 Frequencies --   1064.2328              1100.5371              1119.8522
 Red. masses --      2.5845                 2.6948                 2.6523
 Frc consts  --      1.7247                 1.9231                 1.9597
 IR Inten    --      8.3269                51.1079                76.8904

                     22                     23                     24
                      A                      A                      A
 Frequencies --   1166.3182              1204.5026              1228.5496
 Red. masses --      1.2229                 1.1595                 1.2586
 Frc consts  --      0.9801                 0.9912                 1.1192
 IR Inten    --     17.7623                18.6886                 1.3788


                     25                     26                     27
                      A                      A                      A
 Frequencies --   1281.5139              1328.7918              1341.6628
 Red. masses --      1.2602                 1.3113                 1.2739
 Frc consts  --      1.2193                 1.3642                 1.3511
 IR Inten    --      2.7289                 7.9665                29.1897

                     28                     29                     30
                      A                      A                      A
 Frequencies --   1366.0830              1368.6132              1433.0389
 Red. masses --      1.3643                 1.3639                 1.8193
 Frc consts  --      1.5000                 1.5052                 2.2012
 IR Inten    --     11.9926                 1.5052                13.5819

                     31                     32                     33
                      A                      A                      A
 Frequencies --   1480.1251              1481.7701              1487.7978
 Red. masses --      1.1036                 1.1047                 1.0934
 Frc consts  --      1.4244                 1.4291                 1.4260
 IR Inten    --      5.7067                16.7918                 6.9288

                     34                     35                     36
                      A                      A                      A
 Frequencies --   1768.9823              3051.7070              3062.6695
 Red. masses --      9.4053                 1.0605                 1.0823
 Frc consts  --     17.3409                 5.8192                 5.9811
 IR Inten    --     80.5693                15.3960                10.1203

                     37                     38                     39
                      A                      A                      A
 Frequencies --   3066.8446              3080.9270              3104.3149
 Red. masses --      1.0634                 1.0622                 1.1028
 Frc consts  --      5.8930                 5.9403                 6.2614
 IR Inten    --     15.3424                16.3789                19.0345

                     40                     41                     42
                      A                      A                      A
 Frequencies --   3132.6692              3150.4981              3492.4473
 Red. masses --      1.1046                 1.1046                 1.0747
 Frc consts  --      6.3868                 6.4595                 7.7233
 IR Inten    --      7.9219                 3.7443                 1.4817
```

10a

```
                      1                      2                      3
                      A                      A                      A
 Frequencies --     97.2725               145.6532               207.1900
 Red. masses --      3.8294                 4.9983                 3.7497
 Frc consts  --      0.0213                 0.0625                 0.0948
 IR Inten    --      0.8086                 0.3874                 3.4645

                      4                      5                      6
                      A                      A                      A
 Frequencies --    261.0765               325.5594               385.0631
 Red. masses --      2.5707                 2.6872                 3.5707
 Frc consts  --      0.1032                 0.1678                 0.3119
 IR Inten    --      2.9279                 2.6117                 1.6893
```

```
                      7                      8                      9
                      A                      A                      A
 Frequencies --    389.6017               465.8533               626.2455
 Red. masses --      3.3100                 2.2766                 4.1752
 Frc consts  --      0.2960                 0.2911                 0.9648
 IR Inten    --      1.1655                 3.0511                 1.5277

                     10                     11                     12
                      A                      A                      A
 Frequencies --    669.9270               690.7391               723.1214
 Red. masses --      4.5188                 2.8559                 4.7100
 Frc consts  --      1.1949                 0.8028                 1.4511
 IR Inten    --      9.6899                10.3235                 3.4693

                     13                     14                     15
                      A                      A                      A
 Frequencies --    747.7475               788.9085               847.0296
 Red. masses --      1.2631                 2.0655                 1.8939
 Frc consts  --      0.4161                 0.7574                 0.8006
 IR Inten    --      1.8995                 0.9798                 7.6642

                     16                     17                     18
                      A                      A                      A
 Frequencies --    936.4222               957.2388               963.0057
 Red. masses --      2.0621                 2.0068                 1.4172
 Frc consts  --      1.0654                 1.0834                 0.7743
 IR Inten    --      1.2811                11.7422                42.2799

                     19                     20                     21
                      A                      A                      A
 Frequencies --   1002.3842              1028.2128              1039.1630
 Red. masses --      1.7968                 2.2355                 1.9513
 Frc consts  --      1.0637                 1.3925                 1.2415
 IR Inten    --     14.0081                22.2164                27.9969

                     22                     23                     24
                      A                      A                      A
 Frequencies --   1068.8620              1185.4999              1198.2138
 Red. masses --      2.5915                 1.1467                 1.3101
 Frc consts  --      1.7444                 0.9495                 1.1082
 IR Inten    --     50.8622                 8.6612                 4.6097

                     25                     26                     27
                      A                      A                      A
 Frequencies --   1247.9673              1298.4826              1324.1560
 Red. masses --      1.1752                 1.5105                 1.4276
 Frc consts  --      1.0784                 1.5006                 1.4748
 IR Inten    --      3.9590                10.7916                11.2653

                     28                     29                     30
                      A                      A                      A
 Frequencies --   1334.2957              1355.5036              1363.5177
 Red. masses --      1.4465                 1.2883                 1.2523
 Frc consts  --      1.5173                 1.3947                 1.3717
 IR Inten    --      2.9076                13.0537                 9.1247

                     31                     32                     33
                      A                      A                      A
 Frequencies --   1379.6000              1452.8600              1462.1024
 Red. masses --      2.1195                 1.0949                 1.1463
 Frc consts  --      2.3768                 1.3617                 1.4437
 IR Inten    --      5.3807                 8.2912                12.0172

                     34                     35                     36
                      A                      A                      A
 Frequencies --   1473.1543              1483.7537              1747.1392
 Red. masses --      1.0937                 1.1285                 5.9012
 Frc consts  --      1.3984                 1.4638                10.6132
 IR Inten    --     10.0935                13.9783                11.2664

                     37                     38                     39
                      A                      A                      A
```

Frequencies --	3061.8545	3063.9299	3067.9146
Red. masses --	1.0599	1.0646	1.0689
Frc consts --	5.8543	5.8885	5.9276
IR Inten --	21.7112	20.1131	1.6940
	40	41	42
	A	A	A
Frequencies --	3081.0465	3112.5628	3136.1418
Red. masses --	1.0800	1.1032	1.1027
Frc consts --	6.0403	6.2968	6.3900
IR Inten --	44.5926	16.5719	11.1285
	43	44	45
	A	A	A
Frequencies --	3142.2068	3151.1834	3238.1856
Red. masses --	1.1017	1.0620	1.1163
Frc consts --	6.4090	6.2130	6.8964
IR Inten --	5.2659	7.0990	10.2566

10e

	1	2	3
	A	A	A
Frequencies --	103.5849	169.5804	255.7268
Red. masses --	3.9299	3.0261	2.4713
Frc consts --	0.0248	0.0513	0.0952
IR Inten --	0.0475	3.2060	5.4312
	4	5	6
	A	A	A
Frequencies --	301.5113	340.4264	349.4285
Red. masses --	3.7392	4.4167	3.8981
Frc consts --	0.2003	0.3016	0.2804
IR Inten --	1.4277	0.7943	3.1281
	7	8	9
	A	A	A
Frequencies --	371.6677	448.2121	545.4585
Red. masses --	5.6909	2.1932	3.6757
Frc consts --	0.4632	0.2596	0.6443
IR Inten --	1.4502	1.1945	7.6236
	10	11	12
	A	A	A
Frequencies --	655.8671	687.1146	752.5516
Red. masses --	2.8684	3.9116	1.1799
Frc consts --	0.7270	1.0881	0.3937
IR Inten --	12.7969	2.4581	0.5830
	13	14	15
	A	A	A
Frequencies --	785.9402	792.5091	844.5703
Red. masses --	3.5256	2.2195	1.8607
Frc consts --	1.2831	0.8213	0.7820
IR Inten --	7.9800	0.9134	8.7465
	16	17	18
	A	A	A
Frequencies --	949.2102	965.2485	968.2675
Red. masses --	1.7172	1.3620	1.6245
Frc consts --	0.9116	0.7477	0.8973
IR Inten --	12.5454	46.8550	4.8220
	19	20	21
	A	A	A
Frequencies --	1021.8262	1042.0752	1068.2976
Red. masses --	2.0171	2.0735	5.1316
Frc consts --	1.2409	1.3266	3.4506
IR Inten --	9.6605	5.0228	86.7305
	22	23	24
	A	A	A
Frequencies --	1090.4602	1168.4838	1193.9531
Red. masses --	2.4030	1.1753	1.4028
Frc consts --	1.6835	0.9455	1.1782
IR Inten --	12.1780	7.1760	0.8871

```
                      25                     26                     27
                       A                      A                      A
 Frequencies --   1223.6744              1277.8506              1297.9106
 Red. masses --      1.2733                 1.2690                 1.5277
 Frc consts  --      1.1233                 1.2208                 1.5162
 IR Inten    --      6.4983                 1.3866                 1.3063

                      28                     29                     30
                       A                      A                      A
 Frequencies --   1330.2372              1366.9577              1373.7271
 Red. masses --      1.2491                 1.3880                 1.3256
 Frc consts  --      1.3023                 1.5281                 1.4739
 IR Inten    --     12.4679                 0.6092                 2.3360

                      31                     32                     33
                       A                      A                      A
 Frequencies --   1405.1499              1448.8455              1477.3674
 Red. masses --      1.6679                 1.1692                 1.0979
 Frc consts  --      1.9403                 1.4460                 1.4119
 IR Inten    --      7.0190                 9.8595                 1.0346

                      34                     35                     36
                       A                      A                      A
 Frequencies --   1482.9132              1487.6822              1747.7455
 Red. masses --      1.0973                 1.1034                 6.2127
 Frc consts  --      1.4217                 1.4389                11.1812
 IR Inten    --     20.7886                 6.5089                14.7796

                      37                     38                     39
                       A                      A                      A
 Frequencies --   3048.2933              3065.7829              3066.2989
 Red. masses --      1.0610                 1.0766                 1.0683
 Frc consts  --      5.8089                 5.9618                 5.9180
 IR Inten    --     19.1687                 3.9380                23.0714

                      40                     41                     42
                       A                      A                      A
 Frequencies --   3080.3534              3100.2928              3130.1787
 Red. masses --      1.0622                 1.1014                 1.1053
 Frc consts  --      5.9380                 6.2374                 6.3809
 IR Inten    --     19.7453                24.7154                10.8834

                      43                     44                     45
                       A                      A                      A
 Frequencies --   3149.1202              3163.9358              3259.0272
 Red. masses --      1.1051                 1.0618                 1.1158
 Frc consts  --      6.4573                 6.2627                 6.9827
 IR Inten    --      6.1235                 6.7353                 3.1400
```

11a

```
                       1                      2                      3
                       A                      A                      A
 Frequencies --    101.1767               160.5896               240.0003
 Red. masses --      6.1572                 3.4545                 4.2361
 Frc consts  --      0.0371                 0.0525                 0.1438
 IR Inten    --      7.3175                 1.4224                 7.2050

                       4                      5                      6
                       A                      A                      A
 Frequencies --    294.4307               390.8713               448.9402
 Red. masses --      3.0058                 1.9420                 5.2179
 Frc consts  --      0.1535                 0.1748                 0.6196
 IR Inten    --      5.1155                 3.1594                 0.9337

                       7                      8                      9
                       A                      A                      A
 Frequencies --    488.7561               509.1130               645.3116
 Red. masses --      4.7533                 2.2922                 3.7919
 Frc consts  --      0.6690                 0.3501                 0.9304
 IR Inten    --     15.9616                 4.1295                12.5120
```

	10	11	12
	A	A	A
Frequencies --	734.6312	768.7435	818.1871
Red. masses --	2.1600	2.2971	2.1907
Frc consts --	0.6868	0.7998	0.8640
IR Inten --	24.5046	29.2213	108.8579

	13	14	15
	A	A	A
Frequencies --	861.7334	902.2703	927.2529
Red. masses --	2.3524	2.0509	2.1586
Frc consts --	1.0292	0.9837	1.0935
IR Inten --	13.3246	10.9055	33.0734

	16	17	18
	A	A	A
Frequencies --	1020.0233	1024.6054	1041.9556
Red. masses --	2.0287	2.8893	2.6940
Frc consts --	1.2436	1.7871	1.7232
IR Inten --	9.3755	11.2426	54.6096

	19	20	21
	A	A	A
Frequencies --	1113.2498	1165.8709	1200.0849
Red. masses --	2.2526	2.7364	1.1898
Frc consts --	1.6448	2.1914	1.0096
IR Inten --	22.3706	8.3915	1.3081

	22	23	24
	A	A	A
Frequencies --	1233.5288	1269.9180	1308.6309
Red. masses --	2.1632	1.5032	1.7141
Frc consts --	1.9393	1.4283	1.7295
IR Inten --	42.0672	2.5779	4.1932

	25	26	27
	A	A	A
Frequencies --	1322.2082	1350.5672	1371.9926
Red. masses --	1.3662	1.3213	1.2106
Frc consts --	1.4072	1.4200	1.3427
IR Inten --	13.1265	7.6626	10.3054

	28	29	30
	A	A	A
Frequencies --	1389.4574	1407.9238	1469.5967
Red. masses --	1.5493	1.3863	1.1080
Frc consts --	1.7623	1.6191	1.4099
IR Inten --	10.7065	11.8966	11.9274

	31	32	33
	A	A	A
Frequencies --	1473.8738	1509.7093	1523.1910
Red. masses --	1.0915	1.1010	1.2062
Frc consts --	1.3970	1.4784	1.6488
IR Inten --	11.9471	7.5468	13.3274

	34	35	36
	A	A	A
Frequencies --	1842.7147	3048.3992	3051.8801
Red. masses --	11.2807	1.0674	1.0654
Frc consts --	22.5686	5.8440	5.8467
IR Inten --	241.3275	30.5554	22.3863

	37	38	39
	A	A	A
Frequencies --	3074.3739	3119.7018	3120.9733
Red. masses --	1.0612	1.0993	1.1003
Frc consts --	5.9098	6.3037	6.3146
IR Inten --	5.9076	24.6483	15.0716

	40	41	42
	A	A	A

Frequencies --	3130.4184	3147.0611	3587.0583
Red. masses --	1.0887	1.1041	1.0744
Frc consts --	6.2858	6.4425	8.1454
IR Inten --	20.4604	12.1021	5.0638

11e

	1	2	3
	A	A	A
Frequencies --	96.1583	153.2983	276.1603
Red. masses --	5.4481	2.4283	8.1820
Frc consts --	0.0297	0.0336	0.3676
IR Inten --	2.6288	3.2386	0.8472
	4	5	6
	A	A	A
Frequencies --	330.4282	351.0983	405.2329
Red. masses --	2.0008	3.5104	5.2860
Frc consts --	0.1287	0.2550	0.5114
IR Inten --	10.6106	6.0233	2.6973
	7	8	9
	A	A	A
Frequencies --	436.7069	525.3327	609.6375
Red. masses --	2.9897	2.8646	4.2324
Frc consts --	0.3359	0.4658	0.9268
IR Inten --	0.6637	5.7098	14.3086
	10	11	12
	A	A	A
Frequencies --	697.8262	747.3084	772.9128
Red. masses --	1.8940	1.9030	2.3597
Frc consts --	0.5434	0.6262	0.8305
IR Inten --	18.3979	76.8353	81.1575
	13	14	15
	A	A	A
Frequencies --	878.5272	912.1389	962.7654
Red. masses --	2.3104	2.3703	1.4830
Frc consts --	1.0506	1.1619	0.8099
IR Inten --	22.4525	11.9792	1.2500
	16	17	18
	A	A	A
Frequencies --	1008.8371	1056.5914	1106.1331
Red. masses --	2.6632	3.1957	2.5104
Frc consts --	1.5970	2.1020	1.8097
IR Inten --	18.1497	3.4362	67.1653
	19	20	21
	A	A	A
Frequencies --	1130.1363	1158.8543	1177.9298
Red. masses --	2.6825	2.0342	1.9498
Frc consts --	2.0186	1.6096	1.5940
IR Inten --	41.4706	23.8521	6.7875
	22	23	24
	A	A	A
Frequencies --	1222.9656	1261.7665	1313.8978
Red. masses --	2.4647	1.3543	1.4973
Frc consts --	2.1719	1.2703	1.5229
IR Inten --	34.7423	1.5555	3.3852
	25	26	27
	A	A	A
Frequencies --	1333.4191	1347.8107	1371.9175
Red. masses --	1.2431	1.3386	1.1834
Frc consts --	1.3022	1.4327	1.3123
IR Inten --	12.5306	2.0073	2.4494
	28	29	30
	A	A	A
Frequencies --	1395.2219	1419.7182	1463.1314
Red. masses --	1.4137	1.5853	1.1042
Frc consts --	1.6214	1.8826	1.3928
IR Inten --	13.2714	1.2593	15.3465

	31	32	33
	A	A	A
Frequencies --	1493.9267	1504.8305	1512.5884
Red. masses --	1.0833	1.1019	1.2270
Frc consts --	1.4244	1.4701	1.6540
IR Inten --	6.6783	6.9568	11.4320
	34	35	36
	A	A	A
Frequencies --	1856.8614	3040.6316	3051.5699
Red. masses --	11.9196	1.0809	1.0685
Frc consts --	24.2143	5.8882	5.8625
IR Inten --	245.8805	8.0585	6.8760
	37	38	39
	A	A	A
Frequencies --	3057.9669	3062.5102	3120.0895
Red. masses --	1.0632	1.0653	1.1008
Frc consts --	5.8580	5.8867	6.3137
IR Inten --	29.2929	39.5136	24.2353
	40	41	42
	A	A	A
Frequencies --	3130.4550	3136.4316	3580.1637
Red. masses --	1.1027	1.1001	1.0747
Frc consts --	6.3667	6.3762	8.1160
IR Inten --	17.5453	14.3036	1.6657

12a

	1	2	3
	A	A	A
Frequencies --	89.9109	157.0795	250.9153
Red. masses --	6.1371	3.5560	4.0955
Frc consts --	0.0292	0.0517	0.1519
IR Inten --	2.1174	1.5328	7.2529
	4	5	6
	A	A	A
Frequencies --	268.2100	355.3750	404.6665
Red. masses --	4.0590	2.9776	2.2982
Frc consts --	0.1720	0.2216	0.2217
IR Inten --	4.0087	1.1303	2.7928
	7	8	9
	A	A	A
Frequencies --	425.4811	506.7107	625.5329
Red. masses --	5.0097	2.5069	5.0632
Frc consts --	0.5344	0.3792	1.1673
IR Inten --	2.5325	1.2232	3.6571
	10	11	12
	A	A	A
Frequencies --	700.5194	749.2140	802.9811
Red. masses --	2.7916	1.8626	2.0534
Frc consts --	0.8071	0.6160	0.7801
IR Inten --	7.6393	66.5568	138.1185
	13	14	15
	A	A	A
Frequencies --	858.3642	886.5778	912.3708
Red. masses --	2.4569	2.3578	2.2178
Frc consts --	1.0665	1.0919	1.0877
IR Inten --	8.6828	8.7061	42.9313
	16	17	18
	A	A	A
Frequencies --	1005.8264	1018.6720	1032.6549
Red. masses --	2.2903	2.6773	2.6312
Frc consts --	1.3652	1.6369	1.6531
IR Inten --	14.4283	22.1546	71.9227

	19	20	21
	A	A	A
Frequencies --	1079.1175	1156.9108	1181.6640
Red. masses --	1.9318	2.5784	1.4863
Frc consts --	1.3254	2.0333	1.2227
IR Inten --	48.0066	19.6747	0.3645
	22	23	24
	A	A	A
Frequencies --	1222.4358	1247.8118	1266.8314
Red. masses --	2.1050	2.0550	1.4599
Frc consts --	1.8533	1.8852	1.3804
IR Inten --	11.7405	27.2696	17.3363
	25	26	27
	A	A	A
Frequencies --	1313.0430	1318.5309	1353.1730
Red. masses --	1.4043	1.5469	1.2888
Frc consts --	1.4265	1.5845	1.3904
IR Inten --	12.3993	83.6645	2.8092
	28	29	30
	A	A	A
Frequencies --	1371.6989	1378.2917	1421.5192
Red. masses --	1.3937	1.3341	1.6862
Frc consts --	1.5450	1.4933	2.0075
IR Inten --	7.0740	30.9851	8.3870
	31	32	33
	A	A	A
Frequencies --	1477.4782	1486.4219	1500.8451
Red. masses --	1.0886	1.1477	1.1043
Frc consts --	1.4001	1.4941	1.4656
IR Inten --	7.5087	13.0150	9.1337
	34	35	36
	A	A	A
Frequencies --	1520.5191	3055.6230	3059.5056
Red. masses --	1.2070	1.0664	1.0642
Frc consts --	1.6441	5.8663	5.8691
IR Inten --	10.9099	26.7491	18.4359
	37	38	39
	A	A	A
Frequencies --	3065.6394	3125.6351	3126.4466
Red. masses --	1.0622	1.1018	1.1009
Frc consts --	5.8815	6.3419	6.3401
IR Inten --	10.3936	11.8725	26.0502
	40	41	42
	A	A	A
Frequencies --	3134.2742	3145.4735	3587.1353
Red. masses --	1.0892	1.1023	1.0745
Frc consts --	6.3045	6.4259	8.1465
IR Inten --	13.6630	7.5802	7.0811

12e

	1	2	3
	A	A	A
Frequencies --	82.9310	162.0845	234.0202
Red. masses --	5.3720	2.6000	14.7716
Frc consts --	0.0218	0.0402	0.4766
IR Inten --	0.8452	1.5390	0.8482
	4	5	6
	A	A	A
Frequencies --	315.8577	356.6600	378.0080
Red. masses --	2.3486	3.4902	3.2070
Frc consts --	0.1380	0.2616	0.2700
IR Inten --	7.0449	3.9221	3.1376
	7	8	9
	A	A	A

```
 Frequencies --    437.2959                496.4262                579.8455
 Red. masses --      3.3315                  3.7744                  4.1920
 Frc consts  --      0.3753                  0.5480                  0.8304
 IR Inten    --      0.8442                  5.7002                  7.3566

                     10                      11                      12
                      A                       A                       A
 Frequencies --    657.3817                723.5487                765.6042
 Red. masses --      2.3115                  2.2886                  1.9695
 Frc consts  --      0.5885                  0.7059                  0.6802
 IR Inten    --      3.1970                 57.7449                146.2833

                     13                      14                      15
                      A                       A                       A
 Frequencies --    848.7270                902.7876                980.8773
 Red. masses --      1.8421                  2.4780                  1.5471
 Frc consts  --      0.7818                  1.1899                  0.8770
 IR Inten    --     11.0477                  4.9959                  1.1053

                     16                      17                      18
                      A                       A                       A
 Frequencies --    998.6195               1028.2587               1089.2228
 Red. masses --      2.7837                  3.1928                  1.9758
 Frc consts  --      1.6356                  1.9890                  1.3811
 IR Inten    --     36.9188                  4.6526                 16.1018

                     19                      20                      21
                      A                       A                       A
 Frequencies --   1112.3706               1151.2553               1170.3478
 Red. masses --      3.2407                  1.8823                  1.9079
 Frc consts  --      2.3626                  1.4699                  1.5397
 IR Inten    --     64.6322                 32.7039                 16.1583

                     22                      23                      24
                      A                       A                       A
 Frequencies --   1209.6892               1232.7866               1293.9365
 Red. masses --      2.4331                  2.0036                  1.4487
 Frc consts  --      2.0978                  1.7941                  1.4291
 IR Inten    --      7.1407                 22.0854                  1.0835

                     25                      26                      27
                      A                       A                       A
 Frequencies --   1318.4107               1333.3274               1348.7071
 Red. masses --      1.2816                  1.6112                  1.5722
 Frc consts  --      1.3125                  1.6876                  1.6850
 IR Inten    --      8.0068                 66.8634                 57.2981

                     28                      29                      30
                      A                       A                       A
 Frequencies --   1360.2035               1397.0953               1413.2685
 Red. masses --      1.1850                  1.5265                  1.5841
 Frc consts  --      1.2917                  1.7555                  1.8642
 IR Inten    --      8.2630                 10.3075                  8.6173

                     31                      32                      33
                      A                       A                       A
 Frequencies --   1484.4503               1494.8531               1506.2273
 Red. masses --      1.1203                  1.0847                  1.1124
 Frc consts  --      1.4546                  1.4280                  1.4869
 IR Inten    --     15.5302                  4.8673                  9.7181

                     34                      35                      36
                      A                       A                       A
 Frequencies --   1516.0952               3037.2031               3045.5619
 Red. masses --      1.2085                  1.0731                  1.0757
 Frc consts  --      1.6366                  5.8324                  5.8787
 IR Inten    --      8.2199                  0.4629                 24.6021


                     37                      38                      39
                      A                       A                       A
 Frequencies --   3059.3675               3061.6074               3126.5485
 Red. masses --      1.0642                  1.0648                  1.1006
 Frc consts  --      5.8689                  5.8807                  6.3388
 IR Inten    --     28.0889                 34.0231                 24.9606
```

```
                     40                     41                     42
                      A                      A                      A
 Frequencies --   3129.3038              3133.7732              3581.3270
 Red. masses --      1.1037                 1.0991                 1.0748
 Frc consts  --      6.3678                 6.3594                 8.1223
 IR Inten    --     17.1989                 9.3230                 2.6124
```

13a

```
                      1                      2                      3
                      A                      A                      A
 Frequencies --    104.1287               157.9502               240.1499
 Red. masses --      4.4807                 3.3303                 4.3580
 Frc consts  --      0.0286                 0.0490                 0.1481
 IR Inten    --      3.0829                 1.8769                 6.0572

                      4                      5                      6
                      A                      A                      A
 Frequencies --    294.1353               391.7566               440.4326
 Red. masses --      2.6830                 1.9230                 3.6523
 Frc consts  --      0.1368                 0.1739                 0.4174
 IR Inten    --      8.5428                 1.9825                10.2410

                      7                      8                      9
                      A                      A                      A
 Frequencies --    478.7821               503.5330               647.7356
 Red. masses --      4.8511                 2.0886                 4.2282
 Frc consts  --      0.6552                 0.3120                 1.0452
 IR Inten    --     11.0597                 3.8275                 4.0342

                     10                     11                     12
                      A                      A                      A
 Frequencies --    716.3711               778.0676               809.7841
 Red. masses --      1.9909                 2.3062                 2.0891
 Frc consts  --      0.6020                 0.8226                 0.8071
 IR Inten    --     24.5443                52.3300               101.5154

                     13                     14                     15
                      A                      A                      A
 Frequencies --    861.9435               897.7486               934.0462
 Red. masses --      2.3288                 1.8728                 2.0227
 Frc consts  --      1.0194                 0.8893                 1.0397
 IR Inten    --      9.2708                18.5083                39.4008

                     16                     17                     18
                      A                      A                      A
 Frequencies --    951.7917              1020.5097              1032.8805
 Red. masses --      1.5272                 2.9396                 1.8901
 Frc consts  --      0.8151                 1.8037                 1.1881
 IR Inten    --     59.1134                 1.2207                 9.0350

                     19                     20                     21
                      A                      A                      A
 Frequencies --   1052.2781              1115.2660              1147.6869
 Red. masses --      2.5104                 2.0475                 1.5931
 Frc consts  --      1.6378                 1.5005                 1.2363
 IR Inten    --     45.3409                13.1350                66.3311

                     22                     23                     24
                      A                      A                      A
 Frequencies --   1189.7352              1215.9892              1255.6910
 Red. masses --      3.1238                 1.1476                 1.3414
 Frc consts  --      2.6051                 0.9997                 1.2462
 IR Inten    --     21.5482                 3.7347                12.3208

                     25                     26                     27
                      A                      A                      A
 Frequencies --   1320.0998              1327.4222              1350.4786
 Red. masses --      1.6974                 1.4058                 1.2345
 Frc consts  --      1.7428                 1.4594                 1.3265
 IR Inten    --      2.2828                11.6533                13.1485

                     28                     29                     30
                      A                      A                      A
```

Frequencies --	1360.6386	1381.2588	1410.6206
Red. masses --	1.3010	1.2118	1.4007
Frc consts --	1.4191	1.3622	1.6422
IR Inten --	3.5498	5.9099	22.2133
	31	32	33
	A	A	A
Frequencies --	1420.2726	1468.7671	1471.3789
Red. masses --	2.0158	1.1100	1.1112
Frc consts --	2.3957	1.4109	1.4174
IR Inten --	8.1903	4.4598	19.3386
	34	35	36
	A	A	A
Frequencies --	1505.8069	1520.3245	1761.3196
Red. masses --	1.1014	1.2094	9.1855
Frc consts --	1.4714	1.6470	16.7892
IR Inten --	7.1920	13.0588	84.2325
	37	38	39
	A	A	A
Frequencies --	3044.9164	3047.6188	3065.3392
Red. masses --	1.0678	1.0659	1.0634
Frc consts --	5.8330	5.8332	5.8872
IR Inten --	29.8108	19.8426	11.3119
	40	41	42
	A	A	A
Frequencies --	3082.2434	3117.6101	3118.7627
Red. masses --	1.0875	1.0988	1.0999
Frc consts --	6.0871	6.2925	6.3036
IR Inten --	42.4574	33.0881	16.5540
	43	44	45
	A	A	A
Frequencies --	3140.5696	3476.0380	3581.6567
Red. masses --	1.1010	1.0744	1.0743
Frc consts --	6.3982	7.6485	8.1201
IR Inten --	12.2537	2.0660	3.9301

**13e**	1	2	3
	A	A	A
Frequencies --	111.8163	159.4204	283.4200
Red. masses --	4.2671	2.4339	5.1113
Frc consts --	0.0314	0.0364	0.2419
IR Inten --	0.5166	1.4936	5.0185
	4	5	6
	A	A	A
Frequencies --	329.9288	353.9885	400.5435
Red. masses --	2.0258	3.4506	4.5976
Frc consts --	0.1299	0.2548	0.4346
IR Inten --	14.4163	8.0946	0.4124
	7	8	9
	A	A	A
Frequencies --	439.6790	513.8494	598.3456
Red. masses --	2.8946	2.8580	3.8977
Frc consts --	0.3297	0.4446	0.8222
IR Inten --	2.8930	3.2262	21.5871
	10	11	12
	A	A	A
Frequencies --	685.4339	756.8494	778.8753
Red. masses --	1.8723	1.7578	2.5455
Frc consts --	0.5183	0.5933	0.9098
IR Inten --	13.4391	129.6530	30.8911
	13	14	15
	A	A	A
Frequencies --	876.8031	900.3686	925.7533
Red. masses --	1.9316	2.1692	1.3500
Frc consts --	0.8749	1.0361	0.6817
IR Inten --	23.7020	23.4617	25.5383

	16	17	18
	A	A	A
Frequencies --	1006.9312	1026.2600	1055.8715
Red. masses --	2.6438	1.4944	2.4847
Frc consts --	1.5794	0.9273	1.6321
IR Inten --	14.2166	11.6592	34.0661
	19	20	21
	A	A	A
Frequencies --	1088.5564	1116.9787	1141.9650
Red. masses --	2.9159	2.4006	2.3098
Frc consts --	2.0357	1.7647	1.7747
IR Inten --	69.9101	19.3086	16.0563
	22	23	24
	A	A	A
Frequencies --	1192.2490	1196.5234	1250.9294
Red. masses --	1.5235	2.1566	1.3301
Frc consts --	1.2759	1.8191	1.2263
IR Inten --	8.8930	12.7610	12.0058
	25	26	27
	A	A	A
Frequencies --	1320.2227	1323.2338	1336.5049
Red. masses --	1.3229	1.3451	1.3049
Frc consts --	1.3586	1.3877	1.3733
IR Inten --	17.1784	12.5542	3.7474
	28	29	30
	A	A	A
Frequencies --	1365.2422	1375.3139	1401.1056
Red. masses --	1.3129	1.2050	1.4200
Frc consts --	1.4418	1.3429	1.6425
IR Inten --	2.2554	1.3620	27.4893
	31	32	33
	A	A	A
Frequencies --	1429.7746	1469.0999	1492.8824
Red. masses --	1.8521	1.1279	1.0858
Frc consts --	2.2308	1.4343	1.4258
IR Inten --	12.2328	14.8130	6.1584
	34	35	36
	A	A	A
Frequencies --	1503.2113	1510.7099	1767.4290
Red. masses --	1.1101	1.2140	9.8666
Frc consts --	1.4780	1.6324	18.1595
IR Inten --	8.0040	10.4201	96.7598
	37	38	39
	A	A	A
Frequencies --	3042.7141	3053.1745	3054.6654
Red. masses --	1.0688	1.0747	1.0661
Frc consts --	5.8302	5.9026	5.8609
IR Inten --	7.1456	5.0906	32.6566
	40	41	42
	A	A	A
Frequencies --	3062.8737	3116.0581	3129.3413
Red. masses --	1.0703	1.1013	1.1026
Frc consts --	5.9158	6.3003	6.3617
IR Inten --	49.2197	28.9923	22.2853
	43	44	45
	A	A	A
Frequencies --	3134.3454	3487.4583	3575.6982
Red. masses --	1.0978	1.0736	1.0746
Frc consts --	6.3543	7.6934	8.0950
IR Inten --	13.5870	5.2009	1.1476

14a

```
                      1                      2                      3
                      A                      A                      A
 Frequencies --    110.7162               163.8920               243.5637
 Red. masses --      4.6246                 3.4610                 3.7905
 Frc consts  --      0.0334                 0.0548                 0.1325
 IR Inten    --      3.6442                 2.3558                 7.7007

                      4                      5                      6
                      A                      A                      A
 Frequencies --    295.3179               393.0401               442.2362
 Red. masses --      2.7870                 1.9342                 4.4152
 Frc consts  --      0.1432                 0.1760                 0.5088
 IR Inten    --      3.8137                 4.0093                 4.9427

                      7                      8                      9
                      A                      A                      A
 Frequencies --    477.9011               507.7455               650.4429
 Red. masses --      4.0176                 2.1302                 4.0595
 Frc consts  --      0.5406                 0.3236                 1.0119
 IR Inten    --      9.5900                 4.9979                 6.9613

                     10                     11                     12
                      A                      A                      A
 Frequencies --    723.5296               775.7931               814.3616
 Red. masses --      2.1217                 2.3988                 2.0822
 Frc consts  --      0.6544                 0.8506                 0.8136
 IR Inten    --     21.8681                24.5295               114.7400

                     13                     14                     15
                      A                      A                      A
 Frequencies --    863.2103               891.1930               931.5357
 Red. masses --      2.3246                 1.7596                 2.0057
 Frc consts  --      1.0206                 0.8234                 1.0254
 IR Inten    --     24.0388                17.1089                36.3824

                     16                     17                     18
                      A                      A                      A
 Frequencies --    949.2212              1021.3286              1032.4168
 Red. masses --      1.5143                 2.9422                 1.9518
 Frc consts  --      0.8039                 1.8082                 1.2258
 IR Inten    --     15.7481                 1.8614                 6.7816

                     19                     20                     21
                      A                      A                      A
 Frequencies --   1051.4564              1110.3250              1146.1685
 Red. masses --      2.2940                 2.1450                 1.4600
 Frc consts  --      1.4942                 1.5581                 1.1300
 IR Inten    --    100.0751                29.1538                 9.0010

                     22                     23                     24
                      A                      A                      A
 Frequencies --   1186.6580              1209.4657              1249.7727
 Red. masses --      2.8879                 1.1666                 1.4312
 Frc consts  --      2.3960                 1.0054                 1.3171
 IR Inten    --     31.3317                 2.5626                18.1755

                     25                     26                     27
                      A                      A                      A
 Frequencies --   1315.9370              1326.5705              1340.3773
 Red. masses --      1.5988                 1.4289                 1.3071
 Frc consts  --      1.6312                 1.4815                 1.3836
 IR Inten    --      2.5175                 1.9331                 6.2594

                     28                     29                     30
                      A                      A                      A
 Frequencies --   1361.2347              1374.4547              1406.0511
 Red. masses --      1.2962                 1.1943                 1.3778
 Frc consts  --      1.4151                 1.3293                 1.6049
 IR Inten    --     13.6179                 7.0007                10.6298

                     31                     32                     33
                      A                      A                      A
```

Frequencies --	1425.2205	1472.9138	1474.6954
Red. masses --	2.2494	1.0881	1.1049
Frc consts --	2.6921	1.3909	1.4157
IR Inten --	50.3689	1.3679	21.8627
	34	35	36
	A	A	A
Frequencies --	1504.7289	1521.4528	1762.5006
Red. masses --	1.0989	1.2105	9.2147
Frc consts --	1.4660	1.6509	16.8651
IR Inten --	6.5341	13.1038	86.9330
	37	38	39
	A	A	A
Frequencies --	3041.5782	3051.2524	3058.2039
Red. masses --	1.0677	1.0652	1.0595
Frc consts --	5.8198	5.8432	5.8383
IR Inten --	33.0827	24.0629	14.0239
	40	41	42
	A	A	A
Frequencies --	3110.3125	3117.5048	3118.5235
Red. masses --	1.0999	1.1028	1.1019
Frc consts --	6.2694	6.3148	6.3139
IR Inten --	30.4939	10.7068	29.8033
	43	44	45
	A	A	A
Frequencies --	3132.1497	3480.2211	3583.5148
Red. masses --	1.0885	1.0743	1.0744
Frc consts --	6.2916	7.6666	8.1286
IR Inten --	18.1863	1.9634	4.3036

14e

	1	2	3
	A	A	A
Frequencies --	102.5665	162.7053	288.7590
Red. masses --	4.5669	2.4508	6.1101
Frc consts --	0.0283	0.0382	0.3002
IR Inten --	1.1790	3.6903	0.3478
	4	5	6
	A	A	A
Frequencies --	330.8755	352.3707	404.7063
Red. masses --	1.9913	3.3188	4.5014
Frc consts --	0.1284	0.2428	0.4344
IR Inten --	7.3688	1.0606	7.8011
	7	8	9
	A	A	A
Frequencies --	439.0916	518.3351	597.1728
Red. masses --	2.9217	2.8064	3.6526
Frc consts --	0.3319	0.4442	0.7675
IR Inten --	1.3245	12.8172	11.1350
	10	11	12
	A	A	A
Frequencies --	684.9638	760.9771	784.8116
Red. masses --	1.9218	1.7041	2.5416
Frc consts --	0.5312	0.5814	0.9224
IR Inten --	9.6964	155.3698	14.2576
	13	14	15
	A	A	A
Frequencies --	876.6862	892.4248	915.2568
Red. masses --	2.0797	1.5993	1.7131
Frc consts --	0.9418	0.7504	0.8455
IR Inten --	15.5711	21.2419	15.9137
	16	17	18
	A	A	A
Frequencies --	1006.8304	1021.9550	1058.5967
Red. masses --	2.5123	1.5684	2.5318
Frc consts --	1.5005	0.9651	1.6716
IR Inten --	21.1545	22.4276	1.5970

	19	20	21
	A	A	A
Frequencies --	1097.5769	1127.3628	1142.6396
Red. masses --	2.1508	2.7785	2.5958
Frc consts --	1.5266	2.0806	1.9968
IR Inten --	19.4432	48.8270	78.9843
	22	23	24
	A	A	A
Frequencies --	1183.7992	1198.1270	1247.3608
Red. masses --	1.5482	1.8946	1.3814
Frc consts --	1.2783	1.6024	1.2663
IR Inten --	17.6453	15.3618	19.0450
	25	26	27
	A	A	A
Frequencies --	1318.9831	1332.4605	1341.9127
Red. masses --	1.3946	1.3953	1.2248
Frc consts --	1.4295	1.4596	1.2995
IR Inten --	6.2308	8.1216	17.9999
	28	29	30
	A	A	A
Frequencies --	1365.2298	1374.4911	1399.7193
Red. masses --	1.3442	1.2117	1.3981
Frc consts --	1.4761	1.3487	1.6139
IR Inten --	8.3201	1.2689	5.6133
	31	32	33
	A	A	A
Frequencies --	1440.9357	1474.2678	1493.1090
Red. masses --	1.8612	1.1051	1.0894
Frc consts --	2.2768	1.4151	1.4310
IR Inten --	12.1857	17.0076	5.2889
	34	35	36
	A	A	A
Frequencies --	1503.5000	1511.4689	1768.5521
Red. masses --	1.0999	1.2263	9.4043
Frc consts --	1.4649	1.6506	17.3305
IR Inten --	7.0159	9.9403	92.6969
	37	38	39
	A	A	A
Frequencies --	3031.5942	3042.8463	3052.3106
Red. masses --	1.0810	1.0658	1.0640
Frc consts --	5.8535	5.8142	5.8404
IR Inten --	13.1814	19.1360	30.8774
	40	41	42
	A	A	A
Frequencies --	3061.4344	3104.7527	3116.4311
Red. masses --	1.0649	1.0997	1.1033
Frc consts --	5.8806	6.2456	6.3131
IR Inten --	33.2446	31.2355	27.7343
	43	44	45
	A	A	A
Frequencies --	3130.8966	3491.8296	3574.9779
Red. masses --	1.1025	1.0746	1.0746
Frc consts --	6.3673	7.7194	8.0917
IR Inten --	21.6670	2.1363	1.1728

15a

	1	2	3
	A	A	A
Frequencies --	115.1155	163.5407	241.9274
Red. masses --	3.6576	3.3105	3.6355
Frc consts --	0.0286	0.0522	0.1254
IR Inten --	1.0086	3.0153	6.5719

```
                    4                      5                      6
                    A                      A                      A
Frequencies --    293.4859               383.9027               412.1519
Red. masses --      2.5470                 2.0255                 2.2638
Frc consts  --      0.1293                 0.1759                 0.2266
IR Inten    --      5.0764                 1.8050                 1.6549

                    7                      8                      9
                    A                      A                      A
Frequencies --    467.1428               497.9879               640.2622
Red. masses --      4.5725                 2.1223                 4.0787
Frc consts  --      0.5879                 0.3101                 0.9851
IR Inten    --      3.1176                 2.8136                 1.4725

                   10                     11                     12
                    A                      A                      A
Frequencies --    706.4452               749.9431               789.7344
Red. masses --      1.9095                 1.2303                 2.1585
Frc consts  --      0.5615                 0.4077                 0.7932
IR Inten    --     24.1866                10.2243                28.1735

                   13                     14                     15
                    A                      A                      A
Frequencies --    811.0937               863.9641               908.6924
Red. masses --      2.0101                 2.4951                 2.0683
Frc consts  --      0.7791                 1.0973                 1.0062
IR Inten    --    124.9482                15.1826                 0.5656

                   16                     17                     18
                    A                      A                      A
Frequencies --    926.6319               963.0163              1008.1455
Red. masses --      1.8550                 1.3600                 1.8134
Frc consts  --      0.9385                 0.7431                 1.0859
IR Inten    --     31.4421                37.2037                13.1167

                   19                     20                     21
                    A                      A                      A
Frequencies --   1017.5270              1037.6195              1045.4591
Red. masses --      2.6340                 2.1358                 2.1635
Frc consts  --      1.6068                 1.3549                 1.3932
IR Inten    --      8.6577                61.0174                14.7185

                   22                     23                     24
                    A                      A                      A
Frequencies --   1121.5263              1187.6772              1216.3447
Red. masses --      1.8199                 3.5651                 1.1170
Frc consts  --      1.3487                 2.9629                 0.9737
IR Inten    --     12.8328                27.4141                 6.5595

                   25                     26                     27
                    A                      A                      A
Frequencies --   1253.6885              1313.4240              1324.9028
Red. masses --      1.4836                 1.5888                 1.5888
Frc consts  --      1.3739                 1.6148                 1.6432
IR Inten    --      9.7095                 2.8858                 0.1719

                   28                     29                     30
                    A                      A                      A
Frequencies --   1336.0132              1353.8653              1375.6138
Red. masses --      1.3426                 1.3836                 1.2311
Frc consts  --      1.4119                 1.4942                 1.3725
IR Inten    --      3.1613                11.9020                 2.5150

                   31                     32                     33
                    A                      A                      A
Frequencies --   1392.1384              1407.5615              1462.8290
Red. masses --      1.7080                 1.3943                 1.1532
Frc consts  --      1.9503                 1.6275                 1.4539
IR Inten    --      2.0469                10.6469                 8.4459

                   34                     35                     36
                    A                      A                      A
```

```
 Frequencies --   1471.1829              1483.7810              1501.5104
 Red. masses --      1.0888                 1.1388                 1.0996
 Frc consts  --      1.3885                 1.4772                 1.4607
 IR Inten    --      5.8257                16.4734                 6.4233

                      37                     38                     39
                       A                      A                      A
 Frequencies --   1518.3770              1747.5293              3037.7926
 Red. masses --      1.2101                 5.9775                 1.0677
 Frc consts  --      1.6437                10.7552                 5.8050
 IR Inten    --     12.4088                15.6085                34.0980

                      40                     41                     42
                       A                      A                      A
 Frequencies --   3047.6157              3051.0700              3094.8694
 Red. masses --      1.0654                 1.0617                 1.0877
 Frc consts  --      5.8303                 5.8234                 6.1382
 IR Inten    --     21.4050                21.0880                41.3355

                      43                     44                     45
                       A                      A                      A
 Frequencies --   3106.9332              3112.0063              3116.1802
 Red. masses --      1.0969                 1.1039                 1.1002
 Frc consts  --      6.2382                 6.2990                 6.2948
 IR Inten    --     37.5997                26.9726                25.2487

                      46                     47                     48
                       A                      A                      A
 Frequencies --   3145.6342              3233.3573              3578.7116
 Red. masses --      1.0615                 1.1164                 1.0742
 Frc consts  --      6.1886                 6.8764                 8.1058
 IR Inten    --      7.5390                11.9739                 3.1375
```

15e

```
                       1                      2                      3
                       A                      A                      A
 Frequencies --    115.5288               171.5085               284.3570
 Red. masses --      3.5234                 2.4312                 3.8951
 Frc consts  --      0.0277                 0.0421                 0.1856
 IR Inten    --      1.1009                 1.4236                 2.9167

                       4                      5                      6
                       A                      A                      A
 Frequencies --    326.6084               352.2894               390.2105
 Red. masses --      2.0312                 3.1585                 3.2096
 Frc consts  --      0.1277                 0.2310                 0.2879
 IR Inten    --     10.0573                 1.8898                 3.9725

                       7                      8                      9
                       A                      A                      A
 Frequencies --    440.5715               489.4622               574.8299
 Red. masses --      2.8085                 3.1213                 3.4508
 Frc consts  --      0.3212                 0.4406                 0.6718
 IR Inten    --      2.1700                 2.8511                 9.0231

                      10                     11                     12
                       A                      A                      A
 Frequencies --    664.6529               751.5482               763.9128
 Red. masses --      1.8413                 1.1441                 1.6106
 Frc consts  --      0.4792                 0.3807                 0.5538
 IR Inten    --     12.2754                 4.1579               163.2536

                      13                     14                     15
                       A                      A                      A
 Frequencies --    795.0839               866.4412               900.9088
 Red. masses --      2.3174                 1.9926                 2.0882
 Frc consts  --      0.8631                 0.8814                 0.9986
 IR Inten    --      2.1339                 6.7347                 2.9835

                      16                     17                     18
                       A                      A                      A
 Frequencies --    957.0264               989.6026              1009.8322
 Red. masses --      1.3438                 1.9950                 1.6149
 Frc consts  --      0.7251                 1.1511                 0.9703
 IR Inten    --     46.5221                11.7802                 3.6959
```

```
                     19                     20                     21
                      A                      A                      A
Frequencies --   1014.8496              1081.3864              1093.4128
Red. masses --      2.1051                 2.4495                 5.0101
Frc consts  --      1.2774                 1.6877                 3.5291
IR Inten    --     12.1148                 0.5780                98.5620

                     22                     23                     24
                      A                      A                      A
Frequencies --   1128.2528              1176.5693              1197.1629
Red. masses --      2.0145                 2.1803                 1.5366
Frc consts  --      1.5109                 1.7783                 1.2976
IR Inten    --      2.4434                16.2270                 7.8335

                     25                     26                     27
                      A                      A                      A
Frequencies --   1238.8725              1284.0626              1324.8998
Red. masses --      1.6222                 1.5054                 1.2921
Frc consts  --      1.4669                 1.4625                 1.3363
IR Inten    --      7.8224                 3.6014                 1.0485

                     28                     29                     30
                      A                      A                      A
Frequencies --   1336.7796              1365.2098              1371.7059
Red. masses --      1.3336                 1.4008                 1.2636
Frc consts  --      1.4041                 1.5382                 1.4008
IR Inten    --      1.5186                 1.6361                 2.7615

                     31                     32                     33
                      A                      A                      A
Frequencies --   1391.4390              1412.4064              1450.4561
Red. masses --      1.4042                 1.5676                 1.1786
Frc consts  --      1.6018                 1.8425                 1.4609
IR Inten    --      4.5671                 9.0755                 7.0344

                     34                     35                     36
                      A                      A                      A
Frequencies --   1480.8835              1491.1135              1500.7003
Red. masses --      1.1206                 1.0856                 1.1047
Frc consts  --      1.4479                 1.4221                 1.4658
IR Inten    --     16.0484                 4.0106                 7.9226

                     37                     38                     39
                      A                      A                      A
Frequencies --   1509.7133              1749.7086              3027.4068
Red. masses --      1.2129                 6.2834                 1.0694
Frc consts  --      1.6287                11.3339                 5.7747
IR Inten    --      8.1761                20.2432                10.6497

                     40                     41                     42
                      A                      A                      A
Frequencies --   3035.5922              3049.3762              3058.3133
Red. masses --      1.0809                 1.0639                 1.0636
Frc consts  --      5.8684                 5.8285                 5.8613
IR Inten    --     38.9117                33.7136                37.3027

                     43                     44                     45
                      A                      A                      A
Frequencies --   3104.3237              3112.1329              3122.9499
Red. masses --      1.0957                 1.1032                 1.1038
Frc consts  --      6.2213                 6.2956                 6.3426
IR Inten    --     34.6420                33.2444                28.4770

                     46                     47                     48
                      A                      A                      A
Frequencies --   3162.4630              3258.5388              3570.9375
Red. masses --      1.0616                 1.1158                 1.0745
Frc consts  --      6.2556                 6.9802                 8.0725
IR Inten    --      7.1459                 4.2078                 0.6376
```

16a

```
                      1                      2                      3
                      A                      A                      A
 Frequencies --     86.0322               167.8847               224.4395
 Red. masses --      5.6158                 3.3954                 4.3616
 Frc consts  --      0.0245                 0.0564                 0.1294
 IR Inten    --      7.1728                 0.0271                 3.2755

                      4                      5                      6
                      A                      A                      A
 Frequencies --    296.5316               383.1506               431.5751
 Red. masses --      3.3160                 1.9329                 5.3563
 Frc consts  --      0.1718                 0.1672                 0.5878
 IR Inten    --      1.6562                 4.1830                 7.1492

                      7                      8                      9
                      A                      A                      A
 Frequencies --    467.0641               501.3099               621.5657
 Red. masses --      2.9185                 2.5280                 2.5822
 Frc consts  --      0.3751                 0.3743                 0.5878
 IR Inten    --     28.6333                 9.9220                47.4737

                     10                     11                     12
                      A                      A                      A
 Frequencies --    680.6377               766.6159               814.6839
 Red. masses --      2.4909                 3.4732                 1.9376
 Frc consts  --      0.6799                 1.2026                 0.7577
 IR Inten    --     50.8729                21.0573                13.4884

                     13                     14                     15
                      A                      A                      A
 Frequencies --    891.7450               900.3059               955.1618
 Red. masses --      2.4432                 2.3967                 2.1343
 Frc consts  --      1.1447                 1.1446                 1.1473
 IR Inten    --     11.0385                16.0843                29.1377

                     16                     17                     18
                      A                      A                      A
 Frequencies --   1018.5561              1055.3135              1066.6637
 Red. masses --      2.9589                 2.2613                 1.7180
 Frc consts  --      1.8086                 1.4838                 1.1516
 IR Inten    --      1.9547                35.7639                 2.0297

                     19                     20                     21
                      A                      A                      A
 Frequencies --   1112.5589              1173.8730              1200.2035
 Red. masses --      2.8499                 2.2222                 1.6944
 Frc consts  --      2.0784                 1.8042                 1.4381
 IR Inten    --     29.1646                 3.6315                 7.7612

                     22                     23                     24
                      A                      A                      A
 Frequencies --   1215.4847              1268.9336              1305.1986
 Red. masses --      1.2286                 1.5842                 1.3164
 Frc consts  --      1.0694                 1.5030                 1.3212
 IR Inten    --     15.9378                18.1831                 3.3829

                     25                     26                     27
                      A                      A                      A
 Frequencies --   1329.5489              1350.7215              1372.6730
 Red. masses --      1.5663                 1.3501                 1.5881
 Frc consts  --      1.6313                 1.4513                 1.7631
 IR Inten    --      9.7391                 6.1854                15.4360

                     28                     29                     30
                      A                      A                      A
 Frequencies --   1382.0727              1428.2446              1458.6296
 Red. masses --      1.3852                 1.4954                 1.1126
 Frc consts  --      1.5589                 1.7973                 1.3946
 IR Inten    --     32.3023                 7.1959                16.3282
```

	31	32	33
	A	A	A
Frequencies --	1481.9163	1496.6108	1521.9606
Red. masses --	1.0871	1.3450	1.1081
Frc consts --	1.4066	1.7750	1.5123
IR Inten --	5.4749	12.4797	3.4216
	34	35	36
	A	A	A
Frequencies --	1849.1168	2940.6593	2947.6456
Red. masses --	11.3514	1.0719	1.0732
Frc consts --	22.8680	5.4612	5.4940
IR Inten --	224.6700	72.4706	72.0422
	37	38	39
	A	A	A
Frequencies --	3080.5968	3112.7692	3113.8828
Red. masses --	1.0612	1.0894	1.0901
Frc consts --	5.9335	6.2194	6.2277
IR Inten --	5.1098	27.6116	15.1577
	40	41	42
	A	A	A
Frequencies --	3123.9465	3155.6293	3615.2036
Red. masses --	1.0882	1.1041	1.0756
Frc consts --	6.2573	6.4779	8.2824
IR Inten --	21.3489	9.2571	5.2881

**16e**	1	2	3
	A	A	A
Frequencies --	90.2261	163.4368	275.8385
Red. masses --	5.1263	2.4764	7.4553
Frc consts --	0.0246	0.0390	0.3342
IR Inten --	1.9866	3.8319	2.1530
	4	5	6
	A	A	A
Frequencies --	342.1605	358.4817	400.0986
Red. masses --	2.7183	2.9663	4.1376
Frc consts --	0.1875	0.2246	0.3902
IR Inten --	0.7375	11.0417	5.2405
	7	8	9
	A	A	A
Frequencies --	436.6952	513.8487	604.4732
Red. masses --	3.2786	2.4403	3.9550
Frc consts --	0.3684	0.3796	0.8514
IR Inten --	2.0230	14.0498	25.3402
	10	11	12
	A	A	A
Frequencies --	663.3861	757.3671	791.9584
Red. masses --	2.5408	2.3083	1.6537
Frc consts --	0.6588	0.7801	0.6111
IR Inten --	52.9898	8.4607	37.8689
	13	14	15
	A	A	A
Frequencies --	898.6318	914.3751	993.0370
Red. masses --	2.6016	2.5682	1.4455
Frc consts --	1.2378	1.2651	0.8398
IR Inten --	42.7417	7.5675	5.1857
	16	17	18
	A	A	A
Frequencies --	1014.2005	1096.6759	1118.9054
Red. masses --	2.4762	2.1350	2.9675
Frc consts --	1.5007	1.5129	2.1889
IR Inten --	7.7959	0.9274	21.6962
	19	20	21
	A	A	A
Frequencies --	1124.9484	1165.6506	1188.4433
Red. masses --	3.0769	2.8974	1.6949
Frc consts --	2.2942	2.3195	1.4104

```
 IR Inten    --     65.5900                35.0475                 2.5403

                     22                     23                     24
                      A                      A                      A
 Frequencies --   1203.6499              1264.5171              1298.2891
 Red. masses --      1.4438                 1.3608                 1.3766
 Frc consts  --      1.2324                 1.2820                 1.3671
 IR Inten    --     24.8991                 6.0125                 6.3964

                     25                     26                     27
                      A                      A                      A
 Frequencies --   1325.9496              1346.9976              1361.5958
 Red. masses --      1.2522                 1.3249                 1.3904
 Frc consts  --      1.2971                 1.4163                 1.5187
 IR Inten    --      7.2905                 4.5238                 7.0264

                     28                     29                     30
                      A                      A                      A
 Frequencies --   1398.6684              1434.1231              1458.9927
 Red. masses --      1.7072                 1.5548                 1.0990
 Frc consts  --      1.9677                 1.8841                 1.3784
 IR Inten    --     14.3294                 2.2211                14.8886

                     31                     32                     33
                      A                      A                      A
 Frequencies --   1491.3618              1506.5522              1517.4828
 Red. masses --      1.2272                 1.1586                 1.1035
 Frc consts  --      1.6081                 1.5493                 1.4971
 IR Inten    --     12.1037                 0.8563                 6.6650

                     34                     35                     36
                      A                      A                      A
 Frequencies --   1858.9693              2960.8880              2970.6133
 Red. masses --     12.0496                 1.0726                 1.0716
 Frc consts  --     24.5338                 5.5403                 5.5716
 IR Inten    --    234.8251                74.7112                48.2208

                     37                     38                     39
                      A                      A                      A
 Frequencies --   3064.2875              3070.1740              3111.6604
 Red. masses --      1.0719                 1.0745                 1.0908
 Frc consts  --      5.9300                 5.9675                 6.2225
 IR Inten    --      2.8842                23.4791                27.8036

                     40                     41                     42
                      A                      A                      A
 Frequencies --   3123.3256              3144.8830              3606.6558
 Red. masses --      1.0925                 1.1010                 1.0755
 Frc consts  --      6.2791                 6.4158                 8.2429
 IR Inten    --     19.1993                10.3063                 5.5438
```

17a

```
                      1                      2                      3
                      A                      A                      A
 Frequencies --     82.3767               165.0404               236.2311
 Red. masses --      5.7205                 3.7791                 3.7428
 Frc consts  --      0.0229                 0.0606                 0.1231
 IR Inten    --      2.2924                 0.5059                 3.0080

                      4                      5                      6
                      A                      A                      A
 Frequencies --    270.1701               352.3308               392.8300
 Red. masses --      4.6396                 3.0488                 2.3390
 Frc consts  --      0.1995                 0.2230                 0.2127
 IR Inten    --      1.4426                 2.5933                 5.2326

                      7                      8                      9
                      A                      A                      A
 Frequencies --    413.5029               486.1813               596.5183
 Red. masses --      4.8766                 2.0323                 2.4980
 Frc consts  --      0.4913                 0.2830                 0.5237
```

```
IR Inten    --     10.6237                24.6570                65.5939



                     10                     11                     12
                      A                      A                      A
Frequencies --    650.9503               745.9798               802.4858
Red. masses --      3.4956                 3.1980                 1.8075
Frc consts  --      0.8727                 1.0485                 0.6858
IR Inten    --     23.8533                22.9227                25.9992

                     13                     14                     15
                      A                      A                      A
Frequencies --    884.9297               893.9685               948.9005
Red. masses --      2.8989                 2.4807                 2.1639
Frc consts  --      1.3375                 1.1681                 1.1480
IR Inten    --      8.2213                17.3291                34.3635

                     16                     17                     18
                      A                      A                      A
Frequencies --   1007.3557              1047.5545              1067.6355
Red. masses --      2.7686                 1.9134                 1.9651
Frc consts  --      1.6553                 1.2371                 1.3197
IR Inten    --      1.3986                12.9468                11.8603

                     19                     20                     21
                      A                      A                      A
Frequencies --   1091.2327              1167.5257              1188.0442
Red. masses --      2.6509                 2.0660                 1.7325
Frc consts  --      1.8599                 1.6593                 1.4408
IR Inten    --     61.4668                 3.2079                17.0267

                     22                     23                     24
                      A                      A                      A
Frequencies --   1207.9772              1253.5853              1262.7425
Red. masses --      1.7907                 1.9111                 1.4350
Frc consts  --      1.5395                 1.7695                 1.3481
IR Inten    --     16.8852                38.9914                10.8033

                     25                     26                     27
                      A                      A                      A
Frequencies --   1304.8629              1335.0394              1347.4665
Red. masses --      1.3180                 1.2485                 1.4438
Frc consts  --      1.3221                 1.3111                 1.5446
IR Inten    --     26.7977                 3.6695                18.7115

                     28                     29                     30
                      A                      A                      A
Frequencies --   1371.8807              1398.7797              1433.7067
Red. masses --      1.5054                 1.8759                 1.5510
Frc consts  --      1.6693                 2.1625                 1.8784
IR Inten    --     16.2561                48.3488                 1.6542

                     31                     32                     33
                      A                      A                      A
Frequencies --   1477.4411              1485.2185              1493.5541
Red. masses --      1.1444                 1.0906                 1.3342
Frc consts  --      1.4719                 1.4174                 1.7535
IR Inten    --     13.1317                 6.2919                15.3182

                     34                     35                     36
                      A                      A                      A
Frequencies --   1515.5144              2950.8648              2954.5783
Red. masses --      1.1132                 1.0715                 1.0727
Frc consts  --      1.5064                 5.4974                 5.5172
IR Inten    --      5.5576                66.4426                59.6196

                     37                     38                     39
                      A                      A                      A
Frequencies --   3069.5592              3113.3231              3114.1631
Red. masses --      1.0628                 1.0899                 1.0911
Frc consts  --      5.8998                 6.2242                 6.2342
IR Inten    --      3.8510                26.8946                17.3779
```

	40	41	42
	A	A	A
Frequencies --	3129.9077	3152.3857	3615.2864
Red. masses --	1.0886	1.1021	1.0756
Frc consts --	6.2830	6.4527	8.2826
IR Inten --	14.5392	6.5003	6.5772

17e

	1	2	3
	A	A	A
Frequencies --	77.1469	170.2108	235.7503
Red. masses --	4.9452	2.7000	13.7609
Frc consts --	0.0173	0.0461	0.4506
IR Inten --	0.3633	2.4639	1.0508
	4	5	6
	A	A	A
Frequencies --	316.7612	366.4978	376.0195
Red. masses --	2.9234	3.4132	2.9174
Frc consts --	0.1728	0.2701	0.2430
IR Inten --	2.9289	9.1567	2.1169
	7	8	9
	A	A	A
Frequencies --	438.4577	484.0394	581.1267
Red. masses --	3.4237	3.1845	4.1520
Frc consts --	0.3878	0.4396	0.8261
IR Inten --	1.1278	7.6515	8.9956
	10	11	12
	A	A	A
Frequencies --	607.0457	741.3755	780.8703
Red. masses --	2.3784	3.2230	1.5376
Frc consts --	0.5164	1.0437	0.5524
IR Inten --	62.1060	0.7713	69.4990
	13	14	15
	A	A	A
Frequencies --	862.2925	910.8458	1001.7116
Red. masses --	2.1554	2.6438	1.9476
Frc consts --	0.9442	1.2923	1.1514
IR Inten --	16.9263	3.0765	11.4239
	16	17	18
	A	A	A
Frequencies --	1014.6907	1075.6076	1098.7907
Red. masses --	1.8434	1.9011	2.6520
Frc consts --	1.1182	1.2959	1.8865
IR Inten --	3.0127	16.9277	1.0413
	19	20	21
	A	A	A
Frequencies --	1123.5601	1159.8438	1185.8249
Red. masses --	3.4272	2.8790	1.6663
Frc consts --	2.5491	2.2818	1.3806
IR Inten --	37.1615	52.1185	2.0100
	22	23	24
	A	A	A
Frequencies --	1202.6429	1218.9778	1283.0176
Red. masses --	1.5995	1.7147	1.4076
Frc consts --	1.3631	1.5012	1.3652
IR Inten --	14.1283	25.6448	3.7958
	25	26	27
	A	A	A
Frequencies --	1318.7400	1335.6444	1342.7501
Red. masses --	1.2914	1.3397	1.4164
Frc consts --	1.3232	1.4081	1.5046
IR Inten --	0.7106	21.7167	27.4851
	28	29	30
	A	A	A
Frequencies --	1367.7080	1396.5414	1431.5367
Red. masses --	2.0942	1.6161	1.5259
Frc consts --	2.3081	1.8571	1.8424

```
 IR Inten    --     51.0011                17.2157                 4.9214

                     31                     32                     33
                      A                      A                      A
 Frequencies --   1473.7800              1490.2634              1509.7844
 Red. masses --      1.1109                 1.2747                 1.1200
 Frc consts  --      1.4216                 1.6679                 1.5042
 IR Inten    --     14.4214                16.7291                 1.2427

                     34                     35                     36
                      A                      A                      A
 Frequencies --   1522.8448              2968.3730              2972.8991
 Red. masses --      1.1034                 1.0720                 1.0711
 Frc consts  --      1.5077                 5.5652                 5.5776
 IR Inten    --      8.4876                67.6726                39.9675

                     37                     38                     39
                      A                      A                      A
 Frequencies --   3052.0411              3065.8618              3114.6570
 Red. masses --      1.0671                 1.0816                 1.0913
 Frc consts  --      5.8564                 5.9900                 6.2378
 IR Inten    --      4.0986                21.4018                29.2002

                     40                     41                     42
                      A                      A                      A
 Frequencies --   3119.9680              3147.8535              3607.7580
 Red. masses --      1.0930                 1.0980                 1.0754
 Frc consts  --      6.2688                 6.4103                 8.2471
 IR Inten    --     19.4489                 6.9987                 6.3080
```

18a

```
                      1                      2                      3
                      A                      A                      A
 Frequencies --     95.0521               171.6274               231.6182
 Red. masses --      4.2885                 3.3917                 4.3211
 Frc consts  --      0.0228                 0.0589                 0.1366
 IR Inten    --      3.0313                 0.3619                 3.1069

                      4                      5                      6
                      A                      A                      A
 Frequencies --    300.5078               387.2857               429.6379
 Red. masses --      3.0286                 1.9209                 3.9667
 Frc consts  --      0.1611                 0.1698                 0.4314
 IR Inten    --      1.0783                 5.3710                19.1640

                      7                      8                      9
                      A                      A                      A
 Frequencies --    461.0815               496.3925               631.2419
 Red. masses --      3.3324                 2.1126                 2.6528
 Frc consts  --      0.4174                 0.3067                 0.6228
 IR Inten    --     15.0011                16.4883                46.4403

                     10                     11                     12
                      A                      A                      A
 Frequencies --    666.7291               779.1152               808.2298
 Red. masses --      2.5968                 3.2080                 1.8070
 Frc consts  --      0.6801                 1.1473                 0.6955
 IR Inten    --     28.6933                35.8520                18.9837

                     13                     14                     15
                      A                      A                      A
 Frequencies --    888.3876               903.9383               938.0761
 Red. masses --      2.0103                 2.4332                 1.7641
 Frc consts  --      0.9348                 1.1714                 0.9147
 IR Inten    --      7.0798                 8.3435               117.8907

                     16                     17                     18
                      A                      A                      A
 Frequencies --    964.7816              1020.3984              1071.6963
 Red. masses --      1.6847                 3.1250                 1.9024
 Frc consts  --      0.9239                 1.9171                 1.2874
 IR Inten    --      3.0236                 2.4741                18.4644
```

	19	20	21
	A	A	A
Frequencies --	1074.8126	1110.9249	1141.5224
Red. masses --	1.7859	2.4702	1.6707
Frc consts --	1.2156	1.7962	1.2827
IR Inten --	2.2289	26.7049	52.7336

	22	23	24
	A	A	A
Frequencies --	1201.6806	1219.1678	1227.7239
Red. masses --	2.7425	1.1925	1.0970
Frc consts --	2.3334	1.0443	0.9742
IR Inten --	14.6426	9.7662	8.0645

	25	26	27
	A	A	A
Frequencies --	1309.3616	1333.6635	1339.7821
Red. masses --	1.2989	1.5278	1.3231
Frc consts --	1.3120	1.6011	1.3994
IR Inten --	9.2202	18.9685	10.5789

	28	29	30
	A	A	A
Frequencies --	1368.8014	1374.3435	1416.3391
Red. masses --	1.3920	1.2559	2.1189
Frc consts --	1.5366	1.3976	2.5043
IR Inten --	13.1233	15.9024	21.7338

	31	32	33
	A	A	A
Frequencies --	1428.9792	1464.2319	1480.9107
Red. masses --	1.5245	1.1361	1.0872
Frc consts --	1.8342	1.4351	1.4048
IR Inten --	7.0229	16.4901	5.1259

	34	35	36
	A	A	A
Frequencies --	1497.5558	1518.4871	1763.8807
Red. masses --	1.3472	1.1134	9.2590
Frc consts --	1.7801	1.5126	16.9729
IR Inten --	12.4366	3.6294	75.8026

	37	38	39
	A	A	A
Frequencies --	2933.7725	2946.2650	3076.1146
Red. masses --	1.0720	1.0730	1.0627
Frc consts --	5.4362	5.4876	5.9247
IR Inten --	70.5366	77.3283	7.6055

	40	41	42
	A	A	A
Frequencies --	3078.1611	3111.2436	3112.7248
Red. masses --	1.0874	1.0893	1.0900
Frc consts --	6.0705	6.2126	6.2225
IR Inten --	35.4233	39.6083	13.3548

	43	44	45
	A	A	A
Frequencies --	3149.8396	3477.5311	3609.7408
Red. masses --	1.1024	1.0744	1.0755
Frc consts --	6.4439	7.6551	8.2568
IR Inten --	9.4717	1.9074	4.0270

18e

	1	2	3
	A	A	A
Frequencies --	109.2360	171.3759	297.5640
Red. masses --	4.1136	2.4925	4.4607
Frc consts --	0.0289	0.0431	0.2327
IR Inten --	0.4881	2.3669	6.0088

```
                      4                      5                      6
                      A                      A                      A
Frequencies --    345.9156               362.9932               399.8600
Red. masses --      2.7110                 3.0931                 4.1638
Frc consts  --      0.1911                 0.2401                 0.3922
IR Inten    --      0.4385                14.5743                 1.3955

                      7                      8                      9
                      A                      A                      A
Frequencies --    438.6448               507.1783               597.2567
Red. masses --      3.1494                 2.4071                 3.6043
Frc consts  --      0.3570                 0.3648                 0.7575
IR Inten    --      0.3387                25.1567                19.1949

                     10                     11                     12
                      A                      A                      A
Frequencies --    658.8293               777.7470               786.9070
Red. masses --      2.4478                 2.5024                 1.5428
Frc consts  --      0.6260                 0.8918                 0.5629
IR Inten    --     38.4974                 3.3322                62.3576

                     13                     14                     15
                      A                      A                      A
Frequencies --    896.3407               906.3728               936.6738
Red. masses --      2.4166                 2.1075                 1.2792
Frc consts  --      1.1439                 1.0201                 0.6612
IR Inten    --     51.8107                 5.3059                40.3021

                     16                     17                     18
                      A                      A                      A
Frequencies --   1005.0258              1055.1168              1092.3604
Red. masses --      2.4531                 1.5424                 2.5408
Frc consts  --      1.4599                 1.0117                 1.7863
IR Inten    --      5.3239                17.7508                51.9483

                     19                     20                     21
                      A                      A                      A
Frequencies --   1100.9096              1117.3823              1139.4004
Red. masses --      2.1808                 2.4376                 2.5600
Frc consts  --      1.5573                 1.7932                 1.9581
IR Inten    --     19.3475                 9.0925                24.0775

                     22                     23                     24
                      A                      A                      A
Frequencies --   1191.6527              1212.1804              1240.5538
Red. masses --      2.9589                 1.1117                 1.2216
Frc consts  --      2.4756                 0.9625                 1.1077
IR Inten    --     11.9892                 8.0459                14.4130

                     25                     26                     27
                      A                      A                      A
Frequencies --   1299.6238              1326.2802              1332.7231
Red. masses --      1.3504                 1.2852                 1.2312
Frc consts  --      1.3438                 1.3319                 1.2884
IR Inten    --      3.8829                 9.5709                29.0432

                     28                     29                     30
                      A                      A                      A
Frequencies --   1362.1945              1367.5226              1422.9311
Red. masses --      1.3508                 1.3506                 1.7339
Frc consts  --      1.4768                 1.4881                 2.0685
IR Inten    --      6.9072                 2.7006                34.5005

                     31                     32                     33
                      A                      A                      A
Frequencies --   1436.4012              1464.7780              1490.2617
Red. masses --      1.7253                 1.1214                 1.2280
Frc consts  --      2.0973                 1.4176                 1.6068
IR Inten    --      2.9920                16.0877                10.7881

                     34                     35                     36
```

```
                       A                      A                      A
 Frequencies --   1506.7847              1516.8584              1769.8599
 Red. masses --      1.1655                 1.1034                 9.9738
 Frc consts  --      1.5591                 1.4958                18.4073
 IR Inten    --      0.8837                 7.2957                91.1079

                      37                     38                     39
                       A                      A                      A
 Frequencies --   2960.0459              2968.1875              3063.5561
 Red. masses --      1.0724                 1.0713                 1.0650
 Frc consts  --      5.5361                 5.5609                 5.8892
 IR Inten    --     71.7907                51.4204                 9.5161

                      40                     41                     42
                       A                      A                      A
 Frequencies --   3084.0962              3110.1554              3121.1627
 Red. masses --      1.0839                 1.0909                 1.0926
 Frc consts  --      6.0741                 6.2174                 6.2712
 IR Inten    --     19.9031                32.7152                23.3839

                      43                     44                     45
                       A                      A                      A
 Frequencies --   3144.3942              3494.0377              3603.5650
 Red. masses --      1.0997                 1.0734                 1.0755
 Frc consts  --      6.4061                 7.7209                 8.2286
 IR Inten    --     10.2201                 4.6127                 4.4678
```

19a

```
                       1                      2                      3
                       A                      A                      A
 Frequencies --    103.4099               174.7959               231.3072
 Red. masses --      4.3917                 3.5505                 3.8267
 Frc consts  --      0.0277                 0.0639                 0.1206
 IR Inten    --      3.3055                 0.0878                 2.6257

                       4                      5                      6
                       A                      A                      A
 Frequencies --    302.8111               387.7483               433.3181
 Red. masses --      3.0624                 1.9475                 4.8529
 Frc consts  --      0.1654                 0.1725                 0.5369
 IR Inten    --      3.2210                 3.9799                 3.6818

                       7                      8                      9
                       A                      A                      A
 Frequencies --    464.0761               497.5276               635.8278
 Red. masses --      3.1047                 2.0309                 2.5345
 Frc consts  --      0.3940                 0.2962                 0.6037
 IR Inten    --     42.5566                 1.5821                38.8144

                      10                     11                     12
                       A                      A                      A
 Frequencies --    672.1465               779.7012               809.9965
 Red. masses --      2.7591                 3.4274                 1.8738
 Frc consts  --      0.7344                 1.2276                 0.7243
 IR Inten    --     42.4275                20.5169                24.9852

                      13                     14                     15
                       A                      A                      A
 Frequencies --    890.6358               903.3979               933.8443
 Red. masses --      1.9406                 2.4617                 1.6889
 Frc consts  --      0.9070                 1.1837                 0.8678
 IR Inten    --     26.8146                14.0432                12.8326

                      16                     17                     18
                       A                      A                      A
 Frequencies --    964.2067              1021.0987              1073.5147
 Red. masses --      1.6840                 3.1446                 1.7536
 Frc consts  --      0.9224                 1.9318                 1.1907
 IR Inten    --     41.0781                 1.3306                 6.4482

                      19                     20                     21
                       A                      A                      A
 Frequencies --   1076.7453              1109.2027              1143.2348
 Red. masses --      2.0351                 2.4701                 1.6496
 Frc consts  --      1.3901                 1.7906                 1.2703
 IR Inten    --     71.6712                23.2233                15.8734
```

	22	23	24
	A	A	A
Frequencies --	1201.1942	1220.0856	1235.9702
Red. masses --	2.8367	1.1707	1.1052
Frc consts --	2.4115	1.0268	0.9948
IR Inten --	21.7326	3.7327	10.9208
	25	26	27
	A	A	A
Frequencies --	1314.1856	1331.1100	1358.4788
Red. masses --	1.2645	1.4273	1.4800
Frc consts --	1.2867	1.4901	1.6092
IR Inten --	4.3255	10.2040	16.8982
	28	29	30
	A	A	A
Frequencies --	1366.1349	1385.7166	1423.1810
Red. masses --	1.3084	1.2376	2.3892
Frc consts --	1.4388	1.4001	2.8512
IR Inten --	7.2301	20.1611	49.3497
	31	32	33
	A	A	A
Frequencies --	1437.7279	1466.3434	1484.0629
Red. masses --	1.4989	1.1079	1.0886
Frc consts --	1.8254	1.4036	1.4126
IR Inten --	11.1481	14.1807	5.8353
	34	35	36
	A	A	A
Frequencies --	1502.2919	1521.3915	1767.6604
Red. masses --	1.3366	1.1181	9.1975
Frc consts --	1.7774	1.5248	16.9324
IR Inten --	12.7301	3.3573	78.0436
	37	38	39
	A	A	A
Frequencies --	2939.5078	2945.9353	3069.7989
Red. masses --	1.0727	1.0722	1.0595
Frc consts --	5.4613	5.4822	5.8829
IR Inten --	85.6308	62.6362	13.2990
	40	41	42
	A	A	A
Frequencies --	3104.0296	3115.0661	3122.8446
Red. masses --	1.0895	1.0899	1.0887
Frc consts --	6.1849	6.2310	6.2557
IR Inten --	35.1274	13.9732	22.9382
	43	44	45
	A	A	A
Frequencies --	3128.0090	3482.9566	3614.0729
Red. masses --	1.1055	1.0743	1.0755
Frc consts --	6.3730	7.6788	8.2768
IR Inten --	12.7273	1.9569	4.1339

**19e**	1	2	3
	A	A	A
Frequencies --	97.0669	172.3516	288.4591
Red. masses --	4.3818	2.5062	5.5796
Frc consts --	0.0243	0.0439	0.2735
IR Inten --	0.7992	3.7193	0.2375
	4	5	6
	A	A	A
Frequencies --	343.5006	360.4944	400.8312
Red. masses --	2.5943	2.8939	3.8603
Frc consts --	0.1804	0.2216	0.3654
IR Inten --	3.3279	5.5179	11.5421

```
                    7                      8                      9
                    A                      A                      A
Frequencies --    436.9536               508.9983               593.0026
Red. masses --      3.1365                 2.4210                 3.3839
Frc consts  --      0.3528                 0.3696                 0.7011
IR Inten    --      2.4690                 4.9880                36.5698

                   10                     11                     12
                    A                      A                      A
Frequencies --    658.1601               782.1276               786.4170
Red. masses --      2.4999                 1.9987                 1.7946
Frc consts  --      0.6380                 0.7204                 0.6539
IR Inten    --     36.6492                23.8968                40.0860

                   13                     14                     15
                    A                      A                      A
Frequencies --    893.3469               903.6409               923.4983
Red. masses --      2.5409                 1.5398                 1.6878
Frc consts  --      1.1947                 0.7408                 0.8481
IR Inten    --     18.4992                31.3426                11.1324

                   16                     17                     18
                    A                      A                      A
Frequencies --   1003.6624              1053.1885              1089.8497
Red. masses --      2.3983                 1.5539                 1.8340
Frc consts  --      1.4234                 1.0155                 1.2834
IR Inten    --     25.7225                14.2866                22.7081

                   19                     20                     21
                    A                      A                      A
Frequencies --   1107.6602              1116.2478              1146.0952
Red. masses --      2.1767                 2.8335                 3.2787
Frc consts  --      1.5735                 2.0802                 2.5374
IR Inten    --     11.2190                 9.2055               101.8248

                   22                     23                     24
                    A                      A                      A
Frequencies --   1193.1984              1211.5119              1226.7350
Red. masses --      3.0737                 1.1239                 1.1526
Frc consts  --      2.5783                 0.9720                 1.0220
IR Inten    --     17.5633                 6.2509                11.8782

                   25                     26                     27
                    A                      A                      A
Frequencies --   1297.8995              1330.5647              1336.9054
Red. masses --      1.3663                 1.2406                 1.3203
Frc consts  --      1.3561                 1.2941                 1.3903
IR Inten    --      6.7353                 6.3658                26.2603

                   28                     29                     30
                    A                      A                      A
Frequencies --   1359.9475              1368.2468              1423.1444
Red. masses --      1.3074                 1.4221                 1.7105
Frc consts  --      1.4246                 1.5686                 2.0412
IR Inten    --     10.1623                12.8916                 2.5430

                   31                     32                     33
                    A                      A                      A
Frequencies --   1441.4968              1466.1619              1490.1407
Red. masses --      1.7464                 1.1083                 1.2394
Frc consts  --      2.1381                 1.4037                 1.6215
IR Inten    --      9.9346                15.7906                11.9832

                   34                     35                     36
                    A                      A                      A
Frequencies --   1506.0515              1517.1425              1770.7197
Red. masses --      1.1583                 1.1046                 9.5005
Frc consts  --      1.5480                 1.4979                17.5508
IR Inten    --      0.9174                 7.3254                86.8360

                   37                     38                     39
```

	A	A	A
Frequencies --	2954.9397	2971.5015	3058.5132
Red. masses --	1.0725	1.0713	1.0663
Frc consts --	5.5174	5.5733	5.8769
IR Inten --	73.0232	50.2649	3.9862
	40	41	42
	A	A	A
Frequencies --	3064.5411	3108.2616	3120.2485
Red. masses --	1.0783	1.0903	1.1037
Frc consts --	5.9662	6.2064	6.3312
IR Inten --	31.1415	33.6501	20.5215
	43	44	45
	A	A	A
Frequencies --	3123.8957	3490.3587	3603.1219
Red. masses --	1.0925	1.0746	1.0755
Frc consts --	6.2817	7.7132	8.2264
IR Inten --	22.1865	2.0872	4.4604

**20a**	1	2	3
	A	A	A
Frequencies --	106.8183	175.5039	230.9133
Red. masses --	3.4805	3.4521	3.6094
Frc consts --	0.0234	0.0626	0.1134
IR Inten --	0.7190	0.9267	2.2733
	4	5	6
	A	A	A
Frequencies --	301.2841	381.5738	404.0571
Red. masses --	2.7998	2.0552	2.3808
Frc consts --	0.1497	0.1763	0.2290
IR Inten --	1.0312	4.2588	2.8228
	7	8	9
	A	A	A
Frequencies --	454.3725	486.1609	640.7943
Red. masses --	3.7803	1.9763	3.1794
Frc consts --	0.4598	0.2752	0.7692
IR Inten --	25.0760	8.3041	22.2904
	10	11	12
	A	A	A
Frequencies --	646.5897	743.6523	794.4739
Red. masses --	2.2476	1.2009	2.6189
Frc consts --	0.5536	0.3913	0.9739
IR Inten --	43.4355	10.8875	31.8985
	13	14	15
	A	A	A
Frequencies --	807.8572	894.3995	907.7320
Red. masses --	1.7688	2.3417	2.5327
Frc consts --	0.6801	1.1037	1.2296
IR Inten --	19.7277	27.3745	6.7199
	16	17	18
	A	A	A
Frequencies --	939.0477	962.7435	1013.2341
Red. masses --	1.9056	1.3614	1.8991
Frc consts --	0.9900	0.7435	1.1487
IR Inten --	36.9328	37.6732	10.3345
	19	20	21
	A	A	A
Frequencies --	1020.9351	1063.8205	1084.3782
Red. masses --	2.5161	2.0784	1.8067
Frc consts --	1.5451	1.3859	1.2517
IR Inten --	5.8372	46.8445	0.7629
	22	23	24
	A	A	A
Frequencies --	1108.2548	1202.9159	1222.6635
Red. masses --	2.2303	2.8357	1.2938
Frc consts --	1.6140	2.4176	1.1396
IR Inten --	19.9358	17.5839	4.7472

	25	26	27
	A	A	A
Frequencies --	1235.8991	1312.9124	1323.4976
Red. masses --	1.1056	1.4060	1.3656
Frc consts --	0.9950	1.4279	1.4093
IR Inten --	6.0580	1.8935	3.6662

	28	29	30
	A	A	A
Frequencies --	1338.9427	1366.7597	1377.1485
Red. masses --	1.6116	1.3253	1.2661
Frc consts --	1.7023	1.4586	1.4147
IR Inten --	24.9835	4.4062	16.7774

	31	32	33
	A	A	A
Frequencies --	1389.8821	1437.0580	1460.1050
Red. masses --	1.8500	1.5241	1.1390
Frc consts --	2.1056	1.8544	1.4307
IR Inten --	7.9430	4.1056	8.2355

	34	35	36
	A	A	A
Frequencies --	1478.5503	1483.4183	1502.1574
Red. masses --	1.1485	1.0918	1.3366
Frc consts --	1.4792	1.4155	1.7769
IR Inten --	13.9982	6.6735	11.9920

	37	38	39
	A	A	A
Frequencies --	1517.0504	1751.2315	2938.0250
Red. masses --	1.1259	6.0142	1.0728
Frc consts --	1.5267	10.8672	5.4558
IR Inten --	3.8402	12.4205	78.7328

	40	41	42
	A	A	A
Frequencies --	2939.0640	3067.1072	3086.9378
Red. masses --	1.0720	1.0608	1.0875
Frc consts --	5.4557	5.8798	6.1057
IR Inten --	77.0577	14.8543	33.6412

	43	44	45
	A	A	A
Frequencies --	3101.9654	3111.7010	3123.8999
Red. masses --	1.0893	1.0895	1.1042
Frc consts --	6.1756	6.2154	6.3486
IR Inten --	42.4611	25.6719	18.9772

	46	47	48
	A	A	A
Frequencies --	3147.8638	3235.1627	3610.1438
Red. masses --	1.0618	1.1161	1.0754
Frc consts --	6.1992	6.8826	8.2581
IR Inten --	8.3533	12.1994	2.7422

20e

	1	2	3
	A	A	A
Frequencies --	113.0675	183.0919	289.9914
Red. masses --	3.4310	2.5053	3.7091
Frc consts --	0.0258	0.0495	0.1838
IR Inten --	1.0453	1.4608	4.0375

	4	5	6
	A	A	A
Frequencies --	337.7101	363.9578	390.2033
Red. masses --	2.5478	2.8754	3.0814
Frc consts --	0.1712	0.2244	0.2764

```
 IR Inten    --      3.3185                 7.8357                 4.3591



                       7                      8                      9
                       A                      A                      A
 Frequencies --    440.8414               481.9789               572.7861
 Red. masses --      3.0147                 2.5976                 3.0410
 Frc consts  --      0.3452                 0.3555                 0.5878
 IR Inten    --      0.2518                10.1187                20.9278

                      10                     11                     12
                       A                      A                      A
 Frequencies --    638.6733               751.2685               779.9709
 Red. masses --      2.2974                 1.1802                 1.5412
 Frc consts  --      0.5521                 0.3925                 0.5524
 IR Inten    --     29.9023                 0.7153                74.5920

                      13                     14                     15
                       A                      A                      A
 Frequencies --    805.1218               879.9792               911.7408
 Red. masses --      2.0626                 2.0154                 2.3654
 Frc consts  --      0.7877                 0.9195                 1.1585
 IR Inten    --      2.8017                14.7166                 2.6244

                      16                     17                     18
                       A                      A                      A
 Frequencies --    959.8195               982.1115              1029.2201
 Red. masses --      1.3437                 2.1109                 1.7138
 Frc consts  --      0.7294                 1.1996                 1.0696
 IR Inten    --     46.9959                21.9430                 5.8559

                      19                     20                     21
                       A                      A                      A
 Frequencies --   1046.2846              1094.1347              1108.4801
 Red. masses --      1.6996                 2.9952                 2.4545
 Frc consts  --      1.0962                 2.1126                 1.7769
 IR Inten    --      4.9420                49.8817                12.6855

                      22                     23                     24
                       A                      A                      A
 Frequencies --   1124.8932              1190.5898              1208.8615
 Red. masses --      2.6205                 3.3762                 1.3734
 Frc consts  --      1.9537                 2.8197                 1.1825
 IR Inten    --     27.7822                17.1259                 1.6503

                      25                     26                     27
                       A                      A                      A
 Frequencies --   1213.5416              1287.7449              1303.1842
 Red. masses --      1.0932                 1.4834                 1.3453
 Frc consts  --      0.9485                 1.4494                 1.3461
 IR Inten    --      5.3874                 2.4790                 4.0551

                      28                     29                     30
                       A                      A                      A
 Frequencies --   1327.5651              1361.3157              1366.2335
 Red. masses --      1.2465                 1.3549                 1.3687
 Frc consts  --      1.2943                 1.4794                 1.5053
 IR Inten    --      6.5820                 8.1170                11.1120

                      31                     32                     33
                       A                      A                      A
 Frequencies --   1403.8800              1430.3700              1452.5516
 Red. masses --      1.7811                 1.5492                 1.1564
 Frc consts  --      2.0682                 1.8674                 1.4376
 IR Inten    --      1.4594                 2.9223                 5.2119

                      34                     35                     36
                       A                      A                      A
 Frequencies --   1475.7242              1487.6088              1504.9656
 Red. masses --      1.1299                 1.2402                 1.1609
 Frc consts  --      1.4498                 1.6170                 1.5491
 IR Inten    --     15.7826                10.5219                 1.0868

                      37                     38                     39
```

	A	A	A
Frequencies --	1515.0745	1751.9618	2954.1869
Red. masses --	1.1038	6.2435	1.0724
Frc consts --	1.4928	11.2909	5.5141
IR Inten --	7.8577	17.9500	71.7318

	40	41	42
	A	A	A
Frequencies --	2969.4304	3051.5387	3068.3578
Red. masses --	1.0706	1.0638	1.0837
Frc consts --	5.5620	5.8364	6.0112
IR Inten --	55.9552	15.7114	29.1316

	43	44	45
	A	A	A
Frequencies --	3105.6868	3113.5425	3116.9219
Red. masses --	1.0906	1.0931	1.1007
Frc consts --	6.1975	6.2434	6.3006
IR Inten --	41.3306	29.4674	23.0573

	46	47	48
	A	A	A
Frequencies --	3162.0300	3257.3726	3602.6783
Red. masses --	1.0616	1.1158	1.0754
Frc consts --	6.2537	6.9753	8.2240
IR Inten --	7.7851	4.5290	3.2553

21a

	1	2	3
	A	A	A
Frequencies --	96.7339	154.8942	234.7893
Red. masses --	5.4967	3.2610	4.0258
Frc consts --	0.0303	0.0461	0.1308
IR Inten --	6.3116	0.5396	5.3804

	4	5	6
	A	A	A
Frequencies --	286.7747	369.9112	436.9408
Red. masses --	2.5663	2.0634	4.6860
Frc consts --	0.1243	0.1664	0.5271
IR Inten --	2.2809	1.3659	2.3782

	7	8	9
	A	A	A
Frequencies --	467.3242	499.4383	620.8526
Red. masses --	2.5896	2.9778	3.3574
Frc consts --	0.3332	0.4376	0.7625
IR Inten --	4.4385	12.8106	8.9923

	10	11	12
	A	A	A
Frequencies --	709.4216	763.0406	827.6799
Red. masses --	2.7086	2.9490	1.6331
Frc consts --	0.8032	1.0116	0.6591
IR Inten --	2.2208	4.9153	4.4343

	13	14	15
	A	A	A
Frequencies --	852.7177	903.0581	941.6732
Red. masses --	2.6382	2.0735	1.6112
Frc consts --	1.1302	0.9963	0.8418
IR Inten --	0.8591	3.3207	12.8288

	16	17	18
	A	A	A
Frequencies --	991.8758	1014.4124	1045.7281
Red. masses --	2.4424	2.8818	1.9694
Frc consts --	1.4157	1.7472	1.2689
IR Inten --	39.7459	21.8381	9.6037

	19	20	21
	A	A	A
Frequencies --	1093.3514	1111.4381	1154.5276
Red. masses --	1.6274	2.4073	1.4244
Frc consts --	1.1462	1.7521	1.1187

IR Inten --	2.9214	25.9266	14.8879

	22	23	24
	A	A	A
Frequencies --	1176.9417	1250.4722	1283.2474
Red. masses --	1.4966	1.6319	1.3135
Frc consts --	1.2214	1.5034	1.2744
IR Inten --	3.9584	7.2901	5.0145

	25	26	27
	A	A	A
Frequencies --	1296.4668	1346.0894	1355.7236
Red. masses --	1.3736	1.3119	1.6603
Frc consts --	1.3603	1.4005	1.7980
IR Inten --	4.5628	2.5804	14.0034

	28	29	30
	A	A	A
Frequencies --	1362.6906	1381.3357	1394.1783
Red. masses --	1.3427	1.5325	1.5170
Frc consts --	1.4690	1.7229	1.7373
IR Inten --	7.4850	2.9069	0.6645

	31	32	33
	A	A	A
Frequencies --	1399.5503	1472.4418	1475.9414
Red. masses --	1.4675	1.0954	1.1000
Frc consts --	1.6936	1.3993	1.4118
IR Inten --	2.1848	4.6577	19.1511

	34	35	36
	A	A	A
Frequencies --	1503.8581	1512.9170	1847.6189
Red. masses --	1.0800	1.0957	11.2985
Frc consts --	1.4391	1.4777	22.7246
IR Inten --	7.2674	4.5192	226.1074

	37	38	39
	A	A	A
Frequencies --	3043.3059	3053.9195	3064.2449
Red. masses --	1.0670	1.0641	1.0626
Frc consts --	5.8223	5.8469	5.8785
IR Inten --	23.5005	13.5826	16.8483

	40	41	42
	A	A	A
Frequencies --	3069.3062	3103.7337	3108.2806
Red. masses --	1.0630	1.0968	1.0995
Frc consts --	5.9002	6.2248	6.2586
IR Inten --	9.4677	31.4071	23.7668

	43	44	45
	A	A	A
Frequencies --	3115.6941	3129.4225	3146.8524
Red. masses --	1.1046	1.0887	1.1016
Frc consts --	6.3177	6.2816	6.4276
IR Inten --	29.8743	22.1042	12.0722

21e

	1	2	3
	A	A	A
Frequencies --	93.2051	151.2463	279.5732
Red. masses --	4.9938	2.4730	7.3259
Frc consts --	0.0256	0.0333	0.3374
IR Inten --	1.4524	2.4705	0.9443

	4	5	6
	A	A	A
Frequencies --	312.7370	350.0942	396.7097
Red. masses --	1.8936	3.5198	4.4994
Frc consts --	0.1091	0.2542	0.4172
IR Inten --	0.3189	5.3131	2.2324

```
                      7                      8                      9
                      A                      A                      A
Frequencies --    423.3384               508.8918               602.6018
Red. masses --      2.8835                 2.2938                 4.0539
Frc consts  --      0.3045                 0.3500                 0.8673
IR Inten    --      0.7767                 2.0861                15.9648

                     10                     11                     12
                      A                      A                      A
Frequencies --    679.9319               750.7814               851.1205
Red. masses --      2.9108                 2.1920                 1.7460
Frc consts  --      0.7929                 0.7280                 0.7452
IR Inten    --      2.7447                 2.0866                11.8949

                     13                     14                     15
                      A                      A                      A
Frequencies --    872.0839               900.5492               930.0562
Red. masses --      2.5241                 1.8068                 1.7068
Frc consts  --      1.1310                 0.8634                 0.8698
IR Inten    --      1.2065                22.0001                 8.3587

                     16                     17                     18
                      A                      A                      A
Frequencies --    974.7945              1058.2528              1093.9797
Red. masses --      2.4044                 1.4630                 1.5838
Frc consts  --      1.3461                 0.9653                 1.1168
IR Inten    --      3.2827                 8.5794                 4.8823

                     19                     20                     21
                      A                      A                      A
Frequencies --   1095.6244              1133.4165              1146.4257
Red. masses --      2.4406                 3.3529                 1.8026
Frc consts  --      1.7261                 2.5378                 1.3959
IR Inten    --     23.3070                96.9103                 3.2514

                     22                     23                     24
                      A                      A                      A
Frequencies --   1159.8628              1249.2716              1271.7533
Red. masses --      2.3375                 1.4904                 1.3812
Frc consts  --      1.8528                 1.3704                 1.3162
IR Inten    --      3.3806                 2.7725                 0.5655

                     25                     26                     27
                      A                      A                      A
Frequencies --   1292.1921              1336.9858              1357.8528
Red. masses --      1.2587                 1.2493                 1.3953
Frc consts  --      1.2383                 1.3157                 1.5157
IR Inten    --      0.8489                 4.0276                 4.9165

                     28                     29                     30
                      A                      A                      A
Frequencies --   1363.9084              1377.4663              1398.9474
Red. masses --      1.4134                 1.4985                 1.4595
Frc consts  --      1.5491                 1.6752                 1.6829
IR Inten    --      1.6098                 3.1115                 0.5472


                     31                     32                     33
                      A                      A                      A
Frequencies --   1411.2181              1469.5087              1495.4483
Red. masses --      1.6296                 1.0946                 1.0932
Frc consts  --      1.9122                 1.3926                 1.4405
IR Inten    --      6.3283                11.9048                 5.7368

                     34                     35                     36
                      A                      A                      A
Frequencies --   1499.6685              1511.0876              1859.7425
Red. masses --      1.0846                 1.0979                11.9970
Frc consts  --      1.4371                 1.4771                24.4472
IR Inten    --      9.6696                 3.8949               235.4443

                     37                     38                     39
                      A                      A                      A
Frequencies --   3032.4776              3041.7451              3045.2577
Red. masses --      1.0806                 1.0704                 1.0694
Frc consts  --      5.8545                 5.8348                 5.8430
IR Inten    --      6.9285                19.5341                 8.0023
```

	40	41	42
	A	A	A
Frequencies --	3054.2344	3059.7246	3107.8681
Red. masses --	1.0625	1.0621	1.0980
Frc consts --	5.8394	5.8586	6.2487
IR Inten --	31.4919	27.7931	31.5542

	43	44	45
	A	A	A
Frequencies --	3110.9844	3118.1644	3140.7045
Red. masses --	1.0979	1.1039	1.0968
Frc consts --	6.2604	6.3239	6.3741
IR Inten --	33.4864	27.1040	12.7500

22a

	1	2	3
	A	A	A
Frequencies --	87.7194	151.9116	245.6554
Red. masses --	5.2557	3.4312	3.3726
Frc consts --	0.0238	0.0467	0.1199
IR Inten --	1.5091	1.1024	3.9316

	4	5	6
	A	A	A
Frequencies --	262.5357	343.4632	389.6157
Red. masses --	3.6785	2.8707	2.5829
Frc consts --	0.1494	0.1995	0.2310
IR Inten --	2.0716	0.5339	2.6278

	7	8	9
	A	A	A
Frequencies --	417.9795	473.2700	604.5443
Red. masses --	4.4298	2.1009	4.0720
Frc consts --	0.4560	0.2772	0.8768
IR Inten --	1.6668	1.8255	3.3326

	10	11	12
	A	A	A
Frequencies --	664.6653	745.7642	821.5901
Red. masses --	3.1141	2.6136	1.5635
Frc consts --	0.8106	0.8564	0.6218
IR Inten --	2.7275	3.5922	4.3047

	13	14	15
	A	A	A
Frequencies --	848.2188	891.2723	933.8438
Red. masses --	2.8386	2.2705	1.6024
Frc consts --	1.2033	1.0626	0.8233
IR Inten --	1.5599	6.8506	13.5356

	16	17	18
	A	A	A
Frequencies --	978.0672	1003.1850	1045.6500
Red. masses --	2.6410	2.5565	1.9648
Frc consts --	1.4885	1.5158	1.2658
IR Inten --	19.7097	49.2797	1.4702

	19	20	21
	A	A	A
Frequencies --	1074.5744	1092.6893	1143.5241
Red. masses --	1.9566	1.7712	1.5893
Frc consts --	1.3311	1.2460	1.2244
IR Inten --	39.4770	13.1454	9.7075

	22	23	24
	A	A	A
Frequencies --	1171.6762	1230.4473	1245.5298
Red. masses --	1.6056	1.9360	1.7835
Frc consts --	1.2987	1.7269	1.6302
IR Inten --	5.0324	37.6523	13.9684

	25	26	27
	A	A	A
Frequencies --	1280.1951	1310.7673	1340.8870
Red. masses --	1.3992	1.3474	1.3980
Frc consts --	1.3511	1.3640	1.4810
IR Inten --	12.3421	29.6251	7.5493

	28	29	30
	A	A	A
Frequencies --	1345.5232	1369.1376	1379.1352
Red. masses --	1.4020	1.4140	1.4656
Frc consts --	1.4955	1.5617	1.6424
IR Inten --	3.3604	2.6572	10.0744

	31	32	33
	A	A	A
Frequencies --	1398.0080	1419.7426	1475.1376
Red. masses --	1.5174	1.8015	1.0899
Frc consts --	1.7473	2.1395	1.3973
IR Inten --	3.3049	18.2324	7.2616

	34	35	36
	A	A	A
Frequencies --	1488.5218	1496.5714	1507.5523
Red. masses --	1.1238	1.0785	1.1023
Frc consts --	1.4670	1.4233	1.4760
IR Inten --	11.7946	7.1674	5.9843

	37	38	39
	A	A	A
Frequencies --	3049.6620	3056.4825	3057.8176
Red. masses --	1.0666	1.0632	1.0629
Frc consts --	5.8444	5.8518	5.8558
IR Inten --	15.2673	13.7981	13.1794

	40	41	42
	A	A	A
Frequencies --	3067.2399	3105.7001	3110.4778
Red. masses --	1.0626	1.0988	1.1014
Frc consts --	5.8899	6.2445	6.2784
IR Inten --	21.2615	30.2813	21.1125

	43	44	45
	A	A	A
Frequencies --	3119.0319	3134.2604	3145.8296
Red. masses --	1.1047	1.0890	1.0989
Frc consts --	6.3320	6.3028	6.4076
IR Inten --	31.8467	14.6628	7.3276

22e

	1	2	3
	A	A	A
Frequencies --	81.0435	155.4921	236.9758
Red. masses --	4.5944	2.6544	14.3566
Frc consts --	0.0178	0.0378	0.4750
IR Inten --	0.0224	1.2643	0.5803

	4	5	6
	A	A	A
Frequencies --	292.8982	354.3188	377.3020
Red. masses --	2.0347	3.1127	3.8824
Frc consts --	0.1028	0.2302	0.3256
IR Inten --	0.2174	2.7112	0.9260

	7	8	9
	A	A	A
Frequencies --	415.8603	484.2445	573.9140
Red. masses --	3.0186	2.8198	3.8498
Frc consts --	0.3076	0.3896	0.7471
IR Inten --	0.5354	2.8167	7.3052

```
                      10                     11                     12
                       A                      A                      A
Frequencies --    616.0010               732.3572               831.0794
Red. masses --      2.7503                 3.0386                 1.6327
Frc consts  --      0.6149                 0.9602                 0.6644
IR Inten    --      3.5204                 1.4767                 6.6463

                      13                     14                     15
                       A                      A                      A
Frequencies --    860.2545               886.6189               936.1310
Red. masses --      2.1266                 2.1515                 1.5480
Frc consts  --      0.9273                 0.9965                 0.7993
IR Inten    --      5.8279                 9.4055                 0.4152

                      16                     17                     18
                       A                      A                      A
Frequencies --    966.3479              1050.0905              1072.6170
Red. masses --      2.4115                 1.8752                 1.6408
Frc consts  --      1.3268                 1.2183                 1.1122
IR Inten    --      4.8499                11.4834                 9.1636

                      19                     20                     21
                       A                      A                      A
Frequencies --   1093.8330              1126.7631              1135.8216
Red. masses --      1.6050                 3.9270                 1.8576
Frc consts  --      1.1314                 2.9375                 1.4120
IR Inten    --      1.6133                77.7283                 6.7064

                      22                     23                     24
                       A                      A                      A
Frequencies --   1147.8397              1218.2480              1262.7365
Red. masses --      2.1239                 1.8830                 1.4233
Frc consts  --      1.6487                 1.6465                 1.3372
IR Inten    --      1.7983                12.5001                 0.6266

                      25                     26                     27
                       A                      A                      A
Frequencies --   1280.3754              1320.8613              1331.0770
Red. masses --      1.3674                 1.2954                 1.5851
Frc consts  --      1.3208                 1.3316                 1.6547
IR Inten    --     11.4959                17.9522                13.7009

                      28                     29                     30
                       A                      A                      A
Frequencies --   1353.5033              1364.6671              1385.2015
Red. masses --      1.3985                 1.6191                 1.5186
Frc consts  --      1.5095                 1.7765                 1.7168
IR Inten    --      1.0674                32.3732                15.7362


                      31                     32                     33
                       A                      A                      A
Frequencies --   1397.1571              1404.8496              1486.0996
Red. masses --      1.4935                 1.6672                 1.1023
Frc consts  --      1.7176                 1.9386                 1.4343
IR Inten    --     14.0323                12.9972                 6.5037

                      34                     35                     36
                       A                      A                      A
Frequencies --   1493.9531              1497.4597              1511.4409
Red. masses --      1.0831                 1.0953                 1.0979
Frc consts  --      1.4243                 1.4471                 1.4777
IR Inten    --     12.4310                 5.3530                 5.9355

                      37                     38                     39
                       A                      A                      A
Frequencies --   3028.7192              3037.9997              3044.2821
Red. masses --      1.0753                 1.0750                 1.0694
Frc consts  --      5.8114                 5.8457                 5.8395
IR Inten    --      0.2130                22.1485                23.0354
```

	40	41	42
	A	A	A
Frequencies --	3055.9588	3062.2072	3108.9376
Red. masses --	1.0625	1.0621	1.0950
Frc consts --	5.8461	5.8678	6.2356
IR Inten --	27.4048	20.5982	34.6386

	43	44	45
	A	A	A
Frequencies --	3112.5462	3121.9380	3136.3730
Red. masses --	1.1019	1.1040	1.0960
Frc consts --	6.2898	6.3397	6.3519
IR Inten --	31.4039	26.7830	8.3018

23a

	1	2	3
	A	A	A
Frequencies --	102.2293	154.3367	236.3825
Red. masses --	4.1419	3.1316	4.0852
Frc consts --	0.0255	0.0440	0.1345
IR Inten --	2.3313	0.9641	4.7989

	4	5	6
	A	A	A
Frequencies --	288.2266	373.6395	431.4981
Red. masses --	2.4093	2.0360	3.6161
Frc consts --	0.1179	0.1675	0.3967
IR Inten --	1.8070	1.9472	9.6419

	7	8	9
	A	A	A
Frequencies --	462.9537	488.9661	628.0359
Red. masses --	2.8795	2.3755	3.7097
Frc consts --	0.3636	0.3346	0.8621
IR Inten --	3.2910	10.4879	4.6445

	10	11	12
	A	A	A
Frequencies --	685.9433	773.8041	822.8547
Red. masses --	2.5000	3.0265	1.5346
Frc consts --	0.6931	1.0677	0.6122
IR Inten --	2.5566	4.1812	3.3223

	13	14	15
	A	A	A
Frequencies --	858.7042	894.5700	938.3222
Red. masses --	2.5916	1.9370	1.5905
Frc consts --	1.1259	0.9133	0.8251
IR Inten --	5.4401	4.2845	32.2591

	16	17	18
	A	A	A
Frequencies --	944.5129	997.1094	1017.7946
Red. masses --	1.5779	2.1330	2.4922
Frc consts --	0.8294	1.2495	1.5211
IR Inten --	83.5763	4.3855	22.1710

	19	20	21
	A	A	A
Frequencies --	1054.8422	1090.0120	1113.2561
Red. masses --	1.8967	1.4853	2.3002
Frc consts --	1.2435	1.0398	1.6796
IR Inten --	6.2107	10.5662	20.0546

	22	23	24
	A	A	A
Frequencies --	1144.1147	1183.3956	1199.4266
Red. masses --	1.7394	1.3456	1.2457
Frc consts --	1.3415	1.1102	1.0558
IR Inten --	41.4821	6.5316	17.8021

	25	26	27
	A	A	A
Frequencies --	1276.9723	1294.1017	1342.4920
Red. masses --	1.2915	1.2753	1.2820
Frc consts --	1.2408	1.2584	1.3613

```
 IR Inten    --      4.6582                 3.1183                10.3151



                     28                     29                     30
                      A                      A                      A
 Frequencies --   1348.0474              1368.0323              1374.8781
 Red. masses --      1.3350                 1.5457                 1.3551
 Frc consts  --      1.4293                 1.7044                 1.5092
 IR Inten    --      8.1999                 5.1339                 6.6955

                     31                     32                     33
                      A                      A                      A
 Frequencies --   1393.7469              1396.9406              1417.4003
 Red. masses --      1.5325                 1.4512                 2.0686
 Frc consts  --      1.7539                 1.6685                 2.4486
 IR Inten    --      2.4051                 0.9640                16.2466

                     34                     35                     36
                      A                      A                      A
 Frequencies --   1472.3625              1475.9900              1501.8568
 Red. masses --      1.0896                 1.1229                 1.0812
 Frc consts  --      1.3917                 1.4414                 1.4368
 IR Inten    --      1.0283                22.4003                 7.4678

                     37                     38                     39
                      A                      A                      A
 Frequencies --   1510.9065              1762.6393              3041.0605
 Red. masses --      1.0985                 9.2036                 1.0671
 Frc consts  --      1.4775                16.8475                 5.8145
 IR Inten    --      4.3111                75.4234                23.8691

                     40                     41                     42
                      A                      A                      A
 Frequencies --   3051.8545              3061.4612              3062.4197
 Red. masses --      1.0648                 1.0640                 1.0638
 Frc consts  --      5.8431                 5.8756                 5.8780
 IR Inten    --      9.9588                14.9608                15.6548

                     43                     44                     45
                      A                      A                      A
 Frequencies --   3082.8387              3101.6825              3107.6965
 Red. masses --      1.0874                 1.0969                 1.0988
 Frc consts  --      6.0889                 6.2173                 6.2522
 IR Inten    --     44.8851                35.9137                30.7842

                     46                     47                     48
                      A                      A                      A
 Frequencies --   3113.9388              3142.3086              3473.6881
 Red. masses --      1.1043                 1.0991                 1.0743
 Frc consts  --      6.3088                 6.3940                 7.6379
 IR Inten    --     32.1969                11.8481                 2.3841
```

23e

```
                      1                      2                      3
                      A                      A                      A
 Frequencies --    111.9789               157.9850               304.8343
 Red. masses --      3.9891                 2.4604                 2.5979
 Frc consts  --      0.0295                 0.0362                 0.1422
 IR Inten    --      0.0292                 1.0660                 0.6458

                      4                      5                      6
                      A                      A                      A
 Frequencies --    321.5384               358.4563               395.6937
 Red. masses --      2.6252                 3.9137                 4.5184
 Frc consts  --      0.1599                 0.2963                 0.4168
 IR Inten    --      2.7620                 8.2990                 0.0335

                      7                      8                      9
                      A                      A                      A
 Frequencies --    427.4739               503.6101               594.5605
 Red. masses --      2.8290                 2.2841                 3.6225
 Frc consts  --      0.3046                 0.3413                 0.7545
 IR Inten    --      2.1670                 1.5292                21.7299
```

```
                      10                     11                     12
                       A                      A                      A
Frequencies --    666.2133               767.5230               849.5265
Red. masses --      2.6363                 2.3179                 1.6433
Frc consts  --      0.6894                 0.8045                 0.6988
IR Inten    --      1.1394                 2.7690                15.4671

                      13                     14                     15
                       A                      A                      A
Frequencies --    869.6601               893.4302               910.7659
Red. masses --      2.2694                 1.8332                 1.5298
Frc consts  --      1.0112                 0.8622                 0.7477
IR Inten    --      3.4160                21.7792                28.8342

                      16                     17                     18
                       A                      A                      A
Frequencies --    972.0240               994.0085              1065.2628
Red. masses --      2.4038                 1.2597                 1.6305
Frc consts  --      1.3381                 0.7333                 1.0902
IR Inten    --      2.2113                26.9834                20.0673

                      19                     20                     21
                       A                      A                      A
Frequencies --   1080.5569              1096.7274              1112.5881
Red. masses --      2.3727                 2.0318                 2.4435
Frc consts  --      1.6322                 1.4399                 1.7821
IR Inten    --     30.2245                33.3401                21.5314

                      22                     23                     24
                       A                      A                      A
Frequencies --   1141.3145              1173.5658              1223.8770
Red. masses --      2.1632                 1.4900                 1.3668
Frc consts  --      1.6602                 1.2091                 1.2062
IR Inten    --      8.3687                 2.7116                10.3273

                      25                     26                     27
                       A                      A                      A
Frequencies --   1275.7113              1287.8439              1330.9372
Red. masses --      1.3387                 1.2558                 1.2951
Frc consts  --      1.2836                 1.2271                 1.3516
IR Inten    --      0.8976                 3.9162                20.4211

                      28                     29                     30
                       A                      A                      A
Frequencies --   1345.9184              1367.7638              1372.1819
Red. masses --      1.1877                 1.4086                 1.3960
Frc consts  --      1.2677                 1.5526                 1.5486
IR Inten    --      4.6865                 1.8380                 2.0264

                      31                     32                     33
                       A                      A                      A
Frequencies --   1389.8648              1396.8140              1432.7304
Red. masses --      1.4800                 1.4611                 1.8688
Frc consts  --      1.6845                 1.6796                 2.2601
IR Inten    --     12.8274                 1.6105                29.4420

                      34                     35                     36
                       A                      A                      A
Frequencies --   1476.0809              1495.5402              1499.8887
Red. masses --      1.1064                 1.1047                 1.0871
Frc consts  --      1.4203                 1.4557                 1.4409
IR Inten    --     11.9715                 7.1411                 8.9726

                      37                     38                     39
                       A                      A                      A
Frequencies --   1510.4797              1769.9126              3036.7636
Red. masses --      1.0997                 9.9732                 1.0700
Frc consts  --      1.4782                18.4073                 5.8140
IR Inten    --      4.1809                90.5318                 7.7928

                      40                     41                     42
```

	A	A	A
Frequencies --	3040.4626	3050.3774	3052.6178
Red. masses --	1.0692	1.0792	1.0638
Frc consts --	5.8238	5.9163	5.8408
IR Inten --	17.7743	5.2822	37.4402
	43	44	45
	A	A	A
Frequencies --	3061.3274	3106.4147	3109.0366
Red. masses --	1.0642	1.0966	1.0995
Frc consts --	5.8764	6.2347	6.2619
IR Inten --	33.0093	37.4442	37.7771
	46	47	48
	A	A	A
Frequencies --	3119.4934	3136.8028	3496.3642
Red. masses --	1.1037	1.0956	1.0732
Frc consts --	6.3283	6.3514	7.7296
IR Inten --	31.0128	12.1487	4.5179

24a

	1	2	3
	A	A	A
Frequencies --	109.9164	158.6231	239.6836
Red. masses --	4.2735	3.2338	3.5710
Frc consts --	0.0304	0.0479	0.1209
IR Inten --	2.6824	0.8275	4.1796
	4	5	6
	A	A	A
Frequencies --	288.3635	374.8406	435.1566
Red. masses --	2.4232	2.0733	4.3760
Frc consts --	0.1187	0.1716	0.4882
IR Inten --	3.2005	1.1402	5.0542
	7	8	9
	A	A	A
Frequencies --	462.0431	490.4157	633.3462
Red. masses --	2.7287	2.2320	3.5840
Frc consts --	0.3432	0.3163	0.8470
IR Inten --	4.0167	9.6915	4.9011
	10	11	12
	A	A	A
Frequencies --	690.9434	770.7504	825.9322
Red. masses --	2.6727	2.9798	1.6119
Frc consts --	0.7518	1.0430	0.6478
IR Inten --	1.5624	4.2595	4.6076
	13	14	15
	A	A	A
Frequencies --	856.7602	890.9136	937.0183
Red. masses --	2.4636	1.8151	1.5933
Frc consts --	1.0655	0.8488	0.8242
IR Inten --	0.6872	17.7642	27.7366
	16	17	18
	A	A	A
Frequencies --	942.9602	998.5712	1019.1330
Red. masses --	1.6606	2.0827	2.3485
Frc consts --	0.8700	1.2236	1.4372
IR Inten --	6.2664	27.0361	55.0377
	19	20	21
	A	A	A
Frequencies --	1055.7271	1091.3177	1110.3815
Red. masses --	1.9555	1.5579	2.3806
Frc consts --	1.2842	1.0932	1.7294
IR Inten --	15.4762	10.9088	33.8744
	22	23	24
	A	A	A
Frequencies --	1142.4284	1182.0057	1199.1776
Red. masses --	1.5177	1.4186	1.2359
Frc consts --	1.1670	1.1677	1.0471
IR Inten --	11.0975	5.2891	16.3925

	25	26	27
	A	A	A
Frequencies --	1275.1769	1291.5037	1341.6571
Red. masses --	1.3041	1.2698	1.3270
Frc consts --	1.2494	1.2479	1.4073
IR Inten --	0.1744	1.8186	5.2705
	28	29	30
	A	A	A
Frequencies --	1350.0977	1366.8441	1375.7456
Red. masses --	1.2711	1.3978	1.4548
Frc consts --	1.3651	1.5386	1.6223
IR Inten --	5.5528	4.8541	11.6825
	31	32	33
	A	A	A
Frequencies --	1394.6548	1398.9966	1423.4997
Red. masses --	1.5100	1.5043	2.2570
Frc consts --	1.7304	1.7347	2.6946
IR Inten --	0.3808	2.4575	51.6569
	34	35	36
	A	A	A
Frequencies --	1475.1525	1479.4848	1500.5805
Red. masses --	1.0954	1.1000	1.0824
Frc consts --	1.4044	1.4186	1.4360
IR Inten --	5.6069	18.8746	5.4271
	37	38	39
	A	A	A
Frequencies --	1509.6665	1765.3810	3038.8933
Red. masses --	1.0972	9.1955	1.0673
Frc consts --	1.4734	16.8851	5.8073
IR Inten --	4.7913	77.6767	25.2520
	40	41	42
	A	A	A
Frequencies --	3054.7613	3057.8610	3063.5115
Red. masses --	1.0643	1.0604	1.0622
Frc consts --	5.8513	5.8417	5.8733
IR Inten --	15.0053	13.3310	22.0608
	43	44	45
	A	A	A
Frequencies --	3101.2730	3107.8642	3112.2916
Red. masses --	1.0957	1.1003	1.1039
Frc consts --	6.2088	6.2616	6.3001
IR Inten --	40.2332	34.7514	2.2707
	46	47	48
	A	A	A
Frequencies --	3116.0573	3132.8632	3481.4947
Red. masses --	1.1047	1.0888	1.0743
Frc consts --	6.3196	6.2961	7.6720
IR Inten --	43.5598	17.9907	2.0663

24e

	1	2	3
	A	A	A
Frequencies --	102.0738	162.1378	293.0035
Red. masses --	4.2699	2.4860	5.3646
Frc consts --	0.0262	0.0385	0.2714
IR Inten --	0.2432	2.5019	0.1391
	4	5	6
	A	A	A
Frequencies --	314.1878	352.9295	397.1884
Red. masses --	1.9214	3.2782	4.3289
Frc consts --	0.1118	0.2406	0.4024
IR Inten --	0.1748	0.5865	4.4733

```
                        7                      8                      9
                        A                      A                      A
Frequencies --    427.6658               505.5153               589.4419
Red. masses --      2.7477                 2.2677                 3.3519
Frc consts  --      0.2961                 0.3414                 0.6862
IR Inten    --      0.3070                 8.1994                15.9129

                       10                     11                     12
                        A                      A                      A
Frequencies --    666.7807               775.2390               850.2402
Red. masses --      2.7042                 2.2612                 1.7237
Frc consts  --      0.7084                 0.8007                 0.7341
IR Inten    --      1.3544                 0.1758                 6.5786

                       13                     14                     15
                        A                      A                      A
Frequencies --    871.8657               891.3823               896.6487
Red. masses --      1.9587                 1.4824                 2.0854
Frc consts  --      0.8773                 0.6940                 0.9878
IR Inten    --     12.3844                19.2527                15.4247

                       16                     17                     18
                        A                      A                      A
Frequencies --    973.3765               984.1552              1066.8363
Red. masses --      2.3790                 1.3227                 1.5514
Frc consts  --      1.3280                 0.7548                 1.0403
IR Inten    --      9.5682                24.5108                 7.3104

                       19                     20                     21
                        A                      A                      A
Frequencies --   1075.6575              1098.9611              1131.1607
Red. masses --      2.0215                 1.8560                 3.5892
Frc consts  --      1.3781                 1.3206                 2.7058
IR Inten    --     20.0352                 4.1101                96.1245

                       22                     23                     24
                        A                      A                      A
Frequencies --   1134.3699              1170.9461              1206.3218
Red. masses --      2.1237                 1.4963                 1.3017
Frc consts  --      1.6101                 1.2088                 1.1161
IR Inten    --     14.9923                 8.7179                18.0724

                       25                     26                     27
                        A                      A                      A
Frequencies --   1275.3707              1287.7914              1330.8083
Red. masses --      1.3500                 1.2583                 1.3207
Frc consts  --      1.2938                 1.2295                 1.3781
IR Inten    --      0.7721                 0.9796                14.4260

                       28                     29                     30
                        A                      A                      A
Frequencies --   1351.4233              1364.8196              1369.6283
Red. masses --      1.2095                 1.3837                 1.4288
Frc consts  --      1.3015                 1.5186                 1.5792
IR Inten    --     14.7744                 3.9533                 3.4134

                       31                     32                     33
                        A                      A                      A
Frequencies --   1386.2318              1398.3239              1437.2838
Red. masses --      1.5834                 1.4552                 1.8312
Frc consts  --      1.7927                 1.6765                 2.2288
IR Inten    --      9.0816                 0.7750                10.1940

                       34                     35                     36
                        A                      A                      A
Frequencies --   1483.1308              1494.1882              1500.9386
Red. masses --      1.0977                 1.0991                 1.0880
Frc consts  --      1.4226                 1.4457                 1.4441
IR Inten    --     13.6806                 4.4684                10.5558

                       37                     38                     39
```

	A	A	A
Frequencies --	1511.2789	1770.5138	3026.6578
Red. masses --	1.1004	9.4826	1.0808
Frc consts --	1.4808	17.5136	5.8333
IR Inten --	3.1172	86.0018	9.4553

	40	41	42
	A	A	A
Frequencies --	3038.5622	3040.7658	3052.1237
Red. masses --	1.0678	1.0696	1.0621
Frc consts --	5.8087	5.8269	5.8296
IR Inten --	20.4422	22.1571	28.2967

	43	44	45
	A	A	A
Frequencies --	3060.3880	3104.9475	3106.9177
Red. masses --	1.0624	1.0971	1.0972
Frc consts --	5.8626	6.2318	6.2404
IR Inten --	24.1670	35.6548	38.6215

	46	47	48
	A	A	A
Frequencies --	3110.9806	3119.9819	3489.5566
Red. masses --	1.1009	1.1035	1.0745
Frc consts --	6.2775	6.3289	7.7091
IR Inten --	25.6621	32.5364	2.2741

25a

	1	2	3
	A	A	A
Frequencies --	114.0818	161.2919	239.1231
Red. masses --	3.4428	3.0615	3.4207
Frc consts --	0.0264	0.0469	0.1152
IR Inten --	0.6467	1.8057	3.4074

	4	5	6
	A	A	A
Frequencies --	289.5739	373.8027	404.0799
Red. masses --	2.3298	2.0081	2.5044
Frc consts --	0.1151	0.1653	0.2409
IR Inten --	1.2353	1.7555	0.8809

	7	8	9
	A	A	A
Frequencies --	456.6925	474.7704	630.7319
Red. masses --	3.3429	2.0430	4.0891
Frc consts --	0.4108	0.2713	0.9585
IR Inten --	2.0668	3.0091	2.0731

	10	11	12
	A	A	A
Frequencies --	665.0632	743.7750	784.4801
Red. masses --	2.2857	1.2327	2.3212
Frc consts --	0.5957	0.4018	0.8416
IR Inten --	6.8090	1.2405	2.2673

	13	14	15
	A	A	A
Frequencies --	827.1020	867.1159	895.6638
Red. masses --	1.6784	2.4645	2.1773
Frc consts --	0.6765	1.0918	1.0291
IR Inten --	4.8890	2.9239	12.8923

	16	17	18
	A	A	A
Frequencies --	925.0929	962.4695	981.5992
Red. masses --	1.4897	1.3928	2.4502
Frc consts --	0.7511	0.7602	1.3910
IR Inten --	20.9892	27.6528	50.7964

	19	20	21
	A	A	A
Frequencies --	1006.0698	1014.4067	1060.0956
Red. masses --	1.5486	2.7430	1.8653
Frc consts --	0.9235	1.6630	1.2351
IR Inten --	14.5558	28.2220	4.6479

```
                      22                     23                     24
                       A                      A                      A
 Frequencies --   1102.5290              1117.8357              1184.1525
 Red. masses --      1.7111                 1.8303                 1.2635
 Frc consts  --      1.2254                 1.3475                 1.0439
 IR Inten    --     11.0452                 8.8665                11.5846

                      25                     26                     27
                       A                      A                      A
 Frequencies --   1190.9744              1277.1755              1295.0062
 Red. masses --      1.4995                 1.3541                 1.2435
 Frc consts  --      1.2531                 1.3014                 1.2287
 IR Inten    --      4.1667                 0.1323                 1.5958

                      28                     29                     30
                       A                      A                      A
 Frequencies --   1323.7411              1346.3096              1363.2667
 Red. masses --      1.5650                 1.2502                 1.3470
 Frc consts  --      1.6158                 1.3351                 1.4749
 IR Inten    --      2.9227                 7.0448                 5.3636

                      31                     32                     33
                       A                      A                      A
 Frequencies --   1375.1300              1382.4243              1396.6860
 Red. masses --      1.6105                 1.6157                 1.5364
 Frc consts  --      1.7943                 1.8192                 1.7659
 IR Inten    --      4.1457                 4.5701                 1.5617

                      34                     35                     36
                       A                      A                      A
 Frequencies --   1398.9316              1465.1725              1474.0142
 Red. masses --      1.6183                 1.1621                 1.0965
 Frc consts  --      1.8660                 1.4698                 1.4037
 IR Inten    --      0.5707                 6.6788                 8.5476

                      37                     38                     39
                       A                      A                      A
 Frequencies --   1487.0279              1499.0609              1509.4389
 Red. masses --      1.1228                 1.0832                 1.0958
 Frc consts  --      1.4629                 1.4342                 1.4710
 IR Inten    --     16.1860                 6.6687                 4.0773

                      40                     41                     42
                       A                      A                      A
 Frequencies --   1748.6808              3035.5996              3051.0937
 Red. masses --      5.9596                 1.0670                 1.0630
 Frc consts  --     10.7372                 5.7932                 5.8304
 IR Inten    --     11.9865                26.3319                16.7427


                      43                     44                     45
                       A                      A                      A
 Frequencies --   3052.5558              3059.6897              3094.6732
 Red. masses --      1.0639                 1.0624                 1.0887
 Frc consts  --      5.8407                 5.8602                 6.1432
 IR Inten    --     14.2919                22.3382                63.6511

                      46                     47                     48
                       A                      A                      A
 Frequencies --   3096.9608              3104.4292              3110.5234
 Red. masses --      1.0957                 1.0997                 1.1012
 Frc consts  --      6.1915                 6.2445                 6.2772
 IR Inten    --     20.6773                47.1315                 2.5457

                      49                     50                     51
                       A                      A                      A
 Frequencies --   3112.7719              3145.6809              3232.2725
 Red. masses --      1.1035                 1.0616                 1.1162
 Frc consts  --      6.2999                 6.1892                 6.8710
 IR Inten    --     51.9840                 8.7889                12.8270
```

25e

```
                       1                      2                      3
                       A                      A                      A
 Frequencies --    114.8073               171.0738               294.4932
 Red. masses --      3.3337                 2.4394                 3.4184
```

```
Frc consts  --      0.0259                 0.0421                 0.1747
IR Inten    --      1.1628                 0.4184                 2.0913



                      4                      5                      6
                      A                      A                      A
Frequencies --    312.9655               355.2174               390.6705
Red. masses --      1.9932                 3.1155                 3.6243
Frc consts  --      0.1150                 0.2316                 0.3259
IR Inten    --      0.9210                 1.6835                 1.2224

                      7                      8                      9
                      A                      A                      A
Frequencies --    426.0272               482.5561               566.3530
Red. masses --      2.5722                 2.4846                 2.9425
Frc consts  --      0.2751                 0.3409                 0.5561
IR Inten    --      0.4742                 2.3544                 9.8770

                     10                     11                     12
                      A                      A                      A
Frequencies --    641.4912               749.9727               789.3686
Red. masses --      2.3796                 1.1675                 2.0173
Frc consts  --      0.5769                 0.3869                 0.7406
IR Inten    --      6.0737                 0.2346                 1.0649

                     13                     14                     15
                      A                      A                      A
Frequencies --    844.9504               878.5069               879.8423
Red. masses --      1.6832                 2.1063                 1.9481
Frc consts  --      0.7080                 0.9578                 0.8885
IR Inten    --      8.0904                 3.4869                 9.0312

                     16                     17                     18
                      A                      A                      A
Frequencies --    954.2103               958.7318               966.3581
Red. masses --      1.7790                 1.3690                 1.8413
Frc consts  --      0.9544                 0.7414                 1.0131
IR Inten    --      5.2317                43.0575                 9.4344

                     19                     20                     21
                      A                      A                      A
Frequencies --   1008.0048              1070.0492              1094.9440
Red. masses --      1.6148                 1.7652                 3.6347
Frc consts  --      0.9667                 1.1908                 2.5675
IR Inten    --      5.8077                12.9809                76.3105

                     22                     23                     24
                      A                      A                      A
Frequencies --   1098.7330              1124.4088              1171.9819
Red. masses --      2.0042                 1.7278                 1.5341
Frc consts  --      1.4255                 1.2870                 1.2415
IR Inten    --      3.1328                 4.0481                 2.4991

                     25                     26                     27
                      A                      A                      A
Frequencies --   1186.2302              1276.9733              1279.8233
Red. masses --      1.5568                 1.4074                 1.3839
Frc consts  --      1.2907                 1.3522                 1.3355
IR Inten    --      1.0424                 0.3211                 2.5950

                     28                     29                     30
                      A                      A                      A
Frequencies --   1294.8532              1343.9998              1365.1830
Red. masses --      1.3547                 1.2104                 1.3891
Frc consts  --      1.3383                 1.2882                 1.5253
IR Inten    --      0.1347                 0.1923                 1.1265

                     31                     32                     33
                      A                      A                      A
Frequencies --   1371.6328              1385.0016              1396.1376
Red. masses --      1.4603                 1.5439                 1.4535
Frc consts  --      1.6187                 1.7449                 1.6693
IR Inten    --      1.2176                 2.6647                 0.1168

                     34                     35                     36
```

```
                      A                      A                      A
Frequencies --   1410.3041              1455.9635              1488.0481
Red. masses --      1.6681                 1.1673                 1.1080
Frc consts  --      1.9548                 1.4579                 1.4455
IR Inten    --     14.0130                 5.8322                10.2704

                     37                     38                     39
                      A                      A                      A
Frequencies --   1492.7502              1499.3677              1509.9190
Red. masses --      1.0997                 1.0863                 1.0995
Frc consts  --      1.4437                 1.4389                 1.4769
IR Inten    --      6.4779                10.3503                 3.3999

                     40                     41                     42
                      A                      A                      A
Frequencies --   1751.9798              3024.5846              3033.0365
Red. masses --      6.2121                 1.0702                 1.0798
Frc consts  --     11.2343                 5.7685                 5.8524
IR Inten    --     17.1252                 9.7443                21.6268

                     43                     44                     45
                      A                      A                      A
Frequencies --   3036.8166              3048.6274              3058.8846
Red. masses --      1.0702                 1.0621                 1.0619
Frc consts  --      5.8149                 5.8157                 5.8542
IR Inten    --     37.8454                29.6426                24.1336

                     46                     47                     48
                      A                      A                      A
Frequencies --   3100.0809              3102.3070              3107.3487
Red. masses --      1.0954                 1.0989                 1.0984
Frc consts  --      6.2023                 6.2314                 6.2488
IR Inten    --     45.1766                48.1851                21.7139

                     49                     50                     51
                      A                      A                      A
Frequencies --   3116.5491              3162.2109              3258.5360
Red. masses --      1.1042                 1.0618                 1.1155
Frc consts  --      6.3190                 6.2554                 6.9783
IR Inten    --     40.3702                 8.3538                 4.5577
```

Vibrational frequencies (implicit DMSO)

1a

```
                      1                      2                      3
                      A                      A                      A
 Frequencies --     79.4945               158.0562               227.3005
 Red. masses --      6.0240                 3.3364                 4.8661
 Frc consts  --      0.0224                 0.0491                 0.1481
 IR Inten    --     16.8227                 1.3962                 7.4769

                      4                      5                      6
                      A                      A                      A
 Frequencies --    291.9247               400.5551               435.7659
 Red. masses --      4.0419                 2.1720                 4.8323
 Frc consts  --      0.2029                 0.2053                 0.5406
 IR Inten    --      4.7004                13.4629                 3.4851

                      7                      8                      9
                      A                      A                      A
 Frequencies --    477.5911               507.6887               651.1146
 Red. masses --      5.7165                 2.2605                 3.9490
 Frc consts  --      0.7682                 0.3433                 0.9864
 IR Inten    --     29.2819                 7.8584                26.2191

                     10                     11                     12
                      A                      A                      A
 Frequencies --    747.0564               790.6997               868.8744
 Red. masses --      4.0451                 2.4781                 2.8697
 Frc consts  --      1.3301                 0.9128                 1.2764
 IR Inten    --     14.9574                30.1885                23.0007

                     13                     14                     15
                      A                      A                      A
 Frequencies --    888.0910               943.7780              1012.3503
 Red. masses --      2.5009                 2.1586                 3.3404
 Frc consts  --      1.1622                 1.1328                 2.0170
 IR Inten    --     41.6439                28.6458                 2.9358

                     16                     17                     18
                      A                      A                      A
 Frequencies --   1027.1320              1053.6446              1105.5931
 Red. masses --      2.4238                 2.0278                 2.4963
 Frc consts  --      1.5066                 1.3264                 1.7978
 IR Inten    --    112.9408                26.2755                29.6881

                     19                     20                     21
                      A                      A                      A
 Frequencies --   1149.2298              1207.8180              1257.1782
 Red. masses --      4.4413                 1.4234                 1.7581
 Frc consts  --      3.4560                 1.2234                 1.6372
 IR Inten    --     74.9094                22.2429               140.8987

                     22                     23                     24
                      A                      A                      A
 Frequencies --   1268.6505              1315.8987              1339.3320
 Red. masses --      1.4698                 1.4806                 1.6554
 Frc consts  --      1.3938                 1.5105                 1.7496
 IR Inten    --      4.1214                20.8499                17.3923

                     25                     26                     27
                      A                      A                      A
 Frequencies --   1351.1199              1368.6698              1397.2699
 Red. masses --      1.2866                 1.2134                 1.3925
 Frc consts  --      1.3838                 1.3392                 1.6018
 IR Inten    --      7.6562                23.2642                12.4812

                     28                     29                     30
                      A                      A                      A
 Frequencies --   1422.2167              1445.9309              1473.5060
 Red. masses --      1.3548                 1.1149                 1.0856
 Frc consts  --      1.6145                 1.3733                 1.3888
```

IR Inten --	24.3831	23.1945	12.1167
	31	32	33
	A	A	A
Frequencies --	1511.8945	1825.5921	3017.6694
Red. masses --	1.0919	11.3060	1.0699
Frc consts --	1.4706	22.2007	5.7401
IR Inten --	8.0888	420.1456	50.8208
	34	35	36
	A	A	A
Frequencies --	3025.3454	3076.7079	3132.2814
Red. masses --	1.0706	1.0626	1.0886
Frc consts --	5.7732	5.9266	6.2927
IR Inten --	70.4699	5.6768	22.6191
	37	38	39
	A	A	A
Frequencies --	3144.5651	3149.5949	3154.5480
Red. masses --	1.0947	1.0955	1.1027
Frc consts --	6.3780	6.4031	6.4651
IR Inten --	27.4441	22.8260	10.6193

1e

	1	2	3
	A	A	A
Frequencies --	96.0657	153.0802	278.5646
Red. masses --	5.5544	2.5132	8.1429
Frc consts --	0.0302	0.0347	0.3723
IR Inten --	6.5510	12.0572	2.0516
	4	5	6
	A	A	A
Frequencies --	343.4734	353.0514	409.2507
Red. masses --	3.2453	3.6455	4.5702
Frc consts --	0.2256	0.2677	0.4510
IR Inten --	6.8448	31.0038	2.1453
	7	8	9
	A	A	A
Frequencies --	447.9679	520.9053	607.4852
Red. masses --	3.0650	3.1684	4.5958
Frc consts --	0.3624	0.5065	0.9993
IR Inten --	1.5758	10.5264	26.9936
	10	11	12
	A	A	A
Frequencies --	717.2358	766.5935	877.1134
Red. masses --	2.8945	2.5167	2.5177
Frc consts --	0.8773	0.8714	1.1412
IR Inten --	21.6542	7.1658	27.3569
	13	14	15
	A	A	A
Frequencies --	914.8942	996.9423	1003.7645
Red. masses --	2.7425	1.4088	2.7885
Frc consts --	1.3525	0.8250	1.6553
IR Inten --	35.6661	7.4953	61.4134
	16	17	18
	A	A	A
Frequencies --	1073.9311	1104.1337	1123.1127
Red. masses --	3.6582	3.6955	2.7751
Frc consts --	2.4858	2.6544	2.0624
IR Inten --	35.9414	134.2966	0.3164
	19	20	21
	A	A	A
Frequencies --	1148.2358	1187.7875	1247.3114
Red. masses --	4.2494	1.7370	1.6651
Frc consts --	3.3010	1.4439	1.5263
IR Inten --	94.0156	38.9158	113.9691
	22	23	24
	A	A	A
Frequencies --	1266.7460	1313.8000	1331.7185

```
 Red. masses --      1.3405                 1.4156                 1.2602
 Frc consts  --      1.2674                 1.4396                 1.3168
 IR Inten    --      3.2607                12.6376                15.2376




                      25                     26                     27
                       A                      A                      A
 Frequencies --   1355.5282              1363.5649              1409.9666
 Red. masses --      1.2756                 1.3203                 1.4077
 Frc consts  --      1.3809                 1.4463                 1.6489
 IR Inten    --      6.0955                 8.0283                20.4205

                      28                     29                     30
                       A                      A                      A
 Frequencies --   1430.3507              1452.8447              1497.7867
 Red. masses --      1.4510                 1.1047                 1.0761
 Frc consts  --      1.7491                 1.3738                 1.4223
 IR Inten    --      8.0244                20.2919                 3.3856

                      31                     32                     33
                       A                      A                      A
 Frequencies --   1511.0539              1824.7785              3024.0332
 Red. masses --      1.0960                11.9475                 1.0695
 Frc consts  --      1.4744                23.4394                 5.7622
 IR Inten    --     10.4168               450.2216                58.7916

                      34                     35                     36
                       A                      A                      A
 Frequencies --   3029.2083              3072.3680              3089.9463
 Red. masses --      1.0703                 1.0633                 1.0831
 Frc consts  --      5.7865                 5.9136                 6.0929
 IR Inten    --     63.9080                 4.9091                21.5320

                      37                     38                     39
                       A                      A                      A
 Frequencies --   3146.3785              3150.1677              3152.8525
 Red. masses --      1.0993                 1.0987                 1.0975
 Frc consts  --      6.4117                 6.4237                 6.4277
 IR Inten    --     23.7838                19.1117                21.6996
```

2a

```
                       1                      2                      3
                       A                      A                      A
 Frequencies --     77.0683               157.4106               238.2260
 Red. masses --      6.3929                 3.8677                 4.1114
 Frc consts  --      0.0224                 0.0565                 0.1375
 IR Inten    --      6.6618                 0.1397                11.6816

                       4                      5                      6
                       A                      A                      A
 Frequencies --    268.7886               350.6183               406.3305
 Red. masses --      5.6464                 3.8148                 2.7447
 Frc consts  --      0.2403                 0.2763                 0.2670
 IR Inten    --      4.0960                 3.8823                14.4729

                       7                      8                      9
                       A                      A                      A
 Frequencies --    417.2906               503.2886               633.2173
 Red. masses --      3.6777                 2.6218                 5.2349
 Frc consts  --      0.3773                 0.3913                 1.2367
 IR Inten    --      2.0469                 2.4330                10.6308

                      10                     11                     12
                       A                      A                      A
 Frequencies --    704.4912               778.0639               865.4249
 Red. masses --      4.3268                 2.2804                 3.0416
 Frc consts  --      1.2652                 0.8134                 1.3422
 IR Inten    --     40.3416                34.5498                17.3652

                      13                     14                     15
                       A                      A                      A
 Frequencies --    877.9695               933.0409              1004.4689
```

Red. masses --	2.7226	2.2329	3.2475
Frc consts --	1.2365	1.1453	1.9305
IR Inten --	47.2310	35.2458	13.2787

	16	17	18
	A	A	A
Frequencies --	1021.3217	1046.4565	1080.8860
Red. masses --	2.8390	1.8137	2.3403
Frc consts --	1.7448	1.1702	1.6109
IR Inten --	161.5318	13.8234	91.9415

	19	20	21
	A	A	A
Frequencies --	1147.1870	1178.8926	1236.3048
Red. masses --	4.2410	1.8256	3.1057
Frc consts --	3.2885	1.4948	2.7968
IR Inten --	98.9431	36.2636	63.7813

	22	23	24
	A	A	A
Frequencies --	1257.6626	1267.2299	1319.2820
Red. masses --	1.4798	1.3676	1.4920
Frc consts --	1.3790	1.2940	1.5300
IR Inten --	44.5807	50.4486	99.0491

	25	26	27
	A	A	A
Frequencies --	1338.7622	1339.8592	1384.0888
Red. masses --	1.3895	1.1781	1.5807
Frc consts --	1.4673	1.2460	1.7841
IR Inten --	54.9981	6.0047	80.5779

	28	29	30
	A	A	A
Frequencies --	1389.6948	1430.4507	1464.0051
Red. masses --	1.3122	1.4322	1.1768
Frc consts --	1.4931	1.7266	1.4861
IR Inten --	15.5587	3.1708	15.3890

	31	32	33
	A	A	A
Frequencies --	1476.6226	1509.1106	3021.7064
Red. masses --	1.0836	1.0954	1.0698
Frc consts --	1.3921	1.4698	5.7552
IR Inten --	10.7375	10.9877	81.3611

	34	35	36
	A	A	A
Frequencies --	3023.8332	3068.9413	3138.8638
Red. masses --	1.0694	1.0639	1.0900
Frc consts --	5.7610	5.9039	6.3274
IR Inten --	34.7163	6.0275	11.6079

	37	38	39
	A	A	A
Frequencies --	3143.9051	3152.7961	3155.5606
Red. masses --	1.0947	1.0955	1.1016
Frc consts --	6.3752	6.4157	6.4627
IR Inten --	27.9931	30.1294	6.2503

2e

	1	2	3
	A	A	A
Frequencies --	81.6613	157.4221	237.2004
Red. masses --	5.5898	2.6604	13.9630
Frc consts --	0.0220	0.0388	0.4629
IR Inten --	2.0097	6.9103	1.3304

	4	5	6
	A	A	A
Frequencies --	316.6825	357.3404	385.5046

```
Red. masses --      3.6689                 4.3233                 2.7674
Frc consts  --      0.2168                 0.3253                 0.2423
IR Inten    --     10.4746                21.3036                 5.9102

                      7                      8                      9
                      A                      A                      A
Frequencies --    446.3850               486.4910               576.3928
Red. masses --      3.2973                 4.3492                 4.4775
Frc consts  --      0.3871                 0.6065                 0.8764
IR Inten    --      1.3850                 4.5731                 7.3746

                     10                     11                     12
                      A                      A                      A
Frequencies --    657.3603               743.1863               845.6887
Red. masses --      3.2092                 3.1182                 2.1341
Frc consts  --      0.8171                 1.0147                 0.8992
IR Inten    --     49.6104                 4.3124                17.7408

                     13                     14                     15
                      A                      A                      A
Frequencies --    909.9734               994.4687              1011.8297
Red. masses --      2.6468                 3.0367                 1.4609
Frc consts  --      1.2913                 1.7694                 0.8813
IR Inten    --     12.0412               106.1039                 3.8508

                     16                     17                     18
                      A                      A                      A
Frequencies --   1049.4814              1087.3826              1105.5302
Red. masses --      2.9701                 2.6697                 3.6700
Frc consts  --      1.9274                 1.8598                 2.6428
IR Inten    --      8.1727                67.5493                70.8348

                     19                     20                     21
                      A                      A                      A
Frequencies --   1143.0319              1185.1618              1209.0216
Red. masses --      4.5282                 1.8177                 2.0762
Frc consts  --      3.4857                 1.5043                 1.7881
IR Inten    --    128.8777                22.8133                57.4202

                     22                     23                     24
                      A                      A                      A
Frequencies --   1246.6490              1293.7883              1322.3411
Red. masses --      1.6357                 1.4908                 1.2657
Frc consts  --      1.4978                 1.4703                 1.3039
IR Inten    --     70.3085                 2.0699                 5.3562

                     25                     26                     27
                      A                      A                      A
Frequencies --   1334.0231              1353.6567              1363.0382
Red. masses --      1.6420                 1.4369                 1.2867
Frc consts  --      1.7217                 1.5513                 1.4085
IR Inten    --    191.1923                73.2026                35.5175

                     28                     29                     30
                      A                      A                      A
Frequencies --   1404.7529              1430.1850              1464.2058
Red. masses --      1.3793                 1.4237                 1.1292
Frc consts  --      1.6036                 1.7157                 1.4264
IR Inten    --      5.1334                11.5003                19.8845

                     31                     32                     33
                      A                      A                      A
Frequencies --   1500.3780              1514.4317              3023.4198
Red. masses --      1.0765                 1.0974                 1.0689
Frc consts  --      1.4278                 1.4830                 5.7567
IR Inten    --      2.0727                12.5126                53.5424

                     34                     35                     36
                      A                      A                      A
Frequencies --   3031.5789              3059.0887              3084.8334
Red. masses --      1.0700                 1.0663                 1.0829
Frc consts  --      5.7941                 5.8791                 6.0715
```

```
 IR Inten    --     63.1543                 8.2726                22.4081
                      37                     38                     39
                       A                      A                      A
 Frequencies --   3152.0085              3153.1485              3155.0605
 Red. masses --      1.0964                 1.0981                 1.0980
 Frc consts  --      6.4181                 6.4324                 6.4398
 IR Inten    --     37.1768                 0.7833                25.2041
```

3a

```
                       1                      2                      3
                       A                      A                      A
 Frequencies --     99.8112               163.2068               235.0889
 Red. masses --      4.6909                 3.3997                 4.7495
 Frc consts  --      0.0275                 0.0534                 0.1547
 IR Inten    --      8.3974                 0.3335                 6.4185
                       4                      5                      6
                       A                      A                      A
 Frequencies --    296.6955               401.1395               435.8214
 Red. masses --      3.5929                 2.1003                 3.6516
 Frc consts  --      0.1863                 0.1991                 0.4086
 IR Inten    --      8.0381                17.5252                12.7803
                       7                      8                      9
                       A                      A                      A
 Frequencies --    472.5644               506.5008               652.6620
 Red. masses --      5.7043                 2.1319                 4.2068
 Frc consts  --      0.7505                 0.3222                 1.0558
 IR Inten    --     15.3261                10.0375                10.5717
                      10                     11                     12
                       A                      A                      A
 Frequencies --    740.7283               794.8065               870.2047
 Red. masses --      3.2955                 2.5932                 2.7726
 Frc consts  --      1.0653                 0.9652                 1.2370
 IR Inten    --     55.7398                17.3025                22.1792
                      13                     14                     15
                       A                      A                      A
 Frequencies --    884.9251               929.5587               953.7319
 Red. masses --      2.2192                 1.9014                 1.6627
 Frc consts  --      1.0239                 0.9680                 0.8911
 IR Inten    --     15.3316               189.1231                15.3603
                      16                     17                     18
                       A                      A                      A
 Frequencies --   1013.3056              1040.7517              1069.8466
 Red. masses --      3.1743                 2.3020                 1.9440
 Frc consts  --      1.9204                 1.4691                 1.3109
 IR Inten    --      1.7858                82.7634                21.9195
                      19                     20                     21
                       A                      A                      A
 Frequencies --   1105.4503              1145.1895              1173.3113
 Red. masses --      2.1939                 1.9414                 3.0207
 Frc consts  --      1.5796                 1.5001                 2.4501
 IR Inten    --     28.6513                73.2838               142.5948
                      22                     23                     24
                       A                      A                      A
 Frequencies --   1224.8545              1267.4248              1319.9065
 Red. masses --      1.2176                 1.2850                 1.4347
 Frc consts  --      1.0763                 1.2161                 1.4727
 IR Inten    --      7.3456                59.8282                28.0045
                      25                     26                     27
                       A                      A                      A
 Frequencies --   1342.7703              1351.4117              1368.5121
 Red. masses --      1.3005                 1.5100                 1.2358
 Frc consts  --      1.3815                 1.6248                 1.3637
 IR Inten    --     11.6430                 5.0235                19.3892
                      28                     29                     30
                       A                      A                      A
 Frequencies --   1380.6786              1420.4460              1426.0076
 Red. masses --      1.2581                 1.4439                 1.6702
 Frc consts  --      1.4131                 1.7165                 2.0011
```

```
 IR Inten    --     11.0580                42.1574                 4.6937
                     31                     32                     33
                      A                      A                      A
 Frequencies --   1455.4020              1472.5076              1507.6991
 Red. masses --      1.1651                 1.0883                 1.0947
 Frc consts  --      1.4541                 1.3903                 1.4662
 IR Inten    --     24.3388                13.3455                 8.0365
                     34                     35                     36
                      A                      A                      A
 Frequencies --   1758.5030              3015.1378              3016.7502
 Red. masses --      9.2322                 1.0698                 1.0707
 Frc consts  --     16.8206                 5.7302                 5.7412
 IR Inten    --    149.6740                64.0131                65.0820
                     37                     38                     39
                      A                      A                      A
 Frequencies --   3074.2510              3108.2259              3141.9282
 Red. masses --      1.0630                 1.0881                 1.0946
 Frc consts  --      5.9191                 6.1935                 6.3666
 IR Inten    --     12.6609                33.8412                42.6912
                     40                     41                     42
                      A                      A                      A
 Frequencies --   3144.3543              3146.4246              3488.3164
 Red. masses --      1.0961                 1.1015                 1.0746
 Frc consts  --      6.3849                 6.4249                 7.7041
 IR Inten    --     15.2713                20.3859                 1.6574
```

3e

```
                      1                      2                      3
                      A                      A                      A
 Frequencies --    109.5353               160.9863               284.7147
 Red. masses --      4.4183                 2.5086                 5.1862
 Frc consts  --      0.0312                 0.0383                 0.2477
 IR Inten    --      1.5034                 7.2834                 8.9916
                      4                      5                      6
                      A                      A                      A
 Frequencies --    343.9930               356.1399               406.2585
 Red. masses --      3.2547                 3.6099                 4.0868
 Frc consts  --      0.2269                 0.2698                 0.3974
 IR Inten    --      7.2830                40.6053                 1.2605
                      7                      8                      9
                      A                      A                      A
 Frequencies --    448.9761               511.2871               597.5293
 Red. masses --      2.8932                 3.1026                 4.2191
 Frc consts  --      0.3436                 0.4779                 0.8875
 IR Inten    --      1.0801                13.6105                22.7144
                     10                     11                     12
                      A                      A                      A
 Frequencies --    705.7978               778.9827               876.4179
 Red. masses --      2.5990                 2.5902                 2.2643
 Frc consts  --      0.7628                 0.9261                 1.0247
 IR Inten    --     23.6167                 5.5977                18.6059
                     13                     14                     15
                      A                      A                      A
 Frequencies --    902.0984               922.0369              1001.8429
 Red. masses --      2.2729                 1.3011                 2.8655
 Frc consts  --      1.0898                 0.6517                 1.6945
 IR Inten    --     71.5415                64.8809                50.4336
                     16                     17                     18
                      A                      A                      A
 Frequencies --   1045.5023              1065.0801              1093.2857
 Red. masses --      1.6533                 2.7360                 2.8550
 Frc consts  --      1.0648                 1.8287                 2.0106
 IR Inten    --     15.5645                86.5472               101.5334
                     19                     20                     21
                      A                      A                      A
 Frequencies --   1121.1862              1136.3538              1172.2605
 Red. masses --      2.7275                 2.4904                 3.5795
```

Frc consts --	2.0201	1.8947	2.8982
IR Inten --	7.0215	47.0075	83.7718

	22	23	24
	A	A	A
Frequencies --	1228.0429	1262.5189	1320.0974
Red. masses --	1.1906	1.3043	1.3733
Frc consts --	1.0579	1.2249	1.4100
IR Inten --	18.3891	46.3116	9.7949

	25	26	27
	A	A	A
Frequencies --	1328.2203	1336.4024	1366.1241
Red. masses --	1.3124	1.1922	1.2913
Frc consts --	1.3642	1.2545	1.4199
IR Inten --	51.5863	6.2226	4.9212

	28	29	30
	A	A	A
Frequencies --	1380.8808	1419.6457	1437.5498
Red. masses --	1.3409	1.4136	1.6700
Frc consts --	1.5065	1.6786	2.0334
IR Inten --	4.5309	50.3582	4.0590

	31	32	33
	A	A	A
Frequencies --	1458.5289	1497.2984	1510.3250
Red. masses --	1.1439	1.0809	1.0976
Frc consts --	1.4338	1.4278	1.4751
IR Inten --	21.8034	3.4245	10.9777

	34	35	36
	A	A	A
Frequencies --	1757.8249	3019.8009	3020.3873
Red. masses --	10.0146	1.0691	1.0703
Frc consts --	18.2319	5.7443	5.7526
IR Inten --	190.5423	52.5090	78.0685

	37	38	39
	A	A	A
Frequencies --	3069.0784	3097.4421	3141.9144
Red. masses --	1.0639	1.0843	1.0969
Frc consts --	5.9043	6.1290	6.3799
IR Inten --	12.6212	24.4317	35.2811

	40	41	42
	A	A	A
Frequencies --	3144.5029	3148.0286	3490.7463
Red. masses --	1.1000	1.0973	1.0736
Frc consts --	6.4083	6.4070	7.7076
IR Inten --	19.3244	28.4208	9.0377

4a

	1	2	3
	A	A	A
Frequencies --	101.8050	167.3342	232.0004
Red. masses --	4.7422	3.6192	4.2057
Frc consts --	0.0290	0.0597	0.1334
IR Inten --	9.4985	1.8451	10.0434

	4	5	6
	A	A	A
Frequencies --	300.3188	403.5836	435.1130
Red. masses --	3.5539	2.1471	4.2143
Frc consts --	0.1889	0.2060	0.4701
IR Inten --	3.2769	10.9876	8.0638

	7	8	9
	A	A	A
Frequencies --	469.4509	507.5457	656.4325
Red. masses --	4.4655	2.2074	4.1648
Frc consts --	0.5798	0.3350	1.0574
IR Inten --	26.6796	2.4608	11.6847

	10	11	12
	A	A	A

```
Frequencies --    746.2480               791.1427               870.5095
Red. masses --      3.9072                 2.5039                 2.9732
Frc consts  --      1.2820                 0.9234                 1.3274
IR Inten    --     28.2318                23.8764                19.0793

                    13                     14                     15
                     A                      A                      A
Frequencies --    884.5445               927.1296               956.0215
Red. masses --      2.1817                 1.7225                 1.6677
Frc consts  --      1.0057                 0.8724                 0.8981
IR Inten    --     77.3333                16.9608                61.2163

                    16                     17                     18
                     A                      A                      A
Frequencies --   1011.4785              1038.0373              1070.6042
Red. masses --      3.2169                 2.2901                 1.9671
Frc consts  --      1.9391                 1.4539                 1.3284
IR Inten    --      4.8052                97.5194               106.3472

                    19                     20                     21
                     A                      A                      A
Frequencies --   1106.0048              1147.7440              1172.2330
Red. masses --      2.1998                 1.8465                 3.2635
Frc consts  --      1.5854                 1.4332                 2.6422
IR Inten    --     14.2747                37.0209               138.5160

                    22                     23                     24
                     A                      A                      A
Frequencies --   1230.2664              1271.4659              1324.7132
Red. masses --      1.1922                 1.2815                 1.4329
Frc consts  --      1.0632                 1.2206                 1.4815
IR Inten    --      1.8927                56.9637                17.1558

                    25                     26                     27
                     A                      A                      A
Frequencies --   1336.7668              1360.5347              1369.9986
Red. masses --      1.4057                 1.3319                 1.2124
Frc consts  --      1.4800                 1.4526                 1.3407
IR Inten    --      0.6969                 3.5729                25.8632

                    28                     29                     30
                     A                      A                      A
Frequencies --   1380.8307              1425.8338              1430.4721
Red. masses --      1.3585                 1.4163                 1.8862
Frc consts  --      1.5262                 1.6965                 2.2740
IR Inten    --     28.7902                 2.9126                85.7123

                    31                     32                     33
                     A                      A                      A
Frequencies --   1455.1739              1476.2377              1512.5778
Red. masses --      1.1047                 1.0874                 1.0946
Frc consts  --      1.3782                 1.3962                 1.4755
IR Inten    --     20.4463                11.8912                 8.9561

                    34                     35                     36
                     A                      A                      A
Frequencies --   1759.8949              3016.1532              3017.9874
Red. masses --      9.2676                 1.0700                 1.0699
Frc consts  --     16.9119                 5.7353                 5.7416
IR Inten    --    150.9799                92.7695                37.3490

                    37                     38                     39
                     A                      A                      A
Frequencies --   3073.7094              3131.4806              3134.5867
Red. masses --      1.0611                 1.0892                 1.1010
Frc consts  --      5.9064                 6.2933                 6.3740
IR Inten    --     11.4218                28.7845                22.4253

                    40                     41                     42
                     A                      A                      A
Frequencies --   3140.1603              3142.4764              3491.0827
Red. masses --      1.0976                 1.0956                 1.0745
Frc consts  --      6.3765                 6.3745                 7.7157
```

```
 IR Inten    --    33.5082                20.5044                 2.1083

4e                   1                      2                      3
                     A                      A                      A
 Frequencies --    98.6630               160.4623               291.0184
 Red. masses --     4.6627                 2.5180                 5.8033
 Frc consts  --     0.0267                 0.0382                 0.2896
 IR Inten    --     3.5612                11.2115                 0.9366

                     4                      5                      6
                     A                      A                      A
 Frequencies --   345.5015               354.1084               408.5985
 Red. masses --     3.2195                 3.5037                 3.9340
 Frc consts  --     0.2264                 0.2589                 0.3870
 IR Inten    --     7.7935                18.4816                 6.2703

                     7                      8                      9
                     A                      A                      A
 Frequencies --   447.4889               513.6473               594.3130
 Red. masses --     2.9166                 3.1278                 4.0282
 Frc consts  --     0.3441                 0.4862                 0.8383
 IR Inten    --     2.8146                 9.5795                28.7183

                    10                     11                     12
                     A                      A                      A
 Frequencies --   704.1221               785.0283               875.8357
 Red. masses --     2.6120                 2.5492                 2.6025
 Frc consts  --     0.7630                 0.9256                 1.1762
 IR Inten    --    27.9038                 1.7645                28.2390

                    13                     14                     15
                     A                      A                      A
 Frequencies --   893.9234               919.1432              1001.7659
 Red. masses --     1.4836                 1.8125                 2.8022
 Frc consts  --     0.6985                 0.9022                 1.6568
 IR Inten    --    41.8581                34.8385                90.7313

                    16                     17                     18
                     A                      A                      A
 Frequencies --  1045.9081              1073.7472              1097.5521
 Red. masses --     1.6356                 2.6209                 3.4675
 Frc consts  --     1.0542                 1.7803                 2.4610
 IR Inten    --    23.3291                 8.6173               156.5402

                    19                     20                     21
                     A                      A                      A
 Frequencies --  1118.5380              1133.7133              1171.5322
 Red. masses --     2.6660                 2.2542                 3.7070
 Frc consts  --     1.9652                 1.7071                 2.9977
 IR Inten    --    13.1365                81.3524               117.2048

                    22                     23                     24
                     A                      A                      A
 Frequencies --  1223.1716              1262.6604              1320.0202
 Red. masses --     1.1559                 1.3187                 1.3912
 Frc consts  --     1.0189                 1.2387                 1.4283
 IR Inten    --     4.0245                57.0062                20.6767

                    25                     26                     27
                     A                      A                      A
 Frequencies --  1336.3742              1341.2995              1364.5964
 Red. masses --     1.2431                 1.2818                 1.2695
 Frc consts  --     1.3080                 1.3587                 1.3928
 IR Inten    --     7.0200                33.3142                 7.2800

                    28                     29                     30
                     A                      A                      A
 Frequencies --  1383.6046              1420.4753              1443.6339
 Red. masses --     1.3243                 1.3976                 1.7491
 Frc consts  --     1.4937                 1.6615                 2.1477
 IR Inten    --     6.8634                11.3868                19.8664

                    31                     32                     33
                     A                      A                      A
 Frequencies --  1460.2211              1497.4478              1510.9516
 Red. masses --     1.1183                 1.0817                 1.0983
 Frc consts  --     1.4049                 1.4291                 1.4774
```

```
 IR Inten    --     25.2677                 2.7195                11.3667
                      34                     35                     36
                       A                      A                      A
 Frequencies --   1759.9695              3017.8159              3021.0551
 Red. masses --      9.5298                 1.0693                 1.0702
 Frc consts  --     17.3917                 5.7375                 5.7549
 IR Inten    --    185.4009                60.9777                70.0532
                      37                     38                     39
                       A                      A                      A
 Frequencies --   3067.5556              3085.2366              3131.1063
 Red. masses --      1.0623                 1.0836                 1.1025
 Frc consts  --      5.8897                 6.0772                 6.3684
 IR Inten    --      8.9248                30.7736                24.4242
                      40                     41                     42
                       A                      A                      A
 Frequencies --   3144.2190              3148.1631              3501.4604
 Red. masses --      1.0965                 1.0971                 1.0746
 Frc consts  --      6.3869                 6.4062                 7.7622
 IR Inten    --     34.6227                28.4378                 2.9390
```

5a

```
                       1                      2                      3
                       A                      A                      A
 Frequencies --    107.0120               168.0789               233.6731
 Red. masses --      3.6967                 3.6044                 3.9326
 Frc consts  --      0.0249                 0.0600                 0.1265
 IR Inten    --      1.5446                 0.5051                10.5770
                       4                      5                      6
                       A                      A                      A
 Frequencies --    298.6678               383.8461               415.8988
 Red. masses --      3.1409                 2.3188                 2.1306
 Frc consts  --      0.1651                 0.2013                 0.2171
 IR Inten    --      3.9748                 7.2049                 8.4312
                       7                      8                      9
                       A                      A                      A
 Frequencies --    459.7700               498.5154               643.0518
 Red. masses --      5.2436                 2.2547                 4.1543
 Frc consts  --      0.6531                 0.3301                 1.0121
 IR Inten    --      8.5805                 1.5122                 4.9291
                      10                     11                     12
                       A                      A                      A
 Frequencies --    724.7031               761.5314               800.3644
 Red. masses --      1.9989                 1.4966                 2.3610
 Frc consts  --      0.6185                 0.5114                 0.8911
 IR Inten    --     51.7862                20.3961                10.1861
                      13                     14                     15
                       A                      A                      A
 Frequencies --    866.3554               884.9859               926.6468
 Red. masses --      2.7922                 3.0486                 1.9618
 Frc consts  --      1.2348                 1.4068                 0.9925
 IR Inten    --     25.0173                84.7469                35.5508
                      16                     17                     18
                       A                      A                      A
 Frequencies --    975.2587              1004.7511              1013.0059
 Red. masses --      1.3530                 2.6199                 1.7880
 Frc consts  --      0.7582                 1.5583                 1.0810
 IR Inten    --     69.5267                18.7772                 4.6254
                      19                     20                     21
                       A                      A                      A
 Frequencies --   1034.1583              1075.6197              1102.6694
 Red. masses --      2.4385                 1.8401                 2.0404
 Frc consts  --      1.5366                 1.2543                 1.4617
 IR Inten    --    126.9543                13.5186                17.8635
                      22                     23                     24
                       A                      A                      A
 Frequencies --   1166.2236              1233.5903              1272.0451
```

```
Red. masses --      5.1705                1.2156                1.3271
Frc consts  --      4.1433                1.0899                1.2652
IR Inten    --    148.7730                6.3671               48.1791



                     25                    26                    27
                      A                     A                     A
Frequencies --   1313.7589             1327.7607             1352.5728
Red. masses --      1.6379                1.3398                1.5352
Frc consts  --      1.6656                1.3917                1.6548
IR Inten    --      2.1213                9.1154               10.5048

                     28                    29                    30
                      A                     A                     A
Frequencies --   1364.8050             1369.1793             1400.6401
Red. masses --      1.2031                1.5575                1.4076
Frc consts  --      1.3203                1.7203                1.6270
IR Inten    --     25.8463                6.2515                2.7711

                     31                    32                    33
                      A                     A                     A
Frequencies --   1426.3973             1455.5140             1471.8745
Red. masses --      1.3801                1.1318                1.1475
Frc consts  --      1.6544                1.4127                1.4646
IR Inten    --     17.3029               11.5055               16.2684

                     34                    35                    36
                      A                     A                     A
Frequencies --   1475.0941             1505.7399             1746.9086
Red. masses --      1.0992                1.0965                6.0796
Frc consts  --      1.4091                1.4647               10.9311
IR Inten    --     17.7933                8.6856               19.3233

                     37                    38                    39
                      A                     A                     A
Frequencies --   3009.8359             3014.2177             3063.9246
Red. masses --      1.0703                1.0695                1.0630
Frc consts  --      5.7128                5.7253                5.8793
IR Inten    --     86.9126               55.7030               27.6723

                     40                    41                    42
                      A                     A                     A
Frequencies --   3110.7838             3125.9346             3134.9737
Red. masses --      1.0879                1.1006                1.0957
Frc consts  --      6.2027                6.3364                6.3446
IR Inten    --     38.8195               33.4073               49.7235

                     43                    44                    45
                      A                     A                     A
Frequencies --   3137.5700             3146.9124             3236.6683
Red. masses --      1.0956                1.0622                1.1168
Frc consts  --      6.3544                6.1977                6.8930
IR Inten    --     30.8727                9.8604               14.1555
```

5e

```
                      1                     2                     3
                      A                     A                     A
Frequencies --    112.0832              171.6266              286.0770
Red. masses --      3.6431                2.5145                3.9348
Frc consts  --      0.0270                0.0436                0.1897
IR Inten    --      0.2087                4.6937                6.2019

                      4                     5                     6
                      A                     A                     A
Frequencies --    338.0123              355.2970              396.3061
Red. masses --      2.9819                3.5806                2.9162
Frc consts  --      0.2007                0.2663                0.2699
IR Inten    --     14.1189               18.5370                2.8629

                      7                     8                     9
                      A                     A                     A
Frequencies --    447.7333              484.9141              573.0979
```

```
 Red. masses --      2.7773                 3.4034                 3.7912
 Frc consts  --      0.3280                 0.4715                 0.7336
 IR Inten    --      0.6001                 3.4823                11.0716




                      10                     11                     12
                       A                      A                      A
 Frequencies --    681.3230               750.2469               800.8316
 Red. masses --      2.3345                 1.1517                 2.2781
 Frc consts  --      0.6385                 0.3820                 0.8608
 IR Inten    --     39.8130                 0.2416                 0.5023

                      13                     14                     15
                       A                      A                      A
 Frequencies --    869.7201               894.1062               953.4400
 Red. masses --      2.1897                 2.3473                 1.3481
 Frc consts  --      0.9759                 1.1056                 0.7220
 IR Inten    --     12.4736                19.5551                82.3703

                      16                     17                     18
                       A                      A                      A
 Frequencies --    981.0173              1018.0532              1047.7886
 Red. masses --      2.2883                 1.9107                 1.6630
 Frc consts  --      1.2975                 1.1667                 1.0757
 IR Inten    --     61.9782                27.1610                 2.5531

                      19                     20                     21
                       A                      A                      A
 Frequencies --   1071.9773              1097.6033              1123.1930
 Red. masses --      4.5758                 2.5307                 2.4401
 Frc consts  --      3.0981                 1.7963                 1.8137
 IR Inten    --    163.0206                 7.6866                13.5786

                      22                     23                     24
                       A                      A                      A
 Frequencies --   1158.7977              1218.2073              1258.5775
 Red. masses --      5.3194                 1.2955                 1.3895
 Frc consts  --      4.2085                 1.1327                 1.2968
 IR Inten    --    120.7813                 3.5825                43.9613

                      25                     26                     27
                       A                      A                      A
 Frequencies --   1286.1253              1322.6004              1334.9999
 Red. masses --      1.5192                 1.3481                 1.2018
 Frc consts  --      1.4805                 1.3894                 1.2619
 IR Inten    --      4.1732                 8.7605                 6.2964

                      28                     29                     30
                       A                      A                      A
 Frequencies --   1364.5190              1379.5211              1405.7865
 Red. masses --      1.3066                 1.4418                 1.4570
 Frc consts  --      1.4334                 1.6166                 1.6965
 IR Inten    --      3.5368                 8.4359                 5.6605

                      31                     32                     33
                       A                      A                      A
 Frequencies --   1424.1131              1447.4724              1468.7630
 Red. masses --      1.4005                 1.1568                 1.1358
 Frc consts  --      1.6735                 1.4279                 1.4436
 IR Inten    --     13.7569                 6.4750                22.7389

                      34                     35                     36
                       A                      A                      A
 Frequencies --   1496.1005              1509.3304              1746.5805
 Red. masses --      1.0802                 1.0987                 6.3666
 Frc consts  --      1.4245                 1.4746                11.4429
 IR Inten    --      1.4928                11.7379                37.5385

                      37                     38                     39
                       A                      A                      A
 Frequencies --   3013.0594              3014.6722              3055.4536
 Red. masses --      1.0699                 1.0683                 1.0645
 Frc consts  --      5.7226                 5.7203                 5.8555
```

```
 IR Inten    --     64.6240                78.8439                28.8708

                      40                     41                     42
                       A                      A                      A
 Frequencies --   3077.2834              3125.2066              3133.9569
 Red. masses --      1.0841                 1.0993                 1.0967
 Frc consts  --      6.0485                 6.3258                 6.3462
 IR Inten    --     45.9745                35.6795                41.1437

                      43                     44                     45
                       A                      A                      A
 Frequencies --   3136.0147              3160.3895              3253.7217
 Red. masses --      1.0975                 1.0614                 1.1165
 Frc consts  --      6.3591                 6.2464                 6.9644
 IR Inten    --     44.7123                 8.1905                 7.6362
```

6a

```
                       1                      2                      3
                       A                      A                      A
 Frequencies --     72.0032               147.8959               202.9739
 Red. masses --      7.0805                 4.7381                 4.6261
 Frc consts  --      0.0216                 0.0611                 0.1123
 IR Inten    --     17.5717                 0.6560                 2.4925

                       4                      5                      6
                       A                      A                      A
 Frequencies --    249.2078               323.5439               385.8771
 Red. masses --      3.0080                 2.6325                 6.1761
 Frc consts  --      0.1101                 0.1624                 0.5418
 IR Inten    --      8.7334                 3.1766                 5.9381

                       7                      8                      9
                       A                      A                      A
 Frequencies --    415.6996               499.0087               615.0839
 Red. masses --      4.3352                 2.9506                 4.1273
 Frc consts  --      0.4414                 0.4329                 0.9200
 IR Inten    --      6.7529                24.4700                18.5256

                      10                     11                     12
                       A                      A                      A
 Frequencies --    668.3859               721.5615               729.7600
 Red. masses --      4.6742                 4.9091                 4.0751
 Frc consts  --      1.2303                 1.5059                 1.2786
 IR Inten    --     13.3734                 2.7742                 3.3563

                      13                     14                     15
                       A                      A                      A
 Frequencies --    775.5375               835.2100               951.4171
 Red. masses --      2.3295                 1.8184                 2.6496
 Frc consts  --      0.8255                 0.7474                 1.4131
 IR Inten    --      8.8132                 9.7725                 5.0926

                      16                     17                     18
                       A                      A                      A
 Frequencies --    976.6431              1001.8880              1010.1219
 Red. masses --      2.1806                 2.5628                 1.9784
 Frc consts  --      1.2254                 1.5157                 1.1893
 IR Inten    --     37.9459                24.9952                16.2620

                      19                     20                     21
                       A                      A                      A
 Frequencies --   1061.4708              1153.5456              1163.4875
 Red. masses --      3.0453                 1.5673                 1.1667
 Frc consts  --      2.0216                 1.2288                 0.9306
 IR Inten    --    111.6282                29.8958                 1.1388

                      22                     23                     24
                       A                      A                      A
 Frequencies --   1237.4183              1282.3497              1294.6721
 Red. masses --      1.1646                 1.5476                 1.4653
 Frc consts  --      1.0507                 1.4995                 1.4471
 IR Inten    --      1.3688                 9.5243                 8.8565

                      25                     26                     27
                       A                      A                      A
 Frequencies --   1323.6721              1352.8194              1360.1949
```

```
 Red. masses --      1.3285                 1.3924                 1.4118
 Frc consts  --      1.3715                 1.5013                 1.5389
 IR Inten    --     18.3466                35.5941                30.3651
```

```
                       28                     29                     30
                        A                      A                      A
 Frequencies --   1362.6722              1449.8213              1456.0626
 Red. masses --      1.5958                 1.0883                 1.1065
 Frc consts  --      1.7458                 1.3478                 1.3822
 IR Inten    --      9.4631                12.5752                29.4656
                       31                     32                     33
                        A                      A                      A
 Frequencies --   1474.7511              1819.9459              3080.8850
 Red. masses --      1.0855                11.2249                 1.0640
 Frc consts  --      1.3909                21.9054                 5.9503
 IR Inten    --     11.7522               420.6176                17.2436
                       34                     35                     36
                        A                      A                      A
 Frequencies --   3083.6950              3087.2656              3127.6249
 Red. masses --      1.0598                 1.0617                 1.0875
 Frc consts  --      5.9375                 5.9622                 6.2679
 IR Inten    --     14.8469                16.3846                26.5957
                       37                     38                     39
                        A                      A                      A
 Frequencies --   3147.3983              3153.1010              3154.9382
 Red. masses --      1.1032                 1.1040                 1.1045
 Frc consts  --      6.4388                 6.4670                 6.4773
 IR Inten    --     11.4190                 6.0289                 4.1985
```

6e

```
                        1                      2                      3
                        A                      A                      A
 Frequencies --     84.4209               154.0399               253.2386
 Red. masses --      6.8026                 2.9333                 2.5959
 Frc consts  --      0.0286                 0.0410                 0.0981
 IR Inten    --      9.9764                 9.6789                 2.7791
                        4                      5                      6
                        A                      A                      A
 Frequencies --    295.0869               341.4988               345.9978
 Red. masses --      6.4477                 3.7657                 6.7551
 Frc consts  --      0.3308                 0.2587                 0.4765
 IR Inten    --      1.1516                12.6681                 2.1358
                        7                      8                      9
                        A                      A                      A
 Frequencies --    367.4065               467.6097               587.4874
 Red. masses --      6.4209                 2.7011                 5.1140
 Frc consts  --      0.5107                 0.3480                 1.0399
 IR Inten    --      5.3539                 1.4348                30.6477
                       10                     11                     12
                        A                      A                      A
 Frequencies --    686.9113               702.0723               762.8583
 Red. masses --      3.7807                 3.5787                 2.4945
 Frc consts  --      1.0511                 1.0393                 0.8553
 IR Inten    --      6.4858                 7.4304                 1.4550
                       13                     14                     15
                        A                      A                      A
 Frequencies --    789.1984               849.5363               921.3827
 Red. masses --      4.3048                 1.9921                 1.5605
 Frc consts  --      1.5797                 0.8471                 0.7805
 IR Inten    --     12.9439                40.3951                14.3409
                       16                     17                     18
                        A                      A                      A
 Frequencies --    996.7474              1041.9005              1059.3886
```

```
 Red. masses --      2.3637                 2.2877                 4.2232
 Frc consts  --      1.3836                 1.4632                 2.7926
 IR Inten    --     27.6302                12.9867               105.2869




                      19                     20                     21
                       A                      A                      A
 Frequencies --   1085.6651              1132.7350              1151.0608
 Red. masses --      2.4634                 1.3266                 1.8171
 Frc consts  --      1.7107                 1.0029                 1.4185
 IR Inten    --     76.9728                23.0638                17.9351

                      22                     23                     24
                       A                      A                      A
 Frequencies --   1220.2875              1265.8944              1287.2731
 Red. masses --      1.2898                 1.3407                 1.2969
 Frc consts  --      1.1316                 1.2659                 1.2662
 IR Inten    --      4.2830                 4.7765                 8.0481

                      25                     26                     27
                       A                      A                      A
 Frequencies --   1327.9089              1352.0814              1359.2273
 Red. masses --      1.2225                 1.3975                 1.4411
 Frc consts  --      1.2701                 1.5052                 1.5687
 IR Inten    --     21.9901                14.2571                 5.4772

                      28                     29                     30
                       A                      A                      A
 Frequencies --   1395.6778              1454.2615              1476.3675
 Red. masses --      1.5376                 1.0942                 1.0970
 Frc consts  --      1.7647                 1.3634                 1.4088
 IR Inten    --      2.6286                21.2161                 7.8704

                      31                     32                     33
                       A                      A                      A
 Frequencies --   1483.7023              1824.3227              3070.0086
 Red. masses --      1.0862                11.9285                 1.0623
 Frc consts  --      1.4088                23.3904                 5.8989
 IR Inten    --     14.0651               450.7478                 7.1058

                      34                     35                     36
                       A                      A                      A
 Frequencies --   3079.1446              3083.1534              3089.6731
 Red. masses --      1.0635                 1.0658                 1.0768
 Frc consts  --      5.9408                 5.9690                 6.0564
 IR Inten    --     17.1844                12.7780                26.1974

                      37                     38                     39
                       A                      A                      A
 Frequencies --   3135.7522              3147.4708              3154.4583
 Red. masses --      1.1018                 1.1056                 1.1057
 Frc consts  --      6.3830                 6.4530                 6.4824
 IR Inten    --     15.2822                 7.4769                 3.6755
```

7a

```
                        1                      2                      3
                        A                      A                      A
 Frequencies --     67.1821               146.1682               200.7949
 Red. masses --      7.9245                 5.0307                 4.2255
 Frc consts  --      0.0211                 0.0633                 0.1004
 IR Inten    --      7.2911                 0.4524                 2.5402

                        4                      5                      6
                        A                      A                      A
 Frequencies --    244.1094               317.7835               344.0834
 Red. masses --      4.1919                 2.7819                 7.1240
 Frc consts  --      0.1472                 0.1655                 0.4969
 IR Inten    --     10.6645                 4.8781                 0.3880

                        7                      8                      9
                        A                      A                      A
 Frequencies --    383.9294               438.1561               592.3502
```

Red. masses --	4.2184	2.5857	5.5834
Frc consts --	0.3664	0.2925	1.1543
IR Inten --	5.5674	1.3024	11.6627

	10	11	12
	A	A	A
Frequencies --	667.6530	680.3170	710.6886
Red. masses --	4.3041	5.1051	3.9564
Frc consts --	1.1304	1.3921	1.1774
IR Inten --	20.3030	7.2621	2.2804
	13	14	15
	A	A	A
Frequencies --	764.7230	841.7730	936.9637
Red. masses --	2.4952	1.8129	2.8526
Frc consts --	0.8597	0.7569	1.4755
IR Inten --	5.3715	13.8548	3.7434
	16	17	18
	A	A	A
Frequencies --	960.2620	986.6050	1007.1334
Red. masses --	2.1232	2.2730	2.2442
Frc consts --	1.1535	1.3036	1.3412
IR Inten --	19.4749	26.1248	71.7547
	19	20	21
	A	A	A
Frequencies --	1040.6986	1132.8079	1149.9126
Red. masses --	3.5567	1.4435	1.4385
Frc consts --	2.2696	1.0914	1.1207
IR Inten --	174.3686	9.6967	17.6724
	22	23	24
	A	A	A
Frequencies --	1217.0353	1248.0017	1266.9240
Red. masses --	1.4906	1.5936	1.5027
Frc consts --	1.3008	1.4624	1.4211
IR Inten --	45.6117	119.7291	8.3118
	25	26	27
	A	A	A
Frequencies --	1298.7531	1314.9923	1336.8800
Red. masses --	1.5058	1.2393	1.6376
Frc consts --	1.4965	1.2626	1.7244
IR Inten --	56.2792	20.8142	15.2663
	28	29	30
	A	A	A
Frequencies --	1353.1027	1405.9083	1452.7980
Red. masses --	1.2041	1.7301	1.0846
Frc consts --	1.2989	2.0149	1.3487
IR Inten --	12.4823	40.9194	14.5946
	31	32	33
	A	A	A
Frequencies --	1468.5054	1474.1216	3071.3902
Red. masses --	1.0870	1.1327	1.0635
Frc consts --	1.3812	1.4502	5.9109
IR Inten --	22.2545	10.9286	8.3868
	34	35	36
	A	A	A
Frequencies --	3077.9046	3083.5570	3132.6917
Red. masses --	1.0615	1.0623	1.0879
Frc consts --	5.9246	5.9511	6.2904
IR Inten --	21.3962	15.8447	16.1900
	37	38	39
	A	A	A
Frequencies --	3144.3164	3149.4829	3153.8525
Red. masses --	1.1014	1.1050	1.1035
Frc consts --	6.4158	6.4581	6.4668

```
 IR Inten    --      8.9700                 7.0316                 2.8685
```

7e

```
                        1                      2                      3
                        A                      A                      A
 Frequencies --     69.4268               154.2965               240.5783
 Red. masses --      7.4601                 3.0963                 3.2035
 Frc consts  --      0.0212                 0.0434                 0.1092
 IR Inten    --      3.0363                 6.3863                 5.3540

                        4                      5                      6
                        A                      A                      A
 Frequencies --    245.6976               322.8389               343.5864
 Red. masses --      9.6311                 4.5315                 4.1292
 Frc consts  --      0.3426                 0.2783                 0.2872
 IR Inten    --      2.6007                 0.3923                 9.7071

                        7                      8                      9
                        A                      A                      A
 Frequencies --    370.6517               436.1545               551.3342
 Red. masses --      6.1068                 2.7217                 5.0470
 Frc consts  --      0.4943                 0.3050                 0.9039
 IR Inten    --      3.3353                 0.8914                12.5072

                       10                     11                     12
                        A                      A                      A
 Frequencies --    624.7191               690.0380               732.9669
 Red. masses --      4.3734                 3.9411                 2.8346
 Frc consts  --      1.0056                 1.1056                 0.8972
 IR Inten    --     17.4909                 7.3146                 1.5993

                       13                     14                     15
                        A                      A                      A
 Frequencies --    790.0481               830.5534               936.2074
 Red. masses --      4.5845                 1.7277                 1.6164
 Frc consts  --      1.6860                 0.7022                 0.8347
 IR Inten    --     15.2924                17.6660                15.6896

                       16                     17                     18
                        A                      A                      A
 Frequencies --    982.8267              1002.4030              1047.6395
 Red. masses --      2.5629                 2.2674                 3.0128
 Frc consts  --      1.4586                 1.3424                 1.9483
 IR Inten    --     12.0281                55.3031                 8.3677

                       19                     20                     21
                        A                      A                      A
 Frequencies --   1071.8835              1128.1114              1142.0242
 Red. masses --      4.0021                 1.3238                 1.6944
 Frc consts  --      2.7091                 0.9926                 1.3020
 IR Inten    --    143.1611                26.0771                 2.9278

                       22                     23                     24
                        A                      A                      A
 Frequencies --   1188.0549              1233.4015              1272.3322
 Red. masses --      1.5181                 1.5897                 1.2457
 Frc consts  --      1.2625                 1.4248                 1.1881
 IR Inten    --     14.2379                62.5050                 7.5260

                       25                     26                     27
                        A                      A                      A
 Frequencies --   1302.6027              1324.4051              1330.0364
 Red. masses --      1.5943                 1.4420                 1.2752
 Frc consts  --      1.5939                 1.4902                 1.3291
 IR Inten    --    120.4759                10.3046                 6.0505

                       28                     29                     30
                        A                      A                      A
 Frequencies --   1377.7954              1380.9390              1471.0502
 Red. masses --      1.4301                 1.7172                 1.0840
```

Frc consts --	1.5995	1.9294	1.3821
IR Inten --	13.8161	72.1903	4.2613
	31	32	33
	A	A	A
Frequencies --	1475.1969	1480.2130	3063.9196
Red. masses --	1.1035	1.0974	1.0640
Frc consts --	1.4149	1.4166	5.8848
IR Inten --	29.3127	8.0852	6.0296
	34	35	36
	A	A	A
Frequencies --	3082.7581	3087.6908	3091.1023
Red. masses --	1.0727	1.0673	1.0658
Frc consts --	6.0064	5.9951	6.0001
IR Inten --	0.8313	28.1855	27.5192
	37	38	39
	A	A	A
Frequencies --	3137.1255	3156.7102	3160.9736
Red. masses --	1.1007	1.1052	1.1053
Frc consts --	6.3826	6.4886	6.5069
IR Inten --	11.3888	7.7559	2.9719

8a

	1	2	3
	A	A	A
Frequencies --	82.0928	148.7560	208.2145
Red. masses --	5.0032	4.7945	4.3575
Frc consts --	0.0199	0.0625	0.1113
IR Inten --	8.3910	0.2987	2.2036
	4	5	6
	A	A	A
Frequencies --	257.7576	325.7414	386.5937
Red. masses --	2.8583	2.6835	5.2686
Frc consts --	0.1119	0.1678	0.4639
IR Inten --	8.7451	3.3879	4.2417
	7	8	9
	A	A	A
Frequencies --	413.2399	491.0041	622.8270
Red. masses --	3.6997	2.5128	4.2485
Frc consts --	0.3722	0.3569	0.9710
IR Inten --	17.0552	23.4002	9.7586
	10	11	12
	A	A	A
Frequencies --	667.8810	710.8739	722.5647
Red. masses --	4.7140	3.3785	5.1881
Frc consts --	1.2389	1.0059	1.5959
IR Inten --	12.9791	13.0199	5.6335
	13	14	15
	A	A	A
Frequencies --	780.1139	835.8122	925.7057
Red. masses --	2.3690	1.8034	1.4651
Frc consts --	0.8494	0.7423	0.7397
IR Inten --	6.6941	3.6856	143.1321
	16	17	18
	A	A	A
Frequencies --	951.5412	976.7210	1018.7672
Red. masses --	2.8333	2.0550	2.0268
Frc consts --	1.5115	1.1550	1.2394
IR Inten --	16.0546	25.4205	20.6712
	19	20	21
	A	A	A
Frequencies --	1024.5352	1056.6311	1133.2149
Red. masses --	1.7904	2.6936	1.7687
Frc consts --	1.1072	1.7719	1.3382
IR Inten --	30.6482	89.1912	60.3974
	22	23	24
	A	A	A

```
 Frequencies --   1181.5908              1213.0800              1244.8811
 Red. masses --      1.1150                 1.1549                 1.1804
 Frc consts  --      0.9172                 1.0013                 1.0778
 IR Inten    --      8.0321                36.3289                 2.6477

                    25                     26                     27
                     A                      A                      A
 Frequencies --   1297.6321              1327.0017              1346.3736
 Red. masses --      1.4889                 1.3148                 1.3248
 Frc consts  --      1.4771                 1.3641                 1.4150
 IR Inten    --     14.4712                17.8848                40.3878

                    28                     29                     30
                     A                      A                      A
 Frequencies --   1352.6727              1371.1206              1418.4122
 Red. masses --      1.2589                 1.4258                 1.9279
 Frc consts  --      1.3572                 1.5793                 2.2853
 IR Inten    --      7.1867                23.9493                25.5953

                    31                     32                     33
                     A                      A                      A
 Frequencies --   1448.9615              1460.9862              1471.0107
 Red. masses --      1.0973                 1.1248                 1.1034
 Frc consts  --      1.3574                 1.4146                 1.4067
 IR Inten    --     14.5421                28.3596                10.9605

                    34                     35                     36
                     A                      A                      A
 Frequencies --   1753.1875              3076.2230              3079.6187
 Red. masses --      9.1462                 1.0636                 1.0612
 Frc consts  --     16.5633                 5.9302                 5.9295
 IR Inten    --    145.2013                15.3003                26.6179

                    37                     38                     39
                     A                      A                      A
 Frequencies --   3084.9132              3100.8271              3138.1889
 Red. masses --      1.0631                 1.0856                 1.1026
 Frc consts  --      5.9611                 6.1503                 6.3979
 IR Inten    --      9.3293                46.1117                16.6740

                    40                     41                     42
                     A                      A                      A
 Frequencies --   3151.0573              3152.5880              3486.9986
 Red. masses --      1.1041                 1.1035                 1.0746
 Frc consts  --      6.4590                 6.4618                 7.6986
 IR Inten    --     11.4547                 3.7227                 1.2373
```

8e

```
                     1                      2                      3
                     A                      A                      A
 Frequencies --     99.5583               160.4967               255.1383
 Red. masses --      4.9771                 3.0130                 2.5404
 Frc consts  --      0.0291                 0.0457                 0.0974
 IR Inten    --      3.3143                 7.1415                 5.1008

                     4                      5                      6
                     A                      A                      A
 Frequencies --    307.7665               344.9462               351.9556
 Red. masses --      4.4705                 3.8878                 6.4161
 Frc consts  --      0.2495                 0.2726                 0.4683
 IR Inten    --      4.0892                15.1109                 7.4047

                     7                      8                      9
                     A                      A                      A
 Frequencies --    368.6976               463.7197               577.6804
 Red. masses --      5.9582                 2.5347                 4.3238
 Frc consts  --      0.4772                 0.3211                 0.8501
 IR Inten    --      1.2627                 4.5198                32.0774

                    10                     11                     12
                     A                      A                      A
```

```
 Frequencies --    682.9275               689.9493               772.0723
 Red. masses --      3.6366                 3.3907                 2.6836
 Frc consts  --      0.9993                 0.9510                 0.9425
 IR Inten    --     17.4811                 2.0376                 4.3102



                     13                     14                     15
                      A                      A                      A
 Frequencies --    787.4989               847.3159               902.3498
 Red. masses --      3.9745                 1.8058                 1.3951
 Frc consts  --      1.4522                 0.7639                 0.6693
 IR Inten    --     12.0177                42.6645                69.1695

                     16                     17                     18
                      A                      A                      A
 Frequencies --    975.4370              1005.7475              1037.0611
 Red. masses --      1.7779                 1.7019                 2.0829
 Frc consts  --      0.9967                 1.0143                 1.3199
 IR Inten    --     33.8478                54.4869                11.0688

                     19                     20                     21
                      A                      A                      A
 Frequencies --   1043.5032              1079.9376              1124.9582
 Red. masses --      3.7266                 2.4036                 1.9686
 Frc consts  --      2.3908                 1.6516                 1.4678
 IR Inten    --    104.2967                35.7039                16.9217

                     22                     23                     24
                      A                      A                      A
 Frequencies --   1169.1591              1215.6898              1239.3466
 Red. masses --      1.2469                 1.2258                 1.2636
 Frc consts  --      1.0042                 1.0674                 1.1435
 IR Inten    --     15.0406                 2.1943                26.5115

                     25                     26                     27
                      A                      A                      A
 Frequencies --   1279.7355              1328.6941              1335.7896
 Red. masses --      1.2467                 1.2373                 1.2993
 Frc consts  --      1.2029                 1.2870                 1.3659
 IR Inten    --      2.6452                24.2124                37.7950

                     28                     29                     30
                      A                      A                      A
 Frequencies --   1366.9323              1369.9667              1428.4241
 Red. masses --      1.3073                 1.3398                 1.8573
 Frc consts  --      1.4391                 1.4815                 2.2327
 IR Inten    --      3.4882                13.8176                35.6481

                     31                     32                     33
                      A                      A                      A
 Frequencies --   1462.8104              1475.9881              1480.5552
 Red. masses --      1.1054                 1.1238                 1.0916
 Frc consts  --      1.3936                 1.4425                 1.4098
 IR Inten    --     18.4552                11.6237                14.5724

                     34                     35                     36
                      A                      A                      A
 Frequencies --   1755.2926              3066.2345              3077.0235
 Red. masses --     10.0189                 1.0624                 1.0619
 Frc consts  --     18.1874                 5.8849                 5.9240
 IR Inten    --    183.9407                14.2064                24.7299

                     37                     38                     39
                      A                      A                      A
 Frequencies --   3082.5968              3096.6972              3128.4784
 Red. masses --      1.0623                 1.0829                 1.1018
 Frc consts  --      5.9475                 6.1183                 6.3538
 IR Inten    --     15.4435                21.7150                19.1774

                     40                     41                     42
                      A                      A                      A
 Frequencies --   3144.6807              3154.5847              3495.3666
 Red. masses --      1.1053                 1.1058                 1.0731
```

Frc consts --	6.4402	6.4835	7.7246
IR Inten --	11.0556	5.8356	11.3814

9a

	1	2	3
	A	A	A
Frequencies --	81.9750	150.4343	209.3898
Red. masses --	5.0444	4.8857	4.1477
Frc consts --	0.0200	0.0651	0.1071
IR Inten --	9.2707	1.1681	2.9334
	4	5	6
	A	A	A
Frequencies --	258.6112	327.0195	388.2848
Red. masses --	2.7861	2.6712	5.2265
Frc consts --	0.1098	0.1683	0.4643
IR Inten --	9.6823	3.9289	5.2902
	7	8	9
	A	A	A
Frequencies --	412.8388	490.3554	627.6291
Red. masses --	4.2599	2.3471	4.1707
Frc consts --	0.4278	0.3325	0.9680
IR Inten --	6.4030	18.2783	9.3328
	10	11	12
	A	A	A
Frequencies --	668.2751	716.9876	722.6003
Red. masses --	4.6965	4.0440	4.8787
Frc consts --	1.2358	1.2249	1.5009
IR Inten --	14.7767	7.5908	6.5237
	13	14	15
	A	A	A
Frequencies --	775.0435	835.8119	915.9417
Red. masses --	2.4069	1.7617	1.3691
Frc consts --	0.8519	0.7251	0.6767
IR Inten --	4.4023	20.5211	30.8296
	16	17	18
	A	A	A
Frequencies --	948.7802	973.0053	1018.3501
Red. masses --	2.8862	2.0237	2.2073
Frc consts --	1.5308	1.1289	1.3487
IR Inten --	5.0063	73.4725	54.3853
	19	20	21
	A	A	A
Frequencies --	1026.9695	1056.1549	1135.0766
Red. masses --	1.7397	2.6199	1.7258
Frc consts --	1.0810	1.7219	1.3100
IR Inten --	31.5105	111.1044	32.7808
	22	23	24
	A	A	A
Frequencies --	1181.8042	1208.4776	1241.1071
Red. masses --	1.1454	1.1530	1.1649
Frc consts --	0.9426	0.9921	1.0572
IR Inten --	7.0639	25.1232	1.5887
	25	26	27
	A	A	A
Frequencies --	1301.8570	1327.5123	1340.1888
Red. masses --	1.5007	1.3403	1.3246
Frc consts --	1.4986	1.3917	1.4017
IR Inten --	18.9934	16.2177	1.0315
	28	29	30
	A	A	A
Frequencies --	1359.4912	1365.4775	1420.6621

```
 Red. masses --      1.2011                 1.4132                 2.2370
 Frc consts  --      1.3080                 1.5525                 2.6600
 IR Inten    --     25.2980                17.0821                91.5390

                      31                     32                     33
                       A                      A                      A
 Frequencies --   1449.0929              1464.3454              1471.8518
 Red. masses --      1.1133                 1.0924                 1.0976
 Frc consts  --      1.3773                 1.3801                 1.4010
 IR Inten    --     19.3214                27.8445                10.7397

                      34                     35                     36
                       A                      A                      A
 Frequencies --   1754.8055              3070.6776              3079.6553
 Red. masses --      9.1910                 1.0600                 1.0634
 Frc consts  --     16.6752                 5.8886                 5.9421
 IR Inten    --    146.7220                20.9059                23.9156

                      37                     38                     39
                       A                      A                      A
 Frequencies --   3085.3313              3121.8590              3125.3250
 Red. masses --      1.0624                 1.0884                 1.1029
 Frc consts  --      5.9586                 6.2499                 6.3473
 IR Inten    --     17.6398                27.8406                19.1997

                      40                     41                     42
                       A                      A                      A
 Frequencies --   3150.6259              3153.4443              3491.3040
 Red. masses --      1.1044                 1.1032                 1.0745
 Frc consts  --      6.4589                 6.4633                 7.7170
 IR Inten    --     10.6981                 4.3400                 2.1251
```

9e

```
                       1                      2                      3
                       A                      A                      A
 Frequencies --     99.9991               160.5538               254.4467
 Red. masses --      5.4575                 3.0252                 2.5401
 Frc consts  --      0.0322                 0.0459                 0.0969
 IR Inten    --      5.2833                10.4944                 3.4663

                       4                      5                      6
                       A                      A                      A
 Frequencies --    305.6364               343.0717               348.8685
 Red. masses --      5.3541                 3.5472                 5.8794
 Frc consts  --      0.2947                 0.2460                 0.4216
 IR Inten    --      0.1684                 5.0757                 1.0979

                       7                      8                      9
                       A                      A                      A
 Frequencies --    368.4299               471.2688               575.0380
 Red. masses --      6.3668                 2.5116                 4.0629
 Frc consts  --      0.5092                 0.3286                 0.7916
 IR Inten    --      7.3867                 3.6791                33.4703

                      10                     11                     12
                       A                      A                      A
 Frequencies --    686.3932               690.5674               779.4667
 Red. masses --      3.6065                 3.4715                 2.9018
 Frc consts  --      1.0011                 0.9754                 1.0387
 IR Inten    --     10.1221                 1.9253                 5.1907

                      13                     14                     15
                       A                      A                      A
 Frequencies --    789.7157               850.0531               892.6434
 Red. masses --      3.4436                 1.8648                 1.3716
 Frc consts  --      1.2653                 0.7939                 0.6439
 IR Inten    --     11.4052                22.3541                47.0662

                      16                     17                     18
                       A                      A                      A
 Frequencies --    969.1444              1006.0389              1037.3406
 Red. masses --      1.6452                 1.8460                 2.1470
 Frc consts  --      0.9104                 1.1008                 1.3612
 IR Inten    --     50.8065                39.9591                27.5591

                      19                     20                     21
```

```
                       A                      A                      A
 Frequencies --   1059.5967              1076.9766              1117.9074
 Red. masses --      3.7390                 2.7847                 1.9807
 Frc consts  --      2.4734                 1.9030                 1.4584
 IR Inten    --     67.8463               112.5144                39.1100
```

```
                      22                     23                     24
                       A                      A                      A
 Frequencies --   1167.7856              1208.7328              1230.6711
 Red. masses --      1.2084                 1.1631                 1.2394
 Frc consts  --      0.9709                 1.0012                 1.1060
 IR Inten    --     19.5566                24.6399                 2.0082

                      25                     26                     27
                       A                      A                      A
 Frequencies --   1283.7283              1329.1801              1347.6654
 Red. masses --      1.2487                 1.2798                 1.2789
 Frc consts  --      1.2124                 1.3321                 1.3685
 IR Inten    --      3.7038                16.3040                37.2167

                      28                     29                     30
                       A                      A                      A
 Frequencies --   1366.3464              1368.7987              1434.0089
 Red. masses --      1.2949                 1.3894                 1.8082
 Frc consts  --      1.4243                 1.5338                 2.1908
 IR Inten    --     17.1538                 2.7065                23.3963

                      31                     32                     33
                       A                      A                      A
 Frequencies --   1473.5120              1476.2560              1481.3880
 Red. masses --      1.0962                 1.1284                 1.0940
 Frc consts  --      1.4023                 1.4490                 1.4145
 IR Inten    --     31.2298                 5.9091                11.2782

                      34                     35                     36
                       A                      A                      A
 Frequencies --   1757.1454              3062.2992              3076.8161
 Red. masses --      9.4572                 1.0604                 1.0633
 Frc consts  --     17.2040                 5.8587                 5.9307
 IR Inten    --    176.3734                18.4131                17.3678

                      37                     38                     39
                       A                      A                      A
 Frequencies --   3080.2113              3087.4974              3116.1807
 Red. masses --      1.0694                 1.0752                 1.1036
 Frc consts  --      5.9779                 6.0388                 6.3139
 IR Inten    --     11.3325                34.7352                25.8127

                      40                     41                     42
                       A                      A                      A
 Frequencies --   3144.7189              3155.2402              3501.2872
 Red. masses --      1.1053                 1.1051                 1.0746
 Frc consts  --      6.4404                 6.4819                 7.7616
 IR Inten    --     10.5943                 6.1092                 2.5906
```

10a

```
                       1                      2                      3
                       A                      A                      A
 Frequencies --     95.8492               150.4319               207.7320
 Red. masses --      3.8202                 4.9454                 3.8833
 Frc consts  --      0.0207                 0.0659                 0.0987
 IR Inten    --      1.5895                 0.7383                 6.4076

                       4                      5                      6
                       A                      A                      A
 Frequencies --    260.5130               326.8823               383.6966
 Red. masses --      2.5319                 2.7298                 3.7918
 Frc consts  --      0.1012                 0.1719                 0.3289
 IR Inten    --      4.7947                 4.8153                 3.5686

                       7                      8                      9
```

```
                      A                      A                      A
Frequencies --    387.8086               466.3763               621.7961
Red. masses --      3.1535                 2.2716                 4.2356
Frc consts  --      0.2794                 0.2911                 0.9649
IR Inten    --      1.6594                 5.9082                 3.0987




                     10                     11                     12
                      A                      A                      A
Frequencies --    664.4488               688.1356               718.7438
Red. masses --      4.5628                 2.9342                 4.7427
Frc consts  --      1.1869                 0.8186                 1.4435
IR Inten    --     19.9930                18.0614                 8.3735

                     13                     14                     15
                      A                      A                      A
Frequencies --    748.7091               786.3570               840.1792
Red. masses --      1.2801                 2.0184                 1.9696
Frc consts  --      0.4228                 0.7354                 0.8192
IR Inten    --      2.9159                 1.7335                25.3079

                     16                     17                     18
                      A                      A                      A
Frequencies --    929.7126               952.6847               971.4176
Red. masses --      2.3221                 2.0473                 1.3604
Frc consts  --      1.1826                 1.0948                 0.7564
IR Inten    --     13.4206                20.6356                79.8020

                     19                     20                     21
                      A                      A                      A
Frequencies --    990.4705              1015.3751              1035.5571
Red. masses --      1.9848                 2.2136                 2.0617
Frc consts  --      1.1472                 1.3446                 1.3026
IR Inten    --    102.0719                60.6939                13.1873

                     22                     23                     24
                      A                      A                      A
Frequencies --   1058.6235              1182.3271              1196.1615
Red. masses --      2.0119                 1.1321                 1.2729
Frc consts  --      1.3285                 0.9324                 1.0731
IR Inten    --     31.6390                11.7781                 5.6255

                     25                     26                     27
                      A                      A                      A
Frequencies --   1244.0179              1300.4221              1324.7804
Red. masses --      1.1710                 1.4967                 1.4514
Frc consts  --      1.0677                 1.4912                 1.5008
IR Inten    --      4.4251                19.3541                17.2410

                     28                     29                     30
                      A                      A                      A
Frequencies --   1331.9121              1353.9394              1359.2308
Red. masses --      1.4445                 1.2069                 1.3122
Frc consts  --      1.5098                 1.3035                 1.4284
IR Inten    --      6.4743                26.1727                13.1664

                     31                     32                     33
                      A                      A                      A
Frequencies --   1377.5075              1447.1910              1456.9770
Red. masses --      2.1342                 1.0949                 1.1351
Frc consts  --      2.3861                 1.3511                 1.4197
IR Inten    --      9.5954                14.1226                16.6057

                     34                     35                     36
                      A                      A                      A
Frequencies --   1465.5109              1476.4601              1741.1728
Red. masses --      1.0968                 1.1372                 5.9106
Frc consts  --      1.3879                 1.4606                10.5576
IR Inten    --     15.3294                21.8364                16.9681

                     37                     38                     39
                      A                      A                      A
Frequencies --   3062.0448              3075.0340              3083.1218
```

Red. masses --	1.0614	1.0631	1.0631
Frc consts --	5.8634	5.9225	5.9538
IR Inten --	31.6858	29.1833	10.3520

	40	41	42
	A	A	A
Frequencies --	3101.8820	3115.2891	3145.8674
Red. masses --	1.0857	1.1021	1.1040
Frc consts --	6.1550	6.3018	6.4375
IR Inten --	50.0370	32.5810	17.8384

	43	44	45
	A	A	A
Frequencies --	3150.2243	3150.8103	3239.2358
Red. masses --	1.0693	1.0962	1.1167
Frc consts --	6.2523	6.4117	6.9036
IR Inten --	14.2503	4.2529	13.6740

10e

	1	2	3
	A	A	A
Frequencies --	102.5678	167.9450	254.3239
Red. masses --	3.9183	3.0593	2.4826
Frc consts --	0.0243	0.0508	0.0946
IR Inten --	0.0943	5.9569	9.5767

	4	5	6
	A	A	A
Frequencies --	301.8444	339.7697	348.1284
Red. masses --	3.7082	4.3349	4.0628
Frc consts --	0.1991	0.2949	0.2901
IR Inten --	2.2839	1.5579	5.6210

	7	8	9
	A	A	A
Frequencies --	370.5412	447.6036	541.6603
Red. masses --	5.6685	2.1983	3.6688
Frc consts --	0.4586	0.2595	0.6342
IR Inten --	2.7148	2.1947	13.8490

	10	11	12
	A	A	A
Frequencies --	655.8224	683.2703	749.8270
Red. masses --	2.8767	3.9413	1.1726
Frc consts --	0.7290	1.0841	0.3884
IR Inten --	22.5448	5.3075	0.8297

	13	14	15
	A	A	A
Frequencies --	784.5890	792.0827	842.8425
Red. masses --	3.9373	2.1487	1.8556
Frc consts --	1.4280	0.7943	0.7767
IR Inten --	16.2783	1.5782	18.5341

	16	17	18
	A	A	A
Frequencies --	945.3434	958.5266	967.2173
Red. masses --	1.7051	1.3516	1.6988
Frc consts --	0.8978	0.7316	0.9364
IR Inten --	32.2754	83.0930	7.6671

	19	20	21
	A	A	A
Frequencies --	1015.8798	1040.8947	1042.2095
Red. masses --	2.2281	2.2584	3.4041
Frc consts --	1.3548	1.4417	2.1785
IR Inten --	57.7042	23.7715	97.5798

	22	23	24
	A	A	A
Frequencies --	1088.7387	1169.0434	1194.2190
Red. masses --	2.4014	1.1564	1.4177
Frc consts --	1.6771	0.9312	1.1913
IR Inten --	15.7031	9.3621	1.4207

	25	26	27

	A	A	A
Frequencies --	1222.5159	1278.9410	1295.8927
Red. masses --	1.2696	1.2704	1.5258
Frc consts --	1.1180	1.2243	1.5096
IR Inten --	10.0524	2.8458	2.9572

	28	29	30
	A	A	A
Frequencies --	1329.6536	1366.8452	1371.3029
Red. masses --	1.2413	1.3862	1.3170
Frc consts --	1.2930	1.5258	1.4592
IR Inten --	22.4101	1.4221	4.4666
	31	32	33
	A	A	A
Frequencies --	1401.4940	1444.3965	1471.7741
Red. masses --	1.6472	1.1682	1.0985
Frc consts --	1.9062	1.4359	1.4020
IR Inten --	9.6917	11.7790	1.6400
	34	35	36
	A	A	A
Frequencies --	1475.8437	1480.7135	1741.3856
Red. masses --	1.0974	1.1039	6.2682
Frc consts --	1.4084	1.4261	11.1990
IR Inten --	30.1073	9.5401	31.6922
	37	38	39
	A	A	A
Frequencies --	3052.5463	3073.1080	3076.0689
Red. masses --	1.0619	1.0622	1.0780
Frc consts --	5.8300	5.9101	6.0098
IR Inten --	31.5118	21.2083	18.8761
	40	41	42
	A	A	A
Frequencies --	3083.6684	3107.7014	3138.9803
Red. masses --	1.0667	1.1014	1.1057
Frc consts --	5.9761	6.2672	6.4192
IR Inten --	42.4969	38.1096	17.0460
	43	44	45
	A	A	A
Frequencies --	3153.4156	3161.9704	3254.6276
Red. masses --	1.1055	1.0617	1.1163
Frc consts --	6.4772	6.2539	6.9668
IR Inten --	10.7631	8.8531	6.8049

**11a**	1	2	3
	A	A	A
Frequencies --	91.3670	157.4635	236.5761
Red. masses --	6.0921	3.4414	4.3785
Frc consts --	0.0300	0.0503	0.1444
IR Inten --	13.0050	2.6359	11.3794
	4	5	6
	A	A	A
Frequencies --	291.6676	389.4762	445.7576
Red. masses --	2.9656	1.9621	5.0535
Frc consts --	0.1486	0.1754	0.5916
IR Inten --	8.5042	5.1074	2.0092
	7	8	9
	A	A	A
Frequencies --	486.9410	508.8812	645.5312
Red. masses --	4.8340	2.2908	3.7680
Frc consts --	0.6753	0.3495	0.9251
IR Inten --	26.7564	7.9959	21.8963
	10	11	12

```
                      A                      A                      A
 Frequencies --    735.8126               774.4019               818.2192
 Red. masses --      2.4265                 2.2035                 2.1782
 Frc consts  --      0.7740                 0.7786                 0.8592
 IR Inten    --     23.4167                39.3397               206.9821




                     13                     14                     15
                      A                      A                      A
 Frequencies --    858.0504               904.1149               927.2674
 Red. masses --      2.3769                 2.0082                 2.2154
 Frc consts  --      1.0311                 0.9672                 1.1223
 IR Inten    --     27.1845                22.9705                48.8101

                     16                     17                     18
                      A                      A                      A
 Frequencies --   1014.2019              1016.3521              1031.7175
 Red. masses --      3.1637                 2.0802                 2.6122
 Frc consts  --      1.9173                 1.2660                 1.6382
 IR Inten    --     71.4622                17.1815                53.1056

                     19                     20                     21
                      A                      A                      A
 Frequencies --   1111.5104              1164.4047              1201.1212
 Red. masses --      2.0740                 2.9775                 1.1894
 Frc consts  --      1.5097                 2.3786                 1.0110
 IR Inten    --     28.8723                16.7433                 1.4768

                     22                     23                     24
                      A                      A                      A
 Frequencies --   1232.4187              1274.3941              1309.0783
 Red. masses --      2.0369                 1.5100                 1.6660
 Frc consts  --      1.8228                 1.4449                 1.6821
 IR Inten    --     61.9138                 5.7221                10.4745

                     25                     26                     27
                      A                      A                      A
 Frequencies --   1324.7025              1353.7550              1372.3118
 Red. masses --      1.4031                 1.2722                 1.1995
 Frc consts  --      1.4506                 1.3737                 1.3309
 IR Inten    --     25.3912                12.0815                15.2477

                     28                     29                     30
                      A                      A                      A
 Frequencies --   1393.3738              1411.9080              1459.1760
 Red. masses --      1.5380                 1.3852                 1.1182
 Frc consts  --      1.7593                 1.6270                 1.4028
 IR Inten    --     13.4582                24.5431                21.2557

                     31                     32                     33
                      A                      A                      A
 Frequencies --   1467.0057              1505.9682              1516.7352
 Red. masses --      1.0903                 1.0972                 1.2107
 Frc consts  --      1.3825                 1.4661                 1.6410
 IR Inten    --     17.1744                10.1608                24.5331

                     34                     35                     36
                      A                      A                      A
 Frequencies --   1810.5120              3053.9383              3059.2857
 Red. masses --     11.0818                 1.0672                 1.0651
 Frc consts  --     21.4025                 5.8643                 5.8735
 IR Inten    --    460.4108                49.9217                32.6153

                     37                     38                     39
                      A                      A                      A
 Frequencies --   3076.9456              3127.6283              3128.0774
 Red. masses --      1.0616                 1.1008                 1.0998
 Frc consts  --      5.9218                 6.3442                 6.3405
 IR Inten    --      8.2745                20.7478                36.0250

                     40                     41                     42
                      A                      A                      A
 Frequencies --   3135.0089              3150.1578              3577.9091
```

Red. masses --	1.0891	1.1037	1.0746
Frc consts --	6.3066	6.4531	8.1052
IR Inten --	28.5825	20.3972	8.6070

11e

	1	2	3
	A	A	A
Frequencies --	96.2131	153.0606	282.2793
Red. masses --	5.4914	2.4800	7.9461
Frc consts --	0.0300	0.0342	0.3730
IR Inten --	4.9405	6.8111	1.5553
	4	5	6
	A	A	A
Frequencies --	330.4580	351.0852	403.6034
Red. masses --	2.0725	3.5317	5.3415
Frc consts --	0.1333	0.2565	0.5127
IR Inten --	14.9828	12.3705	2.9562
	7	8	9
	A	A	A
Frequencies --	440.5634	527.0952	608.8856
Red. masses --	3.0999	2.8347	4.3543
Frc consts --	0.3545	0.4640	0.9511
IR Inten --	0.8628	11.1788	27.2886
	10	11	12
	A	A	A
Frequencies --	710.6410	761.2949	797.1168
Red. masses --	2.3349	2.1014	1.8573
Frc consts --	0.6947	0.7176	0.6953
IR Inten --	1.8057	28.0311	267.7971
	13	14	15
	A	A	A
Frequencies --	879.7068	912.4964	963.9217
Red. masses --	2.2914	2.3059	1.4878
Frc consts --	1.0448	1.1312	0.8145
IR Inten --	44.3929	25.7244	2.8252
	16	17	18
	A	A	A
Frequencies --	1005.6143	1055.6619	1088.3914
Red. masses --	2.6036	3.2355	4.0158
Frc consts --	1.5513	2.1244	2.8028
IR Inten --	30.4338	9.6764	179.0933
	19	20	21
	A	A	A
Frequencies --	1123.4600	1156.2524	1175.0714
Red. masses --	2.1026	1.9717	1.8905
Frc consts --	1.5636	1.5531	1.5380
IR Inten --	14.5654	4.8893	7.4575
	22	23	24
	A	A	A
Frequencies --	1222.2044	1265.5167	1313.3034
Red. masses --	2.2686	1.3585	1.5281
Frc consts --	1.9966	1.2819	1.5529
IR Inten --	52.7126	3.1192	12.3042
	25	26	27
	A	A	A
Frequencies --	1334.9687	1349.0567	1370.4143
Red. masses --	1.2036	1.3127	1.1753
Frc consts --	1.2638	1.4076	1.3005
IR Inten --	16.9264	5.0705	5.9157
	28	29	30
	A	A	A

```
 Frequencies --   1395.0300               1419.5409               1458.9507
 Red. masses --      1.4092                  1.5737                  1.1115
 Frc consts  --      1.6158                  1.8684                  1.3939
 IR Inten    --     25.1097                  0.9683                 24.8710
                     31                      32                      33
                      A                       A                       A
 Frequencies --   1489.1057               1499.3576               1512.0546
 Red. masses --      1.0790                  1.0981                  1.2267
 Frc consts  --      1.4097                  1.4544                  1.6525
 IR Inten    --      6.3316                 10.2060                 23.2094
                     34                      35                      36
                      A                       A                       A
 Frequencies --   1813.5947               3058.3533               3060.0742
 Red. masses --     11.7402                  1.0672                  1.0668
 Frc consts  --     22.7513                  5.8813                  5.8855
 IR Inten    --    487.2899                 30.3104                 38.0620
                     37                      38                      39
                      A                       A                       A
 Frequencies --   3064.9446               3080.5291               3131.2636
 Red. masses --      1.0631                  1.0819                  1.0999
 Frc consts  --      5.8841                  6.0490                  6.3540
 IR Inten    --     16.6579                 34.9739                 32.6813
                     40                      41                      42
                      A                       A                       A
 Frequencies --   3137.2026               3141.3944               3570.3352
 Red. masses --      1.1020                  1.1022                  1.0750
 Frc consts  --      6.3902                  6.4083                  8.0741
 IR Inten    --     27.2159                 22.4810                  6.4005
```

12a

```
                      1                       2                       3
                      A                       A                       A
 Frequencies --     87.9829                153.4391                248.9821
 Red. masses --      6.1790                  3.5748                  4.1319
 Frc consts  --      0.0282                  0.0496                  0.1509
 IR Inten    --      4.7637                  2.3821                 12.3486
                      4                       5                       6
                      A                       A                       A
 Frequencies --    264.6963                352.8336                402.9509
 Red. masses --      4.0356                  3.0519                  2.3089
 Frc consts  --      0.1666                  0.2239                  0.2209
 IR Inten    --      6.0440                  1.7440                  5.4200
                      7                       8                       9
                      A                       A                       A
 Frequencies --    423.6778                506.6199                625.6742
 Red. masses --      4.9020                  2.5162                  5.0851
 Frc consts  --      0.5184                  0.3805                  1.1729
 IR Inten    --      4.7939                  2.4658                  7.2762
                     10                      11                      12
                      A                       A                       A
 Frequencies --    701.1737                755.0026                802.3211
 Red. masses --      3.0513                  1.8533                  2.0469
 Frc consts  --      0.8839                  0.6224                  0.7763
 IR Inten    --     10.0492                 94.2604                297.1072
                     13                      14                      15
                      A                       A                       A
 Frequencies --    854.5481                884.9707                907.7443
 Red. masses --      2.4793                  2.3479                  2.2821
 Frc consts  --      1.0667                  1.0834                  1.1079
 IR Inten    --     18.1990                 23.2120                 68.4896
                     16                      17                      18
                      A                       A                       A
 Frequencies --    999.2408               1007.3091               1021.5986
 Red. masses --      2.1755                  4.1905                  2.2055
 Frc consts  --      1.2798                  2.5052                  1.3562
 IR Inten    --     44.4054                156.2171                 64.2635
```

```
                    19                     20                     21
                     A                      A                      A
 Frequencies --   1072.6065              1151.7606              1179.3358
 Red. masses --      1.7688                 2.7280                 1.5122
 Frc consts  --      1.1990                 2.1322                 1.2392
 IR Inten    --     78.9666                43.0811                 1.0258
```

```
                    22                     23                     24
                     A                      A                      A
 Frequencies --   1220.4235              1242.3472              1264.9638
 Red. masses --      1.9652                 2.1642                 1.4455
 Frc consts  --      1.7245                 1.9681                 1.3627
 IR Inten    --     20.4864                65.0811                33.7604
                    25                     26                     27
                     A                      A                      A
 Frequencies --   1303.6855              1312.9569              1350.9988
 Red. masses --      1.5709                 1.3802                 1.2467
 Frc consts  --      1.5731                 1.4018                 1.3407
 IR Inten    --    192.9818                27.3208                 5.7718
                    28                     29                     30
                     A                      A                      A
 Frequencies --   1372.7189              1376.7135              1421.5178
 Red. masses --      1.3845                 1.3276                 1.6029
 Frc consts  --      1.5371                 1.4825                 1.9083
 IR Inten    --     13.9386                47.9126                10.4009
                    31                     32                     33
                     A                      A                      A
 Frequencies --   1471.0073              1480.1328              1493.8287
 Red. masses --      1.0869                 1.1610                 1.0994
 Frc consts  --      1.3857                 1.4986                 1.4454
 IR Inten    --      8.6448                22.9509                10.7520
                    34                     35                     36
                     A                      A                      A
 Frequencies --   1516.6311              3060.4953              3066.1656
 Red. masses --      1.2108                 1.0665                 1.0639
 Frc consts  --      1.6409                 5.8857                 5.8932
 IR Inten    --     20.4264                46.0185                30.8764
                    37                     38                     39
                     A                      A                      A
 Frequencies --   3072.0484              3133.1088              3137.2819
 Red. masses --      1.0622                 1.1021                 1.1004
 Frc consts  --      5.9064                 6.3740                 6.3814
 IR Inten    --     13.6469                17.8993                40.6446
                    40                     41                     42
                     A                      A                      A
 Frequencies --   3140.9040              3151.2630              3579.7024
 Red. masses --      1.0896                 1.1028                 1.0748
 Frc consts  --      6.3334                 6.4526                 8.1149
 IR Inten    --     17.7675                13.4863                13.1976
```

12e

```
                     1                      2                      3
                     A                      A                      A
 Frequencies --     81.4479               157.4116               238.1948
 Red. masses --      5.3995                 2.6076                14.4019
 Frc consts  --      0.0211                 0.0381                 0.4814
 IR Inten    --      1.9092                 3.0071                 1.8008
                     4                      5                      6
                     A                      A                      A
 Frequencies --    316.3326               356.5163               377.9297
 Red. masses --      2.4618                 3.7947                 3.0351
 Frc consts  --      0.1451                 0.2842                 0.2554
 IR Inten    --     11.3907                 8.8599                 3.1705
```

```
                      7                      8                      9
                      A                      A                      A
 Frequencies --    437.6584               496.9242               577.3330
 Red. masses --      3.3859                 3.8668                 4.2349
 Frc consts  --      0.3821                 0.5626                 0.8317
 IR Inten    --      1.0143                13.8905                13.5513




                     10                     11                     12
                      A                      A                      A
 Frequencies --    659.5289               734.7551               794.4229
 Red. masses --      2.6513                 2.7545                 1.7071
 Frc consts  --      0.6795                 0.8762                 0.6348
 IR Inten    --     18.3402                24.8464               351.0151

                     13                     14                     15
                      A                      A                      A
 Frequencies --    847.3889               900.9934               978.8476
 Red. masses --      1.7826                 2.4484                 1.5530
 Frc consts  --      0.7542                 1.1710                 0.8767
 IR Inten    --     33.3904                17.3593                 1.5480

                     16                     17                     18
                      A                      A                      A
 Frequencies --    990.7416              1021.8209              1083.1862
 Red. masses --      2.8383                 3.2757                 2.0303
 Frc consts  --      1.6415                 2.0152                 1.4035
 IR Inten    --     84.3997                11.9979                44.1911

                     19                     20                     21
                      A                      A                      A
 Frequencies --   1089.9377              1144.4425              1165.8709
 Red. masses --      3.8735                 1.9348                 1.6431
 Frc consts  --      2.7112                 1.4931                 1.3159
 IR Inten    --    165.2783                23.8982                12.9740

                     22                     23                     24
                      A                      A                      A
 Frequencies --   1208.0933              1230.0973              1288.6339
 Red. masses --      2.4238                 2.1402                 1.4609
 Frc consts  --      2.0842                 1.9080                 1.4294
 IR Inten    --     33.1437                44.8733                 2.1330

                     25                     26                     27
                      A                      A                      A
 Frequencies --   1312.9577              1321.9360              1344.2895
 Red. masses --      1.3231                 1.8226                 1.2631
 Frc consts  --      1.3438                 1.8766                 1.3449
 IR Inten    --     92.3646               204.0808                28.5741

                     28                     29                     30
                      A                      A                      A
 Frequencies --   1357.6514              1395.4850              1413.9587
 Red. masses --      1.1616                 1.4648                 1.5471
 Frc consts  --      1.2615                 1.6807                 1.8224
 IR Inten    --      8.2246                 9.9375                10.1260

                     31                     32                     33
                      A                      A                      A
 Frequencies --   1480.2942              1490.1142              1501.3957
 Red. masses --      1.1287                 1.0827                 1.1046
 Frc consts  --      1.4573                 1.4164                 1.4670
 IR Inten    --     24.0867                 4.9137                12.7411

                     34                     35                     36
                      A                      A                      A
 Frequencies --   1519.2781              3055.4219              3057.9135
 Red. masses --      1.2141                 1.0670                 1.0658
 Frc consts  --      1.6512                 5.8688                 5.8721
 IR Inten    --     18.1115                14.1348                25.0412

                     37                     38                     39
                      A                      A                      A
```

Frequencies --	3063.6455	3079.1710	3138.1514
Red. masses --	1.0651	1.0821	1.1014
Frc consts --	5.8901	6.0446	6.3906
IR Inten --	48.4694	37.1910	30.7773
	40	41	42
	A	A	A
Frequencies --	3139.1226	3142.7371	3575.9565
Red. masses --	1.0997	1.1018	1.0752
Frc consts --	6.3846	6.4119	8.1008
IR Inten --	28.7855	15.6683	9.7597

**13a**	1	2	3
	A	A	A
Frequencies --	104.4161	153.7493	238.9112
Red. masses --	4.5144	3.3003	4.5098
Frc consts --	0.0290	0.0460	0.1517
IR Inten --	6.1583	3.1141	10.7260
	4	5	6
	A	A	A
Frequencies --	291.2268	389.9025	440.3723
Red. masses --	2.6750	1.9408	3.7129
Frc consts --	0.1337	0.1738	0.4242
IR Inten --	14.0155	3.3446	14.3090
	7	8	9
	A	A	A
Frequencies --	479.0635	503.9598	647.5880
Red. masses --	4.8005	2.0925	4.1881
Frc consts --	0.6491	0.3131	1.0348
IR Inten --	19.0669	8.2624	8.2881
	10	11	12
	A	A	A
Frequencies --	718.0195	780.8806	812.0856
Red. masses --	2.1551	2.3348	2.0112
Frc consts --	0.6546	0.8388	0.7815
IR Inten --	31.9378	56.7663	193.4045
	13	14	15
	A	A	A
Frequencies --	858.1479	898.3454	929.7332
Red. masses --	2.4156	1.7979	2.0319
Frc consts --	1.0481	0.8549	1.0348
IR Inten --	22.0674	41.8167	97.1135
	16	17	18
	A	A	A
Frequencies --	948.8757	1015.5734	1029.1697
Red. masses --	1.6863	2.9016	1.7732
Frc consts --	0.8945	1.7632	1.1066
IR Inten --	80.0208	3.5523	14.7118
	19	20	21
	A	A	A
Frequencies --	1037.9265	1113.1529	1151.0022
Red. masses --	2.6005	1.9688	1.6456
Frc consts --	1.6506	1.4374	1.2845
IR Inten --	71.6202	13.7972	99.6612
	22	23	24
	A	A	A
Frequencies --	1183.8181	1213.0016	1253.2303
Red. masses --	3.0921	1.1484	1.3181
Frc consts --	2.5531	0.9956	1.2197
IR Inten --	37.7872	6.2684	16.8627
	25	26	27
	A	A	A
Frequencies --	1319.7216	1330.7141	1354.9894
Red. masses --	1.5971	1.4641	1.2062
Frc consts --	1.6389	1.5275	1.3048
IR Inten --	10.0415	14.5611	13.8887

```
                      28                     29                     30
                       A                      A                      A
 Frequencies --   1363.5585              1380.2468              1411.3064
 Red. masses --      1.2722                 1.2231                 1.3869
 Frc consts  --      1.3937                 1.3728                 1.6275
 IR Inten    --      4.3563                11.8981                33.8152
```

```
                      31                     32                     33
                       A                      A                      A
 Frequencies --   1425.3363              1461.0971              1465.6488
 Red. masses --      1.8980                 1.1411                 1.1082
 Frc consts  --      2.2718                 1.4352                 1.4026
 IR Inten    --     14.7365                13.1384                25.4940

                      34                     35                     36
                       A                      A                      A
 Frequencies --   1499.9838              1513.0015              1750.1633
 Red. masses --      1.0958                 1.2157                 9.1318
 Frc consts  --      1.4526                 1.6397                16.4803
 IR Inten    --      8.4472                24.9517               170.1800

                      37                     38                     39
                       A                      A                      A
 Frequencies --   3047.0295              3057.5454              3066.7762
 Red. masses --      1.0678                 1.0650                 1.0630
 Frc consts  --      5.8413                 5.8660                 5.8904
 IR Inten    --     51.5375                33.3784                18.5491

                      40                     41                     42
                       A                      A                      A
 Frequencies --   3105.7795              3123.1944              3125.5419
 Red. masses --      1.0882                 1.0987                 1.1008
 Frc consts  --      6.1845                 6.3145                 6.3362
 IR Inten    --     48.0483                44.6283                26.0283

                      43                     44                     45
                       A                      A                      A
 Frequencies --   3137.7210              3485.7691              3571.8290
 Red. masses --      1.1019                 1.0744                 1.0745
 Frc consts  --      6.3918                 7.6915                 8.0769
 IR Inten    --     25.7080                 1.2467                 6.3460
```

13e

```
                       1                      2                      3
                       A                      A                      A
 Frequencies --    110.3511               158.5560               305.9338
 Red. masses --      4.2906                 2.4571                 4.3251
 Frc consts  --      0.0308                 0.0364                 0.2385
 IR Inten    --      1.1930                 3.2203                 2.5528

                       4                      5                      6
                       A                      A                      A
 Frequencies --    332.5609               354.3144               404.1016
 Red. masses --      2.1601                 3.7874                 4.9503
 Frc consts  --      0.1408                 0.2801                 0.4763
 IR Inten    --     25.5371                16.3137                 0.3428

                       7                      8                      9
                       A                      A                      A
 Frequencies --    441.5718               519.0023               598.5448
 Red. masses --      2.9470                 2.7946                 3.9703
 Frc consts  --      0.3386                 0.4435                 0.8380
 IR Inten    --      3.8823                 5.1431                35.1572

                      10                     11                     12
                       A                      A                      A
 Frequencies --    695.4821               774.3160               800.6197
 Red. masses --      2.1589                 2.2805                 1.8189
 Frc consts  --      0.6152                 0.8056                 0.6869
 IR Inten    --      5.5426                22.4063               254.6875

                      13                     14                     15
```

```
                      A                      A                      A
Frequencies --    877.2362               897.1274               920.4935
Red. masses --      1.8388                 1.8486                 1.5000
Frc consts  --      0.8337                 0.8766                 0.7488
IR Inten    --     48.5336                68.4030                21.0661




                     16                     17                     18
                      A                      A                      A
Frequencies --   1004.2305              1018.3916              1054.8711
Red. masses --      2.5776                 1.5270                 3.0411
Frc consts  --      1.5315                 0.9331                 1.9938
IR Inten    --     27.2929                13.7270                76.9608

                     19                     20                     21
                      A                      A                      A
Frequencies --   1074.3650              1116.6754              1145.0972
Red. masses --      3.2287                 2.0783                 2.2316
Frc consts  --      2.1958                 1.5269                 1.7241
IR Inten    --     97.5389                 7.8314                25.7758

                     22                     23                     24
                      A                      A                      A
Frequencies --   1191.7363              1196.2302              1252.9152
Red. masses --      2.9369                 1.2910                 1.3066
Frc consts  --      2.4575                 1.0885                 1.2085
IR Inten    --     24.7867                 8.2281                13.2069

                     25                     26                     27
                      A                      A                      A
Frequencies --   1322.3492              1331.5403              1339.7239
Red. masses --      1.4258                 1.2996                 1.2184
Frc consts  --      1.4690                 1.3576                 1.2884
IR Inten    --      1.0533                35.8202                 4.3980

                     28                     29                     30
                      A                      A                      A
Frequencies --   1367.7190              1373.6482              1401.7481
Red. masses --      1.2736                 1.1933                 1.3899
Frc consts  --      1.4037                 1.3266                 1.6090
IR Inten    --      4.2607                 3.4056                46.0336

                     31                     32                     33
                      A                      A                      A
Frequencies --   1438.5289              1465.6246              1487.4107
Red. masses --      1.7809                 1.1472                 1.0870
Frc consts  --      2.1713                 1.4519                 1.4169
IR Inten    --     22.2356                24.4809                 6.3026

                     34                     35                     36
                      A                      A                      A
Frequencies --   1497.0714              1509.7048              1749.7943
Red. masses --      1.0988                 1.2224                 9.9328
Frc consts  --      1.4510                 1.6416                17.9182
IR Inten    --     10.0398                22.6815               212.3200

                     37                     38                     39
                      A                      A                      A
Frequencies --   3051.8423              3057.8637              3058.5942
Red. masses --      1.0675                 1.0659                 1.0636
Frc consts  --      5.8580                 5.8724                 5.8624
IR Inten    --     33.6614                49.6496                21.0818

                     40                     41                     42
                      A                      A                      A
Frequencies --   3084.2855              3124.3360              3134.4308
Red. masses --      1.0836                 1.0996                 1.1011
Frc consts  --      6.0732                 6.3244                 6.3736
IR Inten    --     35.5112                41.3841                48.3204

                     43                     44                     45
                      A                      A                      A
Frequencies --   3134.9897              3494.4898              3566.4295
```

Red. masses --	1.1015	1.0732	1.0748
Frc consts --	6.3781	7.7211	8.0549
IR Inten --	15.1940	8.1658	4.5801

14a

	1	2	3
	A	A	A
Frequencies --	104.7498	162.0635	240.7467
Red. masses --	4.6431	3.4521	3.8858
Frc consts --	0.0300	0.0534	0.1327
IR Inten --	6.9315	4.3349	12.7264

	4	5	6
	A	A	A
Frequencies --	292.8771	392.9824	439.8581
Red. masses --	2.7502	1.9513	4.3650
Frc consts --	0.1390	0.1775	0.4976
IR Inten --	6.2995	6.9845	7.7942

	7	8	9
	A	A	A
Frequencies --	475.2036	507.1378	650.7198
Red. masses --	4.0502	2.1171	4.0736
Frc consts --	0.5389	0.3208	1.0163
IR Inten --	15.2689	8.8868	12.7954

	10	11	12
	A	A	A
Frequencies --	724.6372	778.3909	818.0090
Red. masses --	2.3389	2.3893	2.0477
Frc consts --	0.7236	0.8529	0.8073
IR Inten --	23.3903	21.4021	211.5886

	13	14	15
	A	A	A
Frequencies --	859.0899	892.0673	930.6291
Red. masses --	2.3951	1.6571	2.0447
Frc consts --	1.0415	0.7770	1.0433
IR Inten --	48.6878	34.2984	30.6467

	16	17	18
	A	A	A
Frequencies --	942.9107	1015.6221	1032.8802
Red. masses --	1.6376	2.8523	1.8227
Frc consts --	0.8578	1.7335	1.1457
IR Inten --	44.6079	21.1007	30.6784

	19	20	21
	A	A	A
Frequencies --	1035.4029	1111.5987	1150.0534
Red. masses --	2.4598	2.0515	1.4917
Frc consts --	1.5537	1.4936	1.1624
IR Inten --	146.9464	33.9043	12.1202

	22	23	24
	A	A	A
Frequencies --	1182.4487	1210.8781	1251.9395
Red. masses --	3.1757	1.1637	1.3445
Frc consts --	2.6161	1.0053	1.2416
IR Inten --	53.1234	3.4386	26.1985

	25	26	27
	A	A	A
Frequencies --	1317.8064	1329.0361	1346.5977
Red. masses --	1.6063	1.4372	1.2648
Frc consts --	1.6435	1.4957	1.3512
IR Inten --	3.7427	3.5443	9.3709

	28	29	30
	A	A	A

Frequencies --	1366.7817	1374.1519	1409.7668
Red. masses --	1.2905	1.1881	1.3783
Frc consts --	1.4204	1.3218	1.6140
IR Inten --	20.3875	11.7247	16.4337
	31	32	33
	A	A	A
Frequencies --	1429.8361	1464.3637	1467.0384
Red. masses --	2.2035	1.0917	1.0998
Frc consts --	2.6543	1.3793	1.3946
IR Inten --	78.5816	7.4513	28.3971
	34	35	36
	A	A	A
Frequencies --	1499.9793	1511.8559	1751.5664
Red. masses --	1.0948	1.2170	9.1720
Frc consts --	1.4513	1.6390	16.5794
IR Inten --	8.1637	24.7955	171.0476
	37	38	39
	A	A	A
Frequencies --	3048.8486	3056.9153	3064.6197
Red. masses --	1.0675	1.0649	1.0607
Frc consts --	5.8462	5.8633	5.8696
IR Inten --	52.9572	36.6884	16.8842
	40	41	42
	A	A	A
Frequencies --	3121.2085	3123.8853	3126.7252
Red. masses --	1.0990	1.1008	1.1044
Frc consts --	6.3079	6.3294	6.3616
IR Inten --	48.0765	21.5976	34.6504
	43	44	45
	A	A	A
Frequencies --	3132.8065	3488.8443	3573.4694
Red. masses --	1.0888	1.0743	1.0744
Frc consts --	6.2958	7.7041	8.0836
IR Inten --	32.4303	1.9984	6.1435

14e

	1	2	3
	A	A	A
Frequencies --	99.5871	158.8273	295.7095
Red. masses --	4.5734	2.4853	5.8429
Frc consts --	0.0267	0.0369	0.3010
IR Inten --	2.3389	7.6079	0.5962
	4	5	6
	A	A	A
Frequencies --	329.7248	352.0252	403.6376
Red. masses --	2.0638	3.3938	4.5865
Frc consts --	0.1322	0.2478	0.4403
IR Inten --	11.0579	2.8605	9.9688
	7	8	9
	A	A	A
Frequencies --	441.8712	521.0796	595.0039
Red. masses --	3.0058	2.7699	3.7407
Frc consts --	0.3458	0.4431	0.7803
IR Inten --	1.4644	22.7331	20.5831
	10	11	12
	A	A	A
Frequencies --	694.4590	781.8678	802.5171
Red. masses --	2.1846	2.1882	1.8409
Frc consts --	0.6208	0.7881	0.6985
IR Inten --	4.1787	29.8353	255.0780
	13	14	15
	A	A	A
Frequencies --	877.6002	889.8392	918.8837
Red. masses --	1.9099	1.6102	1.7545
Frc consts --	0.8667	0.7512	0.8728
IR Inten --	34.3896	39.2317	30.1118

```
                     16                     17                     18
                      A                      A                      A
 Frequencies --   1003.7296              1018.9483              1060.6448
 Red. masses --      2.3801                 1.6232                 2.7082
 Frc consts  --      1.4128                 0.9930                 1.7950
 IR Inten    --     36.4943                35.3256                 2.5515
```

```
                     19                     20                     21
                      A                      A                      A
 Frequencies --   1083.1926              1110.7283              1139.7468
 Red. masses --      3.7481                 2.2545                 1.9807
 Frc consts  --      2.5910                 1.6388                 1.5160
 IR Inten    --    135.4074                76.7008                10.9011
                     22                     23                     24
                      A                      A                      A
 Frequencies --   1185.3916              1194.6548              1249.1571
 Red. masses --      2.0890                 1.4703                 1.3153
 Frc consts  --      1.7295                 1.2364                 1.2092
 IR Inten    --     38.2234                12.2528                28.7673
                     25                     26                     27
                      A                      A                      A
 Frequencies --   1320.7192              1338.8925              1344.7070
 Red. masses --      1.4255                 1.3447                 1.2001
 Frc consts  --      1.4650                 1.4202                 1.2786
 IR Inten    --      8.3510                 4.7033                29.0473
                     28                     29                     30
                      A                      A                      A
 Frequencies --   1367.3506              1373.1579              1399.6196
 Red. masses --      1.3060                 1.2026                 1.3964
 Frc consts  --      1.4387                 1.3360                 1.6116
 IR Inten    --     15.4925                 3.0000                11.9465
                     31                     32                     33
                      A                      A                      A
 Frequencies --   1442.6066              1472.1257              1488.3348
 Red. masses --      1.8607                 1.1085                 1.0854
 Frc consts  --      2.2815                 1.4153                 1.4166
 IR Inten    --     18.7371                29.0718                 4.6712
                     34                     35                     36
                      A                      A                      A
 Frequencies --   1496.7242              1508.9543              1751.8927
 Red. masses --      1.1006                 1.2261                 9.4036
 Frc consts  --      1.4527                 1.6449                17.0043
 IR Inten    --     10.3811                20.3321               205.2505
                     37                     38                     39
                      A                      A                      A
 Frequencies --   3052.8743              3055.7316              3057.3762
 Red. masses --      1.0666                 1.0670                 1.0623
 Frc consts  --      5.8572                 5.8703                 5.8508
 IR Inten    --     29.5226                28.2099                34.4993
                     40                     41                     42
                      A                      A                      A
 Frequencies --   3072.6856              3119.3303              3127.0476
 Red. masses --      1.0820                 1.1005                 1.1025
 Frc consts  --      6.0190                 6.3088                 6.3518
 IR Inten    --     50.3487                39.8314                37.8314
                     43                     44                     45
                      A                      A                      A
 Frequencies --   3133.5206              3499.1804              3564.1286
 Red. masses --      1.1016                 1.0743                 1.0749
 Frc consts  --      6.3728                 7.7503                 8.0447
 IR Inten    --     34.5115                 2.5259                 4.6803
```

15a

```
                      1                      2                      3
                      A                      A                      A
```

```
 Frequencies --    112.0054                161.3559                239.1748
 Red. masses --      3.6618                  3.3167                  3.6928
 Frc consts  --      0.0271                  0.0509                  0.1245
 IR Inten    --      1.8313                  4.9600                 12.3013
                      4                      5                      6
                      A                      A                      A
 Frequencies --    290.6711                381.1965                410.4414
 Red. masses --      2.5080                  2.0687                  2.2405
 Frc consts  --      0.1248                  0.1771                  0.2224
 IR Inten    --      8.7256                  2.9241                  2.8658
                      7                      8                      9
                      A                      A                      A
 Frequencies --    464.9786                495.3259                638.3620
 Red. masses --      4.4905                  2.1191                  4.1703
 Frc consts  --      0.5720                  0.3063                  1.0013
 IR Inten    --      5.0518                  5.6046                  2.7485
                     10                     11                     12
                      A                      A                      A
 Frequencies --    704.7139                751.5753                790.3145
 Red. masses --      2.0096                  1.2449                  2.2145
 Frc consts  --      0.5880                  0.4143                  0.8149
 IR Inten    --     42.0508                  8.7643                 16.7487
                     13                     14                     15
                      A                      A                      A
 Frequencies --    816.2167                854.7650                907.3406
 Red. masses --      1.9470                  2.7514                  2.0730
 Frc consts  --      0.7642                  1.1844                  1.0055
 IR Inten    --    228.8770                 47.6127                  5.6438
                     16                     17                     18
                      A                      A                      A
 Frequencies --    921.3015                968.1195                996.3964
 Red. masses --      1.8810                  1.3648                  2.5407
 Frc consts  --      0.9407                  0.7537                  1.4862
 IR Inten    --     53.7539                 59.9396                 83.3151
                     19                     20                     21
                      A                      A                      A
 Frequencies --   1011.6038               1024.4982               1044.2395
 Red. masses --      1.6945                  2.4866                  1.9434
 Frc consts  --      1.0217                  1.5377                  1.2486
 IR Inten    --     15.8392                 59.7592                  9.6345
                     22                     23                     24
                      A                      A                      A
 Frequencies --   1118.1729               1180.7915               1215.5940
 Red. masses --      1.7580                  3.7429                  1.1129
 Frc consts  --      1.2951                  3.0747                  0.9689
 IR Inten    --     16.7836                 46.2914                 11.6622
                     25                     26                     27
                      A                      A                      A
 Frequencies --   1253.3914               1313.9399               1326.4980
 Red. masses --      1.4558                  1.6046                  1.5008
 Frc consts  --      1.3475                  1.6322                  1.5560
 IR Inten    --     14.8857                  4.0483                  0.1532
                     28                     29                     30
                      A                      A                      A
 Frequencies --   1340.7613               1355.0843               1373.9768
 Red. masses --      1.4218                  1.3559                  1.2227
 Frc consts  --      1.5059                  1.4669                  1.3600
 IR Inten    --      5.6767                 19.3706                  4.2386
                     31                     32                     33
                      A                      A                      A
 Frequencies --   1393.3046               1410.4900               1456.8269
 Red. masses --      1.6585                  1.3890                  1.1517
 Frc consts  --      1.8970                  1.6282                  1.4402
 IR Inten    --      2.6388                 17.9959                 13.4350
                     34                     35                     36
                      A                      A                      A
 Frequencies --   1464.5815               1475.7682               1495.0454
```

Red. masses --	1.0865	1.1460	1.0951
Frc consts --	1.3731	1.4706	1.4421
IR Inten --	7.1267	27.3629	7.3103
	37	38	39
	A	A	A
Frequencies --	1508.8131	1741.4496	3040.7900
Red. masses --	1.2154	6.0004	1.0675
Frc consts --	1.6302	10.7214	5.8156
IR Inten --	23.4493	25.8144	52.3579
	40	41	42
	A	A	A
Frequencies --	3050.7114	3054.7268	3108.8451
Red. masses --	1.0630	1.0646	1.0888
Frc consts --	5.8288	5.8530	6.2001
IR Inten --	41.4206	37.6097	73.6631
	43	44	45
	A	A	A
Frequencies --	3112.1368	3115.8487	3120.1572
Red. masses --	1.0965	1.1030	1.1009
Frc consts --	6.2571	6.3093	6.3145
IR Inten --	33.7993	50.8396	35.4788
	46	47	48
	A	A	A
Frequencies --	3143.4664	3232.5223	3566.5598
Red. masses --	1.0618	1.1167	1.0743
Frc consts --	6.1819	6.8752	8.0512
IR Inten --	12.1406	16.3155	4.2034

**15e**	1	2	3
	A	A	A
Frequencies --	113.2588	170.1147	285.7459
Red. masses --	3.5228	2.4456	3.9551
Frc consts --	0.0266	0.0417	0.1903
IR Inten --	1.8871	2.7207	5.0671
	4	5	6
	A	A	A
Frequencies --	327.3473	351.8401	390.0516
Red. masses --	2.0742	3.2441	3.1931
Frc consts --	0.1310	0.2366	0.2862
IR Inten --	17.4852	4.0002	5.1334
	7	8	9
	A	A	A
Frequencies --	439.9635	488.4404	571.9613
Red. masses --	2.7979	3.1005	3.4553
Frc consts --	0.3191	0.4358	0.6660
IR Inten --	3.0933	5.3608	16.1719
	10	11	12
	A	A	A
Frequencies --	669.5975	748.1263	793.8872
Red. masses --	1.9973	1.1308	2.0477
Frc consts --	0.5276	0.3729	0.7604
IR Inten --	17.3120	0.2944	31.3651
	13	14	15
	A	A	A
Frequencies --	802.4881	865.8334	903.1034
Red. masses --	1.8337	1.9895	1.9737
Frc consts --	0.6958	0.8787	0.9484
IR Inten --	238.1623	16.2802	9.7889
	16	17	18
	A	A	A
Frequencies --	945.3924	985.1855	1006.3824
Red. masses --	1.3552	2.0318	1.6476
Frc consts --	0.7136	1.1619	0.9832
IR Inten --	81.9170	31.6571	8.5577

	19	20	21
	A	A	A
Frequencies --	1013.2573	1062.1477	1080.7119
Red. masses --	2.0494	4.6062	2.4384
Frc consts --	1.2397	3.0617	1.6780
IR Inten --	14.3580	158.6221	0.2857

	22	23	24
	A	A	A
Frequencies --	1129.3112	1173.4105	1195.1910
Red. masses --	1.9616	2.8894	1.3581
Frc consts --	1.4740	2.3440	1.1431
IR Inten --	4.3687	28.3412	7.0891

	25	26	27
	A	A	A
Frequencies --	1237.9257	1284.0512	1326.0607
Red. masses --	1.5937	1.5008	1.3453
Frc consts --	1.4390	1.4579	1.3937
IR Inten --	10.7212	5.9350	1.4002

	28	29	30
	A	A	A
Frequencies --	1338.2260	1365.2026	1370.5930
Red. masses --	1.2652	1.3904	1.2477
Frc consts --	1.3350	1.5268	1.3809
IR Inten --	3.0396	2.1330	4.5623

	31	32	33
	A	A	A
Frequencies --	1390.5572	1410.9339	1445.3138
Red. masses --	1.4191	1.5216	1.1803
Frc consts --	1.6167	1.7847	1.4526
IR Inten --	9.1575	12.6494	7.9779

	34	35	36
	A	A	A
Frequencies --	1476.3565	1485.5794	1494.5111
Red. masses --	1.1186	1.0820	1.1002
Frc consts --	1.4365	1.4070	1.4478
IR Inten --	24.0876	3.3842	10.0142

	37	38	39
	A	A	A
Frequencies --	1507.5953	1741.1713	3038.9001
Red. masses --	1.2167	6.3284	1.0667
Frc consts --	1.6293	11.3038	5.8039
IR Inten --	18.0225	46.5763	31.1256

	40	41	42
	A	A	A
Frequencies --	3049.3005	3051.0834	3062.6762
Red. masses --	1.0642	1.0670	1.0812
Frc consts --	5.8299	5.8521	5.9750
IR Inten --	58.9041	30.8606	72.1217

	43	44	45
	A	A	A
Frequencies --	3111.4970	3118.2094	3123.3646
Red. masses --	1.0974	1.1021	1.1027
Frc consts --	6.2597	6.3137	6.3383
IR Inten --	52.6646	51.1667	47.1651

	46	47	48
	A	A	A
Frequencies --	3159.3693	3252.9538	3560.9027
Red. masses --	1.0614	1.1161	1.0747
Frc consts --	6.2424	6.9583	8.0287
IR Inten --	9.7218	8.8926	2.4278

16a

	1	2	3
	A	A	A
Frequencies --	76.8374	170.3370	223.3085
Red. masses --	5.4649	3.2296	4.5915
Frc consts --	0.0190	0.0552	0.1349
IR Inten --	12.4323	0.0886	5.2913

	4	5	6
	A	A	A
Frequencies --	298.5010	389.1577	428.4253
Red. masses --	3.4523	1.9141	5.2739
Frc consts --	0.1812	0.1708	0.5703
IR Inten --	2.8045	8.0368	10.3162
	7	8	9
	A	A	A
Frequencies --	467.3947	503.2114	629.0584
Red. masses --	3.4467	2.3871	2.9949
Frc consts --	0.4436	0.3561	0.6983
IR Inten --	42.4661	16.2330	56.0395
	10	11	12
	A	A	A
Frequencies --	688.1657	766.6775	820.6711
Red. masses --	2.4075	3.3153	1.8642
Frc consts --	0.6717	1.1481	0.7397
IR Inten --	91.0127	43.8696	30.2911
	13	14	15
	A	A	A
Frequencies --	888.3069	901.2581	954.7008
Red. masses --	2.4635	2.3011	2.1970
Frc consts --	1.1453	1.1012	1.1798
IR Inten --	54.8837	21.2136	57.9191
	16	17	18
	A	A	A
Frequencies --	1013.8687	1034.8438	1068.2531
Red. masses --	3.4138	2.2228	1.6651
Frc consts --	2.0675	1.4025	1.1195
IR Inten --	2.1672	68.7948	2.2501
	19	20	21
	A	A	A
Frequencies --	1100.6371	1175.8117	1190.5954
Red. masses --	2.5151	2.4321	1.5833
Frc consts --	1.7951	1.9811	1.3224
IR Inten --	23.4044	6.1716	10.7774
	22	23	24
	A	A	A
Frequencies --	1210.3624	1270.5991	1302.7837
Red. masses --	1.2285	1.6044	1.3098
Frc consts --	1.0603	1.5261	1.3098
IR Inten --	25.4190	29.9798	7.4090
	25	26	27
	A	A	A
Frequencies --	1327.3721	1348.5143	1369.3168
Red. masses --	1.4583	1.4422	1.5017
Frc consts --	1.5138	1.5453	1.6590
IR Inten --	18.0244	10.1655	16.7913
	28	29	30
	A	A	A
Frequencies --	1377.4914	1427.1303	1446.6174
Red. masses --	1.4582	1.5076	1.1121
Frc consts --	1.6302	1.8090	1.3712
IR Inten --	53.1708	11.8833	23.4990
	31	32	33
	A	A	A

Frequencies --	1476.7544	1493.3903	1516.6477
Red. masses --	1.0860	1.3348	1.1062
Frc consts --	1.3954	1.7540	1.4992
IR Inten --	10.4799	20.3220	5.4095

	34	35	36
	A	A	A
Frequencies --	1819.9592	2960.8019	2972.0357
Red. masses --	11.2718	1.0718	1.0728
Frc consts --	21.9972	5.5357	5.5831
IR Inten --	418.4170	109.5545	99.1024

	37	38	39
	A	A	A
Frequencies --	3071.3003	3120.0874	3122.3750
Red. masses --	1.0632	1.0904	1.0915
Frc consts --	5.9090	6.2544	6.2694
IR Inten --	9.1931	27.1208	33.8507

	40	41	42
	A	A	A
Frequencies --	3135.1784	3151.8288	3600.8573
Red. masses --	1.0885	1.1014	1.0755
Frc consts --	6.3038	6.4463	8.2159
IR Inten --	29.3657	17.3345	13.4079

16e

	1	2	3
	A	A	A
Frequencies --	90.2036	163.5648	282.9371
Red. masses --	5.1424	2.4987	7.0443
Frc consts --	0.0247	0.0394	0.3323
IR Inten --	3.7413	7.5036	4.8008

	4	5	6
	A	A	A
Frequencies --	344.0789	357.6006	401.2311
Red. masses --	2.7849	2.9897	4.2037
Frc consts --	0.1943	0.2253	0.3987
IR Inten --	1.7783	19.9618	7.3950

	7	8	9
	A	A	A
Frequencies --	438.7553	514.8450	601.7492
Red. masses --	3.3308	2.4404	4.0349
Frc consts --	0.3778	0.3811	0.8608
IR Inten --	3.4890	22.5940	44.5876

	10	11	12
	A	A	A
Frequencies --	668.0186	759.4189	804.4417
Red. masses --	2.6274	2.3543	1.6138
Frc consts --	0.6908	0.8000	0.6153
IR Inten --	90.5454	11.7112	68.3788

	13	14	15
	A	A	A
Frequencies --	898.1513	912.5821	990.4794
Red. masses --	2.7025	2.4733	1.4554
Frc consts --	1.2844	1.2136	0.8412
IR Inten --	91.8888	5.1410	11.0653

	16	17	18
	A	A	A
Frequencies --	1013.3758	1095.9847	1097.1535
Red. masses --	2.4259	2.1984	3.7582
Frc consts --	1.4678	1.5558	2.6654
IR Inten --	14.6460	19.6457	123.0380

	19	20	21
	A	A	A
Frequencies --	1122.8661	1159.1207	1183.7749
Red. masses --	2.5926	2.5412	1.8921
Frc consts --	1.9259	2.0116	1.5622
IR Inten --	19.4822	21.1408	6.2241

	22	23	24
	A	A	A
Frequencies --	1200.3642	1265.7038	1295.5088
Red. masses --	1.3334	1.3770	1.3799
Frc consts --	1.1320	1.2997	1.3645
IR Inten --	30.8376	11.5949	11.7146

	25	26	27
	A	A	A
Frequencies --	1323.3069	1342.3985	1359.1679
Red. masses --	1.2315	1.3112	1.4034
Frc consts --	1.2706	1.3921	1.5274
IR Inten --	9.4024	7.7527	11.1232

	28	29	30
	A	A	A
Frequencies --	1394.2072	1429.6516	1451.6447
Red. masses --	1.7416	1.5371	1.1006
Frc consts --	1.9946	1.8510	1.3665
IR Inten --	24.3306	6.9952	20.4850

	31	32	33
	A	A	A
Frequencies --	1488.4705	1503.1364	1512.4039
Red. masses --	1.2187	1.1644	1.0998
Frc consts --	1.5908	1.5501	1.4821
IR Inten --	21.2761	1.2389	9.1981

	34	35	36
	A	A	A
Frequencies --	1819.5297	2974.8504	2977.9718
Red. masses --	11.9733	1.0712	1.0727
Frc consts --	23.3550	5.5855	5.6051
IR Inten --	452.1956	168.8958	38.2289

	37	38	39
	A	A	A
Frequencies --	3067.3619	3087.4870	3124.7998
Red. masses --	1.0640	1.0831	1.0915
Frc consts --	5.8983	6.0834	6.2792
IR Inten --	8.2688	24.1019	35.8851

	40	41	42
	A	A	A
Frequencies --	3130.7333	3145.4644	3599.0264
Red. masses --	1.0929	1.1007	1.0755
Frc consts --	6.3111	6.4164	8.2081
IR Inten --	26.7025	17.4985	14.9887

17a

	1	2	3
	A	A	A
Frequencies --	75.2394	168.0481	233.8647
Red. masses --	5.6334	3.6161	3.8040
Frc consts --	0.0188	0.0602	0.1226
IR Inten --	4.7393	0.5918	5.1823

	4	5	6
	A	A	A
Frequencies --	271.3886	351.5573	395.1949
Red. masses --	5.0030	3.1951	2.1854
Frc consts --	0.2171	0.2327	0.2011
IR Inten --	2.4994	5.2923	8.3899

	7	8	9
	A	A	A
Frequencies --	410.9961	488.0010	607.5585
Red. masses --	5.2961	2.1357	3.0491
Frc consts --	0.5271	0.2997	0.6631
IR Inten --	12.6165	34.3966	89.0160

```
                     10                     11                     12
                      A                      A                      A
Frequencies --    656.9512               750.0439               808.7467
Red. masses --      3.1784                 2.9337                 1.7460
Frc consts  --      0.8082                 0.9724                 0.6728
IR Inten    --     54.4969                42.1806                59.8140




                     13                     14                     15
                      A                      A                      A
Frequencies --    880.6099               890.9969               946.2046
Red. masses --      2.7661                 2.5387                 2.2683
Frc consts  --      1.2638                 1.1875                 1.1965
IR Inten    --     52.4099                31.5952                73.8803

                     16                     17                     18
                      A                      A                      A
Frequencies --   1000.7279              1032.4086              1063.3076
Red. masses --      3.1287                 1.9821                 2.0661
Frc consts  --      1.8461                 1.2448                 1.3763
IR Inten    --      1.8127                46.9530                40.4844

                     19                     20                     21
                      A                      A                      A
Frequencies --   1077.6979              1165.0574              1179.1451
Red. masses --      2.0023                 2.0288                 1.8788
Frc consts  --      1.3702                 1.6225                 1.5391
IR Inten    --     55.2734                 7.4589                36.5623

                     22                     23                     24
                      A                      A                      A
Frequencies --   1201.5824              1243.7561              1260.6715
Red. masses --      1.7296                 1.9810                 1.4383
Frc consts  --      1.4713                 1.8056                 1.3468
IR Inten    --     33.1330               100.5830                 9.6246

                     25                     26                     27
                      A                      A                      A
Frequencies --   1300.3583              1327.0086              1342.6027
Red. masses --      1.3442                 1.2371                 1.4273
Frc consts  --      1.3391                 1.2836                 1.5159
IR Inten    --     58.2012                 6.4613                29.7017

                     28                     29                     30
                      A                      A                      A
Frequencies --   1367.7157              1393.2839              1431.5844
Red. masses --      1.4943                 1.8020                 1.5139
Frc consts  --      1.6470                 2.0610                 1.8280
IR Inten    --     23.6929                88.1165                 2.8706

                     31                     32                     33
                      A                      A                      A
Frequencies --   1463.7892              1480.0236              1491.2055
Red. masses --      1.1635                 1.0834                 1.3250
Frc consts  --      1.4689                 1.3983                 1.7360
IR Inten    --     17.0119                10.6094                27.3211

                     34                     35                     36
                      A                      A                      A
Frequencies --   1509.6874              2969.1682              2973.2002
Red. masses --      1.1133                 1.0713                 1.0724
Frc consts  --      1.4950                 5.5643                 5.5855
IR Inten    --      7.3699               148.8308                48.0028

                     37                     38                     39
                      A                      A                      A
Frequencies --   3064.4568              3121.1085              3124.2119
Red. masses --      1.0646                 1.0910                 1.0918
Frc consts  --      5.8905                 6.2614                 6.2788
IR Inten    --      8.1161                25.3686                38.0189

                     40                     41                     42
                      A                      A                      A
```

Frequencies --	3142.5974	3155.0557	3601.0029
Red. masses --	1.0889	1.0999	1.0753
Frc consts --	6.3358	6.4510	8.2156
IR Inten --	22.6269	11.3070	16.3572

17e

	1	2	3
	A	A	A
Frequencies --	78.5891	169.1511	241.0245
Red. masses --	4.9094	2.7073	13.3638
Frc consts --	0.0179	0.0456	0.4574
IR Inten --	0.7963	4.9081	2.4459
	4	5	6
	A	A	A
Frequencies --	315.9817	363.4978	376.8533
Red. masses --	3.0067	3.4789	2.8264
Frc consts --	0.1769	0.2708	0.2365
IR Inten --	5.5043	17.9600	2.6516
	7	8	9
	A	A	A
Frequencies --	438.0018	482.0726	577.3692
Red. masses --	3.3863	3.2243	4.0371
Frc consts --	0.3828	0.4415	0.7929
IR Inten --	2.6121	11.7421	18.7665
	10	11	12
	A	A	A
Frequencies --	604.9038	741.9473	789.7241
Red. masses --	2.5222	3.2336	1.5446
Frc consts --	0.5437	1.0488	0.5676
IR Inten --	115.0552	0.7070	117.3936
	13	14	15
	A	A	A
Frequencies --	858.7996	908.8358	998.5530
Red. masses --	2.1990	2.6208	1.8151
Frc consts --	0.9556	1.2754	1.0663
IR Inten --	41.1919	5.3274	21.9123
	16	17	18
	A	A	A
Frequencies --	1012.1836	1068.5425	1095.0340
Red. masses --	1.9971	2.0256	2.7310
Frc consts --	1.2055	1.3627	1.9294
IR Inten --	8.8851	56.0291	12.4579
	19	20	21
	A	A	A
Frequencies --	1106.6425	1149.3040	1182.2280
Red. masses --	3.3239	2.5132	1.7968
Frc consts --	2.3983	1.9559	1.4796
IR Inten --	77.4931	39.1814	5.5317
	22	23	24
	A	A	A
Frequencies --	1200.1204	1213.1709	1279.7905
Red. masses --	1.5647	1.7796	1.4390
Frc consts --	1.3278	1.5432	1.3887
IR Inten --	23.2988	70.1934	6.5941
	25	26	27
	A	A	A
Frequencies --	1317.2837	1327.1706	1336.9859
Red. masses --	1.2887	1.4819	1.3197
Frc consts --	1.3176	1.5379	1.3898
IR Inten --	1.5511	89.8422	37.6396
	28	29	30

	A	A	A
Frequencies --	1359.9798	1390.3095	1430.8320
Red. masses --	1.8688	1.5146	1.5114
Frc consts --	2.0364	1.7250	1.8230
IR Inten --	104.0990	22.9347	6.7014
	31	32	33
	A	A	A
Frequencies --	1464.1085	1487.0208	1505.8058
Red. masses --	1.1202	1.2701	1.1207
Frc consts --	1.4148	1.6547	1.4972
IR Inten --	19.5981	31.4133	1.5078
	34	35	36
	A	A	A
Frequencies --	1517.3558	2977.5229	2982.1503
Red. masses --	1.1007	1.0705	1.0723
Frc consts --	1.4931	5.5918	5.6188
IR Inten --	10.8948	123.0110	69.9992
	37	38	39
	A	A	A
Frequencies --	3055.4328	3082.7464	3127.4077
Red. masses --	1.0667	1.0827	1.0920
Frc consts --	5.8675	6.0624	6.2928
IR Inten --	8.6506	23.6836	37.6837
	40	41	42
	A	A	A
Frequencies --	3128.6731	3152.9645	3599.2289
Red. masses --	1.0930	1.0978	1.0754
Frc consts --	6.3037	6.4298	8.2081
IR Inten --	28.9083	12.8817	17.2093

**18a**	1	2	3
	A	A	A
Frequencies --	98.5574	176.3633	232.6496
Red. masses --	4.2638	3.3324	4.3739
Frc consts --	0.0244	0.0611	0.1395
IR Inten --	5.9343	0.3523	5.6333
	4	5	6
	A	A	A
Frequencies --	303.5284	392.6514	430.6830
Red. masses --	3.1600	1.9078	3.9428
Frc consts --	0.1715	0.1733	0.4309
IR Inten --	1.7636	11.4103	25.0983
	7	8	9
	A	A	A
Frequencies --	465.3115	501.3814	637.6232
Red. masses --	3.7637	2.1107	3.3336
Frc consts --	0.4801	0.3126	0.7985
IR Inten --	21.6508	26.7530	44.2562
	10	11	12
	A	A	A
Frequencies --	677.9673	780.4526	816.9266
Red. masses --	2.3488	3.2370	1.7022
Frc consts --	0.6361	1.1617	0.6693
IR Inten --	64.6384	53.3993	54.1450
	13	14	15
	A	A	A
Frequencies --	885.5679	904.4906	925.2274
Red. masses --	2.1820	2.2301	1.8247
Frc consts --	1.0082	1.0749	0.9203
IR Inten --	21.1430	57.6321	194.8969
	16	17	18
	A	A	A
Frequencies --	966.4156	1015.7664	1054.7709
Red. masses --	1.6945	3.3859	2.0038
Frc consts --	0.9324	2.0583	1.3135
IR Inten --	4.6837	5.6181	32.4885

```
                       19                     20                     21
                        A                      A                      A
 Frequencies --   1075.1120              1100.6745              1144.0807
 Red. masses --      1.6872                 2.1970                 1.6801
 Frc consts  --      1.1490                 1.5682                 1.2957
 IR Inten    --      2.4980                25.4395                73.6119
```

```
                       22                     23                     24
                        A                      A                      A
 Frequencies --   1191.5380              1214.4293              1224.1648
 Red. masses --      2.6499                 1.2106                 1.0928
 Frc consts  --      2.2166                 1.0520                 0.9649
 IR Inten    --     20.7688                19.8531                11.9498

                       25                     26                     27
                        A                      A                      A
 Frequencies --   1304.8704              1333.0554              1340.2981
 Red. masses --      1.3047                 1.4545                 1.3377
 Frc consts  --      1.3089                 1.5228                 1.4159
 IR Inten    --     16.5401                31.0417                 9.6287

                       28                     29                     30
                        A                      A                      A
 Frequencies --   1370.0186              1372.3802              1420.1812
 Red. masses --      1.4410                 1.2283                 2.0126
 Frc consts  --      1.5935                 1.3631                 2.3916
 IR Inten    --     20.7550                22.2602                26.5583

                       31                     32                     33
                        A                      A                      A
 Frequencies --   1427.5394              1456.3071              1477.2475
 Red. masses --      1.5220                 1.1519                 1.0857
 Frc consts  --      1.8274                 1.4394                 1.3959
 IR Inten    --     14.2420                24.3543                10.0256

                       34                     35                     36
                        A                      A                      A
 Frequencies --   1495.1338              1512.3547              1754.0766
 Red. masses --      1.3324                 1.1149                 9.2383
 Frc consts  --      1.7549                 1.5024                16.7471
 IR Inten    --     20.6589                 4.8476               149.8616

                       37                     38                     39
                        A                      A                      A
 Frequencies --   2960.8979              2964.2164              3069.0019
 Red. masses --      1.0713                 1.0731                 1.0637
 Frc consts  --      5.5336                 5.5556                 5.9031
 IR Inten    --    159.9199                58.4093                17.1959

                       40                     41                     42
                        A                      A                      A
 Frequencies --   3108.1234              3117.9472              3119.6064
 Red. masses --      1.0882                 1.0903                 1.0911
 Frc consts  --      6.1939                 6.2449                 6.2562
 IR Inten    --     38.5415                60.9070                17.1925

                       43                     44                     45
                        A                      A                      A
 Frequencies --   3143.3996              3485.7530              3596.1655
 Red. masses --      1.1009                 1.0745                 1.0754
 Frc consts  --      6.4090                 7.6920                 8.1940
 IR Inten    --     21.7348                 1.0410                10.3131
```

18e

```
                        1                      2                      3
                        A                      A                      A
 Frequencies --    106.1149               170.8066               289.3884
 Red. masses --      4.1074                 2.5023                 4.8794
 Frc consts  --      0.0273                 0.0430                 0.2408
 IR Inten    --      1.0248                 4.7275                13.0926
```

```
                      4                      5                      6
                      A                      A                      A
 Frequencies --    345.1997               361.6299               399.2877
 Red. masses --      2.7528                 3.0081                 3.9805
 Frc consts  --      0.1933                 0.2318                 0.3739
 IR Inten    --      0.2363                26.8086                 1.8762




                      7                      8                      9
                      A                      A                      A
 Frequencies --    439.1426               505.8509               593.2765
 Red. masses --      3.0667                 2.4360                 3.6310
 Frc consts  --      0.3484                 0.3673                 0.7530
 IR Inten    --      0.6741                39.7133                32.5542

                     10                     11                     12
                      A                      A                      A
 Frequencies --    663.9893               776.4378               799.0367
 Red. masses --      2.5044                 2.4766                 1.5348
 Frc consts  --      0.6505                 0.8797                 0.5773
 IR Inten    --     61.9836                 7.1691               109.5661

                     13                     14                     15
                      A                      A                      A
 Frequencies --    891.1364               904.1611               926.1839
 Red. masses --      2.5391                 1.7474                 1.4062
 Frc consts  --      1.1880                 0.8417                 0.7107
 IR Inten    --    105.0988                15.3001                56.4240

                     16                     17                     18
                      A                      A                      A
 Frequencies --   1002.1877              1047.1241              1079.1086
 Red. masses --      2.4056                 1.6514                 3.1552
 Frc consts  --      1.4235                 1.0669                 2.1647
 IR Inten    --     10.9704                31.5000               105.7863

                     19                     20                     21
                      A                      A                      A
 Frequencies --   1093.9694              1113.3750              1130.8376
 Red. masses --      1.8371                 2.2914                 2.4473
 Frc consts  --      1.2954                 1.6736                 1.8439
 IR Inten    --     10.3720                16.1220                20.4044

                     22                     23                     24
                      A                      A                      A
 Frequencies --   1186.7205              1208.8801              1234.7534
 Red. masses --      3.0192                 1.1158                 1.2164
 Frc consts  --      2.5052                 0.9607                 1.0926
 IR Inten    --     16.7912                12.9500                24.5346

                     25                     26                     27
                      A                      A                      A
 Frequencies --   1296.9597              1323.1887              1327.3517
 Red. masses --      1.3658                 1.3143                 1.2040
 Frc consts  --      1.3536                 1.3558                 1.2498
 IR Inten    --      5.6245                19.7092                36.1985

                     28                     29                     30
                      A                      A                      A
 Frequencies --   1356.3177              1363.6114              1418.7484
 Red. masses --      1.3146                 1.3821                 1.8181
 Frc consts  --      1.4248                 1.5141                 2.1562
 IR Inten    --      8.3790                 6.5772                46.5153

                     31                     32                     33
                      A                      A                      A
 Frequencies --   1431.9769              1456.9132              1488.2101
 Red. masses --      1.6699                 1.1254                 1.2153
 Frc consts  --      2.0174                 1.4074                 1.5858
 IR Inten    --      0.7484                21.4891                19.0896

                     34                     35                     36
```

	A	A	A
Frequencies --	1503.2162	1512.0554	1752.8920
Red. masses --	1.1761	1.1008	10.0780
Frc consts --	1.5657	1.4828	18.2447
IR Inten --	1.5054	9.8283	193.7460

	37	38	39
	A	A	A
Frequencies --	2969.1009	2975.3417	3065.5982
Red. masses --	1.0720	1.0715	1.0646
Frc consts --	5.5680	5.5885	5.8947
IR Inten --	142.3483	72.6163	16.7645

	40	41	42
	A	A	A
Frequencies --	3095.2558	3120.4120	3128.3643
Red. masses --	1.0841	1.0912	1.0930
Frc consts --	6.1196	6.2603	6.3021
IR Inten --	26.7593	43.7679	34.6105

	43	44	45
	A	A	A
Frequencies --	3142.0753	3489.7448	3600.6973
Red. masses --	1.1001	1.0733	1.0754
Frc consts --	6.3993	7.7012	8.2150
IR Inten --	22.0670	8.5588	11.9594

19a

	1	2	3
	A	A	A
Frequencies --	95.9087	176.3350	229.7553
Red. masses --	4.3121	3.4350	3.9290
Frc consts --	0.0234	0.0629	0.1222
IR Inten --	6.3540	0.1746	4.2252

	4	5	6
	A	A	A
Frequencies --	304.3152	396.9740	430.5785
Red. masses --	3.1925	1.9189	4.7064
Frc consts --	0.1742	0.1782	0.5141
IR Inten --	5.3571	6.8540	6.9662

	7	8	9
	A	A	A
Frequencies --	463.5558	499.8030	642.0673
Red. masses --	3.5173	2.0436	3.2518
Frc consts --	0.4453	0.3008	0.7898
IR Inten --	58.6913	2.6960	31.8184

	10	11	12
	A	A	A
Frequencies --	681.4167	777.6253	817.9302
Red. masses --	2.4754	3.1809	1.8354
Frc consts --	0.6772	1.1333	0.7234
IR Inten --	86.8235	32.4521	57.6128

	13	14	15
	A	A	A
Frequencies --	887.0657	904.5395	923.4142
Red. masses --	2.1505	2.1784	1.7121
Frc consts --	0.9970	1.0501	0.8602
IR Inten --	93.1472	21.5831	14.2074

	16	17	18
	A	A	A
Frequencies --	965.8731	1014.8957	1055.1643
Red. masses --	1.6985	3.4615	2.0355
Frc consts --	0.9336	2.1006	1.3353
IR Inten --	70.5563	2.2051	143.8736

	19	20	21
	A	A	A
Frequencies --	1075.3008	1101.2357	1147.3333
Red. masses --	1.6891	2.2214	1.6614
Frc consts --	1.1507	1.5873	1.2886
IR Inten --	1.8744	8.2834	23.2982

	22	23	24
	A	A	A
Frequencies --	1192.0122	1217.3399	1232.3950
Red. masses --	2.7518	1.1838	1.1019
Frc consts --	2.3037	1.0336	0.9860
IR Inten --	30.7409	7.8880	14.2410

	25	26	27
	A	A	A
Frequencies --	1310.9243	1329.9354	1357.7225
Red. masses --	1.2699	1.4230	1.3351
Frc consts --	1.2858	1.4829	1.4501
IR Inten --	7.7715	21.0768	20.4753
	28	29	30
	A	A	A
Frequencies --	1362.2169	1379.3215	1424.3438
Red. masses --	1.4306	1.2275	2.3970
Frc consts --	1.5641	1.3759	2.8652
IR Inten --	4.8035	30.3094	82.3813
	31	32	33
	A	A	A
Frequencies --	1436.2550	1455.3627	1479.3825
Red. masses --	1.5083	1.1025	1.0858
Frc consts --	1.8332	1.3758	1.4001
IR Inten --	15.8055	20.5093	9.9435
	34	35	36
	A	A	A
Frequencies --	1499.2567	1515.4865	1756.3504
Red. masses --	1.3290	1.1171	9.2583
Frc consts --	1.7601	1.5116	16.8269
IR Inten --	20.1041	5.7157	151.2755
	37	38	39
	A	A	A
Frequencies --	2962.6715	2966.7669	3069.7915
Red. masses --	1.0713	1.0729	1.0613
Frc consts --	5.5404	5.5638	5.8925
IR Inten --	173.9712	45.2391	16.3049
	40	41	42
	A	A	A
Frequencies --	3114.2488	3119.9403	3129.4644
Red. masses --	1.0909	1.0905	1.0922
Frc consts --	6.2334	6.2540	6.3021
IR Inten --	48.7040	24.4340	37.2451
	43	44	45
	A	A	A
Frequencies --	3131.9046	3488.3956	3599.6138
Red. masses --	1.1000	1.0744	1.0754
Frc consts --	6.3574	7.7029	8.2101
IR Inten --	19.5578	1.5644	10.5523

19e

	1	2	3
	A	A	A
Frequencies --	96.3852	171.1527	296.3543
Red. masses --	4.3375	2.5135	5.3722
Frc consts --	0.0237	0.0434	0.2780
IR Inten --	1.9117	7.2058	0.8374
	4	5	6
	A	A	A
Frequencies --	346.2757	359.8987	401.7152
Red. masses --	2.7775	2.8579	3.9031
Frc consts --	0.1962	0.2181	0.3711
IR Inten --	4.8326	12.5869	15.2707

```
                      7                      8                      9
                      A                      A                      A
Frequencies --    438.2161               509.9484               590.0768
Red. masses --      3.1053                 2.4576                 3.5132
Frc consts  --      0.3513                 0.3765                 0.7207
IR Inten    --      4.3225                 8.0917                57.3120

                     10                     11                     12
                      A                      A                      A
Frequencies --    664.3587               782.3249               799.6645
Red. masses --      2.5494                 2.3463                 1.5713
Frc consts  --      0.6630                 0.8461                 0.5920
IR Inten    --     61.3249                 8.8078               103.4305
                     13                     14                     15
                      A                      A                      A
Frequencies --    890.9432               901.0712               922.9936
Red. masses --      2.1427                 1.6086                 1.8272
Frc consts  --      1.0021                 0.7695                 0.9171
IR Inten    --     35.0445                76.8579                10.3115
                     16                     17                     18
                      A                      A                      A
Frequencies --   1001.9957              1048.4477              1090.7862
Red. masses --      2.3106                 1.6564                 3.3599
Frc consts  --      1.3668                 1.0728                 2.3554
IR Inten    --     44.5581                29.4039               105.3078
                     19                     20                     21
                      A                      A                      A
Frequencies --   1093.6625              1112.8941              1129.8906
Red. masses --      1.8956                 2.3034                 2.4038
Frc consts  --      1.3358                 1.6809                 1.8081
IR Inten    --     25.3880                15.8193                69.1894
                     22                     23                     24
                      A                      A                      A
Frequencies --   1188.1778              1209.3568              1227.6283
Red. masses --      3.0700                 1.1185                 1.1422
Frc consts  --      2.5536                 0.9639                 1.0142
IR Inten    --     23.8190                 7.1211                14.2093
                     25                     26                     27
                      A                      A                      A
Frequencies --   1296.5669              1327.8142              1340.9777
Red. masses --      1.3711                 1.2152                 1.3136
Frc consts  --      1.3581                 1.2623                 1.3917
IR Inten    --     11.1393                14.8388                27.9009
                     28                     29                     30
                      A                      A                      A
Frequencies --   1357.0720              1365.8938              1422.6477
Red. masses --      1.2802                 1.4158                 1.7102
Frc consts  --      1.3891                 1.5562                 2.0393
IR Inten    --     17.4872                15.7494                 5.2701
                     31                     32                     33
                      A                      A                      A
Frequencies --   1437.6379              1459.3667              1488.9111
Red. masses --      1.7566                 1.1130                 1.2195
Frc consts  --      2.1391                 1.3966                 1.5929
IR Inten    --     22.1801                22.9509                20.3461
                     34                     35                     36
                      A                      A                      A
Frequencies --   1503.3755              1512.5509              1755.7493
Red. masses --      1.1750                 1.1022                 9.5582
Frc consts  --      1.5647                 1.4857                17.3600
IR Inten    --      1.8854                 9.7483               186.9804
                     37                     38                     39
```

```
                          A                      A                      A
 Frequencies --   2969.4805              2972.9608              3064.3447
 Red. masses --      1.0716                 1.0719                 1.0628
 Frc consts  --      5.5672                 5.5818                 5.8797
 IR Inten    --    162.0711                53.8806                13.7406

                         40                     41                     42
                          A                      A                      A
 Frequencies --   3082.8832              3120.2141              3127.1153
 Red. masses --      1.0836                 1.0911                 1.0929
 Frc consts  --      6.0679                 6.2585                 6.2966
 IR Inten    --     33.2838                44.5607                39.0423

                         43                     44                     45
                          A                      A                      A
 Frequencies --   3129.4905              3498.2651              3597.9303
 Red. masses --      1.1025                 1.0745                 1.0754
 Frc consts  --      6.3615                 7.7475                 8.2022
 IR Inten    --     22.0830                 2.1388                11.6267
```

20a

```
                          1                      2                      3
                          A                      A                      A
 Frequencies --    108.1769               180.4559               232.8482
 Red. masses --      3.4378                 3.4239                 3.6207
 Frc consts  --      0.0237                 0.0657                 0.1157
 IR Inten    --      1.4152                 1.3939                 4.0610

                          4                      5                      6
                          A                      A                      A
 Frequencies --    304.6629               384.8590               407.0704
 Red. masses --      2.9199                 2.1725                 2.2050
 Frc consts  --      0.1597                 0.1896                 0.2153
 IR Inten    --      1.6370                 7.1088                 5.2499

                          7                      8                      9
                          A                      A                      A
 Frequencies --    456.7423               490.1165               637.8266
 Red. masses --      4.1950                 2.0225                 4.1437
 Frc consts  --      0.5156                 0.2862                 0.9932
 IR Inten    --     32.0787                15.3325                 7.7638

                         10                     11                     12
                          A                      A                      A
 Frequencies --    663.9272               744.7859               796.5375
 Red. masses --      2.0861                 1.2239                 2.5431
 Frc consts  --      0.5418                 0.4000                 0.9507
 IR Inten    --     80.9647                22.5081                20.4717

                         13                     14                     15
                          A                      A                      A
 Frequencies --    817.5590               877.9035               907.8791
 Red. masses --      1.6966                 2.7927                 2.2664
 Frc consts  --      0.6681                 1.2681                 1.1006
 IR Inten    --     79.7964               122.3383                 4.8148

                         16                     17                     18
                          A                      A                      A
 Frequencies --    933.6161               970.2023              1003.5943
 Red. masses --      1.9262                 1.3606                 2.3370
 Frc consts  --      0.9892                 0.7546                 1.3868
 IR Inten    --     66.8100                61.4158                16.6451

                         19                     20                     21
                          A                      A                      A
 Frequencies --   1019.0741              1042.8745              1084.5617
 Red. masses --      2.2131                 1.8926                 1.7503
 Frc consts  --      1.3541                 1.2127                 1.2130
 IR Inten    --     20.8896                64.3217                 1.5403

                         22                     23                     24
                          A                      A                      A
 Frequencies --   1097.7852              1191.6395              1218.9651
 Red. masses --      1.9988                 2.7498                 1.3147
 Frc consts  --      1.4192                 2.3006                 1.1510
 IR Inten    --     12.1565                26.3817                11.6218
```

```
                     25                     26                     27
                      A                      A                      A
 Frequencies --   1230.6690              1310.1667              1321.8546
 Red. masses --      1.1031                 1.3574                 1.4293
 Frc consts  --      0.9844                 1.3728                 1.4714
 IR Inten    --      6.8346                 5.0903                 6.2803
```

```
                     28                     29                     30
                      A                      A                      A
 Frequencies --   1337.7112              1362.4806              1372.1472
 Red. masses --      1.5839                 1.2999                 1.2666
 Frc consts  --      1.6699                 1.4217                 1.4050
 IR Inten    --     41.4524                 4.8895                21.8933

                     31                     32                     33
                      A                      A                      A
 Frequencies --   1386.4081              1435.8092              1455.7879
 Red. masses --      1.8647                 1.5392                 1.1355
 Frc consts  --      2.1117                 1.8695                 1.4179
 IR Inten    --     13.3376                 6.2945                11.5134

                     34                     35                     36
                      A                      A                      A
 Frequencies --   1472.8456              1479.1409              1498.9478
 Red. masses --      1.1532                 1.0871                 1.3190
 Frc consts  --      1.4739                 1.4014                 1.7461
 IR Inten    --     21.5979                10.1807                19.9902

                     37                     38                     39
                      A                      A                      A
 Frequencies --   1510.4079              1744.1536              2956.3823
 Red. masses --      1.1314                 6.0201                 1.0719
 Frc consts  --      1.5208                10.7901                 5.5198
 IR Inten    --      5.1293                19.9038               161.3424

                     40                     41                     42
                      A                      A                      A
 Frequencies --   2963.2847              3061.2014              3107.4166
 Red. masses --      1.0722                 1.0632                 1.0885
 Frc consts  --      5.5470                 5.8701                 6.1926
 IR Inten    --     74.5732                31.6745                65.8404

                     43                     44                     45
                      A                      A                      A
 Frequencies --   3108.3320              3115.5641              3123.5269
 Red. masses --      1.0903                 1.0903                 1.1011
 Frc consts  --      6.2066                 6.2353                 6.3294
 IR Inten    --     35.1044                48.1097                31.0113

                     46                     47                     48
                      A                      A                      A
 Frequencies --   3146.7088              3235.5986              3593.4411
 Red. masses --      1.0621                 1.1165                 1.0753
 Frc consts  --      6.1965                 6.8867                 8.1810
 IR Inten    --     12.6502                16.0845                 7.3498
```

20e

```
                      1                      2                      3
                      A                      A                      A
 Frequencies --    110.4868               183.9585               286.8028
 Red. masses --      3.4140                 2.5219                 3.7903
 Frc consts  --      0.0246                 0.0503                 0.1837
 IR Inten    --      1.6220                 2.8809                 7.0574

                      4                      5                      6
                      A                      A                      A
 Frequencies --    338.9542               363.0867               391.8364
 Red. masses --      2.5885                 2.8866                 3.0426
 Frc consts  --      0.1752                 0.2242                 0.2752
 IR Inten    --      5.7854                15.2046                 5.0055
```

```
                      7                      8                      9
                      A                      A                      A
Frequencies --    441.3262               481.7671               570.0411
Red. masses --      2.9217                 2.6757                 3.1357
Frc consts  --      0.3353                 0.3659                 0.6003
IR Inten    --      0.5401                14.9469                33.8020



                     10                     11                     12
                      A                      A                      A
Frequencies --    643.6523               747.4837               793.4869
Red. masses --      2.3017                 1.1757                 1.6601
Frc consts  --      0.5618                 0.3870                 0.6158
IR Inten    --     46.3186                 1.9665               108.3991

                     13                     14                     15
                      A                      A                      A
Frequencies --    805.7960               876.8060               912.3015
Red. masses --      1.8878                 2.0429                 2.2964
Frc consts  --      0.7222                 0.9254                 1.1261
IR Inten    --     28.1319                33.5079                 8.3803

                     16                     17                     18
                      A                      A                      A
Frequencies --    950.7733               976.0127              1025.8350
Red. masses --      1.3515                 2.1021                 1.7219
Frc consts  --      0.7198                 1.1798                 1.0676
IR Inten    --     81.3671                47.0811                14.0243

                     19                     20                     21
                      A                      A                      A
Frequencies --   1040.3212              1073.1870              1104.0110
Red. masses --      1.9218                 3.1792                 2.0291
Frc consts  --      1.2254                 2.1573                 1.4571
IR Inten    --     25.1976                96.9763                 9.2264

                     22                     23                     24
                      A                      A                      A
Frequencies --   1120.5356              1185.2506              1205.1228
Red. masses --      2.5057                 3.4241                 1.3787
Frc consts  --      1.8537                 2.8341                 1.1798
IR Inten    --     14.6218                24.4393                 4.2033

                     25                     26                     27
                      A                      A                      A
Frequencies --   1211.4239              1285.3684              1301.1501
Red. masses --      1.0935                 1.4955                 1.3446
Frc consts  --      0.9455                 1.4558                 1.3412
IR Inten    --      5.9239                 3.4419                 8.3437

                     28                     29                     30
                      A                      A                      A
Frequencies --   1324.9444              1357.3929              1362.1809
Red. masses --      1.2403                 1.3424                 1.3587
Frc consts  --      1.2828                 1.4573                 1.4855
IR Inten    --     11.9805                14.7614                12.6908

                     31                     32                     33
                      A                      A                      A
Frequencies --   1398.4899              1427.2604              1444.5897
Red. masses --      1.7888                 1.5363                 1.1632
Frc consts  --      2.0612                 1.8439                 1.4302
IR Inten    --      1.9548                 5.0577                 6.5307

                     34                     35                     36
                      A                      A                      A
Frequencies --   1468.0075              1486.5977              1501.6916
Red. masses --      1.1265                 1.2217                 1.1753
Frc consts  --      1.4304                 1.5908                 1.5615
IR Inten    --     21.4869                18.3698                 2.2271

                     37                     38                     39
```

	A	A	A
Frequencies --	1510.4325	1743.5528	2963.3107
Red. masses --	1.1021	6.3491	1.0720
Frc consts --	1.4813	11.3718	5.5462
IR Inten --	10.0978	39.0984	141.7087

	40	41	42
	A	A	A
Frequencies --	2971.0485	3050.8789	3076.5242
Red. masses --	1.0707	1.0647	1.0840
Frc consts --	5.5685	5.8391	6.0448
IR Inten --	89.5050	33.9460	47.1533

	43	44	45
	A	A	A
Frequencies --	3113.0692	3115.8407	3120.8664
Red. masses --	1.0911	1.0932	1.0995
Frc consts --	6.2298	6.2534	6.3096
IR Inten --	59.6075	50.9173	31.9175

	46	47	48
	A	A	A
Frequencies --	3159.1809	3252.0596	3594.0723
Red. masses --	1.0615	1.1162	1.0754
Frc consts --	6.2418	6.9553	8.1846
IR Inten --	10.2554	8.8907	8.6728

21a

	1	2	3
	A	A	A
Frequencies --	86.8233	149.4038	231.7497
Red. masses --	5.4480	3.1658	4.2457
Frc consts --	0.0242	0.0416	0.1344
IR Inten --	10.7418	0.9557	8.7811

	4	5	6
	A	A	A
Frequencies --	283.9763	371.9860	432.2632
Red. masses --	2.5804	2.0436	4.6455
Frc consts --	0.1226	0.1666	0.5114
IR Inten --	3.3905	2.3458	4.8511

	7	8	9
	A	A	A
Frequencies --	466.5274	499.2205	620.2244
Red. masses --	2.6932	2.8907	3.3256
Frc consts --	0.3454	0.4245	0.7537
IR Inten --	8.1076	21.4316	15.2341

	10	11	12
	A	A	A
Frequencies --	706.8278	763.3791	823.3109
Red. masses --	2.6880	2.9599	1.6576
Frc consts --	0.7912	1.0163	0.6620
IR Inten --	4.6705	9.2528	10.7121

	13	14	15
	A	A	A
Frequencies --	854.1750	901.0867	935.7361
Red. masses --	2.6234	2.0258	1.6687
Frc consts --	1.1278	0.9691	0.8609
IR Inten --	1.4877	9.4194	24.7130

	16	17	18
	A	A	A
Frequencies --	973.9026	1009.4125	1040.9596
Red. masses --	2.7459	2.9513	1.8466
Frc consts --	1.5345	1.7718	1.1789
IR Inten --	108.1907	18.5229	10.5826

	19	20	21
	A	A	A
Frequencies --	1089.5437	1104.5368	1150.9598
Red. masses --	1.5606	2.2671	1.3953
Frc consts --	1.0915	1.6296	1.0890
IR Inten --	4.7017	31.4985	21.9949

	22	23	24
	A	A	A
Frequencies --	1173.0656	1248.0332	1281.2863
Red. masses --	1.5355	1.5865	1.3140
Frc consts --	1.2450	1.4560	1.2709
IR Inten --	5.7635	10.0862	7.3673

	25	26	27
	A	A	A
Frequencies --	1293.9946	1344.3044	1351.5879
Red. masses --	1.3933	1.3725	1.5424
Frc consts --	1.3746	1.4613	1.6602
IR Inten --	11.3578	1.3119	31.2666

	28	29	30
	A	A	A
Frequencies --	1362.3801	1379.8672	1392.2189
Red. masses --	1.3655	1.5526	1.5043
Frc consts --	1.4933	1.7417	1.7179
IR Inten --	8.4457	5.1427	1.5624

	31	32	33
	A	A	A
Frequencies --	1395.8165	1460.7763	1468.1764
Red. masses --	1.4924	1.1076	1.0956
Frc consts --	1.7132	1.3925	1.3914
IR Inten --	4.5255	16.3335	21.8913

	34	35	36
	A	A	A
Frequencies --	1495.8910	1505.0115	1815.6586
Red. masses --	1.0825	1.0956	11.1350
Frc consts --	1.4272	1.4621	21.6277
IR Inten --	10.4449	6.7510	427.1177

	37	38	39
	A	A	A
Frequencies --	3049.9600	3060.1889	3063.8606
Red. masses --	1.0669	1.0647	1.0627
Frc consts --	5.8476	5.8746	5.8776
IR Inten --	36.4121	18.0517	38.7588

	40	41	42
	A	A	A
Frequencies --	3067.6498	3110.3821	3113.5405
Red. masses --	1.0633	1.0975	1.0995
Frc consts --	5.8955	6.2557	6.2797
IR Inten --	14.5974	45.8965	37.0542

	43	44	45
	A	A	A
Frequencies --	3118.8574	3133.9592	3146.2600
Red. masses --	1.1041	1.0887	1.1009
Frc consts --	6.3275	6.3002	6.4206
IR Inten --	50.2780	36.3890	22.6423

21e

	1	2	3
	A	A	A
Frequencies --	93.6415	151.8839	285.2133
Red. masses --	5.0778	2.4923	6.6345
Frc consts --	0.0262	0.0339	0.3180
IR Inten --	2.4491	4.7246	1.8888

	4	5	6
	A	A	A
Frequencies --	312.9156	350.4984	396.1428
Red. masses --	1.9392	3.5559	4.6822
Frc consts --	0.1119	0.2574	0.4329
IR Inten --	0.6799	9.4691	3.8797

```
                      7                      8                      9
                      A                      A                      A
Frequencies --    426.2739               510.2743               600.5536
Red. masses --      2.9446                 2.2568                 4.1397
Frc consts  --      0.3153                 0.3462                 0.8797
IR Inten    --      1.7769                 3.4934                30.8625
```

```
                     10                     11                     12
                      A                      A                      A
Frequencies --    683.5881               753.7755               851.4823
Red. masses --      2.9175                 2.1718                 1.7649
Frc consts  --      0.8032                 0.7270                 0.7539
IR Inten    --      6.9120                 4.9144                21.9883
                     13                     14                     15
                      A                      A                      A
Frequencies --    872.6901               897.5701               928.5386
Red. masses --      2.5195                 1.8167                 1.6892
Frc consts  --      1.1305                 0.8623                 0.8581
IR Inten    --      2.0288                41.8346                15.9450
                     16                     17                     18
                      A                      A                      A
Frequencies --    972.6046              1058.0023              1086.3724
Red. masses --      2.4113                 1.5275                 3.8245
Frc consts  --      1.3439                 1.0074                 2.6594
IR Inten    --      7.6807                22.1193               121.3794
                     19                     20                     21
                      A                      A                      A
Frequencies --   1092.9023              1108.7045              1146.0129
Red. masses --      1.5975                 2.1488                 1.7508
Frc consts  --      1.1243                 1.5562                 1.3548
IR Inten    --      5.0011                48.1646                 9.0831
                     22                     23                     24
                      A                      A                      A
Frequencies --   1159.3087              1249.8628              1270.3171
Red. masses --      2.2904                 1.5036                 1.3810
Frc consts  --      1.8137                 1.3839                 1.3131
IR Inten    --      7.7148                 6.0819                 1.3519
                     25                     26                     27
                      A                      A                      A
Frequencies --   1289.9369              1335.1973              1354.5601
Red. masses --      1.2629                 1.2414                 1.3594
Frc consts  --      1.2381                 1.3040                 1.4696
IR Inten    --      2.5426                 6.9133                 8.1687
                     28                     29                     30
                      A                      A                      A
Frequencies --   1362.9839              1375.1369              1398.7331
Red. masses --      1.4130                 1.5055                 1.4844
Frc consts  --      1.5466                 1.6774                 1.7110
IR Inten    --      4.0906                 7.9444                 2.1807
                     31                     32                     33
                      A                      A                      A
Frequencies --   1406.0719              1463.4075              1488.9209
Red. masses --      1.5771                 1.1010                 1.0960
Frc consts  --      1.8370                 1.3892                 1.4315
IR Inten    --      4.0706                18.7998                 8.9664
                     34                     35                     36
                      A                      A                      A
Frequencies --   1492.8572              1503.5093              1816.2623
Red. masses --      1.0865                 1.0988                11.8787
Frc consts  --      1.4266                 1.4634                23.0875
IR Inten    --     14.1610                 4.7463               461.3508
                     37                     38                     39
```

	A	A	A
Frequencies --	3049.6327	3051.8628	3056.7492
Red. masses --	1.0699	1.0684	1.0637
Frc consts --	5.8625	5.8628	5.8559
IR Inten --	6.7203	18.6634	23.7321
	40	41	42
	A	A	A
Frequencies --	3057.6194	3067.4955	3115.3586
Red. masses --	1.0635	1.0794	1.0965
Frc consts --	5.8579	5.9839	6.2702
IR Inten --	50.5041	46.3620	43.0808
	43	44	45
	A	A	A
Frequencies --	3117.1017	3122.2943	3139.7403
Red. masses --	1.1002	1.1033	1.0982
Frc consts --	6.2982	6.3371	6.3787
IR Inten --	48.2304	46.6919	24.4233

22a

	1	2	3
	A	A	A
Frequencies --	82.9917	150.6587	242.1800
Red. masses --	5.2999	3.3789	3.4482
Frc consts --	0.0215	0.0452	0.1192
IR Inten --	3.1617	1.4603	7.1767
	4	5	6
	A	A	A
Frequencies --	260.4137	339.9125	385.8860
Red. masses --	3.6571	2.8265	2.5893
Frc consts --	0.1461	0.1924	0.2272
IR Inten --	3.0177	0.9343	4.9609
	7	8	9
	A	A	A
Frequencies --	415.4764	470.6482	602.9471
Red. masses --	4.4504	2.0899	3.9939
Frc consts --	0.4526	0.2727	0.8555
IR Inten --	2.7542	3.4146	7.1574
	10	11	12
	A	A	A
Frequencies --	664.3959	743.7529	817.2812
Red. masses --	3.1637	2.6210	1.5874
Frc consts --	0.8228	0.8542	0.6247
IR Inten --	4.5489	6.2474	11.8203
	13	14	15
	A	A	A
Frequencies --	847.7587	888.1191	927.9734
Red. masses --	2.8617	2.2658	1.6759
Frc consts --	1.2118	1.0530	0.8503
IR Inten --	3.2367	23.2925	27.3829
	16	17	18
	A	A	A
Frequencies --	963.9000	994.7225	1041.6424
Red. masses --	2.6543	2.7185	1.9238
Frc consts --	1.4530	1.5849	1.2298
IR Inten --	85.1330	58.9639	2.4027
	19	20	21
	A	A	A
Frequencies --	1063.5559	1088.3038	1138.9317
Red. masses --	1.9360	1.6371	1.5654
Frc consts --	1.2903	1.1424	1.1964
IR Inten --	85.2132	11.6241	18.7033
	22	23	24
	A	A	A
Frequencies --	1166.9910	1222.8651	1241.3209
Red. masses --	1.6165	2.1094	1.7107
Frc consts --	1.2971	1.8585	1.5531
IR Inten --	8.2303	101.0213	14.5837

	25	26	27
	A	A	A
Frequencies --	1275.6548	1301.2089	1331.7114
Red. masses --	1.4366	1.3282	1.3901
Frc consts --	1.3774	1.3250	1.4525
IR Inten --	33.6992	53.9990	19.9205

	28	29	30
	A	A	A
Frequencies --	1341.9703	1367.9623	1375.1785
Red. masses --	1.3876	1.4147	1.4753
Frc consts --	1.4723	1.5598	1.6438
IR Inten --	6.7325	5.2958	22.5197

	31	32	33
	A	A	A
Frequencies --	1395.0227	1416.3914	1466.2011
Red. masses --	1.5202	1.7212	1.0892
Frc consts --	1.7431	2.0344	1.3796
IR Inten --	4.7386	31.6261	12.3833

	34	35	36
	A	A	A
Frequencies --	1479.5268	1487.9690	1497.9014
Red. masses --	1.1356	1.0819	1.1009
Frc consts --	1.4646	1.4113	1.4553
IR Inten --	17.5408	9.1076	7.8651

	37	38	39
	A	A	A
Frequencies --	3051.8481	3057.7578	3061.7347
Red. masses --	1.0673	1.0632	1.0635
Frc consts --	5.8569	5.8568	5.8740
IR Inten --	14.6055	35.6995	20.1572

	40	41	42
	A	A	A
Frequencies --	3067.7488	3112.1399	3115.4038
Red. masses --	1.0628	1.0981	1.1015
Frc consts --	5.8930	6.2664	6.2987
IR Inten --	38.6709	43.2793	33.1352

	43	44	45
	A	A	A
Frequencies --	3121.4282	3140.0714	3146.8786
Red. masses --	1.1047	1.0891	1.0986
Frc consts --	6.3415	6.3272	6.4096
IR Inten --	58.6752	28.7357	12.4769

22e

	1	2	3
	A	A	A
Frequencies --	79.4454	151.7083	240.8727
Red. masses --	4.5372	2.6474	14.0987
Frc consts --	0.0169	0.0359	0.4820
IR Inten --	0.0983	2.3489	1.3424

	4	5	6
	A	A	A
Frequencies --	288.5819	351.8323	376.1327
Red. masses --	2.0556	3.1232	3.8686
Frc consts --	0.1009	0.2278	0.3225
IR Inten --	0.3955	5.5376	1.7851

	7	8	9
	A	A	A
Frequencies --	413.0449	482.8470	569.5170
Red. masses --	3.0324	2.8708	3.8276
Frc consts --	0.3048	0.3943	0.7315
IR Inten --	1.0345	5.7302	13.9385

```
                     10                     11                     12
                      A                      A                      A
Frequencies --    614.6591               732.9574               828.5342
Red. masses --      2.8124                 3.0579                 1.6598
Frc consts  --      0.6260                 0.9679                 0.6713
IR Inten    --      9.0489                 2.3740                14.6188
```

```
                     13                     14                     15
                      A                      A                      A
Frequencies --    859.5106               884.6214               934.0559
Red. masses --      2.0657                 2.1812                 1.5390
Frc consts  --      0.8991                 1.0057                 0.7911
IR Inten    --     11.9973                18.9259                 1.3446
                     16                     17                     18
                      A                      A                      A
Frequencies --    963.5457              1044.3984              1068.1597
Red. masses --      2.4606                 1.9431                 1.8303
Frc consts  --      1.3460                 1.2488                 1.2304
IR Inten    --     12.7243                38.2681                30.1433
                     19                     20                     21
                      A                      A                      A
Frequencies --   1090.5756              1096.5177              1133.4900
Red. masses --      1.7554                 2.6612                 1.8357
Frc consts  --      1.2301                 1.8852                 1.3896
IR Inten    --     32.6921                91.8451                 5.5578
                     22                     23                     24
                      A                      A                      A
Frequencies --   1144.7937              1212.0599              1258.2806
Red. masses --      2.0915                 2.0314                 1.4370
Frc consts  --      1.6149                 1.7583                 1.3404
IR Inten    --      1.4396                45.4394                 2.6003
                     25                     26                     27
                      A                      A                      A
Frequencies --   1274.4245              1313.9055              1323.1992
Red. masses --      1.3991                 1.3945                 1.4675
Frc consts  --      1.3388                 1.4184                 1.5138
IR Inten    --     30.1194                72.6272                11.0220
                     28                     29                     30
                      A                      A                      A
Frequencies --   1348.5798              1356.7785              1382.6229
Red. masses --      1.5116                 1.4282                 1.4707
Frc consts  --      1.6198                 1.5490                 1.6565
IR Inten    --     21.5616                67.2887                22.8604
                     31                     32                     33
                      A                      A                      A
Frequencies --   1391.6327              1404.0648              1478.3039
Red. masses --      1.4544                 1.5813                 1.1084
Frc consts  --      1.6596                 1.8367                 1.4272
IR Inten    --     18.6431                14.6952                 9.5781
                     34                     35                     36
                      A                      A                      A
Frequencies --   1485.1122              1491.1880              1503.6657
Red. masses --      1.0868                 1.0979                 1.0970
Frc consts  --      1.4123                 1.4384                 1.4614
IR Inten    --     21.0174                 5.3525                 7.1713
                     37                     38                     39
                      A                      A                      A
Frequencies --   3042.8749              3054.5638              3058.4053
Red. masses --      1.0684                 1.0700                 1.0655
Frc consts  --      5.8282                 5.8822                 5.8720
IR Inten    --      5.4838                26.1520                26.9117
                     40                     41                     42
```

	A	A	A
Frequencies --	3059.7445	3066.7364	3117.2502
Red. masses --	1.0650	1.0757	1.0962
Frc consts --	5.8744	5.9607	6.2760
IR Inten --	32.3827	53.9667	46.1022

	43	44	45
	A	A	A
Frequencies --	3120.4216	3126.6796	3139.5372
Red. masses --	1.1014	1.1033	1.0972
Frc consts --	6.3189	6.3550	6.3720
IR Inten --	46.3634	48.1642	16.4084

23a

	1	2	3
	A	A	A
Frequencies --	101.3503	152.6902	236.3914
Red. masses --	4.2064	3.0933	4.1580
Frc consts --	0.0255	0.0425	0.1369
IR Inten --	4.4886	1.3386	8.5445

	4	5	6
	A	A	A
Frequencies --	286.9464	374.5495	429.8574
Red. masses --	2.4268	2.0379	3.6273
Frc consts --	0.1177	0.1684	0.3949
IR Inten --	2.8141	3.4492	14.2560

	7	8	9
	A	A	A
Frequencies --	462.5456	491.7234	626.7545
Red. masses --	2.8530	2.4033	3.6401
Frc consts --	0.3596	0.3424	0.8425
IR Inten --	5.6420	19.0916	8.9218

	10	11	12
	A	A	A
Frequencies --	685.8283	772.3640	818.9751
Red. masses --	2.5024	3.0258	1.5545
Frc consts --	0.6935	1.0635	0.6143
IR Inten --	3.8297	9.3862	6.3295

	13	14	15
	A	A	A
Frequencies --	858.4601	893.6147	924.8834
Red. masses --	2.5619	1.9190	1.8038
Frc consts --	1.1124	0.9029	0.9091
IR Inten --	11.6955	5.0417	203.8745

	16	17	18
	A	A	A
Frequencies --	936.8132	986.5306	1010.4330
Red. masses --	1.6695	1.8079	2.7493
Frc consts --	0.8633	1.0367	1.6538
IR Inten --	36.1233	10.7255	23.9762

	19	20	21
	A	A	A
Frequencies --	1050.5253	1086.6575	1105.0565
Red. masses --	1.8258	1.4464	2.1673
Frc consts --	1.1872	1.0063	1.5593
IR Inten --	7.5776	11.8851	23.9292

	22	23	24
	A	A	A
Frequencies --	1145.1079	1176.9557	1197.4827
Red. masses --	1.7811	1.3126	1.2680
Frc consts --	1.3760	1.0713	1.0713
IR Inten --	61.0047	12.7585	25.7612

	25	26	27
	A	A	A
Frequencies --	1271.9793	1287.7044	1343.0414
Red. masses --	1.2852	1.2694	1.2789
Frc consts --	1.2251	1.2401	1.3592
IR Inten --	8.8657	2.8760	14.5561

	28	29	30
	A	A	A
Frequencies --	1346.0984	1367.2652	1373.5554
Red. masses --	1.3527	1.5047	1.3661
Frc consts --	1.4442	1.6573	1.5185
IR Inten --	9.0091	6.5564	14.5611

	31	32	33
	A	A	A
Frequencies --	1391.9955	1393.2074	1421.6861
Red. masses --	1.5264	1.4817	1.9483
Frc consts --	1.7426	1.6945	2.3202
IR Inten --	7.0148	0.4486	20.6711
	34	35	36
	A	A	A
Frequencies --	1465.2926	1468.6920	1493.2541
Red. masses --	1.0981	1.1333	1.0841
Frc consts --	1.3892	1.4403	1.4242
IR Inten --	2.4158	34.4301	11.1006
	37	38	39
	A	A	A
Frequencies --	1502.0147	1751.0600	3044.1235
Red. masses --	1.0995	9.1812	1.0672
Frc consts --	1.4615	16.5864	5.8269
IR Inten --	5.8823	153.7269	38.7794
	40	41	42
	A	A	A
Frequencies --	3058.9360	3060.2717	3060.8513
Red. masses --	1.0654	1.0639	1.0628
Frc consts --	5.8737	5.8706	5.8664
IR Inten --	16.6927	33.0741	32.6928
	43	44	45
	A	A	A
Frequencies --	3104.6651	3106.5181	3111.9376
Red. masses --	1.0901	1.0967	1.0975
Frc consts --	6.1907	6.2355	6.2621
IR Inten --	95.5403	15.0387	50.1442
	46	47	48
	A	A	A
Frequencies --	3116.3545	3135.5055	3482.6552
Red. masses --	1.1037	1.0995	1.0744
Frc consts --	6.3151	6.3691	7.6777
IR Inten --	52.2466	27.9561	0.8745

23e

	1	2	3
	A	A	A
Frequencies --	110.7049	158.0871	289.1920
Red. masses --	4.0316	2.4600	4.8132
Frc consts --	0.0291	0.0362	0.2372
IR Inten --	0.0710	1.9318	8.5799
	4	5	6
	A	A	A
Frequencies --	314.0729	355.4461	393.4042
Red. masses --	1.9287	3.4348	4.5619
Frc consts --	0.1121	0.2557	0.4160
IR Inten --	2.1682	15.0840	0.2934
	7	8	9
	A	A	A
Frequencies --	427.1427	501.9294	588.1792
Red. masses --	2.7431	2.2650	3.5721
Frc consts --	0.2949	0.3362	0.7281
IR Inten --	3.5552	3.0692	37.2475

	10	11	12
	A	A	A
Frequencies --	669.4544	768.0273	847.7167
Red. masses --	2.6428	2.3048	1.6377
Frc consts --	0.6979	0.8010	0.6934
IR Inten --	2.1585	5.7717	30.1576

	13	14	15
	A	A	A
Frequencies --	868.2908	888.4306	902.0707
Red. masses --	2.1535	1.8756	1.4930
Frc consts --	0.9566	0.8723	0.7158
IR Inten --	10.6486	47.7259	46.2951
	16	17	18
	A	A	A
Frequencies --	969.5360	982.4248	1057.0021
Red. masses --	2.4198	1.3214	2.2212
Frc consts --	1.3401	0.7514	1.4622
IR Inten --	4.8650	40.2748	92.1209
	19	20	21
	A	A	A
Frequencies --	1070.4529	1084.4323	1101.1680
Red. masses --	2.0550	1.9561	1.9744
Frc consts --	1.3874	1.3553	1.4106
IR Inten --	28.6786	33.7274	7.0674
	22	23	24
	A	A	A
Frequencies --	1135.8570	1171.1016	1214.3786
Red. masses --	2.0430	1.4902	1.3827
Frc consts --	1.5530	1.2042	1.2014
IR Inten --	16.3535	4.3426	19.9472
	25	26	27
	A	A	A
Frequencies --	1273.7646	1283.7449	1321.2924
Red. masses --	1.3384	1.2585	1.3295
Frc consts --	1.2794	1.2220	1.3675
IR Inten --	1.3064	6.7477	32.1903
	28	29	30
	A	A	A
Frequencies --	1344.0526	1365.7960	1367.8008
Red. masses --	1.1737	1.3748	1.4239
Frc consts --	1.2493	1.5109	1.5695
IR Inten --	3.4540	2.8054	2.9958
	31	32	33
	A	A	A
Frequencies --	1383.2199	1396.3430	1424.3061
Red. masses --	1.5066	1.4640	1.8987
Frc consts --	1.6984	1.6818	2.2694
IR Inten --	17.2056	2.5409	23.3681
	34	35	36
	A	A	A
Frequencies --	1471.5846	1488.4589	1493.0033
Red. masses --	1.1078	1.1074	1.0907
Frc consts --	1.4134	1.4456	1.4325
IR Inten --	17.2899	10.3240	13.0951
	37	38	39
	A	A	A
Frequencies --	1503.0833	1752.2032	3046.0432
Red. masses --	1.0996	10.0710	1.0695
Frc consts --	1.4638	18.2175	5.8464
IR Inten --	4.6724	198.0567	13.0381
	40	41	42
	A	A	A

Frequencies --	3049.8665	3053.5346	3058.2352
Red. masses --	1.0677	1.0629	1.0634
Frc consts --	5.8514	5.8393	5.8600
IR Inten --	22.0049	63.1569	29.5143
	43	44	45
	A	A	A
Frequencies --	3075.8119	3111.9645	3114.9372
Red. masses --	1.0825	1.0976	1.0990
Frc consts --	6.0338	6.2627	6.2825
IR Inten --	36.7900	55.6860	56.6046
	46	47	48
	A	A	A
Frequencies --	3123.0647	3131.9387	3488.9552
Red. masses --	1.1030	1.0977	1.0732
Frc consts --	6.3384	6.3440	7.6971
IR Inten --	53.4488	28.5395	8.2339

24a

	1	2	3
	A	A	A
Frequencies --	101.3645	156.2007	237.1415
Red. masses --	4.2667	3.1553	3.7013
Frc consts --	0.0258	0.0454	0.1226
IR Inten --	4.8802	1.3623	6.9263
	4	5	6
	A	A	A
Frequencies --	288.9864	374.1964	431.5744
Red. masses --	2.4367	2.0431	4.3059
Frc consts --	0.1199	0.1686	0.4725
IR Inten --	4.9718	2.1494	8.6682
	7	8	9
	A	A	A
Frequencies --	460.2427	488.5875	631.4663
Red. masses --	2.8510	2.1865	3.5734
Frc consts --	0.3558	0.3075	0.8395
IR Inten --	7.5548	13.9083	9.0218
	10	11	12
	A	A	A
Frequencies --	690.2650	768.4771	822.0974
Red. masses --	2.6880	2.9573	1.6522
Frc consts --	0.7546	1.0290	0.6579
IR Inten --	3.1114	7.4585	12.3662
	13	14	15
	A	A	A
Frequencies --	857.2282	889.7706	925.2831
Red. masses --	2.4105	1.7524	1.9093
Frc consts --	1.0436	0.8174	0.9631
IR Inten --	1.6887	36.8012	35.7449
	16	17	18
	A	A	A
Frequencies --	933.7002	990.1084	1011.6146
Red. masses --	1.6707	1.7525	2.7378
Frc consts --	0.8582	1.0122	1.6508
IR Inten --	28.3374	106.7407	58.7152
	19	20	21
	A	A	A
Frequencies --	1051.3685	1089.3292	1104.8187
Red. masses --	1.8474	1.5120	2.2564
Frc consts --	1.2032	1.0571	1.6228
IR Inten --	17.2784	13.6586	37.4264
	22	23	24
	A	A	A
Frequencies --	1144.2769	1178.7622	1198.7820
Red. masses --	1.5567	1.4009	1.2514
Frc consts --	1.2009	1.1469	1.0595
IR Inten --	15.8681	6.0319	25.4013

	25	26	27
	A	A	A
Frequencies --	1270.3263	1288.5459	1344.5580
Red. masses --	1.2942	1.2446	1.3220
Frc consts --	1.2305	1.2175	1.4081
IR Inten --	0.1410	2.4957	3.7638

	28	29	30
	A	A	A
Frequencies --	1350.9392	1363.5770	1374.4554
Red. masses --	1.3156	1.3546	1.4638
Frc consts --	1.4146	1.4839	1.6293
IR Inten --	6.3701	9.7106	20.1673

	31	32	33
	A	A	A
Frequencies --	1395.1033	1396.1431	1425.4189
Red. masses --	1.4708	1.5661	2.2064
Frc consts --	1.6866	1.7985	2.6413
IR Inten --	3.2636	1.8978	77.9709

	34	35	36
	A	A	A
Frequencies --	1467.1782	1468.8636	1493.7125
Red. masses --	1.0999	1.1025	1.0854
Frc consts --	1.3950	1.4015	1.4268
IR Inten --	7.8845	31.1428	8.0813

	37	38	39
	A	A	A
Frequencies --	1501.9331	1752.5213	3044.1400
Red. masses --	1.0983	9.1867	1.0675
Frc consts --	1.4597	16.6241	5.8285
IR Inten --	6.8319	154.5221	38.4091

	40	41	42
	A	A	A
Frequencies --	3058.3544	3058.7338	3060.4010
Red. masses --	1.0647	1.0618	1.0621
Frc consts --	5.8674	5.8532	5.8608
IR Inten --	27.3729	16.8821	42.6886

	43	44	45
	A	A	A
Frequencies --	3106.9167	3109.4724	3115.5376
Red. masses --	1.0961	1.1007	1.1027
Frc consts --	6.2338	6.2706	6.3065
IR Inten --	56.1480	55.1206	38.4758

	46	47	48
	A	A	A
Frequencies --	3119.3027	3129.7378	3486.0369
Red. masses --	1.1033	1.0889	1.0743
Frc consts --	6.3251	6.2842	7.6920
IR Inten --	45.9462	34.8613	1.5376

24e

	1	2	3
	A	A	A
Frequencies --	100.3533	161.3729	299.6674
Red. masses --	4.2879	2.4929	4.7060
Frc consts --	0.0254	0.0382	0.2490
IR Inten --	0.5734	4.5474	0.1062

	4	5	6
	A	A	A
Frequencies --	315.5065	352.2852	395.8687
Red. masses --	2.0178	3.3528	4.4471
Frc consts --	0.1183	0.2452	0.4106
IR Inten --	0.2089	1.4710	7.7939

```
                      7                      8                      9
                      A                      A                      A
Frequencies --    429.3925               507.1764               586.5205
Red. masses --      2.8141                 2.2475                 3.4180
Frc consts  --      0.3057                 0.3406                 0.6928
IR Inten    --      0.6530                12.7069                28.7735



                     10                     11                     12
                      A                      A                      A
Frequencies --    670.7833               775.9939               850.5048
Red. masses --      2.7100                 2.2438                 1.7343
Frc consts  --      0.7184                 0.7961                 0.7391
IR Inten    --      2.7249                 1.1480                12.7110

                     13                     14                     15
                      A                      A                      A
Frequencies --    870.2718               890.1072               895.0580
Red. masses --      1.7924                 1.5893                 2.0576
Frc consts  --      0.7998                 0.7419                 0.9712
IR Inten    --     29.8248                30.6342                28.4634

                     16                     17                     18
                      A                      A                      A
Frequencies --    970.3193               980.0178              1062.8605
Red. masses --      2.2104                 1.4110                 1.8186
Frc consts  --      1.2262                 0.7984                 1.2104
IR Inten    --     24.4551                38.9713                34.8941

                     19                     20                     21
                      A                      A                      A
Frequencies --   1077.8371              1088.3317              1102.4056
Red. masses --      2.1570                 2.4354                 2.1305
Frc consts  --      1.4764                 1.6996                 1.5255
IR Inten    --     24.2903               125.7000                32.0246

                     22                     23                     24
                      A                      A                      A
Frequencies --   1134.8370              1169.7037              1207.1571
Red. masses --      1.9295                 1.4819                 1.2954
Frc consts  --      1.4641                 1.1946                 1.1122
IR Inten    --      5.1604                12.4546                21.2954

                     25                     26                     27
                      A                      A                      A
Frequencies --   1273.5370              1285.0970              1334.9203
Red. masses --      1.3443                 1.2507                 1.2927
Frc consts  --      1.2846                 1.2170                 1.3572
IR Inten    --      0.7836                 1.1339                12.9174

                     28                     29                     30
                      A                      A                      A
Frequencies --   1353.0462              1362.7945              1368.1347
Red. masses --      1.2048                 1.3716                 1.4140
Frc consts  --      1.2996                 1.5008                 1.5594
IR Inten    --     24.4908                 8.6666                 3.0356

                     31                     32                     33
                      A                      A                      A
Frequencies --   1382.4567              1397.5723              1436.1398
Red. masses --      1.5464                 1.4703                 1.8207
Frc consts  --      1.7413                 1.6920                 2.2126
IR Inten    --     14.0860                 1.3084                16.1827

                     34                     35                     36
                      A                      A                      A
Frequencies --   1479.1338              1487.6303              1494.4426
Red. masses --      1.1011                 1.1028                 1.0948
Frc consts  --      1.4194                 1.4380                 1.4406
IR Inten    --     23.5671                 6.9143                15.4881

                     37                     38                     39
                      A                      A                      A
```

```
 Frequencies --   1504.1161              1753.6432              3046.2885
 Red. masses --      1.1016                 9.5319                 1.0672
 Frc consts  --      1.4683                17.2708                 5.8351
 IR Inten    --      2.7419               190.7894                13.1581

                     40                     41                     42
                      A                      A                      A
 Frequencies --   3049.9050              3054.4122              3055.6871
 Red. masses --      1.0690                 1.0645                 1.0644
 Frc consts  --      5.8587                 5.8510                 5.8556
 IR Inten    --     25.4770                28.3552                36.8274

                     43                     44                     45
                      A                      A                      A
 Frequencies --   3064.1856              3112.3030              3114.5661
 Red. masses --      1.0783                 1.0974                 1.0984
 Frc consts  --      5.9649                 6.2630                 6.2779
 IR Inten    --     63.4004                52.6388                60.7210

                     46                     47                     48
                      A                      A                      A
 Frequencies --   3118.6537              3121.4430              3496.4271
 Red. masses --      1.1008                 1.1030                 1.0743
 Frc consts  --      6.3081                 6.3322                 7.7381
 IR Inten    --     26.9535                61.8374                 1.8493
```

25a

```
                      1                      2                      3
                      A                      A                      A
 Frequencies --    111.7562               159.8149               235.7105
 Red. masses --      3.4420                 3.0324                 3.4841
 Frc consts  --      0.0253                 0.0456                 0.1141
 IR Inten    --      1.1432                 2.8309                 6.2133

                      4                      5                      6
                      A                      A                      A
 Frequencies --    288.6735               372.8275               401.5685
 Red. masses --      2.3235                 2.0077                 2.4986
 Frc consts  --      0.1141                 0.1644                 0.2374
 IR Inten    --      1.9559                 3.3384                 1.3488

                      7                      8                      9
                      A                      A                      A
 Frequencies --    454.9616               472.7757               627.1738
 Red. masses --      3.4016                 2.0170                 4.1580
 Frc consts  --      0.4148                 0.2656                 0.9636
 IR Inten    --      3.6948                 5.8941                 4.0442

                     10                     11                     12
                      A                      A                      A
 Frequencies --    662.9997               743.4937               782.4418
 Red. masses --      2.2880                 1.2573                 2.2210
 Frc consts  --      0.5926                 0.4095                 0.8011
 IR Inten    --     12.2342                 4.0245                 4.1547

                     13                     14                     15
                      A                      A                      A
 Frequencies --    821.3493               866.3992               886.7568
 Red. masses --      1.7714                 2.5147                 2.2646
 Frc consts  --      0.7041                 1.1122                 1.0492
 IR Inten    --     16.2669                 6.0646                60.4916

                     16                     17                     18
                      A                      A                      A
 Frequencies --    918.0352               955.5894               967.7950
 Red. masses --      1.6097                 2.1623                 1.3906
 Frc consts  --      0.7993                 1.1633                 0.7674
 IR Inten    --     38.7827                56.6822               105.1978

                     19                     20                     21
                      A                      A                      A
 Frequencies --   1000.8618              1009.1885              1056.2365
 Red. masses --      1.6760                 2.4633                 1.7935
 Frc consts  --      0.9892                 1.4781                 1.1789
 IR Inten    --     23.3180                16.9089                 4.9976
```

```
                     22                     23                     24
                      A                      A                      A
Frequencies --   1095.9723              1111.4064              1177.5467
Red. masses --      1.6768                 1.7238                 1.2197
Frc consts  --      1.1867                 1.2545                 0.9965
IR Inten    --     15.1722                 8.8164                16.2292



                     25                     26                     27
                      A                      A                      A
Frequencies --   1188.1527              1271.7383              1290.5853
Red. masses --      1.5572                 1.3451                 1.2283
Frc consts  --      1.2952                 1.2818                 1.2054
IR Inten    --      7.6762                 0.2332                 1.8686

                     28                     29                     30
                      A                      A                      A
Frequencies --   1322.9748              1347.4438              1359.8126
Red. masses --      1.5835                 1.2712                 1.3181
Frc consts  --      1.6330                 1.3598                 1.4361
IR Inten    --      5.0591                10.4311                 7.2006

                     31                     32                     33
                      A                      A                      A
Frequencies --   1372.2000              1380.1766              1392.8627
Red. masses --      1.6303                 1.5931                 1.6044
Frc consts  --      1.8086                 1.7880                 1.8340
IR Inten    --      9.8520                 7.7542                 1.6274

                     34                     35                     36
                      A                      A                      A
Frequencies --   1397.7001              1458.9447              1466.5458
Red. masses --      1.5845                 1.1581                 1.0969
Frc consts  --      1.8238                 1.4523                 1.3899
IR Inten    --      1.7805                 9.0477                14.0024

                     37                     38                     39
                      A                      A                      A
Frequencies --   1478.4911              1490.3937              1499.9898
Red. masses --      1.1292                 1.0865                 1.0979
Frc consts  --      1.4543                 1.4220                 1.4554
IR Inten    --     22.0507                 9.6359                 5.5735

                     40                     41                     42
                      A                      A                      A
Frequencies --   1741.5152              3036.8493              3046.5608
Red. masses --      5.9672                 1.0674                 1.0638
Frc consts  --     10.6629                 5.8000                 5.8176
IR Inten    --     20.6894                39.5019                42.3377

                     43                     44                     45
                      A                      A                      A
Frequencies --   3055.4985              3056.8701              3098.5279
Red. masses --      1.0629                 1.0638                 1.0963
Frc consts  --      5.8465                 5.8569                 6.2012
IR Inten    --     43.4254                23.4454                93.2327

                     46                     47                     48
                      A                      A                      A
Frequencies --   3103.2739              3107.5636              3111.3460
Red. masses --      1.0994                 1.0911                 1.1018
Frc consts  --      6.2380                 6.2081                 6.2840
IR Inten    --     50.8434                59.7266                53.6967

                     49                     50                     51
                      A                      A                      A
Frequencies --   3112.4408              3143.6409              3231.4614
Red. masses --      1.0990                 1.0619                 1.1165
Frc consts  --      6.2729                 6.1831                 6.8690
IR Inten    --     47.2604                13.7515                17.8591
```

25e

	1	2	3
	A	A	A
Frequencies --	113.9245	170.2063	288.5704
Red. masses --	3.3272	2.4477	3.7139
Frc consts --	0.0254	0.0418	0.1822
IR Inten --	1.8801	0.7615	3.9630

	4	5	6
	A	A	A
Frequencies --	311.5742	353.7252	389.2068
Red. masses --	1.9215	3.0512	3.6415
Frc consts --	0.1099	0.2249	0.3250
IR Inten --	1.8785	2.9756	2.2338

	7	8	9
	A	A	A
Frequencies --	426.2422	481.5325	562.7025
Red. masses --	2.5842	2.4982	2.9426
Frc consts --	0.2766	0.3413	0.5490
IR Inten --	0.8439	4.1495	18.6021

	10	11	12
	A	A	A
Frequencies --	642.6875	746.3475	788.7642
Red. masses --	2.3663	1.1571	2.0573
Frc consts --	0.5759	0.3798	0.7541
IR Inten --	10.0018	0.6875	2.0463

	13	14	15
	A	A	A
Frequencies --	843.3164	876.0761	878.9650
Red. masses --	1.7049	1.6594	2.5304
Frc consts --	0.7144	0.7504	1.1518
IR Inten --	15.0028	25.3906	0.6248

	16	17	18
	A	A	A
Frequencies --	945.5742	952.9109	960.5092
Red. masses --	1.4086	1.8140	1.7683
Frc consts --	0.7421	0.9705	0.9612
IR Inten --	73.1028	15.2529	25.8919

	19	20	21
	A	A	A
Frequencies --	1001.7785	1053.4807	1074.3170
Red. masses --	1.6556	3.1814	1.9399
Frc consts --	0.9789	2.0803	1.3192
IR Inten --	15.7925	113.2811	29.7297

	22	23	24
	A	A	A
Frequencies --	1095.3930	1121.8996	1170.3922
Red. masses --	1.8574	1.7047	1.5191
Frc consts --	1.3131	1.2642	1.2260
IR Inten --	2.9154	3.5958	3.8242

	25	26	27
	A	A	A
Frequencies --	1182.8219	1273.2714	1276.0966
Red. masses --	1.6013	1.4520	1.3550
Frc consts --	1.3199	1.3869	1.3000
IR Inten --	1.6080	0.7277	3.3930

	28	29	30
	A	A	A
Frequencies --	1291.8085	1341.7828	1362.2322
Red. masses --	1.3565	1.2087	1.3764
Frc consts --	1.3337	1.2821	1.5049
IR Inten --	0.2140	0.2867	1.2822

	31	32	33
	A	A	A

```
 Frequencies --   1368.9279              1381.1097              1393.8764
 Red. masses --      1.4486                 1.5468                 1.4763
 Frc consts  --      1.5994                 1.7384                 1.6900
 IR Inten    --      2.3334                 4.6535                 0.5154

                     34                     35                     36
                      A                      A                      A
 Frequencies --   1403.8104              1445.2560              1482.0723
 Red. masses --      1.6054                 1.1792                 1.1035
 Frc consts  --      1.8641                 1.4512                 1.4282
 IR Inten    --     17.5523                 7.1633                13.3818

                     37                     38                     39
                      A                      A                      A
 Frequencies --   1485.7687              1491.7290              1502.2495
 Red. masses --      1.1017                 1.0921                 1.0990
 Frc consts  --      1.4329                 1.4318                 1.4613
 IR Inten    --     11.2875                13.0993                 3.8027

                     40                     41                     42
                      A                      A                      A
 Frequencies --   1742.1081              3032.3361              3042.5620
 Red. masses --      6.3443                 1.0677                 1.0685
 Frc consts  --     11.3446                 5.7843                 5.8280
 IR Inten    --     40.3659                32.5326                31.3023

                     43                     44                     45
                      A                      A                      A
 Frequencies --   3047.3955              3051.1688              3057.9174
 Red. masses --      1.0625                 1.0724                 1.0729
 Frc consts  --      5.8134                 5.8824                 5.9111
 IR Inten    --     65.2767                 6.6103                76.5911

                     46                     47                     48
                      A                      A                      A
 Frequencies --   3103.5704              3105.5678              3109.6410
 Red. masses --      1.0972                 1.0982                 1.0988
 Frc consts  --      6.2265                 6.2407                 6.2605
 IR Inten    --     71.2010                72.8457                41.6269

                     49                     50                     51
                      A                      A                      A
 Frequencies --   3115.4625              3158.7340              3252.1591
 Red. masses --      1.1035                 1.0616                 1.1158
 Frc consts  --      6.3106                 6.2406                 6.9534
 IR Inten    --     70.6799                11.5214                 9.6845
```
